# lncRNAs GAS5 and MALAT1 Contained in Human Adipose Stem Cell (hASC)-Derived Exosomes Drive the Cell-Free Repair and Regeneration of Wounds In Vivo

**DOI:** 10.3390/ijms26083479

**Published:** 2025-04-08

**Authors:** Meredith Krause-Hauch, Rekha S. Patel, Bangmei Wang, Brenna Osborne, Brianna Jones, Paul Albear, Niketa A. Patel

**Affiliations:** 1 Research Service, James A. Haley Veterans’ Hospital, 13000 Bruce B Downs Blvd, Tampa, FL 33612, USA; meredith.krause-hauch@va.gov (M.K.-H.); rekha.patel1@va.gov (R.S.P.); bangmei.wang@va.gov (B.W.); paul.albear@va.gov (P.A.); 2 Department of Molecular Medicine, University of South Florida, Tampa, FL 33612, USA; brennao@usf.edu; 3 Department of Chemistry, University of South Florida, Tampa, FL 33612, USA; bmjones1@usf.edu

**Keywords:** small extracellular vesicles, exosomes, wound healing, *Gas5*, *Malat1*, adipose stem cells, RNAseq

## Abstract

Wound healing progresses through four phases: hemostasis, inflammation, proliferation, and remodeling. Wounds may become chronic if this process is disrupted. The use of small extracellular vesicle (sEV; EVs < 200 nm) exosomes (exo; ~40–120 nm) derived from human adipose stem cells (hASCs) as a treatment for wounds is well studied. The cargo of these exosomes is of great interest as this accelerates wound healing. Our previous studies identified lncRNAs *GAS5* and *MALAT1* as packaged and enriched in hASC exosomes. In this study, we use a rat model to examine the effects on wound healing when hASC exosomes are depleted of *GAS5* and *MALAT1*. Rats were wounded and wounds were treated with 100 μg hASC_exo_ or hASC_exo_-G-M every 2 days for 1 week. qPCR was completed to evaluate the molecular effects of depletion of *GAS5* and *MALAT1* from hASC_exo_. RNAseq was performed on wound tissue to evaluate the molecular mechanisms changed by hASC_exo_-G-M in wound healing. While hASC_exo_-G-M significantly improved wound healing rate compared to control wounds, healing occurred slower than in wounds treated with hASC_exo_ that were not depleted of *GAS5* and *MALAT1*. Overall, this study reveals that molecular functions associated with healing are reduced in the absence of *GAS5* and *MALAT1*, highlighting the importance of these lncRNAs.

## 1. Introduction

Wound healing is a complex, multifactorial process that is essential to tissue repair and regeneration. Typical wound healing progresses through four overlapping phases: hemostasis, inflammation, proliferation, and remodeling [1,2]. Clinically, chronic wounds—commonly observed in conditions such as diabetes and aging—pose a significant challenge to healthcare systems globally. These wounds fail to progress through the normal stages of healing, leading to prolonged suffering, infections, and sometimes, amputations [2]. It is estimated that approximately 2% of the world’s population will be affected by chronic wounds during their lifetime [3]. Chronic wounds are common among both diabetic and elderly populations, both of which are prevalent within the U.S. Veteran community. The U.S. Department of Veterans’ Affairs estimates that approximately 25% of the VA’s patient population is affected by diabetes. Further, as of 2007, approximately 15% of diabetic patients struggle with chronic diabetic foot ulcers, accounting for over 80% of all diabetes-related lower leg amputations [4]. As the number of individuals affected by chronic wounds continues to rise, especially within vulnerable populations like the elderly and those with diabetes, novel therapeutic strategies are desperately needed. One significant factor that leads to chronic wounds in these patients is the senescence of cells, which in turn results in excessive inflammation [1]. Previous studies have indicated that chronic wounds have an excess of pro-inflammatory cytokines [5]. This imbalance in the ideal ratio of pro- and anti-inflammatory factors within the wound prevents it from progressing into the proliferation phase [6].

One method for cell-to-cell communication to occur includes the secretion of extracellular vesicles (EVs) [7] that will communicate with other cells via surface proteins and cargo molecules [8]. EVs are generally classified as apoptotic bodies, microvesicles, or exosomes [9]. EVs may be classified by size. Small EVs (sEVs) are classified as extracellular vesicles that are <200 nm in diameter and this includes exosomes that are typically classified as being ~40–120 nm in diameter [10]. There are no universally agreed-upon size parameters for classifying EVs, so biogenesis origins have become more commonly used for EV classification. Biogenically, exosomes are defined as internal cellular components that are released out of a cell when multivesicular bodies fuse with the plasma membrane [11]. Most types of cells release EVs, allowing for the transfer of cargo such as proteins, lipids, and nucleic acids from one cell to another, resulting in EV participation in various biological processes including cell motility, differentiation, proliferation, apoptosis, and immunity [12]. The ability for intercellular communication combined with the diverse cargo of EVs have made them a target of research for therapeutics of various diseases and conditions.

In recent years, stem cell-based therapies have emerged as promising treatments for wound healing due to their regenerative potential. Previous studies have pointed to mesenchymal stem cells (MSCs) and adipose-derived stem cells (ASCs) as ideal originator cells for exosomes used in wound healing treatments [13,14]. MSC exosomes have been shown to support wound healing by promoting epithelialization, collagen maturation, and scar reduction [15]. Human adipose-derived stem cells (hASCs) and their exosome-based derivatives have garnered attention for their ability to accelerate wound healing through a variety of mechanisms, including the modulation of inflammation and the promotion of cell proliferation and migration [16]. In addition, exosomes from hASCs have proangiogenic properties due to the presence of microRNA-31, which is beneficial in the promotion of dermal wound healing as well as in ischemic damage [17]. ASC_exos_ have also been investigated in musculoskeletal injury and conditions such as osteoarthritis. These osteogenic properties are thought to be due to the exosome surface proteins BMPR1b and CD90 [18]. In addition to adipocytes, adipose tissue is composed of other cell types including immune cells, and hASCs are known to secrete various growth factors (i.e., VEGF and NGF), interleukins, and cytokines [18]. These factors, combined with the ease and safety of hASC collection from adipose discarded during surgical procedures, make hASC_exos_ ideal for studies of regenerative healing.

This study explores the role of long non-coding RNAs (lncRNAs), specifically Growth arrest-specific 5 (*GAS5*) and metastasis-associated lung adenocarcinoma transcript 1 (*MALAT1*), within hASC-derived exosomes, highlighting their crucial roles in promoting wound repair and regeneration. Previously, we have demonstrated that *GAS5* and *MALAT1* are highly enriched in hASC_exo_ [19]. Further, our work has illuminated that separately *GAS5* and *MALAT1* each play a key role in wound healing when packaged inside hASC_exo_ in dermal wounds and traumatic brain injury (TBI), respectively [20]. We found that the depletion of either *GAS5* or *MALAT1* individually from hASC_exo_ resulted in delayed healing of wounds, indicating the importance of these lncRNAs to the wound healing process. Therefore, to follow up on these findings, in the present study we investigated the efficacy of hASC_exo_ depleted of both *GAS5* and *MALAT1* (hASC_exo_-G-M) and the impact on healing wounds in vivo. By depleting these lncRNAs from exosomes, this research demonstrates that the absence of *GAS5* and *MALAT1* leads to delayed wound healing, underscoring their clinical significance. These findings not only contribute to the understanding of wound healing at the molecular level but also pave the way for optimizing stem cell-based therapies for chronic wounds, offering hope for more effective treatments in clinical settings.

## 2. Results

### 2.1. Automatic Western Blotting and NTA Verify That Isolated sEVs Are Exosomes

**Figure 1:**
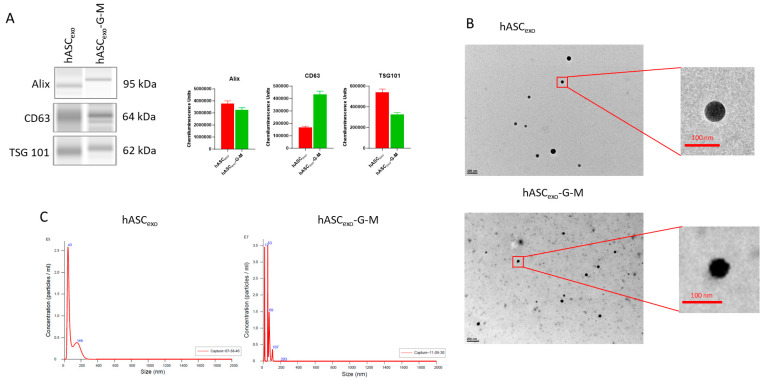
Descriptive morphology of hASC_exo_ and hASC_exo_-G-M. (**A**) Jess Automated Western Blot representative images for exosome surface markers Alix, CD 63, and TSG 101. The presence of these markers verifies that the EVs extracted are indeed exosomes. (**B**) Transmission Electron Microscope (TEM) images of hASC_exo_ and hASC_exo_-G-M at 60k× (hASC_exo_) and 50k× (hASC_exo_-G-M) magnification. (**C**) NanoSight particle size analysis.

Automatic Western blot was performed for tetraspanin CD63 and endosomal sorting complex required for transport (Escrt) proteins TSG101 and Apoptosis-linked gene 2-interacting protein X (Alix) to verify that the isolated sEVs are exosomes. CD63 was observed at a higher molecular weight suggesting glycosylation. The ESCRT proteins TSG101 and Alix are specific for exosome biogenesis. Alix delivers tetraspanins to exosomes. The markers were observed in both sEV samples, confirming that these sEVs are exosomes (Figure 1A).

Transmission electron microscopy (TEM) was used to visualize the exosomes and to evaluate them for any morphological differences. TEM imaging reveals no significant differences in morphology between hASC_exo_ and hASC_exo_-G-M (Figure 1B). Further, to determine the average size and concentration of exosomes, NanoSight was utilized to collect NTA data. The average diameter of hASC_exo_ was 87.6 ± 56.0 nm, while that of hASC_exo_-G-M was 51.1 ± 27.4 nm.

### 2.2. Biochemical Testing of hASC_exo_ and hASC_exo_-G-M Demonstrate Differences in Cargos

**Figure 2:**
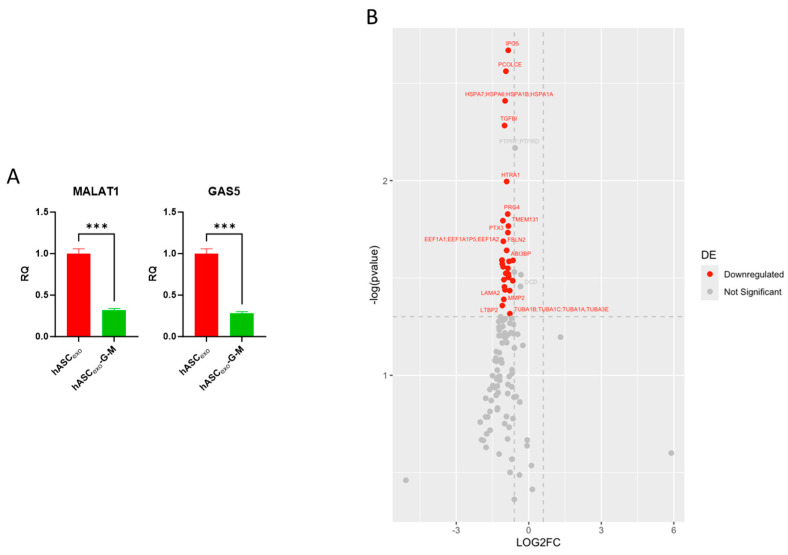
qPCR and proteomic analysis of hASC_exo_ and hASC_exo_-G-M cargo. (**A**) qPCR results for lncRNAs *GAS5* and *MALAT1* as cargo of hASC_exo_ and hASC_exo_-G-M. *** *p* < 0.001. Statistical analysis performed using GraphPad Prism SPSS Analysis Software V.10.02 to calculate unpaired Student’s *t*-test. RQ = Relative Quantification. (**B**) Volcano plot of proteomic results for hASCexo and hASCexo-G-M cargo. Red points = significantly downregulated proteins. Grey points = non-significant proteins. The chosen cut-off for significance was a Log2FC greater than 0.6 and less than −0.6 (i.e., a fold change greater than 1.5), and a *p*-value greater than 0.5. Figure created using R package ggplot2 (V.3.5.1).

qPCR analysis of *GAS5* and *MALAT1* levels in hASC_exo_ and hASC_exo_-G-M confirms that both lncRNAs are depleted by over two-fold in hASC_exo_-G-M compared to hASC_exo_ (Figure 2A).

Proteomics analysis of exosome cargo was completed to evaluate any cargo differences between hASC_exo_ and hASC_exo_-G-M. One hundred and seventeen total proteins were identified in the proteomics analysis. Thirty-one of these proteins were significantly downregulated with a fold change (FC) greater than 1.5 in hASC_exo_-G-M compared to that of hASC_exo_ (Figure 2B). The top 10 significant proteins with the greatest FC included FN1, COL1A2, PLTP, A2M, LTBP2, PTX3, FBN1, EEF1A1, MMP2, and RELN. A volcano plot was generated to visualize which exosome cargo proteins are altered in hASC_exo_-G-M compared to hASC_exo_. Since these proteins were downregulated in hASC_exo_-G-M compared to hASC_exo_, this indicated that the depletion of GAS5 and MALAT1 results in the depletion of some proteins packaged in exosomes.

### 2.3. GAS5 and MALAT1 Improve Wound Healing In Vitro

**Figure 3:**
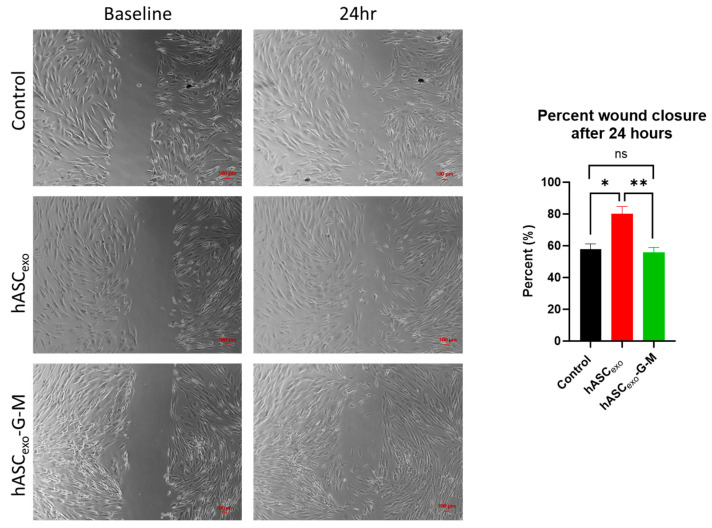
In vitro wound healing model. Scratch assay was performed using human dermal fibroblasts (HDF). Images were taken using a Keyence BZX-800 at baseline and at 24 h then analyzed for percent scratch closure. ns = not significant. * *p* < 0.05, ** *p* < 0.01. Statistical analysis performed using GraphPad Prism SPSS Analysis Software V.10.02 to calculate one-way ANOVA. Scale bar = 100 µm.

To evaluate the hASC_exo_ and hASC_exo_-G-M effects on wound healing, an in vitro scratch assay was performed first (Figure 3). After 24 h, scratches treated with hASC_exo_ were approximately 80% closed, while both untreated control scratches and scratches treated with hASC_exo_-G-M had closed approximately 55%. This indicates the importance of *GAS5* and *MALAT1* in hASC_exo_ for the promotion of wound healing.

### 2.4. The Knockdown of GAS5 and MALAT1 in hASC_exo_ Slows Wound Healing In Vivo

**Figure 4:**
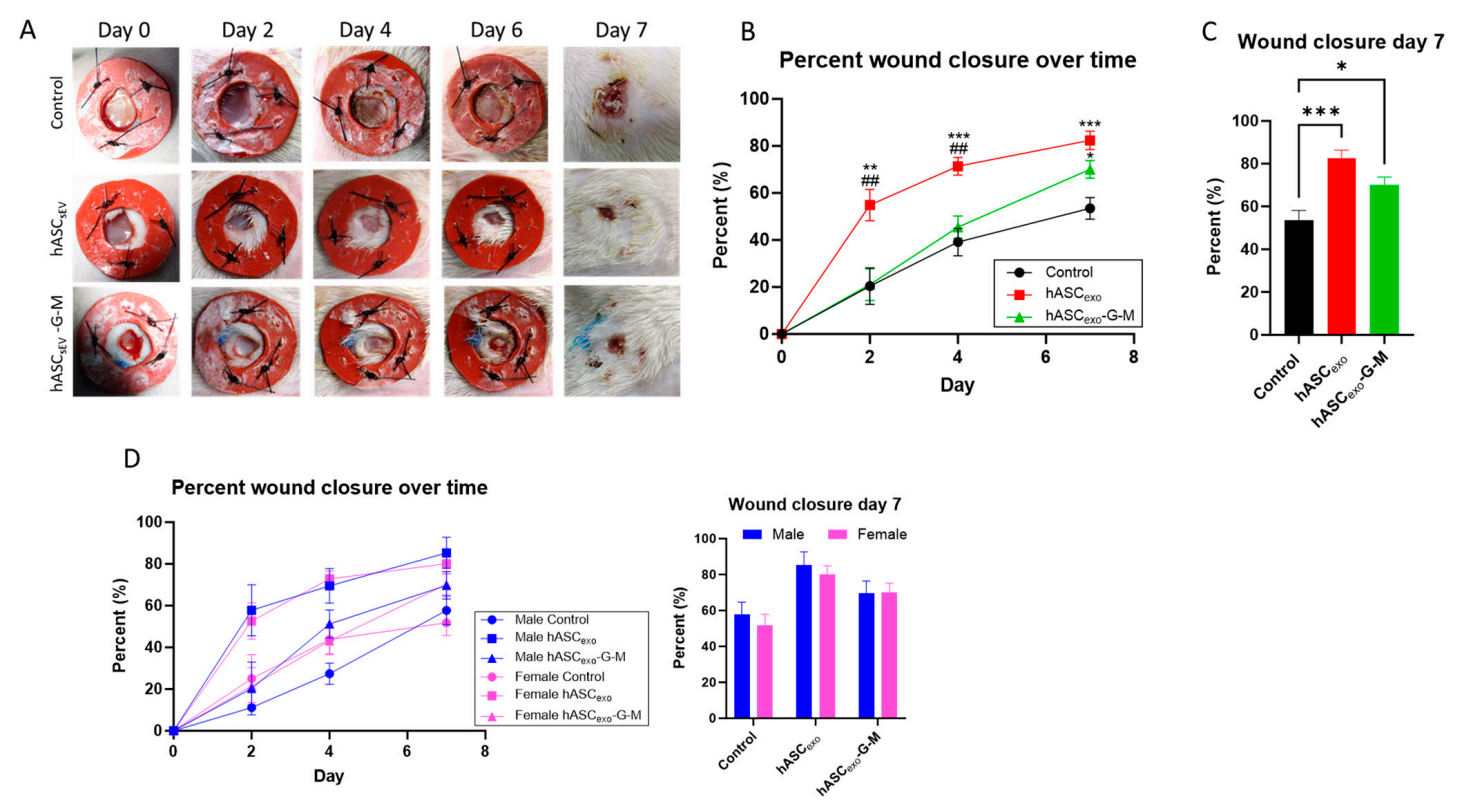
In vivo rat wound healing model. Rats were wounded with a 6 mm biopsy punch on day 0. Silicone rings were affixed to the skin around the wounds to reduce skin contraction over the wound. Wounds were treated with hASC_exo_ or hASC_exo_-G-M as well as photographed and measured with calipers every 2 days to monitor healing. (**A**) Representative photographs of wound healing progression over 7 days. (**B**) Percent wound closure over time in untreated wounds and wounds treated with either hASC_exo_ or hASC_exo_-G-M. #: hASC_exo_ vs. hASC_exo_-G-M.*: hASC_exo_ vs. Control. (**C**) Wound closure percent at day 7 post wounding. (**D**) Percent wound closure over time and percent wound closure at day 7 in male vs. female rats. * *p* < 0.05, ** *p* < 0.01, ^##^ *p* < 0.01, *** *p* < 0.001. Statistical analysis performed using GraphPad Prism SPSS Analysis Software V.10.02 to calculate one-way ANOVA. Analysis of male vs. female was analyzed via two-way ANOVA. Analysis comparing wounds of both males and females *n* = 7, analysis of males vs. females *n* = 3–5.

To evaluate the in vivo efficacy of hASC_exo_ and hASC_exo_-G-M, a rat wound model was used. Six-millimeter wounds were created on the back of each rat and treated topically with PBS vehicle control, 100 µg hASC_exo_, or 100 μg hASC_exo_-G-M at day 0 then every other day for 7 days. Wounds were measured and photographed every 2 days (Figure 4A). The treatment of wounds with hASC_exo_-G-M was significantly slower compared to hASC_exo_ treatment, particularly in the first 4 days of healing. After 2 days, wounds treated with hASC_exo_ were on average approximately 50% closed, while control wounds and those treated with hASC_exo_-G-M had only closed about 20%. On day 4 post-wounding, untreated control wounds had closed approximately 35%, while those treated with hASC_exo_-G-M had closed approximately 40%. Wounds treated with hASC_exo_ were approximately 75% closed by day 4. By the experimental endpoint on day 7, control wounds were 50% closed, while those treated with hASC_exo_ were 80% closed and wounds treated with hASC_exo_-G-M were around 65% closed (Figure 4B,C). Overall, this shows that even with the depletion of *GAS5* and *MALAT1* from hASC_exo_, healing is expedited, however less so than when *GAS5* and *MALAT1* are present in hASC_exo_. Evaluation of differences between male and female responses to hASC_exo_ and hASC_exo_-G-M revealed no significant difference between male and female healing rates over time or by day 7 of healing (Figure 4D).

### 2.5. GAS5 and MALAT1 Knockdown Results in Increased Collagen I and III in the Wound Bed

**Figure 5:**
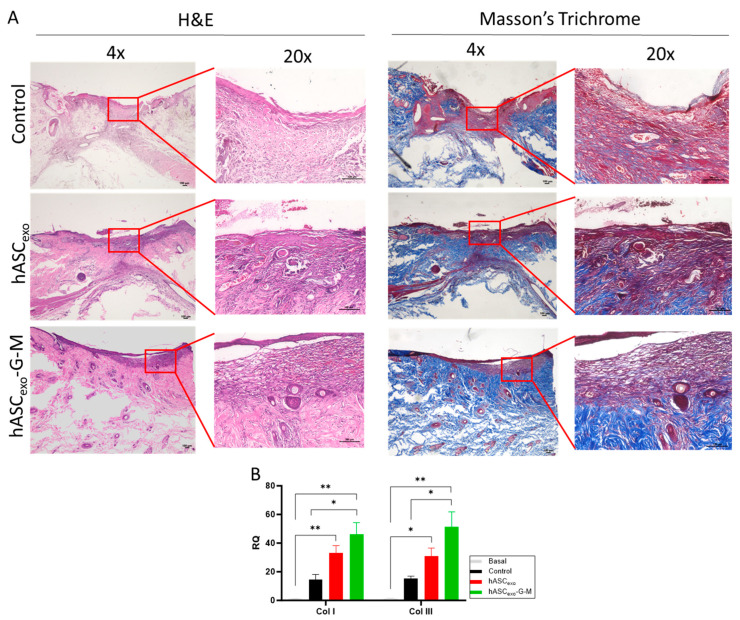
Histology and qPCR analysis of *collagen I* and *III* levels in the wound bed. (**A**) Representative images of H&E and Masson’s Trichrome staining of the wound beds. (**B**) qPCR analysis of collagen I and III levels in the wound bed. Basal samples are unwounded skin samples. * *p* < 0.05, ** *p* < 0.01. Statistical analysis performed using GraphPad Prism SPSS Analysis Software V.10.02 to calculate unpaired one-way ANOVA. RQ = Relative Quantification. *n* = 3. Scale bars = 100 µm.

Collagen in the wound bed was visualized by Masson’s trichrome staining (Figure 5A). Images show more collagen (blue) is present in the wound bed of wounds treated with hASC_exo_ compared to control wounds. Wounds treated with hASC_exo_-G-M show a high content of collagen stain. To verify collagen levels present in each wound, *Col I* and *III* levels were quantified via qPCR. Results show that both *Col I* and *III* levels are significantly increased during the wound healing process. However, the knockdown of *GAS5* and *MALAT1* resulted in significantly higher *Col I* and *III* levels than in control wounds. These levels were also greater than that of wounds treated with hASC_exo_, though not significantly (Figure 5B). These results indicate that the presence of *GAS5* and *MALAT1* in exosomes has some inhibitory effects on excessive collagen production in the wound.

### 2.6. Skin, Epidermis, and Keratinocyte Development Is Suppressed in hASC_exo_-G-M-Treated Wounds While Inflammatory Pathways Are Activated

**Figure 6:**
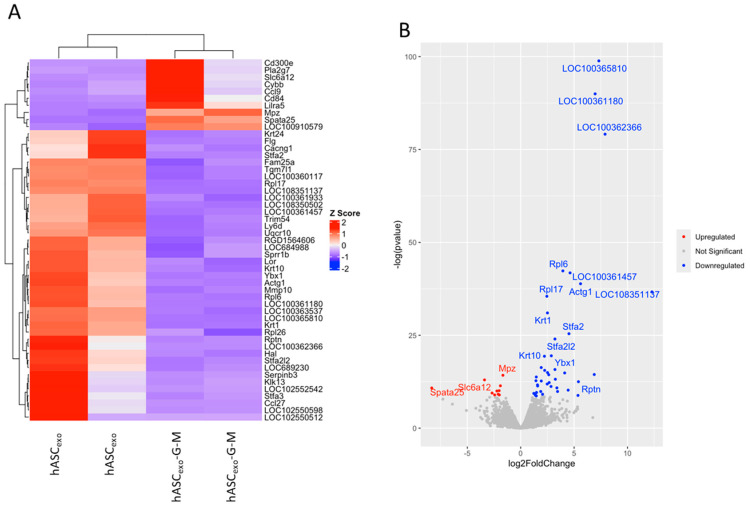

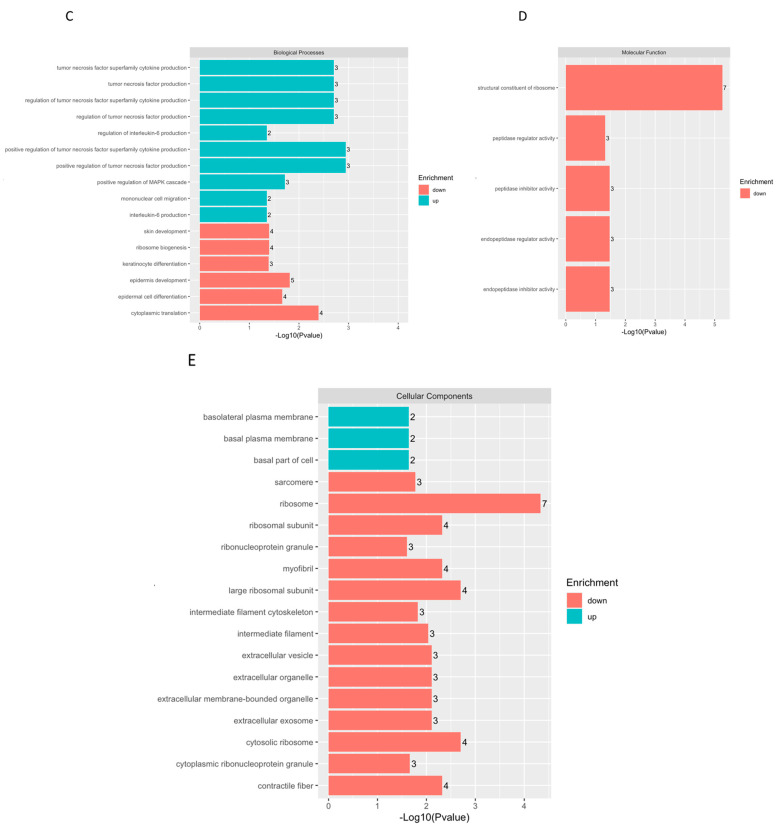

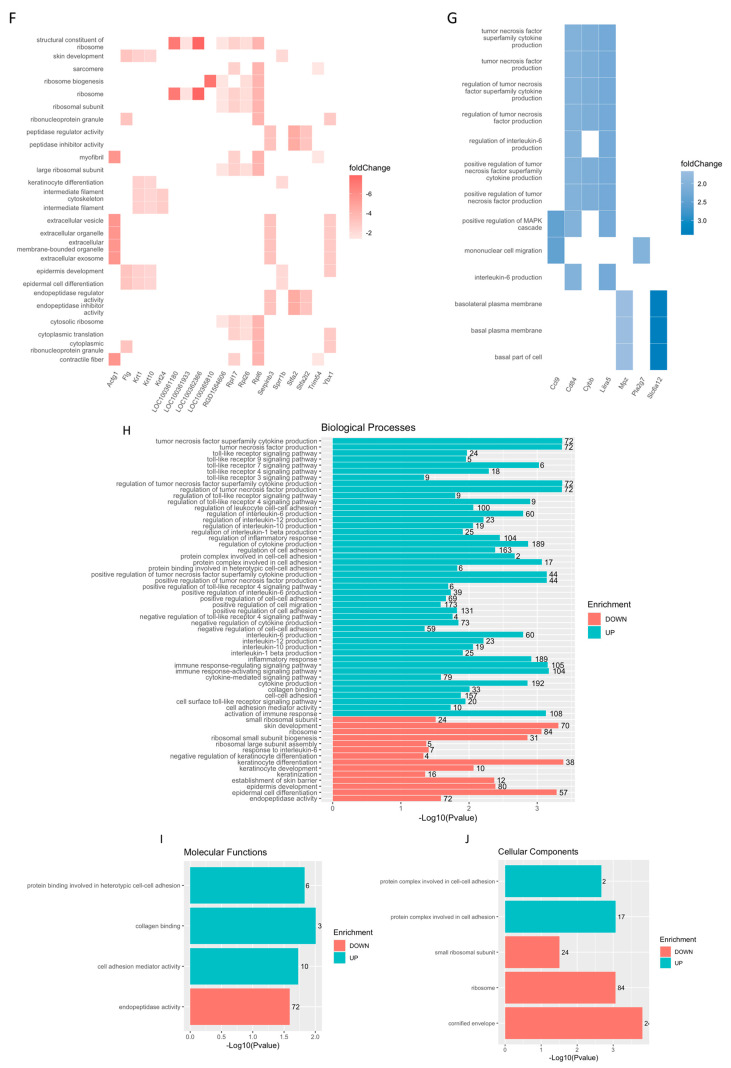

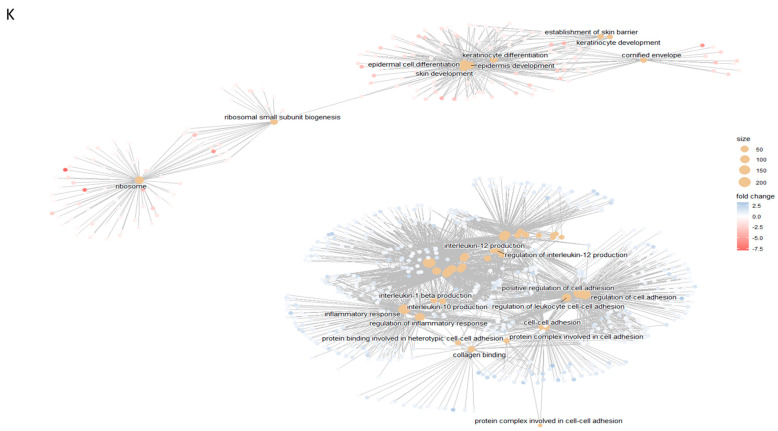
RNAseq results comparing hASC_exo_- and hASC_exo_-G-M-treated wounds. RNAseq analysis completed using RStudio (v 2023.09.0+463) (**A**) Heatmap of significant differentially expressed genes (DEGs). Red = upregulated genes, blue = downregulated genes. DEG analysis completed via R package DESeq2 (v 1.42.1). (**B**) Volcano plot of all genes. Red = upregulated genes, blue = downregulated genes, grey = non-significant genes. Genes with a *p*-value < 0.05 were considered significant and those with a Log2FC greater than 0.5 were considered upregulated while those less than −0.5 were considered downregulated. (**C**) Over-representation analysis (ORA) of biological process (**D**) molecular function, and (**E**) cellular component gene ontology (GO) terms. Blue = pathways upregulated in hASC_exo_-G-M-treated wounds compared to hASC_exo_-treated wounds, red = pathways downregulated in hASC_exo_-G-M-treated wounds compared to hASC_exo_-treated wounds. Numbers at the end of each bar represent the number of significant DEGs present in a pathway. Analysis completed using R package clusterProfiler (v 4.10.1) and figure created using R package ggplot2 (V.3.5.1). (**F**) Heatmap of DEGs present in each ORA GO pathway that is downregulated or (**G**) upregulated in wounds treated with hASC_exo_-G-M compared to those treated with hASC_exo_. (**H**) Gene set enrichment analysis (GSEA) GO analysis of biological process, (**I**) molecular function, and (**J**) cellular component pathways significantly enriched in all DEGs. Blue = pathways upregulated in hASC_exo_-G-M-treated wounds compared to hASC_exo_-treated wounds, red = pathways downregulated in hASC_exo_-G-M-treated wounds compared to hASC_exo_-treated wounds. Numbers at the end of each bar represent the number of significant DEGs present in a pathway. Analysis completed using R package clusterProfiler (v 4.10.1) and figure created using R package ggplot2 (V.3.5.1). (**K**) Netplot of top GSEA GO pathways. Tan circles represent pathways. The size of each tan circle indicates the number of DEGs expressed in the pathway. Each gene is denoted by the smaller blue or red circles. Red = downregulated genes, blue = upregulated genes. Each line from a gene indicates the pathway(s) that the gene is included. Figure generated using R package clusterProfiler (v 4.10.1).

RNAseq was performed on the wounds collected on day 7 to evaluate the differentially expressed genes and pathways in wounds treated with hASC_exo_ and hASC_exo_-G-M. RNA from one male and one female rat from each exosome treatment was analyzed. Fifty-one significant differentially expressed genes (DEGs) were identified with 41 genes downregulated and 10 genes upregulated in hASC_exo_-G-M-treated wounds compared to hASC_exo_-treated wounds (Figure 6A,B).

To identify the functionality of the significant genes enriched in hASC_exo_-G-M- vs. hASC_exo_-treated wounds, over-representation analysis (ORA) in biological process (BP), cellular component (CC), and molecular function (MF) from gene ontology (GO) pathways was performed. Biological process pathways that were upregulated in wounds treated with hASC_exo_-G-M compared to those treated with hASC_exo_ are associated with tumor necrosis factor production, interleukin production, and cell migration, while downregulated pathways are involved with skin or epidermis development, and ribosomes (Figure 6C). All Molecular function pathways were downregulated in hASC_exo_-G-M- vs. hASC_exo_-treated wounds. These pathways were involved with ribosomes and peptidase activity (Figure 6D). Finally, cellular component pathways that are upregulated are involved with the basal plasma membrane, while downregulated pathways are associated with ribosomes, muscle fibers, and extracellular vesicles (Figure 6E). Heatmaps of the DEGs in each GO pathway were created to visualize the genes represented in the samples from each pathway (Figure 6F,G).

To further evaluate pathway enrichment, gene set enrichment analysis (GSEA) was completed on all gene sets for BP, CC, and MF GO terms. Biological process pathways that were upregulated are involved with tumor necrosis factor production, toll-like receptor signaling, interleukin production, immune and inflammatory responses, and cell adhesion. Pathways associated with ribosomes, skin or epidermis development, and keratinocyte development were downregulated (Figure 6H). Enriched molecular function pathways included those associated with cell adhesion and collagen binding (upregulated) and endopeptidase activity (downregulated; Figure 6I). Cellular component pathways that were upregulated also included those associated with cell adhesion, while downregulated pathways are involved with ribosomes and the cornified envelope (Figure 6J). A netplot of the top enriched GSEA Go pathways was generated (Figure 6K). Two major clusters resulted: one that included pathways involved with skin or epidermis development and one that included pathways involved with cell adhesion, interleukin production, and inflammatory response. Genes in the first cluster all have a negative fold change (downregulated in hASC_exo_-G-M-treated vs. hASC_exo_-treated wounds) while those in the second cluster mostly have a positive fold change (upregulated in hASC_exo_-G-M-treated vs. hASC_exo_-treated wounds).

### 2.7. Treatment of Wounds with hASC_exo_ and hASC_exo_-G-M Alters Expression of Molecular Markers for Inflammation, Apoptosis, Angiogenesis, and Collagenase Compared to Untreated Control Wounds

**Figure 7:**
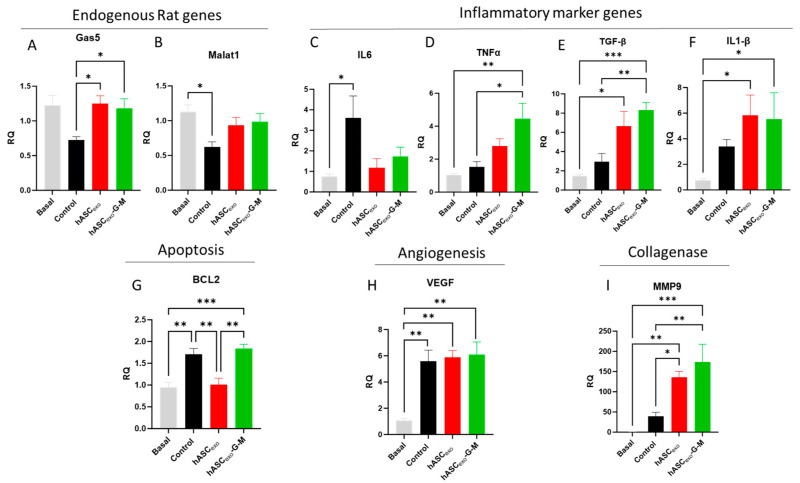
qPCR was performed on combined male and female rat wound samples. qPCR analysis results for endogenous rat genes (**A**) *Gas5* and (**B**) *Malat1*; (**C**) inflammatory markers *IL6*, (**D**) *TNFα*, (**E**) *TGF-β*, and (**F**) *IL1-β*; (**G**) apoptosis marker *BCL2*; (**H**) angiogenesis marker *VEGF*; and (**I**) collagenase *MMP9*. Basal samples are unwounded skin samples. * *p* < 0.05, ** *p* < 0.01, *** *p* < 0.001. Statistical analysis performed using GraphPad Prism SPSS Analysis Software V.10.02 to calculate one-way ANOVA. RQ = Relative Quantification. *n* = 3–5.

Based on the pathways identified by RNAseq, we selected certain genes to verify RNAseq results via real-time qualitative polymerase chain reaction (RT qPCR) on combined male and female wound samples. Unwounded skin samples were used as basal samples. Using rat-specific primers to distinguish endogenous levels of *Gas5* and *Malat1* from *GAS5* and *MALAT1* contained in human exosomes, we found that control wounds had significantly lower levels of rat *Gas5* on day 7 post-wounding compared to either exosome treatment. *Malat1* levels in both exosome treatments were higher than in control wounds. Rat *Gas5* levels increased with exosome treatment to approximately basal levels. *Malat1* levels increased with exosome treatment, though not significantly. There was no significant difference between hASC_exo_ and hASC_exo_-G-M treatments in endogenous rat *Gas5* and *Malat1* levels (Figure 7A,B).

Next, levels of inflammatory markers were tested. *IL6* expression increased significantly in control wounds compared to basal samples. These levels were lowered with exosome treatment; however, there was no significant difference comparing the two exosome treatments (Figure 7C). *TNFα* levels were unchanged on day 7 in control wounds vs. basal conditions. However, *TNFα* levels increased in wounds treated with both exosomes. This increase was significant in wounds treated with hASC_exo_-G-M (Figure 7D). *TGF-β* is known to control functions within the cell such as proliferation and differentiation. We found *TGF-β* increased in control wounds compared to basal skin samples. There was no significant difference between control wounds and those treated with hASC_exo_, however, treatment with hASC_exo_-G-M significantly increased *TGF-β* levels compared to control wounds. Both exosome treatments significantly increased *TGF-β* (Figure 7E). *IL1-β* performs multiple functions within a cell such as inflammatory response, cell proliferation, differentiation, and apoptosis. *IL1-β* levels were increased in wounded skin compared to basal skin, especially in wounds treated with exos. There was no significant difference between hASC_exo_ and hASC_exo_-G-M (Figure 7F). To evaluate apoptosis, we evaluated *BCL2* expression levels. Control wounds had higher *BCL2* levels than basal skin samples. Wounds treated with hASC_exo_ had levels similar to that of basal samples. Wounds treated with hASC_exo_-G-M experienced significantly higher levels of *BCL2* compared to both basal samples and wounds treated with hASC_exo_ (Figure 7G). *VEGF* is involved in multiple areas of wound healing such as angiogenesis, epithelialization, and collagen deposition. *VEGF* was significantly increased in wounded tissue compared to basal tissue. There was no significant difference in *VEGF* levels between control wounds and wounds treated with either exosome. (Figure 7H). *MMP9* is a collagenase that performs functions in keratinocyte migration and angiogenesis. *MMP9* levels were significantly increased in control wounds compared to basal wounds. Levels were highly increased in wounds treated with hASC_exo_-G-M (Figure 7I).

### 2.8. Male and Female Rats Respond Equally to Exosome Treatment

**Figure 8:**
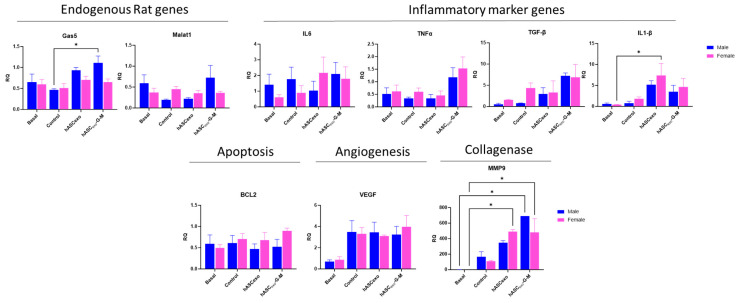
qPCR analysis results comparing male and female for *Gas5, Malat1, IL6, TNFα, TGF-β, IL1-β, BCL2, VEGFa*, and *MMP9*. Basal samples are unwounded skin samples. * *p* < 0.05. Statistical analysis performed using GraphPad Prism SPSS Analysis Software V.10.02 to calculate two-way ANOVA. RQ = Relative Quantification. *n* = 3.

To evaluate for sex differences in exosome treatments of wounds, analysis of qPCR data from Figure 7 was performed to compare the effect of hASC_exo_ and hASC_exo_-G-M between male and female rats. We found no significant difference in the levels of any of the measured genes between males and females (Figure 8). However, results show that wounds on male rats treated with hASC_exo_-G-M had significantly more *Gas5* than untreated control wounds, and significantly more *MMP9* than basal samples. Further, wounds on female rats experienced higher levels of *IL1-β* in hASC_exo_-treated wounds compared to basal samples. Female basal samples also showed significantly lower *MMP9* than in wounds treated with hASC_exo_-G-M.

## 3. Discussion

Our lab and others have previously shown that in wound healing in cell culture and rodent models, treatment with exosomes derived from hASCs significantly improves wound healing [12,18,19,20]. Further, our previous studies have indicated that the lncRNAs metastasis-associated lung adenocarcinoma transcript 1 (*MALAT1*) and growth-arrest specific-5 (*GAS5*) as highly enriched in hASC_exo_. Previously, we have shown that the lncRNAs *anti-NOS2A, DLG2AS, GAS5, HOTAIRM1, lincRNAp21, lincRNA-VLDLR, NEAT1*, and *MALAT1* are enriched in hASC cells as well as in the conditioned media (CM) of hASC cultured cells. Evaluation of the exosomes secreted from hASCs showed that *GAS5, MALAT1*, and *lincRNA-VLDLR* were all enriched in these exosomes. Of these three lncRNAs, *GAS5* and *MALAT1* showed particularly high enrichment in exosomes. Due to this finding, we have previously investigated the effects of *GAS5* and *MALAT1* independently as critical cargos of hASC_exo_ when used as a therapeutic for wound healing. These data have shown that, independently, *MALAT1* and *GAS5* promote wound healing [21,22,23]. Our results presented here demonstrate that by simultaneously knocking down *MALAT1* and *GAS5* in hASC_exo_, several mechanisms within the wound healing process are altered. The in vivo rat wound healing model performed here indicates that the depletion of *GAS5* and *MALAT1* from hASC_exo_ delays wound healing, particularly in the first 4 days of healing, implying that *GAS5* and *MALAT1* are vital for the early mechanisms of wound healing.

In vitro, *MALAT1* promotes wound healing via increasing cell proliferation and migration while inhibiting apoptosis [22,24], while *GAS5* depletes pro-inflammatory markers including *IL6* and *IL1-β* [21]. Additionally, *GAS5* levels are depleted in diabetic patients [25] resulting in an inhibition of glucose uptake and insulin signaling [26]. Both *GAS5* and *MALAT1* have been extensively studied in the field of cancer research. In multiple cancers, *GAS5* is a known tumor suppressor in which high levels are associated with increased apoptosis and decreased cell proliferation [27]. However, *GAS5* appears to function differently in stem cells. Xu et al. (2016) found that overexpression of *GAS5* in human embryonic stem cells promoted self-renewal [28]. *MALAT1* has proven to be important for the promotion of metastasis of various cancers such as colorectal cancer [29] and pancreatic cancer [30], whereas it has been shown to suppress metastasis in breast cancer [31] and glioma [32]. Li et al. (2016) found that in pancreatic cancer, *MALAT1* activates functions associated with metastasis such as autophagy, migration, apoptosis, and cell invasion [30]. Interestingly, these functions are also beneficial to wound healing. Overall, we have demonstrated in this study that *GAS5* and *MALAT1* are both required for wound healing. As previously stated, *GAS5* levels are low in patients with diabetes [25]. Diabetic patients commonly suffer from diabetic foot ulcers that often result in amputation due to delayed healing [33]. One possible reason for these unhealing wounds may be the depletion of *GAS5*. By increasing *GAS5* levels in the wounds of these patients, healing could be improved, resulting in fewer patients needing lower limb amputations. However, further studies are needed to confirm this in human patients. Our previous work has also demonstrated that when using hASC_exo_ intravenously to treat traumatic brain injury (TBI), *MALAT1* in the exosomes reduced the expression of inflammatory markers such as *IL1-β, TNFα*, and *IL10* in the cortex after injury [20]. In a similar study of *MALAT1* in TBI, researchers found that Malat1-deficient mice with TBI experienced a reduction in cellular proliferation at the cortex, density of functional vessels, and cerebral blood flow [34]. Other studies have observed *MALAT1* as protective in other internal injuries such as ischemia-reperfusion injury of the lung after transplant [35] and in hypoxic and ischemic brain injury after strokes [36]. This indicates *MALAT1*’s importance to internal injury repair. More studies of hASC_exo_ delivery of *MALAT1* would elucidate whether this is a viable therapeutic option for promotion of healing in internal injuries.

*Collagens I* and *III* are the major types of collagens present during wound healing. Early in wound healing, *Col III* is the most common in the wound bed, while later in healing, *Col I* is more prominent [37]. Our data show that *Col I* and *Col III* levels are relatively equal in all treatment groups. However, there are significant changes across groups. Non-wounded skin tissue has significantly lower collagen levels than wounded tissue. Further, wounds treated with hASC_exo_-G-M exhibit higher collagen levels than those treated with hASC_exo_. Previous studies have indicated that *GAS5* is a suppressor of collagen deposition and these studies have demonstrated that *GAS5* reduces fibrosis of various organs such as the heart, liver, and kidneys [38,39,40]. High collagen levels can result in increased scarring [41]; therefore, this decrease in endogenous levels of *GAS5* and *MALAT1* in hASC_exo_-G-M-treated wounds may result in a greater incidence of scar tissue. Here, on day 7 the levels of *Col I* and *Col III* are approximately equal in each wound regardless of treatment group. Because *Col I* levels are highest in healed tissue, this indicates that these wounds are at the beginning of the remodeling phase.

RNAseq analysis was completed at day 7 of wound healing to evaluate the differences in molecular markers and pathways that arise when wounds are treated with hASC_exo_ and hASC_exo_-G-M. Over-representation analysis (ORA) and gene set enrichment analysis (GSEA) for gene ontology (GO) revealed that biological process (BP) pathways involved with cytoplasmic translation, epidermis development, epidermal cell differentiation, ribosome biogenesis, skin development, and keratinocyte differentiation were downregulated in wounds treated with hASC_exo_-G-M compared to those treated with hASC_exo_. Specifically, the downregulation of epidermis development, epidermal cell differentiation, skin development, and keratinocyte differentiation reveals that skin regeneration is occurring at a lower rate in hASC_exo_-G-M-treated wounds, reinforcing the idea that *GAS5* and *MALAT1* are critical components of hASC_exo_ when it comes to wound healing. Further, the BP pathway, ribosome biogenesis, several cellular component (CC) pathways involved with ribosomes, and the molecular function (MF) pathway structural constituent of ribosome, are all downregulated in hASC_exo_-G-M-treated wounds. *GAS5* is known to be involved in the biogenesis of ribosomes [42]. This reduction of ribosome biogenesis due to *GAS5* depletion may be one major reason why wound healing is delayed in hASC_exo_-G-M-treated wounds. Additionally, in hASC_exo_-G-M-treated wounds, there is a downregulation of pathways involved with intermediate filaments and the cytoskeleton. *MALAT1* has previously been shown to have effects on cellular cytoskeletons. Cai et al. (2018) demonstrated that a knockdown of *MALAT1* in vitro and in vivo resulted in a reorganization of the cytoskeleton, causing negative effects on processes such as cell motility and migration [43].

To gain insight, proteomic analysis of the exosome cargo was completed to determine differences in the cargo of hASC_exo_ and hASC_exo_-G-M. We found that of the 117 identified proteins, 4 were upregulated while 113 were downregulated in hASC_exo_-G-M compared to hASC_exo_. Of these proteins, 31 were significant—all of which were downregulated. Interestingly, among these proteins were MMP2, TGF-β1, and FN1, which are all important for wound healing. MMP2 is a matrix metalloproteinase, which, in wound healing, works with its inhibitor (TIMP) to break down and build up components of the extracellular matrix (ECM). When there is an imbalance of MMP2 to TIMP, healing can be delayed [44]. Levels of the growth factor TGF-β1 were decreased in hASC_exo_-G-M compared to hASC_exo_. TGF-β performs multiple functions in wound healing such as angiogenesis, inflammation, and fibroblast proliferation, among others and it has been shown that depletion of TGF-β1 is common in chronic wounds [45]. Fibronectin (FN) is another protein that is important for wound healing that we found to be reduced in hASC_exo_-G-M. In particular, FN1 is highly involved in processes such as cellular growth and tissue repair [46]. The reduced levels of proteins such as MMP2, TGF-β1, and FN1 in the cargo of hASC_exo_-G-M likely contribute to slower healing times in wounds treated with these exosomes. The results also suggest that *GAS5* and *MALAT1* influence the packaging of other cargo in the hASC exosomes. This observation will be evaluated in future studies.

It is important to keep in mind that the biochemical experiments that were performed in the presented in vivo experiments were completed at day 7 of wound healing. Our analysis of healing over time in the rats reveals that there is a greater difference in the percent wound closure on days 2 and 4 between wounds treated with hASC_exo_ compared to those treated with hASC_exo_-G-M. However, by day 7, wounds treated with hASC_exo_-G-M have closed almost as much as those treated with hASC_exo_, indicating that *GAS5* and *MALAT1* may be more critical to the earlier stages of wound healing. Further, in this study, we treated wounds on rats with exosomes derived from ASC_exos_ originating from humans. We do not believe this cross-species treatment approach has any effect on the results of the study. A similar pattern of healing to the rat wounds was observed in our in vitro model of wound healing on human dermal fibroblasts (HDF) indicating that using human-derived exosomes to treat rats yields no differences in effects. We currently have not investigated the differences in same-species vs. interspecies treatment; however, future work may be completed to evaluate whether there are any effects. Additionally, the siRNAs for *GAS5* and *MALAT1* were not packaged inside the exosomes as the siRNA-containing media was replaced with fresh medium for 48 h followed by collection of conditioned media for isolation of exosomes. The endogenous rat *Gas5* and *Malat1* were increased in exosome-treated wounds, but there was no significant difference between hASC_exo_- and hASC_exo_-G-M-treated wounds (Figure 7) This suggests that other exosomal cargo affects gene expression.

In conclusion, we have identified the lncRNAs *GAS5* and *MALAT1* as important cargo of exosomes and have demonstrated the therapeutic application of exosomes in wound healing. Knowledge from this study also advances the use of hASC exosomes into clinical use as it defines the role of individual cargo that drives the repair and regeneration of wounds in a cell-free manner.

One limitation of the study is that wound healing was measured only until 7 days post-wounding. At this point, healing is not completed and wounds in all treatment groups are still in the healing process. In the future, healing will be monitored for 28 days, when wounds are expected to be completely healed. Additionally, a daily timepoint study that evaluates molecular markers during early healing would elucidate how *GAS5* and *MALAT1* contained in hASC exosomes affect acute phases of healing. Additionally, in this study, we did not perform any immune response analysis or further investigate the molecular mechanisms at play. We plan to conduct additional studies in which these questions will be addressed in the future.

## 4. Materials and Methods

### 4.1. Isolation of Exosomes from hASCs

Pooled hASCs were purchased from ZenBio and cultured in ZenBio preadipocyte media until 90% confluent. The hASCs were then cultured for 48 h in the serum exosome-free hASC media. After 48 h, the conditioned media (CM) was collected and centrifuged at 1500× *g* for 10 min to remove debris. The CM was concentrated using a 10 kDa molecular weight cut-off filter (MWCO). Isolation of exosomes from CM was completed via size exclusion chromatography (SEC) using the Izon qEV10/35 nm Legacy kit (Izon, Christchurch, New Zealand) according to manufacturer instructions. Samples were collected after flowing through the qEV10 column. Peak diameter and exosome concentration were analyzed using NanoSight (Salisbury, UK, NTA3.1, Build 3.1.46 RRID SCR-014239) nanoparticle tracking analysis.

### 4.2. Knockdown of GAS5 and MALAT1 in hASCs

*GAS5* and *MALAT1* were depleted from hASC_exo_. Each was depleted by simultaneously transfecting 25 nM of their previously validated respective siRNA (GAS5: ThermoFisher/Ambion cat# n272340; MALAT1: Ambion cat# 4455877; verified 3 siRNAs for no off-target effects) into cells with RNAiMax (ThermoFisher cat# 13778075) for 48 h (Waltham, MA, USA). The media was then changed, and hASC_exo_-G-M was incubated in media without siRNA for 48 h prior to collecting CM and isolating the exosomes as described in Section 4.1. By changing the media to remove the siRNAs, they are prevented from being packaged inside the exosomes and affecting the recipient cells.

### 4.3. Transmission Electron Microscopy (TEM)

Transmission electron microscopy (TEM) images were obtained to verify that the knockdown of *GAS5* and *MALAT1* did not alter the morphology of the exosomes. Procedures are as described in our previous publication [47]. Briefly, 4 μL of each exosome preparation was placed on a carbon-filled coated copper mesh grid and incubated at room temperature for 10 min. Excess liquid was removed prior to washing 3 times with 0.2-micron filtered, boiled distilled water to remove PBS. After drying overnight, samples were imaged using a JEOL 1400 Transmission Electron Microscope (JEOL Ltd., Akishima, Japan) at 100k× magnification.

### 4.4. In Vitro Wound Healing Model

An in vitro scratch assay was used to evaluate the effect of hASC_exo_ or hASC_exo_-G-M treatment on wound healing. Human dermal fibroblasts (HDF) (American Type Culture Collection, Manassas, VA, USA) were grown to confluency in DMEM supplemented with 10% FBS within a 12-well culture plate. A scratch was made in each well using a 200 μL pipette tip. Cells within a well were treated with 2 µg hASC_exo_ or hASC_exo_-G-M in triplicate. Images were taken immediately after scratching and again at 24 h using a Keyence BZX-800 microscope (Keyence, Itasca, IL, USA). Images were taken in the same location at both timepoints. The area of the wound was measured via Keyence BZX-800, version 1.1.1.8 analyzer software.

### 4.5. Proteomic Evaluation of Exosome Cargo

Liquid chromatography–mass spectrometry (LC–MS) was utilized to analyze the protein cargo of hASC_exo_ and hASC_exo_-G-M. To prepare the exosome samples for LC–MS, suspension trapping (STRAP) protein digestion was performed. Peptides were characterized using a Thermo Q-exactive-HF-X mass spectrometer (Thermo Fisher, Waltham, MA, USA) coupled to a Thermo Easy nLC 1200 (Thermo Fisher, Waltham, MA, USA). Samples were separated at 300 nL/min via an Acclaim PEPMAP 100 c18 trap column (75 μm, 2 cm, 3 μm, 100 A; Thermo Fisher, Waltham, MA, USA) and a Thermo easy spray c18 column (75 μm, 25 cm, 100 A; Thermo Fisher, Waltham, MA, USA) using a 120-min gradient with an initial starting condition of 2% buffer B (0.1% formic acid in 90% Acetonitrile) and 98% buffer A (0.1% formic acid in water). Buffer B was increased to 28% over 140 min, then up to 40% in an additional 10 min. High buffer B (90%) was run for 15 min afterward. The mass spectrometer was outfitted with a Thermo nanospray easy source (Thermo Fisher, Waltham, MA, USA) with the following parameters: spray voltage: 2.1 V, capillary temperature: 300 dC, funnel RF level = 40. Parameters for data acquisition were as follows: for MS data, the resolution was 60,000 with an AGC target of 3 × 10^6^ and a max IT time of 50 ms; the range was set to 400–1600 *m*/*z*. MS/MS data were acquired with a resolution of 15,000, an AGC of 1 × 10^4^, max IT of 50 ms, and the top 30 peaks were picked with an isolation window of 1.6 *m*/*z* with a dynamic execution of 25 s. Resulting samples were processed using MaxQuant v 2.3.1.0 (MaxQuant, Planegg, Germany). A reviewed human database was downloaded from Uniprot and searched with the following parameters: tryptic enzyme with a max of 2 missed cleavages, a precursor mass tolerance of 10 ppm, and a fragment mass tolerance of 0.02 Da. Modifications included Oxidation, Acetyl, and Carbamidomethyl.

### 4.6. Quantitative Real-Time PCR

RNAzol (TelTest Inc., Friendswood, TX, USA) was used to isolate the total RNA from samples. RNA was then reverse transcribed to cDNA using iScript (BioRad, Hercules, CA, USA, Cat #: 170-8891). 0.5 µL of cDNA with Maxima SYBR Green/Rox qPCR master mix (Applied Biosystems, Waltham, MA, USA, Cat #: A25742) was used to perform qPCR. The primers that were used are listed in Table 1. Real-time PCR was performed in triplicate. Amplification was completed with the ViiA 7 (Applied Biosystems, Waltham, MA, USA). Relative quotient (RQ) was used for analysis.

### 4.7. ProteinSimple Jess Automated Western Blot

For animal wound samples, automated Western Blot was performed using a ProteinSimple Jess system (ProteinSimple, Santa Clara, CA, USA) according to manufacturer instructions. The ProteinSimple 12–230 kDa Separation capillary cartridges were used for sample separation. A 1 mg/mL sample was loaded for each antibody. Antibodies listed in Table 2 were used for Jess Automated Western Blot at a 1:10 dilution. Compass Software, version 6.3 (ProteinSimple, San Jose, CA, USA) was used for automated Western Blot analysis.

### 4.8. Animals

The James A. Haley Veterans’ Hospital and University of South Florida Institutional Animal Care and Use Committee (IACUC) approved all experimental procedures with animals consistent with the governing guidelines and recommendations of AWA and HREA. All experiments complied with the ARRIVE guidelines. All rats were raised and studied in pathogen-free environments housed in plastic, sawdust-covered cages with normal light–dark cycle and free access to chow and water. Twelve-week-old male and female Fisher F344 rats were purchased from Jackson Laboratories.

### 4.9. Wounding and Exosome Treatment of Rats

Six male and six female rats were used in this study. Each rat was wounded in 2 standard locations on their back using a 6 mm biopsy punch. Rodent skin tends to contract over wounds before the wound heals, whereas in humans the wound heals prior to the skin closing. Therefore, to create a model that simulated healing more like that of a human, silicone rings were sutured around each wound to prevent skin constriction. Each wound was treated topically with either PBS vehicle control, 100 µg hASC_exo_, or 100 μg hASC_exo_-G-M at day 0 and then every other day until the experimental endpoint (7 days post-wounding). Wounds were dressed and then re-treated and re-dressed every 2 days. Photographs were taken at day 0 after creating the wound and prior to beginning any treatment and repeated at each dressing change to monitor the healing progress. Further, wounds were measured using calipers as a secondary method of monitoring healing. On day 7, the rats were euthanized, and the wounds and surrounding tissue were collected for biochemical, histological, and RNA sequencing analysis. The quantification of wound size over time was accomplished by calculating the circumference (mm^2^) each day using the formula: Circumference = π · r^2^, where r = the radius of the wound. The percent wound closure was calculated by dividing the wound circumference on a given day by the wound circumference on day 0.

### 4.10. Immunohistocytology

Hematoxylin and Eosin (H&E; Abcam, Cambridge, UK, Cat # ab245880) and Masson’s Trichrome (Abcam, Cambridge, UK, Cat # ab150686) staining were performed according to manufacturer instructions.

### 4.11. RNA Sequencing of Wounds

RNA was isolated from hASC_exo_- and hASC_exo_-G-M-treated wounds on day 7 from 2 male and 2 female rats. Samples from each sex were pooled together for sequencing. The Qubit (Thermo Fisher, Waltham, MA, USA) and Agilent Tape Station (Agilent, Santa Clara, CA, USA) were used for measuring RNA concentration and quality. RIN was insured to be >8.0 for samples. The library was prepared using the TruSeq stranded mRNA Library Prep Kit according to the manufacturer’s instructions (Illumina, San Diego, CA, USA, Cat #: 20040532). The concentration and quality of the resulting DNA library were checked using the Qubit and Agilent Tape Station. Samples were loaded into the Illumina NextSeq 500 with 75 bp pair-end reads with indices. The NextSeq System Suite was utilized for real-time image analysis and base calling. All samples had a minimum of 40 million reads and sequences aligned to >80% to reference genome. Trimmomatic was used to trim reads and then a quality check was performed using FASTQC (v 0.12.1). Reads were mapped using HISAT2 (v 2.2.1) to rat genome GRCm39 (file downloaded from NCBI). Files were converted using SAMtools (v 1.21) and Feature-Counts (v 2.20.0) were used to determine reads. RStudio (v 2023.09.0+463) was used for analysis of differentially expressed genes (DEGs) with R package DESeq2 (v 1.42.1) and over-representation analysis (ORA) and gene set enrichment analysis (GSEA) gene ontology (GO) pathways were investigated using R package clusterProfiler (v 4.10.1).

### 4.12. Statistical Analysis

Experiments were repeated three times for biological replicates. Experimental samples were run in triplicate. Statistical analysis was performed as unpaired Student’s *t*-test, one-way ANOVA, or two-way ANOVA using GraphPad Prism version 10.0.0 for Windows (GraphPad Software, Boston, MA, USA). * *p* < 0.05, ** *p* < 0.01 and *** *p* < 0.001 were used as significant measures.

## 5. Patents

US Patent No. 11,844,779B2 Adipose-derived stem cell exosomes and uses thereof (25 June 2024) awarded to N.A.P. No financial conflict and the patent is not yet licensed.

## Figures and Tables

**Table 1:** qPCR primers used in in vitro and in vivo experiments.

Primer	Sense	AntiSense
Rat *Malat1*	GGTTACCAGCCCAAACCTCA	GCATCAAGGTGAGGGGTGAA
Rat *Gas5*	CTGTGATGGGACATCTGGTGG	TCCCATTTTCTGGCTTCCCAT
Rat *IL1-β*	CACCTCTCAAGCAGAGCACAG	GGGTTCCATGGTGAAGTCAAC
Rat *MMP9*	AGGCGCCGTGGTCCCCACTTACTT	GCAGGGTTTGCCGTCTCCGTTGCC
Rat *TGF-β*	GCAACAACGCAATCTSTGAC	CCTGTATTCCGTCTCCTT
Rat *BCL2*	ATCGCTCTGTGTGGATGACTGAGTAC	AGAGACAGCCAGGAGAAATCAAAC
Rat *VEGF*	CCAGGACTACCCCGATGAGATAG	CTGGCTTTGGTGAGGTTTGATC
Rat *IL6*	TCCTACCCCAACTTCCAATGCTC	TTGGATGGTCTTGGTCCTTAGCC
Rat *TNFα*	AGCACAGAAAGCATGATCCGAG	CCTGGTATGAAGTGGCAAATCG
Rat *Col1*	AGGGAACAACTGATGGTGCTACTG	GGACTGCTGTGCCAAAATAAGAGA
Rat *Col3*	AGGGAACAACTGATGCTGCTACTG	GGACTGCTGTGCCAAAATAAGAGA
Rat *GAPDH*	GGCAAGTTCAATGGCACAGT	TGGTGAAGACGCCAGTAGACTC
Human *GAS5*	CTTCTGGGCTCAAGTGATCCT	TGTGCCATGAGACTCCATCAG
Human *MALAT1*	CTTCCCTAGGGGATTTCAGG	GCCCACAGGAACAAGTCCTA
Human *GAPDH*	GATCATCAGCAATGCCTCCT	TGTGGTCATGAGTCCTTCCA

**Table 2:** Automated Western Blot antibodies.

Antibody	Source	Cat #
Anti-TSG101	Abcam (Cambridge, United Kingdom)	ab125011
Anti-Hu CD63	Millipore Sigma (Burlington, MA, USA)	CBL553
Alix	Cell Signaling Technology (Danvers, MA, USA)	92880
Secondary HRP for rabbit	BioRad (Hercules, CA, USA)	1706515
Secondary HRP for mouse	Invitrogen (Waltham, MA, USA)	62-6520

## Data Availability

The raw data of the RNAseq are uploaded as Supplementary Materials.

## Supplementary Materials

The following supporting information can be downloaded at: https://www.mdpi.com/article/10.3390/ijms26083479/s1.

## Abbreviations

The following abbreviations are used in this manuscript:

EV	Extracellular vesicle
hASC	Human adipose stem cell
exo	exosome
GO	Gene ontology
GSEA	Gene set enrichment analysis
ORA	Over-representation analysis
BP	Biological process
MF	Molecular function
CC	Cellular component
DEG	Differentially expressed gene
TEM	Transmission electron microscopy

## References

[1] Wilkinson H.N., Hardman M.J. (2020). Wound healing: Cellular mechanisms and pathological outcomes. Open Biol..

[2] Raziyeva K., Kim Y., Zharkinbekov Z., Kassymbek K., Jimi S., Saparov A. (2021). Immunology of Acute and Chronic Wound Healing. Biomolecules.

[3] Falanga V., Isseroff R.R., Soulika A.M., Romanelli M., Margolis D., Kapp S., Granick M., Harding K. (2022). Chronic wounds. Nat. Rev. Dis. Primers.

[4] Gallagher K.A., Liu Z.J., Xiao M., Chen H., Goldstein L.J., Buerk D.G., Nedeau A., Thom S.R., Velazquez O.C. (2007). Diabetic impairments in NO-mediated endothelial progenitor cell mobilization and homing are reversed by hyperoxia and SDF-1 alpha. J. Clin. Investig..

[5] Mandrika I., Kumar S., Zandersone B., Eranezhath S.S., Petrovska R., Liduma I., Jezupovs A., Pirags V., Tracevska T. (2021). Antibacterial and Anti-Inflammatory Potential of Polyherbal Formulation Used in Chronic Wound Healing. Evid. Based Complement. Alternat Med..

[6] Zhao B., Liu J.Q., Zheng Z., Zhang J., Wang S.Y., Han S.C., Zhou Q., Guan H., Li C., Su L.L. (2016). Human amniotic epithelial stem cells promote wound healing by facilitating migration and proliferation of keratinocytes via ERK, JNK and AKT signaling pathways. Cell Tissue Res..

[7] Tkach M., Thery C. (2016). Communication by Extracellular Vesicles: Where We Are and Where We Need to Go. Cell.

[8] Buzas E.I. (2023). The roles of extracellular vesicles in the immune system. Nat. Rev. Immunol..

[9] Cheng L., Hill A.F. (2022). Therapeutically harnessing extracellular vesicles. Nat. Rev. Drug Discov..

[10] Nederveen J.P., Warnier G., Di Carlo A., Nilsson M.I., Tarnopolsky M.A. (2020). Extracellular Vesicles and Exosomes: Insights From Exercise Science. Front. Physiol..

[11] Welsh J.A., Goberdhan D.C.I., O’Driscoll L., Buzas E.I., Blenkiron C., Bussolati B., Cai H., Di Vizio D., Driedonks T.A.P., Erdbrugger U. (2024). Minimal information for studies of extracellular vesicles (MISEV2023): From basic to advanced approaches. J. Extracell. Vesicles.

[12] Liu Y.J., Wang C. (2023). A review of the regulatory mechanisms of extracellular vesicles-mediated intercellular communication. Cell Commun. Signal.

[13] An Y., Lin S., Tan X., Zhu S., Nie F., Zhen Y., Gu L., Zhang C., Wang B., Wei W. (2021). Exosomes from adipose-derived stem cells and application to skin wound healing. Cell Prolif..

[14] Zhou C., Zhang B., Yang Y., Jiang Q., Li T., Gong J., Tang H., Zhang Q. (2023). Stem cell-derived exosomes: Emerging therapeutic opportunities for wound healing. Stem Cell Res. Ther..

[15] Lee J.H., Won Y.J., Kim H., Choi M., Lee E., Ryoou B., Lee S.G., Cho B.S. (2023). Adipose Tissue-Derived Mesenchymal Stem Cell-Derived Exosomes Promote Wound Healing and Tissue Regeneration. Int. J. Mol. Sci..

[16] Bonafede R., Brandi J., Manfredi M., Scambi I., Schiaffino L., Merigo F., Turano E., Bonetti B., Marengo E., Cecconi D. (2019). The Anti-Apoptotic Effect of ASC-Exosomes in an In Vitro ALS Model and Their Proteomic Analysis. Cells.

[17] Zhu D., Wang Y., Thomas M., McLaughlin K., Oguljahan B., Henderson J., Yang Q., Chen Y.E., Liu D. (2022). Exosomes from adipose-derived stem cells alleviate myocardial infarction via microRNA-31/FIH1/HIF-1alpha pathway. J. Mol. Cell Cardiol..

[18] Tevlin R., des Jardins-Park H., Huber J., DiIorio S.E., Longaker M.T., Wan D.C. (2022). Musculoskeletal tissue engineering: Adipose derived stromal cell implementation for the treatment of osteoarthritis. Biomaterials.

[19] Patel R.S., Carter G., El Bassit G., Patel A.A., Cooper D.R., Murr M., Patel N.A. (2016). Adipose-derived stem cells from lean and obese humans show depot specific differences in their stem cell markers, exosome contents and senescence: Role of protein kinase C delta (PKCdelta) in adipose stem cell niche. Stem Cell Investig..

[20] Patel N.A., Moss L.D., Lee J.Y., Tajiri N., Acosta S., Hudson C., Parag S., Cooper D.R., Borlongan C.V., Bickford P.C. (2018). Long noncoding RNA MALAT1 in exosomes drives regenerative function and modulates inflammation-linked networks following traumatic brain injury. J. Neuroinflamm..

[21] Patel R.S., Impreso S., Lui A., Vidyarthi G., Albear P., Patel N.A. (2022). Long Noncoding RNA GAS5 Contained in Exosomes Derived from Human Adipose Stem Cells Promotes Repair and Modulates Inflammation in a Chronic Dermal Wound Healing Model. Biology.

[22] Cooper D.R., Wang C., Patel R., Trujillo A., Patel N.A., Prather J., Gould L.J., Wu M.H. (2018). Human Adipose-Derived Stem Cell Conditioned Media and Exosomes Containing MALAT1 Promote Human Dermal Fibroblast Migration and Ischemic Wound Healing. Adv. Wound Care.

[23] El Bassit G., Patel R.S., Carter G., Shibu V., Patel A.A., Song S., Murr M., Cooper D.R., Bickford P.C., Patel N.A. (2017). MALAT1 in Human Adipose Stem Cells Modulates Survival and Alternative Splicing of PKCdeltaII in HT22 Cells. Endocrinology.

[24] Kuang L., Zhang C., Li B., Deng H., Chen R., Li G. (2023). Human Keratinocyte-Derived Exosomal MALAT1 Promotes Diabetic Wound Healing by Upregulating MFGE8 via microRNA-1914-3p. Int. J. Nanomed..

[25] Carter G., Miladinovic B., Patel A.A., Deland L., Mastorides S., Patel N.A. (2015). Circulating long noncoding RNA GAS5 levels are correlated to prevalence of type 2 diabetes mellitus. BBA Clin..

[26] Shi Y., Parag S., Patel R., Lui A., Murr M., Cai J., Patel N.A. (2019). Stabilization of lncRNA GAS5 by a Small Molecule and Its Implications in Diabetic Adipocytes. Cell Chem. Biol..

[27] Yang X., Xie Z., Lei X., Gan R. (2020). Long non-coding RNA GAS5 in human cancer. Oncol. Lett..

[28] Xu C., Zhang Y., Wang Q., Xu Z., Jiang J., Gao Y., Gao M., Kang J., Wu M., Xiong J. (2016). Long non-coding RNA GAS5 controls human embryonic stem cell self-renewal by maintaining NODAL signalling. Nat. Commun..

[29] Ji Q., Zhang L., Liu X., Zhou L., Wang W., Han Z., Sui H., Tang Y., Wang Y., Liu N. (2014). Long non-coding RNA MALAT1 promotes tumour growth and metastasis in colorectal cancer through binding to SFPQ and releasing oncogene PTBP2 from SFPQ/PTBP2 complex. Br. J. Cancer.

[30] Li L., Chen H., Gao Y., Wang Y.W., Zhang G.Q., Pan S.H., Ji L., Kong R., Wang G., Jia Y.H. (2016). Long Noncoding RNA MALAT1 Promotes Aggressive Pancreatic Cancer Proliferation and Metastasis via the Stimulation of Autophagy. Mol. Cancer Ther..

[31] Latorre E., Carelli S., Raimondi I., D’Agostino V., Castiglioni I., Zucal C., Moro G., Luciani A., Ghilardi G., Monti E. (2016). The Ribonucleic Complex HuR-MALAT1 Represses CD133 Expression and Suppresses Epithelial-Mesenchymal Transition in Breast Cancer. Cancer Res..

[32] Han Y., Wu Z., Wu T., Huang Y., Cheng Z., Li X., Sun T., Xie X., Zhou Y., Du Z. (2016). Tumor-suppressive function of long noncoding RNA MALAT1 in glioma cells by downregulation of MMP2 and inactivation of ERK/MAPK signaling. Cell Death Dis..

[33] Chang M., Nguyen T.T. (2021). Strategy for Treatment of Infected Diabetic Foot Ulcers. Acc. Chem. Res..

[34] Wu N., Cheng C.J., Zhong J.J., He J.C., Zhang Z.S., Wang Z.G., Sun X.C., Liu H. (2022). Essential role of MALAT1 in reducing traumatic brain injury. Neural Regen. Res..

[35] Wei L., Li J., Han Z., Chen Z., Zhang Q. (2019). Silencing of lncRNA MALAT1 Prevents Inflammatory Injury after Lung Transplant Ischemia-Reperfusion by Downregulation of IL-8 via p300. Mol. Ther. Nucleic Acids.

[36] Wang L., Li S., Stone S.S., Liu N., Gong K., Ren C., Sun K., Zhang C., Shao G. (2022). The Role of the lncRNA MALAT1 in Neuroprotection against Hypoxic/Ischemic Injury. Biomolecules.

[37] Mathew-Steiner S.S., Roy S., Sen C.K. (2021). Collagen in Wound Healing. Bioengineering.

[38] Liu H.L., Chen C.H., Sun Y.J. (2019). Overexpression of lncRNA GAS5 attenuates cardiac fibrosis through regulating PTEN/MMP-2 signal pathway in mice. Eur. Rev. Med. Pharmacol. Sci..

[39] Yu F., Zheng J., Mao Y., Dong P., Lu Z., Li G., Guo C., Liu Z., Fan X. (2015). Long Non-coding RNA Growth Arrest-specific Transcript 5 (GAS5) Inhibits Liver Fibrogenesis through a Mechanism of Competing Endogenous RNA. J. Biol. Chem..

[40] Yu Y., Jiang H., Niu Y., Huang J., Zhang X., Liu X., Zhang Y., Liu S., Fu H., Yu C. (2020). Long noncoding RNA-GAS5 retards renal fibrosis through repressing miR-21 activity. Exp. Mol. Pathol..

[41] Profyris C., Tziotzios C., Do Vale I. (2012). Cutaneous scarring: Pathophysiology, molecular mechanisms, and scar reduction therapeutics. J. Am. Acad. Dermatol..

[42] Frank F., Kavousi N., Bountali A., Dammer E.B., Mourtada-Maarabouni M., Ortlund E.A. (2020). The lncRNA Growth Arrest Specific 5 Regulates Cell Survival via Distinct Structural Modules with Independent Functions. Cell Rep..

[43] Cai X., Liu Y., Yang W., Xia Y., Yang C., Yang S., Liu X. (2016). Long noncoding RNA MALAT1 as a potential therapeutic target in osteosarcoma. J. Orthop. Res..

[44] Karim R.B., Brito B.L., Dutrieux R.P., Lassance F.P., Hage J.J. (2006). MMP-2 Assessment as an Indicator of Wound Healing: A Feasibility Study. Adv. Skin. Wound Care.

[45] Penn J.W., Grobbelaar A.O., Rolfe K.J. (2012). The role of the TGF-β family in wound healing, burns and scarring: A review. Int. J. Burn. Trauma..

[46] GenÇ S., Yenİ Y., ÇİÇEk B., Hacımüftüoğlu A. (2022). Wound Healing of Quinic Acid in Human Dermal Fibroblasts by Regulating Expression of FN1 and COL1A1 Gene. Türk Doğa Fen Dergisi.

[47] Jones B., Patel R., Wang B., Evans-Nguyen T., Patel N.A. (2025). Lyophilized Small Extracellular Vesicles (sEVs) Derived from Human Adipose Stem Cells Maintain Efficacy to Promote Healing in Neuronal Injuries. Biomedicines.

