# Supplementary material for: lncRNAs GAS5 and MALAT1 Contained in Human Adipose Stem Cell (hASC)-Derived Exosomes Drive the Cell-Free Repair and Regeneration of Wounds In Vivo
Source: Int J Mol Sci. 2025 Apr 8;26(8):3479. doi: 10.3390/ijms26083479 (PMC12027045; doi:10.3390/ijms26083479)
Supplement: Supplementary file 1 [file ijms-26-03479-s001.zip › ijms-3525017-supplementary.pdf]

gene	hASCexo_1	hASCexo_2	hASCexo-G-M_1	hASCexo-G-M_2
A3galt2	1684	1397	1095	2500
A4galt	5	0	8	4
AA926063	44	50	62	193
Aaas	675	936	739	478
Aacs	1555	1086	713	1151
Aadac	17	3	0	17
Aadacl3	120	74	27	51
Aadacl4	1403	475	483	1236
Aaed1	664	738	855	998
Aagab	1365	1901	1553	1288
Aak1	1100	1517	1426	1492
Aamdc	332	269	169	375
Aamp	2944	3999	3026	2543
Aar2	1099	903	915	770
Aard	0	7	15	36
Aars	3052	4027	2956	3147
Aars2	164	160	95	129
Aarsd1	889	1185	763	904
Aasdh	130	93	179	126
Aasdhppt	1782	1202	1253	1726
Aass	3	48	24	24
Aatf	656	701	752	735
Aatk	139	165	126	63
Abca1	2388	1575	2941	4125
Abca12	659	215	397	142
Abca14	12	0	5	4
Abca17	33	97	65	60
Abca2	411	670	326	388
Abca3	1440	1105	1204	1660
Abca4	42	227	81	43
Abca5	120	83	134	79
Abca7	177	234	299	302
Abca8	79	161	84	129
Abca8a	654	448	481	398
Abcb10	360	390	333	420
Abcb1a	295	336	227	596
Abcb6	1001	1512	1044	1229
Abcb7	162	135	188	172
Abcb8	570	886	415	583
Abcb9	131	167	164	113
Abcc1	33	20	28	46
Abcc10	63	67	89	54
Abcc3	169	171	182	167
Abcc4	607	533	525	671
Abcc5	485	578	698	819
Abcc8	64	39	29	22

Abcc9	1185	1036	1071	1215
Abcd1	395	749	575	967
Abcd2	127	106	123	31
Abcd3	3173	1701	1772	1799
Abcd4	178	231	96	187
Abce1	1937	2249	1778	2201
Abcf1	3297	3553	3397	3335
Abcf2	777	902	764	710
Abcf3	1978	1681	2027	1615
Abcg1	1612	1855	2231	2500
Abcg2	96	24	143	74
Abcg3	85	159	161	103
Abcg3l1	41	106	134	213
Abcg3l3	128	219	169	150
Abcg4	129	117	101	150
Abcg5	12	4	5	0
Abhd1	206	340	254	272
Abhd11	478	401	307	262
Abhd12	1405	1664	1851	2192
Abhd12b	350	177	472	45
Abhd13	478	594	569	772
Abhd14a	381	297	298	274
Abhd14b	2042	743	989	808
Abhd15	70	140	78	101
Abhd16a	685	926	626	661
Abhd17a	855	1631	712	889
Abhd17b	562	458	618	641
Abhd17c	1128	542	919	670
Abhd18	123	152	178	93
Abhd2	2420	2741	2297	4944
Abhd3	34	13	23	0
Abhd4	4237	3835	4396	4718
Abhd5	2774	1685	1724	2330
Abhd6	764	691	737	844
Abhd8	205	359	140	145
Abi1	199	347	394	741
Abi2	248	377	399	357
Abi3	143	209	74	209
Abi3bp	1338	1787	1295	1049
Abl2	36	146	142	160
Ablim1	1725	2377	2034	1606
Ablim2	616	759	355	330
Ablim3	538	1866	597	485
Abo	22	53	41	48
Abr	116	577	407	442
Abra	2921	2638	1146	3738
Abracl	3538	3350	3916	4925

Abt1	1027	684	730	1002
Abtb1	477	421	336	495
Abtb2	280	403	260	94
Acaa1	1154	1071	1001	912
Acaa1b	1	0	2	14
Acaa2	5405	3518	4792	3922
Acaca	1829	1340	1508	1503
Acacb	1182	893	632	808
Acad10	499	332	412	338
Acad11	828	416	602	842
Acad8	1277	709	840	1255
Acad9	3579	1709	2091	2022
Acadl	8800	6815	6990	5946
Acadm	10049	5021	5963	7464
Acads	385	689	317	339
Acadsb	20	6	5	10
Acadvl	5100	4112	4006	4043
Acan	114	257	193	196
Acap1	277	220	199	341
Acap2	461	731	683	890
Acap3	314	534	316	592
Acat1	5302	4389	4809	4509
Acat2	626	573	482	505
Acbd3	966	888	1157	1075
Acbd4	309	312	190	221
Acbd5	317	196	283	385
Acbd6	1160	1082	880	921
Accs	110	144	77	111
Accsl	19	13	8	0
Acd	734	815	714	889
Ace	70	479	317	289
Ace2	655	145	512	248
Acer1	863	619	466	560
Acer2	677	590	623	597
Acer3	1789	1051	1368	1527
Acin1	2088	3006	2594	2119
Ackr1	275	421	304	216
Ackr2	478	568	346	105
Ackr3	1161	2941	1258	722
Ackr4	449	493	253	201
Acly	9606	4495	5847	6420
Aco1	1937	2080	1632	2446
Aco2	8723	8693	6017	9588
Acot1	45	10	17	16
Acot13	1042	888	581	844
Acot2	586	332	472	504
Acot3	200	181	155	348

Acot4	11	8	9	15
Acot5	72	138	32	16
Acot7	786	1262	1119	1036
Acot8	204	360	177	211
Acot9	1848	1978	1428	2257
Acox1	2639	2221	2389	2422
Acox3	1192	1274	1162	1236
Acoxl	5	10	3	0
Acp1	51	106	60	35
Acp2	429	976	835	1294
Acp5	1986	2292	1741	4559
Acp6	2052	1063	2030	1091
Acpp	2640	975	1942	898
Acpt	22	18	0	9
Acrbp	150	73	220	140
Acsbg1	5391	2141	2291	2875
Acsf2	1147	641	802	991
Acsf3	255	349	250	347
Acsl1	1669	810	1214	1331
Acsl3	877	995	1204	694
Acsl4	1081	939	1253	1461
Acsl5	7954	5767	5558	7833
Acsl6	204	201	108	155
Acsm1	1133	334	319	1230
Acsm3	370	207	155	121
Acsm4	8	0	62	0
Acss1	396	232	340	466
Acss2	1548	1316	1509	1797
Acss3	159	66	93	188
Acta1	154426	111708	39203	102375
Acta2	3355	9740	7332	8268
Actb	16353	39776	28497	38503
Actg1	3796	2082	21	100
Actg2	741	1351	1022	835
Actl10	15	44	6	27
Actl6a	763	1066	1037	1185
Actn1	2316	2852	3321	2199
Actn2	23858	31002	17087	20096
Actn3	26631	10882	1759	20839
Actn4	1879	7097	4110	4185
Actr10	3146	3504	2798	3762
Actr1b	3856	4676	3813	3801
Actr2	7722	7196	8516	10551
Actr3	4620	6255	5913	10512
Actr3b	21	80	39	12
Actr5	296	285	307	209
Actr6	260	353	337	506

Actr8	754	831	617	921
Acvr1	964	712	996	821
Acvr1b	543	334	436	329
Acvr2a	886	702	804	522
Acvr2b	210	185	176	209
Acvrl1	1035	2298	2028	1978
Acy1	856	892	936	712
Acy3	274	214	457	327
Acyp1	309	494	364	405
Acyp2	495	416	229	488
Ada	439	969	794	1028
Adal	186	186	227	172
Adam10	1246	875	1159	1126
Adam11	53	11	2	0
Adam12	354	1077	917	996
Adam15	1719	3703	2507	3135
Adam17	2571	1945	2420	2543
Adam19	1739	2666	3403	4097
Adam1a	55	60	65	30
Adam22	65	205	119	133
Adam23	269	553	337	542
Adam32	46	11	6	0
Adam33	419	588	272	262
Adam4	34	13	14	34
Adam8	2465	3498	2526	3899
Adam9	938	1617	1661	2018
Adamts1	1042	997	1295	707
Adamts10	536	577	674	700
Adamts12	1570	1361	1730	3409
Adamts13	538	453	262	325
Adamts14	250	1148	579	1040
Adamts15	1598	2786	2377	1985
Adamts16	29	0	86	217
Adamts17	160	305	215	1806
Adamts18	16	23	15	33
Adamts19	30	70	59	54
Adamts2	2280	3754	2552	4168
Adamts20	186	329	129	109
Adamts3	120	47	93	34
Adamts4	486	595	466	750
Adamts5	19	222	45	13
Adamts6	84	230	141	232
Adamts7	86	583	203	215
Adamts8	128	114	104	72
Adamtsl1	268	439	322	548
Adamtsl2	38	97	26	38
Adamtsl3	16	33	36	12

Adamtsl4	1660	1975	1228	1158
Adamtsl5	520	490	185	447
Adap1	78	62	99	161
Adap2	48	202	126	111
Adar	948	1140	984	1024
Adarb1	726	843	573	568
Adat1	74	180	83	141
Adat2	81	54	89	98
Adat3	933	1216	814	1105
Adck1	255	171	217	321
Adck2	676	517	525	541
Adck3	3510	4004	2197	3576
Adck4	434	564	402	577
Adck5	215	326	153	144
Adcy1	968	906	764	472
Adcy10	38	105	48	53
Adcy2	810	872	894	749
Adcy3	108	447	211	311
Adcy4	267	572	406	708
Adcy5	113	167	144	132
Adcy6	1016	1101	1341	1043
Adcy7	227	398	326	261
Adcy9	207	290	251	225
Adcyap1r1	137	154	185	63
Add1	4253	5290	4899	5226
Add2	24	38	54	0
Add3	460	993	909	801
Adgb	43	43	18	3
Adgra1	40	0	98	23
Adgra2	468	401	188	196
Adgra3	2608	1162	3789	1031
Adgrb2	39	42	54	62
Adgrd1	360	797	737	721
Adgre1	515	624	468	1098
Adgre4	18	13	45	59
Adgre5	653	1804	617	2451
Adgrf2	118	34	42	14
Adgrf4	1470	522	946	328
Adgrf5	1188	2349	2604	2024
Adgrg1	3349	3377	3405	2276
Adgrg2	32	111	60	63
Adgrg5	46	54	72	48
Adgrg6	268	277	271	435
Adgrl1	1042	737	981	980
Adgrl2	1670	1019	1709	1130
Adgrl3	594	139	564	234
Adgrl4	939	1580	1324	2000

Adgrv1	95	84	81	127
Adh1	135	119	161	4
Adh4	2566	1974	2416	2267
Adh6a	40	8	3	6
Adh7	118	94	15	134
Adhfe1	81	22	62	74
Adi1	901	730	927	700
Adipoq	1493	293	1590	60
Adipor1	5219	4852	5064	6091
Adipor2	4592	3865	4345	4490
Adk	1300	1185	1359	1328
Adm	182	275	227	237
Adm2	30	28	26	7
Adnp	2245	1725	2139	2285
Adnp2	540	338	468	485
Ado	27	18	24	6
Adora1	63	267	89	39
Adora2a	129	129	147	283
Adora2b	111	125	113	248
Adpgk	789	1050	746	1389
Adprh	1534	1538	1422	1714
Adprhl1	686	949	632	475
Adprhl2	1083	1144	1104	1036
Adprm	570	490	536	930
Adra1a	40	38	33	0
Adra1b	0	58	45	21
Adra1d	122	111	155	329
Adra2a	571	833	1059	1955
Adra2b	15	59	18	29
Adrb2	189	125	215	296
Adrm1	2005	2791	1428	1482
Adsl	2892	2900	1648	2008
Adss	2007	1307	1595	2713
Adssl1	5159	3004	1727	3818
Adtrp	1160	686	1225	278
Aebp1	6664	11259	9829	17810
Aebp2	930	1112	1160	1024
Aen	689	692	563	716
Aes	5661	6340	4824	5313
Afap1	9	6	42	19
Afap1l1	785	857	602	571
Afap1l2	1045	674	816	339
Afdn	4634	1423	3181	1209
Aff1	2353	2091	2001	2636
Aff2	42	59	50	60
Aff3	436	318	387	560
Aff4	1077	1370	1747	1540

Afg3l1	907	1338	1055	1269
Afg3l2	1148	1611	1435	1350
Afmid	79	57	68	178
Aftph	973	792	1046	884
Aga	1277	1566	1511	2498
Agap1	872	1488	940	1068
Agap2	81	69	80	107
Agap3	1281	1586	1395	1050
Agbl1	257	304	167	312
Agbl3	50	81	15	53
Agbl5	66	59	156	68
Ager	161	116	80	43
Agfg1	1050	1219	879	1333
Agfg2	659	610	430	535
Aggf1	815	681	686	536
Agk	366	384	271	343
Agl	5131	2802	1881	3599
Agmat	55	72	71	76
Ago1	1436	662	1283	803
Ago2	134	233	125	128
Ago3	47	44	35	26
Ago4	390	171	223	184
Agpat1	1669	1924	1255	2029
Agpat2	598	623	712	546
Agpat3	7377	3739	4044	5645
Agpat4	478	1270	703	1419
Agpat5	536	665	555	671
Agps	51	66	113	96
Agrn	8159	4138	6820	3619
Agt	368	326	433	129
Agtpbp1	1085	1064	945	1006
Agtr1a	307	386	456	848
Agtrap	828	697	900	1200
Ahctf1	703	1140	1247	964
Ahcy	2046	2600	2073	2054
Ahcyl1	4523	3429	4517	5708
Ahcyl2	457	506	513	443
Ahdc1	500	542	543	307
Ahi1	67	72	42	19
Ahnak	33488	33010	33310	33608
Ahnak2	5377	4096	4055	4242
Ahr	1275	399	504	620
Ahrr	218	147	141	442
Ahsa1	3185	4384	3414	3115
Ahsa2	1556	1195	1429	1664
Ahsp	0	9	6	11
Aida	2034	2802	2328	2903

Aif1	4626	4684	6327	10062
Aif1l	742	1368	1411	866
Aifm1	707	821	781	1424
Aifm2	575	403	532	258
Aifm3	23	18	17	2
Aig1	1312	2258	1473	1743
Aim1	5288	2816	4308	1895
Aim1l	623	402	436	209
Aim2	56	75	83	71
Aimp1	2535	2941	2427	2304
Aimp2	3185	3245	2172	2127
Aip	1121	1069	817	875
Ajap1	6	41	6	2
Ajuba	2478	1715	2045	978
Ak1	78	184	35	93
Ak2	3436	2840	3385	3158
Ak3	5327	964	1496	3453
Ak4	474	206	570	512
Ak5	518	136	373	428
Ak6	2402	2394	2576	2905
Akap1	799	1215	803	818
Akap10	307	311	292	488
Akap11	767	356	650	828
Akap12	415	520	500	774
Akap13	1425	1850	1945	2726
Akap17a	1019	905	1092	865
Akap17b	51	63	42	67
Akap2	907	1502	1374	1222
Akap6	453	539	456	241
Akap7	318	315	403	926
Akap8	2673	1592	2144	2001
Akap8l	800	540	727	638
Akap9	950	1133	1333	1054
Akip1	964	2220	1710	1108
Akirin1	1254	812	1124	1051
Akirin2	2125	1482	1840	1879
Akna	421	393	493	758
Aknad1	75	37	26	0
Akr1a1	20975	19264	23890	25062
Akr1b1	4595	6639	4846	6188
Akr1b10	728	2089	1341	1049
Akr1c12	69	1	11	22
Akr1c13	126	71	138	29
Akr1c14	698	474	1145	661
Akr1c19	810	444	701	312
Akr1c2	1100	688	325	435
Akr1cl	904	455	1091	936

Akr1e2	467	334	372	495
Akr7a2	1066	937	978	756
Akr7a3	16	0	0	19
Akt1	3202	6082	3739	3845
Akt1s1	1531	1683	963	1067
Akt2	3569	4514	3692	3514
Akt3	176	231	200	213
Aktip	2243	1956	2468	2226
Alad	1040	655	903	622
Alas1	1637	1662	1408	1557
Alas2	6	38	65	7
Alcam	3303	1541	2817	3720
Aldh16a1	1169	1555	918	937
Aldh1a1	1726	694	1098	1021
Aldh1a2	45	92	87	36
Aldh1a3	617	116	686	152
Aldh1l1	875	525	743	402
Aldh1l2	734	2251	1509	3241
Aldh2	1931	2829	1556	2014
Aldh3a1	247	100	89	63
Aldh3a2	656	855	617	909
Aldh3b1	129	154	245	207
Aldh3b2	883	391	510	226
Aldh4a1	1793	1344	1295	1162
Aldh5a1	520	317	497	443
Aldh6a1	339	429	303	290
Aldh7a1	1965	2394	2451	2104
Aldh9a1	95	277	208	221
Aldoa	101015	55816	34071	83488
Aldoart2	141	166	108	163
Aldob	14	29	6	0
Aldoc	234	135	257	163
Alg1	320	423	255	567
Alg10	52	7	84	80
Alg11	806	761	828	773
Alg12	323	354	301	458
Alg13	487	509	575	650
Alg14	831	783	861	991
Alg2	739	576	882	995
Alg3	452	520	396	545
Alg5	1142	890	1336	1774
Alg6	218	165	170	223
Alg8	462	415	342	368
Alg9	617	470	489	857
Alkbh1	646	426	489	593
Alkbh2	94	127	90	119
Alkbh3	1862	2056	2188	1943

Alkbh4	134	78	140	107
Alkbh5	1604	1638	1864	2052
Alkbh7	370	338	421	398
Alkbh8	200	114	114	146
Alms1	430	489	746	455
Alox12e	323	16	107	45
Alox5	773	1658	1086	598
Alox5ap	759	1860	865	1460
Aloxe3	334	242	152	118
Alpk1	502	492	527	509
Alpk2	34	56	5	0
Alpk3	2522	2447	1711	2299
Alpl	402	512	330	1412
Alppl2	39	44	8	12
Als2	393	567	462	419
Als2cl	550	616	351	345
Als2cr12	84	73	65	0
Alx3	9	7	2	38
Alyref	2573	2937	2829	2617
Amacr	315	349	438	459
Ambra1	745	858	930	603
Amd1	2384	1837	2272	1702
Amdhd2	511	847	715	880
Amer1	9	14	9	0
Amfr	4115	4085	3946	4667
Amhr2	22	0	6	1
Amigo1	313	239	323	192
Amigo2	117	208	244	103
Amigo3	238	553	296	519
Ammecr1	919	528	885	885
Ammecr1l	166	165	164	129
Amn1	370	175	364	302
Amot	1138	427	509	808
Amotl1	7830	5604	4936	5201
Amotl2	639	652	757	700
Ampd1	7481	7073	4487	3964
Ampd2	326	692	393	517
Ampd3	933	1360	1223	566
Amt	87	57	184	111
Amz1	101	156	147	153
Amz2	1111	1135	939	1190
Anapc1	1511	1540	1404	1621
Anapc10	699	607	521	593
Anapc11	1365	1923	1396	1464
Anapc13	2087	2225	1896	1453
Anapc15	1166	1487	1169	1223
Anapc16	1719	1509	1652	1628

Anapc2	1786	2299	1724	2017
Anapc4	1649	1956	2120	1865
Anapc5	5372	7259	6265	6632
Anapc7	523	769	475	567
Ang	6155	8229	7548	10201
Angel1	484	373	426	236
Angel2	833	1123	1150	1175
Angpt1	85	104	122	254
Angpt2	363	1214	563	774
Angpt4	128	587	221	327
Angptl1	340	1218	528	316
Angptl2	2934	7110	4365	6698
Angptl4	1757	2419	1888	2176
Angptl8	4	34	26	26
Ank1	2202	3325	1062	1615
Ank3	2040	962	1292	861
Ankdd1a	12	27	41	66
Ankdd1b	36	43	20	13
Ankfy1	1236	1560	1669	1444
Ankh	2626	2887	3214	4686
Ankhd1	1980	1570	1816	1341
Ankib1	252	219	283	392
Ankle1	263	230	170	144
Ankle2	1094	864	1109	985
Ankmy1	43	116	143	32
Ankmy2	1549	1317	1670	1513
Ankrd1	8967	20327	12300	8102
Ankrd10	591	846	701	574
Ankrd11	1565	1549	1703	1287
Ankrd12	420	431	608	795
Ankrd13a	3889	4386	3656	3450
Ankrd13b	12	115	16	9
Ankrd13c	411	310	190	142
Ankrd13d	172	86	138	60
Ankrd16	51	67	48	26
Ankrd17	2076	1395	1781	1780
Ankrd2	2882	2737	1640	860
Ankrd22	1240	359	492	349
Ankrd23	11727	6460	4888	5753
Ankrd26	14	44	29	0
Ankrd27	29	93	57	49
Ankrd28	664	985	618	346
Ankrd29	144	113	75	159
Ankrd33b	26	61	23	10
Ankrd34a	19	19	26	14
Ankrd35	259	123	141	35
Ankrd37	108	149	84	69

Ankrd39	172	189	199	96
Ankrd40	601	816	593	754
Ankrd42	171	124	251	118
Ankrd44	605	415	490	891
Ankrd45	7	0	5	29
Ankrd46	1185	953	841	790
Ankrd49	1287	1030	1274	1208
Ankrd50	947	676	984	682
Ankrd52	24	44	20	16
Ankrd53	79	2	8	8
Ankrd54	353	393	424	269
Ankrd55	69	17	110	33
Ankrd6	195	75	164	124
Ankrd63	389	223	406	874
Ankrd66	14	0	39	77
Ankrd9	330	280	125	90
Anks1a	387	396	385	375
Anks3	212	362	173	125
Anks4b	61	13	30	76
Anks6	121	122	98	130
Ankzf1	842	1066	952	1484
Anln	132	357	238	244
Anlnl1	40	145	102	23
Ano1	368	469	206	189
Ano10	1051	1220	1064	1391
Ano2	14	16	0	53
Ano5	2007	1101	605	1495
Ano6	2177	3371	3056	3825
Ano8	46	219	60	83
Ano9	299	193	284	61
Anp32a	200	427	305	368
Anp32b	6035	7216	7964	6587
Anp32e	2810	2794	2905	2833
Anpep	3636	6146	7004	14177
Antxr1	2789	4030	4100	6302
Antxr2	1604	2334	1808	2546
Anxa1	26116	25976	29944	29398
Anxa10	14	115	18	12
Anxa11	4356	5080	4673	5433
Anxa2	43748	52973	46035	36086
Anxa3	810	1335	1453	1056
Anxa4	8174	6908	6855	12183
Anxa5	7332	13711	9301	19747
Anxa6	7873	14454	9616	13330
Anxa7	4486	5264	4387	5297
Anxa8	32307	20145	23223	10468
Anxa9	108	18	37	0

Aoc3	2810	3021	3018	1141
Aox1	323	223	235	86
Aox2	45	110	15	19
Aox3	27	12	30	22
Aox4	478	457	159	82
Ap1ar	386	569	524	639
Ap1b1	2071	2347	1816	1721
Ap1g1	1897	1436	1879	2062
Ap1g2	1438	1702	1487	1604
Ap1m1	660	1374	603	1107
Ap1m2	839	457	569	351
Ap1s1	1899	2861	2221	2734
Ap1s2	1685	1338	975	1769
Ap1s3	179	107	113	115
Ap2a1	960	1354	931	956
Ap2a2	4025	6112	4150	6374
Ap2b1	111	154	156	215
Ap2m1	10501	12984	12034	12872
Ap2s1	2781	3561	2798	3364
Ap3b1	2151	2140	2380	2406
Ap3b2	44	6	24	10
Ap3d1	644	1605	1260	1968
Ap3m1	1183	1430	1395	1685
Ap3m2	503	359	642	503
Ap3s1	708	661	671	1308
Ap3s2	1241	1353	1294	1058
Ap4b1	831	821	818	629
Ap4e1	22	34	63	20
Ap4m1	71	211	107	318
Ap4s1	1639	922	1074	1141
Ap5b1	403	254	187	273
Ap5m1	153	203	126	251
Ap5s1	126	131	129	162
Ap5z1	579	556	515	661
Apaf1	396	448	521	591
Apba3	213	400	302	191
Apbb1	261	661	342	337
Apbb1ip	537	641	576	1343
Apbb2	1349	1687	1422	1768
Apbb3	2991	3381	3092	3355
Apc	1177	1203	1447	1753
Apc2	59	101	98	58
Apcdd1	2800	1136	1688	2583
Apcdd1l	15	0	2	7
Apeh	1330	1470	998	1440
Apex1	4426	3653	4628	3945
Apex2	226	291	257	220

Aph1a	2068	2786	2864	3012
Api5	3545	1697	2734	3099
Apip	724	488	611	393
Apitd1	265	384	311	114
Aplf	369	418	418	457
Apln	115	478	152	196
Aplnr	512	1702	1470	1239
Aplp1	513	577	594	603
Aplp2	8076	8268	7849	7704
Apmap	1146	1327	1795	1291
Apoa1	21	14	33	15
Apobec1	611	737	1041	1177
Apobec2	8887	5491	3083	6258
Apobec3b	146	268	122	146
Apobr	403	383	272	709
Apoc1	2014	709	815	874
Apoc2	101	0	137	20
Apoc3	14	2	38	34
Apoc4	27	39	29	67
Apod	807	647	1682	462
Apoe	121938	97374	122169	130577
Apoh	12	11	11	41
Apol11a	644	259	575	613
Apol3	171	312	450	634
Apol9a	899	1004	972	1169
Apold1	666	570	1178	506
Apom	165	72	104	36
Apoo	1116	477	472	1195
Apool	452	217	540	511
App	11162	12320	12265	15165
Appbp2	1733	1414	1602	1644
Appl1	1323	1305	1130	1600
Appl2	650	659	718	586
Aprt	5443	6006	5308	5575
Aptr	32	0	39	36
Aptx	300	209	302	220
Aqp1	6019	11700	6023	4819
Aqp11	93	5	27	20
Aqp3	1529	4536	2239	1115
Aqp4	149	0	6	237
Aqp5	38	15	41	11
Aqp7	35	0	23	7
Aqp9	210	158	245	127
Aqr	575	935	534	998
Ar	1	7	9	15
Araf	9012	26411	17179	21632
Arap1	1896	2421	2155	2358

Arap2	257	129	268	67
Arap3	243	689	677	571
Arc	86	71	65	41
Arcn1	3473	5390	4774	6706
Areg	204	435	480	111
Arel1	544	516	442	473
Arf1	10049	12681	9638	13337
Arf2	967	1365	1220	1262
Arf3	14	45	6	7
Arf4	5450	6328	6393	10596
Arf5	7269	6285	6769	6613
Arf6	0	13	11	0
Arfgap1	2565	3022	2645	3681
Arfgap2	1816	3019	2686	2024
Arfgap3	709	1464	1154	1428
Arfgef1	1469	864	1592	1159
Arfgef2	684	1016	715	754
Arfip1	1316	814	1462	1462
Arfip2	1672	1766	1309	1985
Arfrp1	1697	1271	1541	1402
Arg1	1224	1530	1706	1714
Arg2	206	69	93	118
Arglu1	3937	2901	3564	2463
Arhgap1	1891	2279	1834	2444
Arhgap10	388	992	412	893
Arhgap11a	421	630	644	467
Arhgap12	329	268	310	477
Arhgap15	287	111	205	354
Arhgap17	1435	1478	1732	1952
Arhgap18	702	1292	1551	1865
Arhgap19	161	208	320	147
Arhgap20	65	182	214	97
Arhgap21	268	642	390	229
Arhgap22	179	414	349	679
Arhgap23	972	1136	1744	1421
Arhgap24	486	558	712	680
Arhgap25	478	457	874	1512
Arhgap26	192	312	209	413
Arhgap27	367	605	448	571
Arhgap28	544	447	794	907
Arhgap29	540	720	752	707
Arhgap30	201	410	406	680
Arhgap31	370	578	554	603
Arhgap32	506	409	667	340
Arhgap33	63	131	83	39
Arhgap35	1285	2014	1414	1643
Arhgap39	72	184	113	113

Arhgap4	332	323	316	535
Arhgap40	13	3	6	0
Arhgap42	781	662	900	834
Arhgap44	10	139	6	24
Arhgap45	573	691	688	942
Arhgap5	304	191	259	153
Arhgap6	41	74	65	171
Arhgap8	233	52	53	85
Arhgap9	1073	1070	1245	1662
Arhgdia	7064	13913	8253	11378
Arhgdib	8794	8591	9549	13791
Arhgef1	2248	3053	2607	2061
Arhgef10	604	854	605	620
Arhgef10l	509	744	811	720
Arhgef11	264	874	394	432
Arhgef12	539	658	581	613
Arhgef16	131	186	105	119
Arhgef18	393	430	400	444
Arhgef19	740	402	718	336
Arhgef2	1942	2283	2155	2253
Arhgef25	565	1172	844	1377
Arhgef26	43	40	42	27
Arhgef28	298	244	241	193
Arhgef3	948	1104	1562	1288
Arhgef33	41	35	54	53
Arhgef37	629	418	554	267
Arhgef39	50	53	55	29
Arhgef4	2103	1649	1678	1375
Arhgef40	1070	1516	1360	1048
Arhgef5	1194	708	987	386
Arhgef6	766	793	877	1169
Arhgef7	1485	2219	1580	1914
Arhgef9	13	33	20	14
Arid1a	1207	1349	1643	1508
Arid1b	707	656	852	515
Arid2	762	451	698	871
Arid3a	163	180	248	244
Arid3b	103	74	111	67
Arid4a	207	107	238	173
Arid5a	1012	852	951	832
Arid5b	39	86	39	81
Arih1	2047	1725	1812	1907
Arih2	1769	1628	1491	1761
Arl1	6426	5629	5472	7320
Arl10	540	351	578	368
Arl11	891	2029	1288	3107
Arl13b	368	252	280	162

Arl14ep	509	387	593	590
Arl15	1823	609	1471	801
Arl16	321	467	240	354
Arl2	538	569	423	462
Arl2bp	2512	2561	2701	3508
Arl3	815	1413	865	1001
Arl4a	2081	1279	1535	1101
Arl4c	3271	1946	3361	4964
Arl4d	318	352	287	78
Arl5a	3017	2735	2973	3474
Arl5b	1280	759	1339	731
Arl6	204	196	175	215
Arl6ip1	2947	2273	3292	2716
Arl6ip4	661	955	620	819
Arl6ip5	4612	7743	5489	6501
Arl6ip6	179	94	214	287
Arl8a	1205	2241	1550	2477
Arl8b	4150	2823	4299	5608
Armc1	896	1205	958	1409
Armc10	936	1037	1339	1220
Armc2	14	12	29	33
Armc3	48	58	50	35
Armc5	600	701	808	690
Armc6	38	173	32	68
Armc7	13	1	29	29
Armc8	906	622	648	517
Armc9	354	426	391	226
Armcx2	455	624	728	713
Armcx3	1341	1431	1553	2550
Armcx4	410	668	512	690
Armcx6	39	128	105	81
Armt1	489	362	357	490
Arnt	862	1193	1453	1194
Arnt2	9	19	29	132
Arntl	284	345	433	366
Arpc1a	2521	2412	2215	2050
Arpc1b	7229	6528	6291	7176
Arpc2	10486	13460	13260	18559
Arpc3	8635	8742	8647	10934
Arpc4	5894	5936	5382	7254
Arpc5	8911	6735	8559	12071
Arpc5l	3160	1926	2192	1682
Arpin	414	421	469	430
Arpp19	3097	1931	2513	2435
Arpp21	342	451	346	179
Arrb1	862	1720	1626	2756
Arrb2	726	578	719	1150

Arrdc1	1095	1192	1482	1197
Arrdc2	375	298	268	219
Arrdc3	1755	1140	2352	1134
Arrdc4	809	373	1175	774
Arsa	1281	1468	1783	1428
Arsb	1114	2540	1974	3616
Arse	1115	570	818	617
Arsg	667	1088	1118	1339
Arsi	470	1177	558	329
Arsj	0	11	11	17
Arsk	84	36	71	165
Art1	2037	2687	1439	1790
Art3	3362	2482	1992	2323
Art4	163	28	166	73
Art5	344	743	302	247
Artn	60	98	47	20
Arv1	441	311	381	288
Arvcf	595	418	501	289
Arx	16	16	6	32
Arxes2	23	0	3	0
As3mt	564	430	444	523
Asah1	1094	1594	1279	2887
Asah2	87	6	68	68
Asap1	1191	1624	1530	2140
Asap2	478	669	493	658
Asap3	203	92	209	409
Asb1	325	404	345	343
Asb10	257	57	47	230
Asb11	639	161	107	554
Asb12	617	556	333	454
Asb13	39	12	57	7
Asb14	426	239	169	178
Asb15	1	11	20	0
Asb16	678	337	280	777
Asb18	8	3	2	0
Asb2	145	158	81	121
Asb3	360	325	331	362
Asb4	28	9	3	25
Asb5	504	944	981	468
Asb6	71	196	55	46
Asb7	36	7	26	22
Asb8	2012	1619	2144	1703
Asb9	14	0	12	29
Ascc1	911	1068	936	1020
Ascc2	341	507	278	359
Ascc3	472	481	540	495
Ascl2	46	54	42	23

Ascl3	9	25	18	29
Ascl4	56	0	35	23
Asf1a	536	342	487	635
Asf1b	380	752	575	390
Asgr1	23	4	15	18
Asgr2	790	2765	1303	1218
Ash1l	826	867	1022	819
Ash2l	1722	2037	1691	1825
Asic1	13	40	21	11
Asic2	10	10	21	6
Asl	232	591	378	481
Asmtl	412	499	367	350
Asna1	1545	2090	1213	1779
Asns	948	1611	1058	682
Asnsd1	1769	1776	1541	1901
Aspa	96	241	164	234
Aspg	111	54	120	69
Asph	3897	2547	2691	3516
Aspm	340	287	208	190
Asprv1	8659	3420	2343	2076
Aspscr1	644	943	509	483
Asrgl1	290	372	348	530
Ass1	684	458	572	443
Aste1	136	79	119	66
Astn1	32	26	51	44
Astn2	3	6	3	5
Asun	682	819	883	625
Asxl1	924	1055	1211	937
Asxl2	481	560	558	686
Asxl3	14	0	26	22
Atad1	1467	1285	1583	1401
Atad2	226	256	307	283
Atad2b	19	18	5	0
Atad3a	853	1010	593	505
Atad5	137	129	131	171
Atat1	64	167	59	67
Ate1	36	39	23	29
Atf1	1095	1030	1354	1315
Atf2	75	149	138	69
Atf3	2867	1016	1834	2008
Atf4	12748	12730	13493	13040
Atf5	3250	3562	3919	2341
Atf6	2268	2933	3155	3485
Atf6b	1326	1614	1532	1494
Atf7ip	1587	1470	1533	1322
Atf7ip2	4	20	14	0
Atg10	315	189	325	459

Atg101	999	1166	712	716
Atg12	1688	1081	1420	1794
Atg13	1307	1494	1482	1334
Atg14	316	165	259	172
Atg16l1	921	899	707	849
Atg16l2	251	188	141	155
Atg2a	695	674	563	423
Atg2b	877	922	1121	1133
Atg3	1332	1918	2177	2195
Atg4a	644	702	405	861
Atg4b	911	1003	828	991
Atg4c	302	196	358	547
Atg4d	728	490	544	532
Atg5	882	863	859	667
Atg7	505	315	388	753
Atg9a	1568	1922	1452	1667
Atg9b	1536	296	711	202
Atic	50	270	128	203
Atl1	31	56	53	2
Atl2	119	204	242	247
Atl3	295	399	245	359
Atm	475	418	527	555
Atmin	151	114	78	177
Atoh8	99	143	96	117
Atox1	4891	3673	4380	4655
Atp10a	223	508	354	422
Atp10b	186	9	108	0
Atp10d	1128	917	1213	1262
Atp11a	923	1409	1453	1593
Atp11b	1168	668	1115	872
Atp11c	92	144	224	216
Atp12a	132	14	44	0
Atp13a1	682	974	542	697
Atp13a2	1041	774	987	912
Atp13a4	117	66	105	0
Atp13a5	4	0	0	14
Atp1a1	8405	11395	10955	10660
Atp1a2	7847	4756	4213	5745
Atp1a3	168	492	624	3084
Atp1b1	2083	1585	2078	3825
Atp1b2	2830	1703	1228	1983
Atp1b3	14043	8702	12634	8778
Atp1b4	151	448	162	128
Atp23	685	401	403	438
Atp2a1	121382	72573	22337	97184
Atp2a2	5191	8646	5353	6680
Atp2a3	489	608	507	462

Atp2b1	1028	1242	1518	1621
Atp2b3	148	153	56	93
Atp2b4	3491	2759	3459	3121
Atp2c1	1244	1073	1125	849
Atp2c2	126	73	66	64
Atp5a1	20726	20692	15228	18623
Atp5b	52706	56056	41212	52534
Atp5c1	11455	10151	7855	11182
Atp5d	4598	6108	3087	3196
Atp5e	10916	10093	8120	8294
Atp5g1	3879	3791	2944	3290
Atp5g2	10837	10260	7658	7982
Atp5g3	24328	14702	14689	16908
Atp5h	16992	12990	15617	15037
Atp5i	9106	7751	6646	5860
Atp5j	15855	9456	11185	11206
Atp5j2	10729	9699	7950	9087
Atp5l	14597	10711	11147	12326
Atp5o	14754	11980	9426	11618
Atp5s	296	279	245	160
Atp5sl	1110	928	1125	1098
Atp6ap1	7852	6636	9032	10424
Atp6ap1l	0	8	6	0
Atp6ap2	1883	1335	1667	4808
Atp6v0a1	1953	2793	2252	2687
Atp6v0a2	1155	1322	1115	1055
Atp6v0a4	830	89	812	240
Atp6v0b	2748	2831	2720	4547
Atp6v0c	9621	10847	10726	15743
Atp6v0d1	6303	6863	8292	10394
Atp6v0d2	237	313	474	2205
Atp6v0e1	6725	4737	5877	7088
Atp6v0e2	805	1018	806	1039
Atp6v1a	1713	1321	1560	3436
Atp6v1b1	7	0	5	0
Atp6v1b2	5217	4722	5976	11850
Atp6v1c1	2844	2195	2741	3894
Atp6v1c2	1402	354	766	351
Atp6v1d	4764	3351	3945	4248
Atp6v1e1	6352	4484	6616	6460
Atp6v1e2	21	65	59	2
Atp6v1f	3214	3086	2869	3588
Atp6v1g1	8660	8370	8742	9043
Atp6v1h	1900	1840	2076	3273
Atp7a	824	461	821	615
Atp8a1	1000	655	615	825
Atp8a2	123	108	119	53

Atp8b2	2516	2935	2818	3828
Atp8b4	50	86	39	131
Atp8b5p	14	10	3	17
Atp9a	1600	1207	1068	915
Atp9b	947	1129	862	712
Atpaf1	837	631	582	630
Atpaf2	659	677	657	655
Atpif1	8464	5803	7746	6173
Atr	514	334	424	294
Atraid	1307	1531	1300	1416
Atrip	529	750	531	560
Atrn	1216	1571	1474	1615
Atrnl1	919	986	1181	1683
Atrx	353	262	340	329
Atxn1	20	67	32	21
Atxn10	3435	4242	3643	5651
Atxn2	455	126	228	342
Atxn2l	1484	1453	1644	1326
Atxn3	1072	767	1008	765
Atxn7	332	180	369	264
Atxn7l1	703	781	662	1042
Atxn7l2	117	154	108	59
Atxn7l3	152	273	95	168
Atxn7l3b	2570	1876	2616	2316
Auh	656	868	667	1142
Aunip	14	21	20	27
Aup1	3019	3298	2057	3239
Aurka	1498	1118	961	1236
Aurkaip1	4484	4672	4395	3832
Aurkb	507	756	689	597
Aurkc	169	105	211	80
Aven	1311	1141	1333	1238
Avil	72	60	57	66
Avl9	122	45	147	122
Avpi1	2329	2331	2079	1882
Avpr1a	77	119	93	209
Awat1	3578	1097	1052	1625
Awat2	6832	3558	2291	3516
Axdnd1	27	57	17	1
Axin2	41	103	60	56
Axl	3809	4907	3948	4878
Azi2	1276	1289	1306	1678
Azin1	1277	1129	1148	1941
Azin2	72	64	39	36
B2m	63159	54684	65252	91082
B3galnt1	1095	718	784	1255
B3galnt2	156	233	150	126

B3galt1	10	0	3	0
B3galt4	262	267	241	254
B3galt6	941	683	829	688
B3gat3	1675	2128	1864	2274
B3glct	544	533	554	832
B3gnt2	805	1195	1092	783
B3gnt3	48	19	18	0
B3gnt5	38	25	11	76
B3gnt7	160	246	260	237
B3gnt8	127	45	107	27
B3gntl1	167	149	222	243
B4galnt1	180	165	170	192
B4galnt2	18	64	30	82
B4galnt3	39	15	41	0
B4galnt4	125	100	110	24
B4galt1	2837	3625	3632	4414
B4galt2	257	724	376	841
B4galt3	935	715	916	739
B4galt4	126	178	199	167
B4galt5	979	1814	1196	2751
B4galt6	584	845	755	1656
B4galt7	463	720	617	915
B9d2	156	484	278	236
Baalc	25	113	89	39
Babam1	1320	1576	1213	955
Bace1	1144	1324	1423	1364
Bace2	1344	1181	1620	1263
Bach1	34	18	39	18
Bach2	4	26	20	0
Bad	1062	1224	864	1054
Bag1	5488	4659	5949	5891
Bag2	448	699	245	639
Bag3	4059	3808	3208	3197
Bag4	55	34	57	54
Bag6	2598	3193	2382	2168
Bahcc1	188	332	253	182
Bahd1	684	650	940	710
Baiap2	985	1179	938	1224
Baiap2l1	316	612	265	172
Baiap2l2	29	28	21	126
Bak1	18	0	5	0
Bambi	43	50	89	57
Banf1	6557	6807	4685	4789
Banp	343	344	218	328
Bap1	1202	1796	1294	1494
Bard1	23	19	24	29
Barx2	1827	719	1762	427

Basp1	2355	1523	1988	2956
Batf	120	170	125	105
Batf2	62	15	8	5
Batf3	48	70	57	60
Bax	1001	1288	970	1394
Baz1a	548	645	812	984
Baz1b	1366	1859	1566	1646
Baz2a	93	139	164	90
Baz2b	847	546	651	624
Bbc3	170	75	81	180
Bbip1	325	252	381	702
Bbof1	307	67	232	344
Bbox1	721	86	301	200
Bbs1	333	307	236	200
Bbs10	130	87	146	117
Bbs12	127	109	86	152
Bbs2	822	699	946	944
Bbs5	145	47	104	129
Bbs9	300	232	263	369
Bbx	7	42	6	0
Bc1	1	5	5	2
Bcam	3032	3246	2806	1231
Bcap29	756	275	385	686
Bcap31	5825	4612	5559	7797
Bcar1	350	935	618	699
Bcar3	270	572	474	515
Bcas1	9	109	69	37
Bcas2	1819	1583	1556	1638
Bcas3	1095	945	945	923
Bcat1	3769	4284	3946	7532
Bcat2	2516	1483	1476	2299
Bccip	1011	1551	1312	1431
Bcdin3d	98	90	95	79
Bche	10	25	9	0
Bckdha	845	1232	662	871
Bckdhb	1935	1149	1050	1944
Bckdk	1789	2576	1738	1590
Bcl10	1713	1140	1646	1495
Bcl11a	269	176	247	104
Bcl11b	504	180	224	162
Bcl2	9	0	8	16
Bcl2a1	2972	2502	3432	5690
Bcl2l1	424	760	429	883
Bcl2l11	680	472	692	496
Bcl2l12	255	225	281	213
Bcl2l13	754	798	752	696
Bcl2l14	167	54	65	150

Bcl2l15	5427	645	4317	841
Bcl2l2	1191	1454	1396	972
Bcl3	705	810	829	1089
Bcl6	1881	1093	1375	1337
Bcl6b	399	851	888	821
Bcl7a	217	275	293	210
Bcl7b	340	590	454	405
Bcl7c	1767	2074	1484	1438
Bcl9	279	263	302	297
Bcl9l	766	1247	986	639
Bclaf1	2451	2023	2996	2529
Bco2	139	237	206	174
Bcor	240	460	423	553
Bcorl1	148	217	173	213
Bcr	179	608	430	270
Bcs1l	588	701	399	522
Bdh1	1387	970	967	747
Bdh2	258	236	308	285
Bdkrb1	15	21	20	68
Bdkrb2	491	482	531	469
Bdnf	61	14	75	38
Bdp1	519	458	557	419
Bean1	138	125	117	130
Becn1	3008	3289	3584	3424
Begain	70	44	143	30
Bend3	389	214	302	234
Bend5	53	41	71	96
Bend6	111	332	123	385
Best3	26	7	18	26
Bet1	490	550	686	1016
Bet1l	1227	1205	1159	1534
Bex2	16	0	9	0
Bex3	1860	929	1411	1405
Bex4	5	12	3	24
Bfar	1200	1486	1559	1706
Bfsp1	174	148	193	142
Bfsp2	69	72	48	26
Bgn	15575	35842	21068	44665
Bhlhb9	405	286	322	339
Bhlhe22	13	7	27	49
Bhlhe23	9	11	30	7
Bhlhe40	2842	2115	3026	2806
Bhlhe41	1367	1183	1372	2405
Bicc1	685	808	710	1406
Bicd2	3377	2621	2999	2510
Bicdl1	25	45	70	36
Bicdl2	178	103	188	64

Bid	2242	1741	1700	2166
Bik	42	49	27	16
Bin1	5972	10800	4451	6255
Bin2	349	472	540	842
Bin2a	57	0	9	56
Bin3	1526	1178	1503	1409
Birc2	2208	1838	2257	3047
Birc5	361	600	406	459
Birc6	807	1175	1068	1013
Bivm	311	279	281	407
Blcap	1643	1188	1004	1314
Bles03	1017	1164	1056	1066
Blk	2	29	12	21
Blm	164	401	251	219
Blmh	3170	3169	3390	2840
Blnk	654	580	739	995
Bloc1s1	1342	1957	1351	1352
Bloc1s2	1749	1883	1891	2633
Bloc1s3	378	399	322	213
Bloc1s4	422	432	378	490
Bloc1s5	1002	714	886	1026
Bloc1s6	840	554	835	519
Blvra	473	783	636	915
Blvrb	1628	2082	1228	1419
Blzf1	433	463	536	452
Bmf	535	864	817	1030
Bmi1	824	212	290	607
Bmp1	5334	8946	6300	8684
Bmp2	485	335	354	177
Bmp2k	463	957	946	980
Bmp3	16	43	3	11
Bmp4	171	182	170	111
Bmp5	58	16	12	52
Bmp6	343	242	251	313
Bmp7	851	539	942	380
Bmper	110	200	104	160
Bmpr1a	332	237	444	318
Bmpr1b	54	152	57	59
Bmpr2	2609	1865	2931	2748
Bms1	892	1170	1055	824
Bmt2	26	23	45	17
Bmx	48	28	78	20
Bmyc	25	13	12	8
Bnc1	37	4	63	1
Bnc2	842	751	1016	758
Bnip1	821	938	582	854
Bnip2	3742	3985	3647	4822

Bnip3	1812	1148	1119	1869
Bnip3l	4970	2807	3274	4642
Bnipl	1238	371	736	223
Boc	1687	1919	1505	1362
Bod1	1166	1526	1360	1637
Bod1l1	405	497	487	388
Bok	1887	1386	2016	895
Bola1	389	340	259	127
Bola3	1125	1303	949	983
Bop1	1044	1094	919	829
Bora	367	307	333	290
Borcs5	405	446	384	325
Borcs6	835	648	919	784
Borcs7	428	657	524	753
Borcs8	822	854	840	770
Bpgm	2691	2022	1882	2446
Bphl	191	175	122	91
Bpifb5	3	12	0	11
Bpifc	632	196	636	146
Bpnt1	674	533	593	566
Bptf	896	712	1022	999
Braf	276	327	430	272
Brap	1005	874	1115	1022
Brat1	446	448	302	292
Brca1	87	126	158	281
Brca2	177	249	211	204
Brcc3	218	187	167	253
Brd1	592	559	718	698
Brd3	1275	1291	1068	849
Brd4	2311	2295	2313	2303
Brd7	3246	3122	3765	4875
Brd8	965	986	1133	1123
Brd9	1001	1079	838	818
Bre	5798	5982	5731	5676
Brf1	783	875	800	751
Brf2	640	1890	1590	2459
Bri3	1651	1782	1399	1786
Bri3bp	463	374	361	371
Bricd5	2245	1791	1531	2361
Brinp1	96	41	110	14
Brip1	84	71	108	59
Brix1	1417	1489	1356	1235
Brk1	5404	5492	4262	6061
Brms1l	13	4	45	10
Brox	2523	1826	2201	2083
Brpf1	495	566	676	510
Brpf3	110	294	236	153

Brsk1	23	62	27	39
Brwd1	1220	747	1002	907
Brwd3	398	152	152	76
Bscl2	1507	1465	1224	1356
Bsdc1	960	1241	1357	1257
Bsg	13692	16709	14668	16415
Bsn	74	10	39	7
Bspry	783	275	644	439
Bst1	396	823	569	1159
Bst2	1151	1387	855	1458
Btaf1	1198	900	1231	1190
Btbd1	3901	3265	3465	4093
Btbd10	607	821	743	862
Btbd11	835	339	588	211
Btbd16	0	17	9	0
Btbd18	13	0	8	0
Btbd19	719	540	758	802
Btbd2	387	451	421	428
Btbd3	417	461	370	228
Btbd6	1188	1351	1106	1036
Btbd7	1160	1331	1207	1105
Btbd8	96	126	158	32
Btbd9	345	440	286	464
Btd	512	899	633	800
Btf3	7676	6325	7798	6200
Btf3l4	386	228	209	307
Btg1	8664	5418	10335	8446
Btg2	3455	1635	2955	1510
Btg3	867	652	852	511
Btk	220	219	203	659
Btla	20	94	38	11
Btn2a2	19	37	53	21
Btnl10	6	15	5	3
Btnl9	59	133	193	213
Btrc	411	548	617	540
Bub1	353	491	421	362
Bub1b	313	539	391	386
Bub3	2934	2370	2901	2061
Bud13	366	546	460	503
Bud31	2665	3174	3113	3079
Bves	382	316	209	186
Bysl	1151	1771	627	1094
Bzw1	6301	5019	5352	5582
Bzw2	833	1118	1402	943
C11H22orf	856	607	963	452
C1d	338	127	345	377
C1galt1	318	211	336	486

C1galt1c1	498	505	474	535
C1H10orf7	16	63	84	23
C1H19orf8	22	0	11	52
C1qa	6534	12154	7351	8867
C1qb	7421	11892	8895	7299
C1qbp	5884	6184	6172	6080
C1qc	5076	8242	4797	5833
C1ql1	0	8	2	0
C1ql4	13	0	11	0
C1qtnf1	1782	2686	1899	2826
C1qtnf2	593	889	805	1490
C1qtnf3	793	3156	1420	4773
C1qtnf4	7	2	3	0
C1qtnf5	1419	1994	1866	3310
C1qtnf6	1229	3924	2418	6582
C1qtnf7	240	244	211	346
C1qtnf9	173	119	191	264
C1r	9118	13933	12124	15867
C1s	4069	2136	3220	4213
C2	4647	9966	6294	13387
C2cd2l	746	946	576	1074
C2cd3	182	267	273	146
C2cd4a	5	18	29	10
C2cd4c	42	70	80	34
C2cd4d	0	4	3	13
C2cd5	464	320	405	269
C3	796	505	873	790
C3ar1	768	1357	1435	2917
C4b	245	737	531	401
C4bpb	8	0	24	8
C5ar1	1423	2493	2795	7116
C5ar2	214	425	229	221
C6	544	844	645	300
C7	665	1050	733	1001
C8g	178	234	266	230
Ca5b	769	681	466	622
Caap1	166	189	152	114
Cab39	4480	4033	4250	4895
Cab39l	1464	1556	1187	1671
Cabin1	266	445	308	224
Cables1	493	495	367	368
Cables2	140	623	224	151
Cabp1	6	20	2	0
Cabyr	243	133	146	190
Cachd1	531	288	393	201
Cacna1a	24	41	60	56
Cacna1c	186	349	382	424

Cacna1d	402	179	416	190
Cacna1e	36	12	23	58
Cacna1g	196	218	220	232
Cacna1h	2	37	0	2
Cacna1s	3181	3571	2173	2488
Cacna2d1	3170	2797	1670	2729
Cacna2d2	103	179	221	117
Cacna2d3	46	50	18	5
Cacnb1	1415	2865	1796	1151
Cacnb3	1085	712	891	1077
Cacnb4	37	35	18	2
Cacng1	3427	5790	1477	1853
Cacng4	3	12	0	6
Cacng6	822	441	436	474
Cacng7	14	30	3	6
Cactin	250	315	289	313
Cacul1	1627	1605	2046	2179
Cacybp	1776	1993	1675	1988
Cad	860	1560	1032	937
Cadm1	207	163	170	168
Cadm3	822	1832	642	787
Cadm4	4	31	35	31
Cadps	12	12	0	15
Cadps2	32	40	143	20
Cage1	18	53	30	27
Cahm	25	8	5	0
Calca	156	16	20	24
Calcb	143	26	65	32
Calcrl	173	169	280	172
Cald1	1129	2957	2971	3446
Calhm2	862	863	1088	1177
Calm1	23698	31550	27275	36700
Calm2	5078	3455	4207	5483
Calm3	3166	3684	2639	3478
Calml3	11079	3531	7500	3477
Calml4	52	88	39	150
Calml5	5250	2426	3453	985
Calr	31185	39248	33303	51449
Calr3	7	42	72	38
Calr4	60	55	104	47
Calu	7119	18089	13359	17329
Caly	10	9	8	1
Camk1	1112	1099	1108	1959
Camk1d	46	48	51	107
Camk2a	1451	1238	453	712
Camk2b	2311	3533	2270	2144
Camk2d	1446	1188	1256	2413

Camk2n1	77	49	122	113
Camk4	126	19	132	61
Camkk1	710	888	718	743
Camkk2	459	360	400	502
Camkmt	153	150	117	236
Camkv	14	17	11	15
Camlg	809	630	722	718
Camsap1	646	647	713	687
Camsap2	1311	881	1046	1278
Camsap3	1313	729	1064	345
Camta1	842	317	558	597
Camta2	2059	2457	1951	1794
Cand1	2372	2729	3108	3204
Cand2	136	300	151	80
Cant1	1019	941	979	1289
Canx	9189	6804	6694	9332
Cap1	10233	10581	10898	13078
Cap2	793	1096	897	731
Capg	11683	10139	10895	12227
Capn1	2741	3162	2477	1922
Capn10	593	477	582	531
Capn12	3523	426	1343	302
Capn13	37	14	6	19
Capn15	240	287	188	214
Capn2	6754	9222	8781	10304
Capn3	1948	1393	1246	1460
Capn5	521	663	500	575
Capn6	78	1419	138	538
Capn7	1163	764	987	794
Capn8	40	9	77	22
Capns2	4371	1170	2550	898
Caprin1	4755	3661	3840	5295
Caprin2	277	196	214	156
Capza1	3046	3554	3430	5150
Capza2	5188	4070	4296	6376
Capzb	6883	9877	7976	10811
Car11	229	219	179	234
Car12	4556	2640	5484	1496
Car13	2218	911	1538	677
Car14	1429	630	787	1428
Car2	1514	386	1071	410
Car3	82899	20077	28778	70843
Car4	495	452	515	202
Car6	170	45	176	53
Car9	90	162	224	181
Card10	1357	1034	825	542
Card11	105	220	56	95

Card14	628	576	769	1160
Card19	347	972	549	679
Card6	509	681	832	457
Card9	1190	1087	732	745
Carf	359	217	326	230
Carhsp1	2486	4815	2728	3381
Carm1	522	1296	796	1368
Carmil1	224	409	397	373
Carmil2	114	34	106	126
Carmil3	10	27	0	8
Carnmt1	851	576	555	929
Carns1	454	339	251	147
Cars	901	1721	967	957
Cars2	475	353	345	312
Casc1	358	420	525	405
Casc3	1960	1460	1938	1582
Casc4	279	476	524	729
Casd1	63	141	175	122
Cask	69	49	90	0
Caskin1	31	106	54	50
Caskin2	404	863	566	425
Casp1	2122	1072	1595	1946
Casp12	168	229	214	189
Casp14	398	186	116	71
Casp16	531	450	429	402
Casp2	968	718	1088	882
Casp3	1591	1791	1845	2222
Casp4	1394	660	978	1204
Casp7	684	615	597	665
Casp8	824	667	787	566
Casp8ap2	118	194	224	172
Casp9	531	594	311	321
Casq1	37150	21960	11551	27262
Casq2	1830	5803	2598	2566
Casr	8	32	0	1
Cass4	229	335	358	531
Cast	2518	2367	2938	3481
Casz1	993	654	943	392
Cat	2894	1943	1708	2368
Catip	11	61	24	40
Catsper2	151	136	83	91
Catsper4	23	11	17	0
Catsperd	38	16	66	23
Catsperg1	99	104	87	86
Cav1	5017	9891	6134	6503
Cav2	621	525	393	466
Cav3	2820	4897	1623	1817

Cbarp	8	17	0	6
Cbfa2t2	744	431	495	517
Cbfa2t3	469	847	617	555
Cbfb	1138	625	734	1090
Cbl	98	66	94	226
Cblb	771	841	721	1763
Cblc	192	251	138	68
Cbll1	383	346	382	539
Cbln1	73	34	62	0
Cbr1	1505	716	931	571
Cbr3	1539	833	1095	691
Cbr4	479	252	310	433
Cbs	1542	362	1438	350
Cbwd1	290	179	119	237
Cbx1	1244	1368	1348	1446
Cbx2	225	175	187	118
Cbx3	1443	1650	1210	1398
Cbx4	18	31	21	35
Cbx5	511	698	701	744
Cbx6	888	1577	1228	1273
Cbx7	264	242	363	243
Cbx8	129	190	104	81
Cby1	384	789	414	597
Cc2d1a	896	934	566	547
Cc2d1b	1758	1149	1517	1286
Cc2d2a	147	222	287	235
Ccar1	807	957	895	1006
Ccar2	1191	1132	1384	1322
Ccbe1	15	110	59	140
Ccdc102a	460	745	524	936
Ccdc106	645	490	632	493
Ccdc107	1448	1871	1425	2096
Ccdc112	116	85	102	86
Ccdc113	35	44	36	26
Ccdc114	1	26	8	4
Ccdc115	13	90	33	21
Ccdc117	199	246	257	165
Ccdc12	1637	1799	1410	1429
Ccdc120	638	215	513	309
Ccdc122	11	64	42	14
Ccdc124	768	1100	533	581
Ccdc125	187	206	299	228
Ccdc126	94	87	83	117
Ccdc127	632	910	852	753
Ccdc130	353	393	286	277
Ccdc134	488	633	498	325
Ccdc136	635	538	561	464

Ccdc137	593	636	544	550
Ccdc138	1269	1372	1757	1559
Ccdc14	179	270	239	233
Ccdc141	114	332	141	159
Ccdc149	284	442	355	394
Ccdc150	212	46	113	58
Ccdc155	12	11	6	15
Ccdc157	12	0	2	3
Ccdc158	137	163	139	136
Ccdc159	123	121	131	209
Ccdc163	98	160	59	111
Ccdc167	200	199	175	263
Ccdc17	496	257	298	344
Ccdc173	11	5	17	0
Ccdc174	377	460	369	414
Ccdc175	526	424	568	607
Ccdc177	17	31	45	0
Ccdc181	524	262	363	439
Ccdc183	913	689	653	869
Ccdc186	587	540	570	552
Ccdc189	69	57	62	7
Ccdc191	54	64	141	71
Ccdc22	1039	987	1188	1347
Ccdc24	152	0	68	0
Ccdc25	1224	1250	1171	1273
Ccdc28a	249	185	134	255
Ccdc28b	392	264	223	308
Ccdc3	561	172	337	247
Ccdc30	46	53	59	44
Ccdc32	981	676	919	1155
Ccdc34	302	330	292	444
Ccdc39	148	106	66	4
Ccdc40	3	6	0	8
Ccdc43	1316	855	772	1059
Ccdc47	1531	1883	1715	2056
Ccdc50	2551	2131	2639	3284
Ccdc51	161	321	172	215
Ccdc53	2150	1616	1942	1892
Ccdc57	5	16	8	44
Ccdc58	691	515	743	725
Ccdc59	739	509	596	540
Ccdc6	1334	1599	1450	1703
Ccdc61	210	191	129	88
Ccdc62	130	137	128	129
Ccdc65	101	66	152	204
Ccdc66	371	437	479	417
Ccdc69	111	81	77	133

Ccdc71	610	594	638	512
Ccdc71l	408	303	533	377
Ccdc73	7	0	9	0
Ccdc74a	88	51	53	55
Ccdc77	119	165	195	190
Ccdc78	8	0	5	0
Ccdc8	774	912	773	1047
Ccdc80	8860	17616	12723	28882
Ccdc81	97	109	36	16
Ccdc82	120	219	323	317
Ccdc84	611	344	497	340
Ccdc85b	362	331	298	107
Ccdc85c	443	293	396	370
Ccdc86	1344	1493	1228	1204
Ccdc88a	332	398	439	603
Ccdc88b	273	241	271	307
Ccdc88c	244	268	196	239
Ccdc9	997	1000	1076	828
Ccdc90b	686	631	773	743
Ccdc91	715	782	620	585
Ccdc92	155	146	117	218
Ccdc93	821	1360	1091	1519
Ccdc94	809	743	721	595
Ccdc96	31	25	18	0
Ccdc97	984	1225	1013	957
Ccer2	67	92	6	40
Cchcr1	323	479	378	317
Cck	22	46	14	12
Ccl1	103	23	33	0
Ccl11	81	67	462	59
Ccl12	564	306	381	332
Ccl17	75	18	87	82
Ccl19	125	29	77	39
Ccl2	3233	2391	2028	2482
Ccl20	327	61	230	38
Ccl21	1259	1751	3942	1612
Ccl22	473	107	290	294
Ccl24	287	63	633	595
Ccl27	2865	951	334	345
Ccl3	1097	2701	1157	1318
Ccl4	325	228	448	285
Ccl5	645	137	411	590
Ccl6	2342	2548	2314	1482
Ccl7	1498	1678	2058	1806
Ccl9	461	928	1494	6038
Ccm2	413	679	375	408
Ccm2l	134	199	230	237

Ccna1	104	92	42	96
Ccna2	630	950	793	913
Ccnb1	836	1302	900	864
Ccnb2	14	15	14	36
Ccnc	906	561	719	955
Ccnd1	1442	3165	1497	1457
Ccnd2	10314	3771	10622	4586
Ccnd3	1521	3973	2024	2094
Ccndbp1	1512	1280	1493	1322
Ccne1	231	381	260	238
Ccne2	113	48	84	52
Ccnf	180	594	330	435
Ccng1	16097	10134	8912	11127
Ccng2	792	633	988	992
Ccnh	1175	900	1321	1241
Ccni	2853	2244	2563	2641
Ccnk	851	665	913	1008
Ccnl1	1074	1167	1259	1141
Ccnl2	2470	2524	2322	1923
Ccnt2	1172	612	885	640
Ccny	2066	1998	2228	2323
Ccnyl1	1003	1374	1261	1260
Ccp110	102	125	117	177
Ccpg1	857	953	1378	1510
Ccpg1os	195	142	149	100
Ccr1	38	115	69	106
Ccr4	23	9	12	14
Ccr5	642	1040	1034	2002
Ccr6	131	50	93	105
Ccr7	184	178	84	225
Ccrl2	173	214	105	196
Ccs	696	925	752	823
Ccsap	223	246	265	178
Ccser1	127	57	96	24
Ccser2	2297	1887	2246	1881
Cct2	2870	3542	3149	3116
Cct3	10066	12099	8499	8934
Cct4	11020	10205	12468	10746
Cct5	10973	11220	9628	9319
Cct6a	3264	3188	3694	3465
Cct6b	27	15	6	11
Cct7	3703	5084	3507	3949
Cct8	8035	7137	8564	7694
Ccz1b	2937	1775	2349	2907
Cd101	12	33	20	26
Cd109	1297	1117	1256	950
Cd14	2334	3612	3843	8826

Cd163	2667	9439	5543	2726
Cd163l1	22	18	20	31
Cd164	6133	4232	4906	9166
Cd164l2	83	35	108	80
Cd180	350	220	349	661
Cd1d1	540	326	451	771
Cd2	415	406	474	651
Cd200	1449	1367	1906	1753
Cd200r1	45	23	53	111
Cd207	2054	605	600	627
Cd209d	55	278	66	19
Cd209e	96	88	21	0
Cd209f	34	95	48	114
Cd22	104	116	81	106
Cd226	42	10	8	6
Cd24	4958	2216	3280	2197
Cd244	102	122	77	181
Cd247	163	21	93	53
Cd27	60	0	38	44
Cd274	403	384	241	220
Cd276	1781	4463	2922	4622
Cd28	25	25	36	129
Cd2ap	236	245	259	191
Cd2bp2	4493	4577	4949	5265
Cd300a	297	630	657	2046
Cd300e	77	73	218	744
Cd300ld	153	379	375	1164
Cd300le	884	1680	1790	4723
Cd300lf	238	40	144	335
Cd300lg	122	77	83	34
Cd302	1177	1343	1149	1941
Cd320	1141	1228	1304	1251
Cd33	835	734	716	1678
Cd34	5734	10063	6768	4156
Cd36	57	109	209	219
Cd37	806	798	782	1217
Cd38	393	618	576	967
Cd3d	126	21	173	61
Cd3e	245	112	209	200
Cd3eap	442	480	363	220
Cd3g	460	70	260	219
Cd4	3335	5720	5923	7400
Cd40	214	118	123	168
Cd44	9431	14001	13337	16081
Cd46	39	18	29	11
Cd47	4317	4587	5434	7081
Cd48	1000	2391	1765	3462

Cd52	32	46	50	54
Cd53	2177	1628	1593	6802
Cd55	1025	1648	688	862
Cd59	6511	10322	6500	7631
Cd5l	101	14	156	149
Cd6	100	98	102	123
Cd63	23249	36938	33535	51516
Cd68	2510	4385	3618	15919
Cd69	211	79	111	30
Cd7	581	138	370	252
Cd70	12	0	2	15
Cd72	141	193	47	128
Cd74	54379	47278	54494	76059
Cd79b	0	27	6	7
Cd80	9	56	15	151
Cd81	7721	11160	6154	9125
Cd82	3733	5632	3763	4434
Cd83	879	418	793	1570
Cd84	310	364	829	1897
Cd86	267	231	286	555
Cd8a	590	1118	1107	2103
Cd8b	52	104	80	236
Cd9	10654	12998	11579	13137
Cd93	1669	5637	3903	4094
Cd96	95	11	62	55
Cd99	686	1432	510	773
Cdadc1	2620	1619	1256	2089
Cdan1	531	447	567	398
Cdc123	1890	2041	2076	2492
Cdc14a	180	153	244	205
Cdc14b	31	0	9	17
Cdc16	1204	1669	1333	1825
Cdc20	677	1319	612	512
Cdc23	1139	1344	1289	1457
Cdc25a	516	647	782	522
Cdc25b	897	1112	823	562
Cdc25c	306	399	444	351
Cdc26	1663	1371	1416	1584
Cdc27	1135	881	928	1073
Cdc34	3164	3236	2316	2042
Cdc37	2886	4498	2761	3199
Cdc37l1	1461	1608	1285	1541
Cdc40	578	484	609	405
Cdc42	18595	15287	18097	20450
Cdc42bpa	108	169	200	246
Cdc42bpb	426	1084	542	479
Cdc42bpg	1576	665	1759	484

Cdc42ep1	1626	1725	1542	935
Cdc42ep2	775	1126	739	886
Cdc42ep3	498	515	793	634
Cdc42ep4	1725	2552	2281	2420
Cdc42ep5	288	365	217	344
Cdc42se1	4574	4795	4899	4288
Cdc42se2	269	127	177	88
Cdc45	271	372	235	132
Cdc5l	1895	1945	2084	2318
Cdc6	108	193	99	146
Cdc7	183	276	193	95
Cdc73	136	138	135	193
Cdca2	154	311	256	164
Cdca3	854	989	1115	1055
Cdca4	1476	1082	1303	997
Cdca5	12	33	9	6
Cdca7	412	574	396	240
Cdca7l	1286	626	999	341
Cdca8	305	754	471	336
Cdcp1	1727	1128	1804	798
Cdh1	11048	2544	7076	2773
Cdh11	183	467	357	974
Cdh13	3958	7667	4494	4131
Cdh15	179	900	147	119
Cdh16	12	35	6	0
Cdh17	18	21	12	84
Cdh19	14	20	8	22
Cdh2	370	301	289	612
Cdh22	5	9	11	0
Cdh23	89	36	99	11
Cdh24	116	117	102	84
Cdh26	99	54	29	4
Cdh3	3011	1600	3895	1347
Cdh4	37	70	8	37
Cdh5	832	2945	2409	2750
Cdhr1	60	209	98	38
Cdhr2	18	0	24	23
Cdhr3	4	0	0	23
Cdhr5	22	41	42	0
Cdip1	557	885	213	302
Cdipt	1941	2082	1843	2104
Cdk1	1370	2152	1792	1459
Cdk10	1279	1331	1287	1241
Cdk11b	886	1026	1008	983
Cdk12	672	615	602	549
Cdk13	1164	624	784	789
Cdk14	818	1450	1286	1842

Cdk16	2860	4238	2952	3400
Cdk17	395	295	487	211
Cdk18	58	52	44	50
Cdk19	740	781	1109	821
Cdk2	470	653	694	361
Cdk20	516	549	432	945
Cdk2ap1	8443	6116	5910	6359
Cdk2ap2	1049	1164	1076	995
Cdk4	7852	7540	8286	8194
Cdk5	551	801	617	629
Cdk5rap1	593	629	460	557
Cdk5rap2	628	870	706	437
Cdk5rap3	786	1246	877	984
Cdk6	164	245	132	129
Cdk7	779	707	879	996
Cdk8	592	538	584	490
Cdk9	2558	2415	2702	2578
Cdkal1	542	382	339	466
Cdkl2	634	318	540	312
Cdkl4	16	6	11	0
Cdkn1a	10629	17017	12422	6674
Cdkn1b	1192	522	1007	989
Cdkn1c	1073	1576	1124	1486
Cdkn2aip	247	242	424	329
Cdkn2aipnl	1394	1756	1244	2126
Cdkn2b	2128	473	2711	573
Cdkn2c	307	417	331	312
Cdkn3	1076	1564	1145	679
Cdnf	94	121	51	91
Cdo1	1046	204	746	136
Cdon	694	1716	1431	1282
Cdpf1	609	497	369	573
Cdr2	766	1042	969	1120
Cdr2l	508	1239	806	1026
Cds1	1397	669	879	760
Cds2	17	64	33	6
Cdsn	2029	835	894	511
Cdt1	183	285	108	66
Cdv3	2704	2529	2866	2177
Cdyl	421	560	575	560
Cdyl2	229	378	224	392
Ceacam1	240	263	296	126
Ceacam16	4	1	3	4
Ceacam18	14	7	17	4
Ceacam19	82	18	32	0
Cebpa	2849	1794	2406	4187
Cebpb	61	81	15	19

Cebpd	508	195	387	253
Cebpe	0	16	3	6
Cebpg	2754	2153	2714	3153
Cebpz	1685	1561	1867	1822
Cecr2	233	231	235	115
Cecr5	480	493	396	430
Cecr6	11	2	14	0
Cel	14	0	11	0
Celf1	2775	1671	2410	2516
Celf2	427	898	611	764
Celf3	2	40	8	0
Celf4	35	14	21	13
Celf5	6	13	15	15
Celsr1	1881	864	1491	473
Celsr2	2168	901	2004	592
Celsr3	27	35	39	0
Cemip	151	399	289	326
Cenpa	29	176	41	59
Cenpb	2281	3319	2197	3006
Cenpc	137	262	280	221
Cenpe	224	574	403	451
Cenpf	566	813	858	555
Cenph	188	263	193	251
Cenpi	69	105	98	114
Cenpj	400	366	486	208
Cenpk	246	190	83	95
Cenpl	337	415	426	403
Cenpm	140	151	158	55
Cenpn	490	412	604	604
Cenpo	427	640	526	364
Cenpq	54	120	113	98
Cenpt	216	295	232	207
Cenpu	174	257	248	274
Cenpv	329	267	254	263
Cenpw	209	296	241	348
Cep104	307	370	328	441
Cep112	38	38	54	118
Cep120	993	733	838	888
Cep126	28	26	38	34
Cep128	365	489	346	336
Cep131	177	189	95	132
Cep135	101	141	74	81
Cep152	158	79	188	168
Cep162	228	176	217	81
Cep164	883	767	642	483
Cep170	520	598	545	646
Cep170b	666	825	785	560

Cep19	1833	2039	2759	1611
Cep250	318	501	390	283
Cep290	119	119	139	97
Cep295	268	295	347	343
Cep295nl	106	0	48	98
Cep350	126	185	220	167
Cep41	153	169	132	154
Cep44	285	340	435	251
Cep55	66	205	125	241
Cep57	90	45	85	71
Cep57l1	295	257	450	562
Cep63	791	512	635	595
Cep68	671	1240	779	1184
Cep70	156	75	60	45
Cep72	41	16	42	14
Cep76	224	254	311	273
Cep78	256	302	384	187
Cep83	676	470	587	500
Cep85	611	524	555	636
Cep85l	32	75	68	41
Cep89	197	423	387	278
Cep95	393	413	506	287
Cep97	146	98	149	52
Cept1	1436	1022	1136	1458
Cercam	1520	3886	1903	5024
Cerk	1571	2229	1983	3237
Cerkl	268	175	182	783
Cers1	229	140	179	137
Cers2	984	1585	1323	3474
Cers3	262	154	134	154
Cers4	575	284	296	219
Cers5	2989	3386	2616	3419
Cers6	35	37	21	52
Ces1a	53	32	30	29
Ces1c	0	18	6	11
Ces1d	6573	1482	2477	8152
Ces2g	625	296	299	195
Ces2h	96	24	48	58
Ces4a	24	20	24	27
Ces5a	3	0	0	32
Cetn2	1800	1048	1493	1556
Cetn4	36	68	59	30
Cfap100	9	12	32	2
Cfap126	12	74	8	50
Cfap157	45	59	0	28
Cfap20	1328	1485	1294	1386
Cfap36	1619	1506	2147	1908

Cfap43	176	46	62	67
Cfap44	78	49	48	514
Cfap45	7	35	12	47
Cfap52	28	21	11	8
Cfap53	1326	941	1134	1101
Cfap57	36	8	6	41
Cfap69	135	97	125	56
Cfap70	386	219	214	376
Cfap74	14	20	27	8
Cfap97	227	182	194	128
Cfap99	10	38	29	60
Cfb	1635	3023	3688	1090
Cfd	6302	3482	6095	1285
Cfdp1	2512	2222	2786	2942
Cfh	1199	2064	1741	2940
Cfi	474	344	500	124
Cfl1	26027	31231	27609	35846
Cfl2	5356	6570	4518	5464
Cflar	1248	952	1058	1075
Cfp	573	1089	772	685
Cggbp1	2294	1133	2061	2166
Cgn	229	22	143	35
Cgnl1	376	626	375	350
Cgref1	1058	1916	785	2137
Cgrrf1	935	1031	740	1020
Ch25h	172	98	156	66
Chac1	483	157	363	142
Chac2	77	104	105	116
Chad	29	0	26	15
Chadl	30	44	48	63
Chaf1a	305	310	182	393
Chaf1b	106	261	159	54
Champ1	470	578	593	539
Chat	6	12	2	0
Chchd1	2552	1979	1652	2543
Chchd3	4867	3832	2911	4133
Chchd4	306	385	199	303
Chchd5	751	792	707	551
Chchd6	403	669	504	584
Chchd7	826	632	969	812
Chd1	1165	772	1073	912
Chd1l	519	515	567	402
Chd2	1193	979	1356	1013
Chd3	1224	1641	1383	2083
Chd4	3950	5302	4381	3974
Chd5	16	0	2	19
Chd6	517	449	709	497

Chd7	134	115	134	143
Chd8	1260	1562	1426	1232
Chd9	677	543	679	580
Chdh	606	242	298	178
Chek1	211	264	241	189
Chek2	255	242	209	162
Cherp	286	649	325	392
Chfr	754	904	820	862
Chga	30	11	9	62
Chi3l1	1109	996	1335	652
Chic1	79	52	65	64
Chic2	930	579	758	727
Chit1	46	92	5	6
Chka	321	311	340	404
Chkb	1100	989	940	1031
Chl1	278	120	190	90
Chm	85	95	98	107
Chml	1	3	17	0
Chmp1a	2754	2572	2550	2707
Chmp1b	1387	1099	1304	1461
Chmp2a	9827	9441	9420	8523
Chmp2b	2000	1802	2192	2058
Chmp3	5653	5251	6167	6151
Chmp4b	617	631	864	1065
Chmp4bl1	2994	3653	3367	4226
Chmp4c	519	259	379	252
Chmp5	2169	1399	1351	1609
Chmp6	925	1208	793	1220
Chmp7	1210	1355	1286	1418
Chn1	188	450	296	278
Chn2	99	81	161	114
Chn3	3	29	24	12
Chodl	201	135	47	87
Chordc1	910	580	578	816
Chp1	1091	1428	1326	1182
Chp2	231	369	469	420
Chpf	1361	2225	1997	2008
Chpf2	49	291	95	188
Chpt1	1239	857	1080	1445
Chrac1	1014	948	642	670
Chrd	176	592	382	308
Chrdl1	63	124	56	16
Chrdl2	99	128	187	23
Chrm3	20	0	54	23
Chrna1	860	2190	3367	678
Chrnb1	864	1232	1023	644
Chrnb2	315	221	271	257

Chrnd	721	3111	3008	492
Chrne	118	338	185	79
Chrng	73	1144	530	133
Chst1	92	571	244	271
Chst10	41	153	138	215
Chst11	754	1150	1029	1454
Chst12	802	1117	1052	1803
Chst13	35	20	6	10
Chst14	301	833	572	845
Chst15	614	401	727	520
Chst2	454	390	591	572
Chst3	168	322	131	166
Chst5	29	19	39	0
Chst7	6	58	17	32
Chst8	8	14	41	5
Chsy1	402	807	749	1110
Chsy3	84	146	89	188
Chtf18	65	145	90	67
Chtf8	1759	1973	2152	1895
Chtop	85	111	80	125
Chuk	319	319	453	311
Churc1	3413	1727	2021	2397
Ciao1	700	928	776	972
Ciapin1	1638	1280	1360	1333
Cib1	2111	2013	1831	2119
Cib2	2167	574	1541	581
Cic	2228	1930	2308	1917
Cidea	755	461	460	788
Cidec	469	59	286	44
Ciita	740	571	564	509
Cilp	3767	2040	2693	1516
Cinp	361	502	789	722
Cipc	1033	765	683	1027
Cir1	659	862	1130	957
Cirbp	1549	1081	1535	1006
Cisd1	6066	6286	5257	4994
Cisd2	1917	1827	1786	1938
Cisd3	799	971	567	636
Cish	474	170	336	150
Cit	13	91	38	77
Cited1	69	80	62	20
Cited2	1092	618	828	925
Cited4	10	23	20	17
Ciz1	546	859	716	681
Ckap2	258	329	363	284
Ckap2l	289	333	322	405
Ckap4	4960	8207	6692	11227

Ckap5	1152	1526	1264	1223
Ckb	1629	2877	1893	4027
Cklf	348	254	224	353
Ckm	43108	27380	7278	33506
Ckmt1b	6118	2719	4506	1390
Ckmt2	1226	2395	621	1680
Cks1b	626	747	648	442
Cks2	1110	1128	682	736
Clasp1	19	101	63	25
Clasp2	1279	1206	1318	1165
Clasrp	240	404	232	197
Clca2	4649	491	1163	645
Clca4	19	24	8	0
Clca5	1571	384	1118	206
Clcc1	1188	744	1016	981
Clcf1	239	379	259	110
Clcn1	332	167	169	164
Clcn3	689	296	442	402
Clcn4	252	183	134	370
Clcn5	4	13	24	78
Clcn6	320	531	453	638
Clcn7	1119	1632	1232	2125
Clcnkb	287	424	302	86
Cldn1	4778	1298	3000	1859
Cldn10	89	18	188	1
Cldn11	23	0	6	0
Cldn12	469	339	390	613
Cldn15	77	141	77	95
Cldn19	9	0	23	25
Cldn2	194	180	400	52
Cldn22	30	0	57	6
Cldn23	151	129	125	129
Cldn3	101	57	21	35
Cldn4	2536	773	1700	797
Cldn5	557	582	730	322
Cldn6	4	21	9	0
Cldn7	60	40	5	22
Cldn8	15	0	32	31
Cldn9	34	8	15	27
Cldnd1	1848	1435	1616	1701
Clec10a	3204	6917	5099	4252
Clec11a	1920	1109	2966	3269
Clec12a	193	73	143	429
Clec12b	11	15	2	6
Clec14a	298	485	618	554
Clec16a	781	951	820	1020
Clec2d	805	989	870	865

Clec2d2	114	52	107	150
Clec2g	12112	8076	10400	12190
Clec2l	86	57	39	60
Clec3b	1028	4517	818	633
Clec4a	278	153	400	853
Clec4a1	3072	2654	4092	7147
Clec4a2	58	137	123	241
Clec4a3	1901	1899	2073	4819
Clec4d	275	579	343	1084
Clec4e	1313	1313	961	1988
Clec4f	7	0	2	0
Clec5a	95	188	281	806
Clec7a	663	325	1070	3421
Clec9a	128	88	227	418
Clic1	8602	12012	9423	12061
Clic2	800	1057	1127	1106
Clic3	185	52	95	66
Clic4	11827	9322	9990	9608
Clic5	3113	1160	802	1735
Clint1	418	773	653	680
Clip1	2079	1601	1842	1821
Clip2	468	745	645	927
Clip4	839	712	865	692
Clk1	3029	1850	2913	2212
Clk2	1362	1176	1189	1041
Clk3	1314	1285	1387	1267
Clk4	768	422	715	475
Clmn	36	4	23	4
Clmp	540	708	864	1291
Cln3	436	491	394	706
Cln5	817	929	943	1589
Cln6	1341	1339	1542	1762
Cln8	751	569	752	1375
Clnk	49	12	30	25
Clns1a	2311	2096	1559	2171
Clock	742	578	670	803
Clp1	369	344	435	440
Clpb	676	895	821	668
Clpp	808	860	641	593
Clptm1	2155	2667	1682	2256
Clptm1l	3712	4012	3606	4149
Clpx	943	818	1035	875
Clrn1	4	0	2	0
Clspn	7	55	18	7
Clstn1	3365	4243	3952	2529
Clstn2	35	20	20	42
Clstn3	454	373	263	288

Clta	7795	6801	6918	9173
Cltb	4483	4077	3461	2697
Cltc	1845	675	2632	1847
Clu	11029	8923	12679	4568
Cluap1	1228	1067	1140	1187
Cluh	3798	3342	3342	2699
Clybl	234	260	196	306
Cma1	719	630	558	190
Cmah	18	15	53	18
Cmas	1597	1232	1258	1958
Cmbl	333	163	129	202
Cmc1	1032	571	742	694
Cmc2	1510	1473	1493	1056
Cmip	2993	2435	2962	3251
Cmklr1	482	851	838	946
Cmpk1	2288	2015	1947	2962
Cmpk2	130	166	84	176
Cmss1	1176	561	644	484
Cmtm3	1805	2394	2574	2926
Cmtm4	30	0	33	0
Cmtm5	33	0	54	25
Cmtm6	2106	3053	4171	4733
Cmtm7	1300	1897	1860	3109
Cmtm8	376	327	357	184
Cmtr1	1530	1639	1461	1486
Cmtr2	101	114	164	119
Cmya5	8860	6053	4029	7222
Cnbd2	49	108	102	111
Cnbp	17157	16208	15317	18194
Cndp1	174	481	348	518
Cndp2	3874	6313	5627	8101
Cnep1r1	667	390	814	879
Cnfn	1624	1203	1260	1018
Cnga4	15	10	15	0
Cngb1	9	5	5	27
Cnih1	3192	3241	3614	5328
Cnih2	7	35	18	83
Cnih4	2954	2081	1835	2468
Cnksr1	752	656	490	291
Cnksr2	193	99	104	133
Cnksr3	332	525	363	570
Cnn1	180	433	187	170
Cnn2	137	587	369	439
Cnn3	3885	5970	4479	6683
Cnnm1	47	51	33	25
Cnnm2	60	85	77	122
Cnnm3	827	584	758	775

Cnnm4	329	314	360	307
Cnot1	3188	2787	2920	2424
Cnot10	751	784	704	597
Cnot11	986	802	1112	1432
Cnot2	2565	1704	2106	1881
Cnot3	507	748	583	734
Cnot4	551	472	623	570
Cnot6	2429	2026	2285	2463
Cnot6l	887	480	969	1020
Cnot7	1061	385	650	688
Cnot8	2377	2130	2562	2694
Cnot9	1764	1431	1808	1686
Cnp	1442	1608	1712	1526
Cnppd1	2706	2750	2671	3823
Cnpy2	3005	3629	3319	5037
Cnpy3	963	1385	1098	1554
Cnpy4	1420	2185	2388	2282
Cnr1	0	12	2	22
Cnr2	168	163	266	344
Cnrip1	472	676	621	803
Cnst	1577	931	948	837
Cntd1	34	15	14	28
Cntf	125	88	138	176
Cntfr	470	414	575	352
Cntln	195	161	169	129
Cntn1	767	280	645	418
Cntn2	92	74	93	70
Cntn3	0	23	0	18
Cntnap1	38	55	39	11
Cntrl	318	396	427	236
Cntrob	48	161	170	86
Coa3	2690	2273	1899	2473
Coa4	624	603	530	643
Coa5	4140	2843	3224	3809
Coa6	0	4	13	0
Coa7	434	610	328	456
Coasy	710	827	621	680
Cobl	970	1361	579	805
Cobll1	365	162	235	106
Coch	720	558	1991	218
Cog1	1035	921	1250	743
Cog2	408	753	339	481
Cog3	587	684	803	893
Cog4	1874	1738	1447	1714
Cog5	363	392	302	344
Cog6	667	638	665	867
Cog7	557	923	758	688

Cog8	1528	1859	1409	1698
Coil	304	326	208	273
Col11a1	459	573	900	2139
Col11a2	23	26	18	3
Col12a1	5894	12635	12157	31564
Col13a1	89	166	50	14
Col14a1	6072	8643	6265	10897
Col16a1	2688	3374	4065	6819
Col17a1	23260	18311	19642	7997
Col18a1	7258	13819	10186	7999
Col19a1	166	163	478	53
Col22a1	96	124	62	35
Col24a1	81	142	93	244
Col26a1	25	335	29	217
Col27a1	711	1320	925	1461
Col28a1	30	77	39	66
Col2a1	93	0	27	12
Col4a1	11406	29147	22435	19620
Col4a2	6549	8490	10834	8375
Col4a3bp	1340	1756	1535	2003
Col4a4	19	0	8	0
Col4a5	206	215	241	123
Col4a6	12	0	11	0
Col5a1	9643	27939	17430	40040
Col5a2	13881	24937	18208	40598
Col5a3	5923	22150	13818	16257
Col6a1	25872	43330	32079	56646
Col6a2	23377	50641	35025	59840
Col6a3	22248	37764	31724	64827
Col6a5	2824	90	2269	1433
Col6a6	151	310	149	140
Col7a1	3196	2970	2544	2282
Col8a1	183	381	278	228
Col8a2	961	665	1092	879
Colca2	5	14	23	0
Colec12	761	1039	852	1729
Colgalt1	2727	3879	3110	4936
Colgalt2	53	119	47	51
Colq	43	74	15	18
Commd1	1973	2101	2209	1981
Commd10	1307	874	993	967
Commd2	951	1015	823	789
Commd3	3169	2352	2935	3213
Commd4	1534	1732	1499	1388
Commd6	1164	1182	1005	1360
Commd7	1460	1196	1118	1226
Commd8	1864	1424	1992	1822

Commd9	1362	1730	1313	1955
Comp	12	55	20	0
Comt	4934	4306	3297	5381
Comtd1	771	383	657	437
Copa	4256	4391	4470	6276
Copb1	2522	3018	3014	3757
Copb2	2164	2665	2514	2927
Cope	2290	3710	2069	2633
Copg1	3495	4855	4183	4911
Copg2	2294	2237	2647	1968
Coprs	751	776	713	716
Cops2	2452	1951	2118	2544
Cops3	2448	2444	1875	2616
Cops4	1056	1337	1526	1467
Cops5	4667	4430	4920	4304
Cops6	4147	4049	3403	4157
Cops7a	3014	3096	2385	2464
Cops7b	465	571	483	521
Cops8	1498	1736	1246	1604
Cops9	6033	5782	5478	5822
Copz1	7329	6397	6634	7419
Copz2	2466	3481	3716	4818
Coq10a	3066	1853	1431	3291
Coq10b	1070	514	670	654
Coq2	1110	680	537	655
Coq3	1163	907	847	1006
Coq4	371	381	295	187
Coq5	870	768	611	1140
Coq6	1547	1655	1127	1510
Coq7	1245	864	972	1013
Coq9	2682	2471	1506	3051
Corin	30	27	51	0
Coro1a	2408	1860	2177	3605
Coro1b	4167	4567	4244	4976
Coro1c	3331	5293	3989	5387
Coro2a	97	106	107	81
Coro2b	207	691	316	214
Coro6	1949	2566	2536	1675
Coro7	543	738	594	894
Cotl1	4307	8188	7554	12807
Cox10	668	497	500	552
Cox11	173	45	78	7
Cox14	1997	1868	1289	1418
Cox15	3	19	29	13
Cox16	1567	860	1095	1177
Cox17	1779	1551	1840	1512
Cox18	248	189	156	99

Cox19	1001	1053	790	960
Cox20	2798	1311	2405	1879
Cox4i1	15775	16550	12101	13711
Cox4i2	583	876	635	507
Cox5a	2385	1126	3346	2839
Cox5b	5309	4809	5648	4918
Cox6a1	9948	10640	9501	9457
Cox6a2	9250	7646	3796	4722
Cox6b2	173	244	141	96
Cox6c	14927	8953	10807	12755
Cox7a1	5	0	0	18
Cox7a2	160	249	185	170
Cox7a2l	8547	6944	6518	7135
Cox7a2l2	8760	6999	4909	5775
Cox7b	11695	7068	8787	8699
Cox7c	5954	6338	5237	5444
Cox8a	6741	6368	3889	4021
Cox8b	6018	6073	2728	3555
Cp	1341	1459	2001	2069
Cpa1	119	529	152	78
Cpa2	31	37	56	106
Cpa3	439	273	260	52
Cpa4	2805	1166	584	849
Cpd	1824	2055	2228	2547
Cpe	4575	2612	6060	4082
Cpeb1	36	128	57	15
Cpeb2	264	249	463	274
Cpeb3	181	124	242	210
Cpeb4	230	254	348	264
Cped1	333	325	441	473
Cpm	980	275	1336	259
Cpne2	272	461	214	373
Cpne3	1221	1097	939	1575
Cpne5	70	67	90	55
Cpne7	197	211	215	106
Cpne8	763	635	612	692
Cpne9	613	578	575	657
Cpox	455	554	692	433
Cpped1	340	360	265	626
Cpq	1734	3255	2206	3721
Cpsf1	907	1128	731	704
Cpsf2	1335	1059	1294	1279
Cpsf3	1288	1450	1432	1476
Cpsf3l	830	990	819	737
Cpsf4	173	223	138	228
Cpsf6	1128	872	1071	1067
Cpsf7	1708	1507	2025	1707

Cpt1a	1528	1312	1777	1482
Cpt1b	1804	1842	1748	1246
Cpt2	1118	985	1020	820
Cptp	733	500	630	567
Cpvl	170	68	102	57
Cpxm1	9033	4538	6542	8496
Cpxm2	2715	1320	2480	1430
Cpz	420	812	691	2662
Cr1l	2379	2469	3191	3484
Crabp1	2810	1551	1948	3195
Crabp2	8139	3919	6432	2742
Cracr2a	44	57	27	17
Cradd	20	45	30	27
Cramp1	360	477	489	418
Crat	2793	3282	2391	3118
Crb1	0	6	0	20
Crb3	238	127	193	105
Crbn	979	588	707	1093
Crcp	809	850	581	911
Creb1	292	183	275	241
Creb3	2178	2941	2738	2816
Creb3l1	1011	3059	1914	5241
Creb3l2	65	133	71	86
Creb3l4	465	79	430	149
Creb5	686	421	654	385
Crebbp	1497	825	1265	1362
Crebl2	176	314	326	201
Crebrf	474	505	471	617
Crebzf	594	460	487	401
Creg1	447	300	370	664
Creld1	1157	1799	1367	1192
Creld2	1507	1829	1581	1452
Crem	1195	1066	952	756
Crhr1	8	33	27	4
Crhr2	195	249	238	132
Crim1	341	603	664	274
Crip1	5575	19312	7344	14067
Crip2	5426	6628	5299	3376
Crip3	26	0	12	16
Cript	1774	1019	1229	1645
Crispld1	32	13	17	0
Crispld2	2125	5765	3716	5147
Crk	2521	2610	3029	2497
Crlf1	191	547	211	250
Crlf2	260	256	188	297
Crlf3	697	673	618	768
Crls1	772	632	875	576

Crnde	279	271	241	161
Crnkl1	213	204	314	285
Crnn	40	0	175	0
Crocc	54	83	50	33
Crocc2	59	36	66	37
Crot	1972	1132	1137	1698
Crtac1	2	0	3	0
Crtap	2433	3535	3441	4881
Crtc1	313	401	307	401
Crtc2	1445	1582	1855	1455
Crtc3	0	5	5	20
Cry1	71	122	78	51
Cry2	350	664	556	485
Cryaa	18	15	6	31
Cryab	26018	27815	22677	18559
Cryba4	35	86	48	8
Crybb1	14	14	0	12
Crybg3	315	512	511	481
Cryga	13	11	5	0
Cryl1	542	626	402	537
Crym	288	420	203	240
Cryz	151	196	227	272
Cryzl1	1331	608	370	530
Cs	11038	9642	8046	10197
Csad	774	678	731	546
Csdc2	129	72	134	63
Csde1	7237	3760	4302	5213
Cse1l	1785	1711	1435	1641
Csf1	1043	1565	1265	2198
Csf1r	3112	6057	3533	7296
Csf2ra	254	579	487	577
Csf2rb	34	372	122	204
Csf3	114	212	233	43
Csf3r	141	229	182	369
Csgalnact1	341	587	462	521
Csgalnact2	572	616	531	497
Csk	714	1198	1174	1484
Csnk1a1	9789	6294	8496	6040
Csnk1d	4318	3762	3946	3668
Csnk1e	1731	1262	2307	1518
Csnk1g1	73	127	140	103
Csnk1g2	1056	1620	1097	1148
Csnk1g3	857	857	782	918
Csnk2a1	2837	2613	2795	3022
Csnk2a2	2004	2443	2194	2465
Csnk2b	2963	4152	3384	3420
Cspg4	1422	2276	1681	1061

Cspg5	16	38	56	64
Cspp1	329	253	352	214
Csrnp1	722	726	770	530
Csrnp2	201	290	242	229
Csrp1	14277	21321	18478	18503
Csrp2	732	976	987	2532
Csrp3	24387	26488	13213	7616
Cst3	29195	18476	23691	34509
Cst6	18042	2494	9438	3041
Cst7	1313	973	1216	2197
Csta	1613	325	372	130
Cstb	23733	10592	21293	18221
Cstf1	649	796	677	682
Cstf2	537	415	402	396
Cstf2t	94	282	78	107
Cstf3	433	335	424	373
Ctbp1	3500	3765	2780	3602
Ctbp2	1025	1161	774	1361
Ctbs	658	1339	921	1616
Ctc1	905	829	876	889
Ctcf	1158	827	1076	1139
Ctdnep1	4311	4374	3634	4544
Ctdp1	503	885	743	732
Ctdsp1	4590	4603	4694	5020
Ctdsp2	4431	5452	6098	5692
Ctdspl	1580	1259	1244	900
Ctdspl2	12	71	42	34
Ctf1	368	198	176	225
Ctgf	8052	6755	9008	8518
Cth	237	201	351	63
Cthrc1	2076	4537	3385	9152
Ctif	4	0	3	0
Ctla2a	784	942	960	793
Ctnna1	4606	4268	4177	3504
Ctnna3	51	20	26	50
Ctnnal1	2416	1536	2392	1329
Ctnnb1	14503	7102	13233	8949
Ctnnbip1	1490	1051	1393	869
Ctnnbl1	682	1136	847	945
Ctnnd1	4486	4269	5469	4384
Ctnnd2	307	90	364	53
Ctns	467	241	450	476
Ctps1	2706	2597	2254	2199
Ctps2	1354	938	1526	1465
Ctr9	1143	1126	1184	1366
Ctrc	86	88	50	33
Ctrl	10	13	5	0

Ctsa	11755	19878	15668	32732
Ctsb	74758	78262	109480	172400
Ctsc	7563	7223	9480	9668
Ctsd	13351	22790	17724	39980
Ctse	398	319	269	641
Ctsf	1897	2822	2675	4602
Ctsh	11216	12967	12682	17974
Ctsk	5542	13221	12000	37361
Ctsl	16378	35947	36513	63086
Ctso	394	291	427	512
Ctss	6519	8083	8572	18945
Ctsw	346	59	149	129
Ctsz	4133	6788	6111	13911
Cttnbp2	180	28	156	40
Cttnbp2nl	1404	1257	1464	1906
Ctu1	558	349	447	515
Ctu2	661	910	701	564
Ctxn1	33	58	38	93
Ctxn3	103	573	179	192
Cubn	0	50	33	71
Cuedc1	651	1131	552	828
Cuedc2	1245	1329	1143	1572
Cul1	4345	3627	4615	4858
Cul2	1105	1013	688	1216
Cul3	4106	2980	3137	3815
Cul4a	1494	2074	1861	1621
Cul4b	1539	885	996	1455
Cul5	1549	1253	1398	1440
Cul9	96	200	161	123
Cuta	2156	1833	1681	1232
Cutc	1035	486	763	836
Cux1	2307	2679	2361	3326
Cwc15	2142	1995	1969	2642
Cwc22	573	452	722	641
Cwc25	319	258	384	203
Cwc27	566	683	895	754
Cwf19l1	681	694	611	558
Cwf19l2	413	291	394	236
Cwh43	585	304	316	235
Cx3cl1	119	21	94	71
Cx3cr1	449	348	373	849
Cxadr	1881	713	781	734
Cxcl1	597	843	903	288
Cxcl10	965	145	343	191
Cxcl12	7401	4420	9137	5873
Cxcl13	939	487	1061	3284
Cxcl14	8275	7899	7881	2837

Cxcl16	7661	2988	6085	6373
Cxcl17	4	0	3	7
Cxcl2	5394	9035	3655	797
Cxcl6	206	41	211	669
Cxcl9	4580	1252	2559	2439
Cxcr2	188	117	98	110
Cxcr3	122	41	74	213
Cxcr4	1259	1625	1780	2093
Cxcr5	65	140	111	48
Cxcr6	45	31	30	0
CXHXorf65	1553	1781	1990	3726
Cxxc1	1189	1157	1059	937
Cxxc4	15	13	14	30
Cxxc5	529	805	664	657
Cyb561	906	720	742	541
Cyb561a3	850	787	909	1131
Cyb561d1	753	518	530	592
Cyb561d2	458	528	475	667
Cyb5a	4376	3777	4587	5242
Cyb5b	4085	2332	2412	3037
Cyb5d1	543	443	387	349
Cyb5d2	113	83	102	78
Cyb5r1	3144	2979	3053	2425
Cyb5r2	81	56	90	58
Cyb5r3	5718	9123	6400	6848
Cyb5r4	1192	1154	1026	1538
Cyb5rl	16	2	12	24
Cyba	1978	2835	2328	4140
Cybb	3128	5587	9334	28317
Cybrd1	284	402	644	621
Cyc1	8624	8049	5165	6399
Cycs	719	324	105	263
Cyfip1	1123	2028	2075	1394
Cyfip2	192	165	241	726
Cygb	825	1461	1453	952
Cyhr1	288	101	199	185
Cyld	641	307	426	417
Cyld-ps1	37	56	96	108
Cyp11a1	16	2	14	8
Cyp17a1	575	418	193	548
Cyp1a1	19	13	11	0
Cyp1b1	633	369	355	457
Cyp20a1	922	1883	1183	1818
Cyp26b1	947	695	545	1278
Cyp26c1	255	590	478	456
Cyp27a1	677	599	591	504
Cyp27b1	23	88	63	22

Cyp2ab1	106	226	35	56
Cyp2b1	45	14	14	12
Cyp2b12	12073	1800	4052	8304
Cyp2b21	484	160	185	281
Cyp2c11	21	0	0	11
Cyp2d1	185	262	208	256
Cyp2e1	18	14	5	31
Cyp2f4	57	12	15	72
Cyp2j10	401	169	187	189
Cyp2j4	447	260	310	470
Cyp2r1	60	8	33	53
Cyp2s1	607	761	639	433
Cyp2t1	596	256	399	685
Cyp2u1	322	374	378	499
Cyp39a1	96	41	105	34
Cyp3a62	6	11	9	0
Cyp3a9	102	74	77	36
Cyp46a1	391	1178	641	1074
Cyp4b1	320	476	653	114
Cyp4f1	221	147	99	114
Cyp4f17	27	65	26	2
Cyp4f18	155	116	62	98
Cyp4f37	0	14	15	0
Cyp4f39	416	299	221	195
Cyp51	8180	4566	5445	6898
Cyp7b1	631	510	718	1165
Cyr61	1758	3718	2422	1999
Cys1	73	92	89	107
Cysltr1	182	201	345	236
Cysrt1	279	102	77	57
Cystm1	917	1014	620	942
Cyth1	371	818	406	450
Cyth2	2208	2472	2481	2210
Cyth3	933	1703	834	937
Cyth4	1106	1407	1614	2914
Cytip	341	186	459	1024
Cytl1	437	332	266	233
Cyyr1	257	378	366	282
D2hgdh	233	217	209	255
Da2-19	32	3	44	39
Daam1	1108	645	1053	1116
Daam2	239	479	348	598
Dab1	8	28	42	20
Dab2	2529	3727	3722	6636
Dab2ip	994	1664	1112	939
Dach1	90	28	60	80
Dact1	137	189	328	571

Dact2	591	230	295	115
Dact3	552	536	712	975
Dad1	4701	5625	4577	5739
Dag1	5407	7632	5138	5419
Dagla	139	331	181	155
Daglb	433	696	521	1006
Dalrd3	324	570	419	357
Dancr	615	254	313	119
Dap	5093	7381	6983	8992
Dap3	781	959	793	770
Dapk1	658	530	1004	1451
Dapk2	1725	626	931	570
Dapk3	344	414	269	448
Dapl1	1408	189	1059	138
Dapp1	603	310	427	685
Dars	945	1208	1056	1107
Dars2	484	439	548	329
Daxx	796	1078	868	974
Dazap1	583	698	697	684
Dazap2	5725	6441	7527	8247
Dbf4	0	55	23	30
Dbi	15121	10127	8826	6999
Dbil5	102	46	108	35
Dbn1	982	2128	1661	1707
Dbndd2	1809	1527	1610	941
Dbnl	1908	2477	1708	2187
Dbp	3256	3512	2582	2795
Dbr1	676	702	823	749
Dbt	646	521	512	757
Dcaf1	980	799	1116	1070
Dcaf10	409	405	372	373
Dcaf11	3498	3024	3024	3326
Dcaf13	1590	1458	1625	1846
Dcaf15	293	482	277	298
Dcaf17	355	487	540	458
Dcaf4	507	620	412	264
Dcaf5	722	941	1035	1097
Dcaf6	3436	2576	2576	3217
Dcaf7	556	672	662	907
Dcaf8	368	887	1019	662
Dcakd	985	1366	1031	1013
Dcbld1	374	270	411	409
Dcbld2	1054	718	1162	1089
Dcc	14	0	12	0
Dchs1	212	684	483	819
Dck	408	373	298	260
Dclk1	2323	6414	4186	3203

Dclk2	42	91	92	25
Dclk3	77	139	126	133
Dclre1a	297	464	280	410
Dclre1b	496	420	651	618
Dclre1c	406	215	426	351
Dcn	19830	36234	30535	41999
Dcp1a	10	79	26	21
Dcp1b	205	201	197	246
Dcps	941	1008	867	872
Dcst2	1246	902	873	827
Dcstamp	41	41	68	235
Dct	551	572	116	364
Dctd	229	558	360	460
Dctn1	1989	2219	1735	1943
Dctn2	7585	8412	7466	7298
Dctn3	1280	1158	1094	837
Dctn4	1553	809	1183	1340
Dctn5	1796	2052	2042	2178
Dctn6	1258	1821	1404	1341
Dctpp1	185	527	132	309
Dcun1d1	740	571	668	707
Dcun1d3	149	143	122	105
Dcun1d4	490	246	444	533
Dcun1d5	2515	1631	1511	1763
Dcx	21	151	56	106
Dcxr	358	331	275	472
Dda1	1321	1345	1086	1039
Ddah1	2756	1744	3045	2031
Ddb1	6734	10593	8737	10339
Ddb2	959	836	1165	1374
Ddc	34	81	51	35
Ddhd1	830	488	407	644
Ddhd2	869	561	700	573
Ddi2	2065	1776	1902	1947
Ddias	12	23	26	52
Ddit3	825	914	533	811
Ddit4	2006	1174	1620	956
Ddit4l	577	249	187	312
Ddn	18	105	0	12
Ddo	23	2	17	2
Ddost	10096	11088	10227	13111
Ddr1	2196	1673	2167	1300
Ddr2	1432	2203	2013	2593
Ddrgk1	1601	1742	1614	1459
Ddt	1742	2008	1742	1738
Ddx1	3901	3851	3584	3711
Ddx10	528	667	533	601

Ddx11	225	224	238	203
Ddx17	7090	5871	6893	6197
Ddx18	1020	1200	653	723
Ddx19a	2507	2643	2779	3267
Ddx20	684	815	736	805
Ddx21	494	829	585	486
Ddx23	1266	1939	1363	1786
Ddx24	1222	1573	1291	1455
Ddx25	38	99	45	179
Ddx27	1110	1073	1288	1167
Ddx28	560	408	417	659
Ddx3	0	269	0	174
Ddx31	291	590	406	302
Ddx39a	2626	3314	2582	2733
Ddx39b	6797	7837	6703	5771
Ddx3x	6035	2508	3918	4352
Ddx41	367	615	355	465
Ddx42	1878	2006	2451	2151
Ddx46	1988	1507	1896	2049
Ddx47	1878	2652	2103	2208
Ddx49	807	1087	739	634
Ddx5	12710	11977	15745	14624
Ddx50	790	936	868	760
Ddx51	503	430	587	573
Ddx52	610	345	396	486
Ddx54	1358	1487	1256	1257
Ddx55	237	470	388	424
Ddx56	304	628	376	387
Ddx58	465	492	481	584
Ddx59	453	763	668	504
Ddx6	1349	1220	1118	1511
Ddx60	19	58	2	0
Deaf1	374	611	328	312
Decr1	1994	1696	2337	1769
Decr2	334	246	302	245
Dedd	300	475	555	433
Dedd2	287	290	202	261
Def6	38	101	51	43
Def8	1359	1088	1035	781
Defb1	1576	314	453	217
Defb14	367	161	78	66
Degs1	5125	4563	4157	5451
Degs2	224	164	89	39
Dek	3164	1325	1871	2414
Dennd1a	360	351	370	467
Dennd1b	685	558	510	679
Dennd1c	425	478	400	668

Dennd2a	165	440	320	436
Dennd2c	855	438	746	591
Dennd2d	409	182	265	273
Dennd3	354	458	469	319
Dennd4a	244	258	227	651
Dennd4b	1202	765	922	898
Dennd4c	111	129	138	130
Dennd5a	984	1461	1484	1897
Dennd5b	31	85	68	67
Dennd6a	1432	676	1110	958
Dennd6b	91	133	107	85
Denr	2271	2162	2522	1791
Depdc1	125	133	75	191
Depdc1b	97	181	137	110
Depdc5	445	340	357	307
Depdc7	327	369	310	364
Deptor	1984	1679	2136	1348
Dera	1845	1926	1911	2217
Derl1	3087	2453	2651	3868
Derl2	1187	1263	1417	1735
Des	47879	80997	37015	42628
Desi1	2750	1808	1840	1577
Desi2	1511	1507	1604	1938
Det1	265	226	256	185
Dexi	897	841	737	660
Dffa	2973	1596	1623	2188
Dffb	248	180	254	252
Dfna5	26	28	17	92
Dfnb59	0	15	12	29
Dgat1	688	1041	712	897
Dgat2	1505	1372	1083	1427
Dgat2l6	212	129	116	41
Dgcr14	527	521	615	723
Dgcr6	1034	1317	1148	1149
Dgcr8	1203	936	918	890
Dgka	2872	2005	2501	1209
Dgkb	8	9	0	10
Dgkd	650	729	912	862
Dgke	290	293	277	259
Dgkg	27	67	41	19
Dgkh	55	69	57	76
Dgki	0	28	42	84
Dgkq	21	146	23	31
Dgkz	1371	1903	1192	1744
Dguok	317	204	196	243
Dhcr24	18674	9758	11462	14359
Dhcr7	2799	1618	1840	1398

Dhdds	1861	2685	1887	2033
Dhdh	0	9	1	6
Dhfr	694	637	644	451
Dhh	84	64	185	37
Dhodh	431	526	373	426
Dhps	1446	1605	1034	1263
Dhrs1	1135	1306	1268	1086
Dhrs11	480	304	328	329
Dhrs13	679	516	557	670
Dhrs3	1299	1177	1374	1444
Dhrs4	653	746	727	686
Dhrs7	14	53	5	19
Dhrs7b	1565	1410	1372	1260
Dhrs7c	1650	1451	331	854
Dhrs9	1265	488	1256	946
Dhrsx	247	271	199	187
Dhtkd1	52	45	105	75
Dhx15	1713	1484	1599	1721
Dhx16	783	853	638	681
Dhx29	720	634	818	590
Dhx30	617	681	600	587
Dhx32	1015	782	718	809
Dhx33	439	572	475	468
Dhx34	103	134	122	151
Dhx35	167	216	126	129
Dhx36	1038	628	636	710
Dhx37	321	418	287	283
Dhx38	318	563	378	456
Dhx40	959	611	667	558
Dhx57	432	509	506	441
Dhx58	1056	1310	966	915
Dhx8	727	647	796	771
Dhx9	546	1044	1435	1100
Diablo	437	515	406	420
Diaph1	2554	3984	2495	3940
Diaph2	10	86	35	25
Diaph3	348	405	375	150
Dicer1	1019	1080	1106	867
Dido1	395	371	570	285
Diexf	437	481	487	505
Dimt1	1361	850	811	815
Dio2	707	579	492	228
Dio3	16	73	51	14
Dip2a	45	95	35	71
Dip2b	219	392	457	298
Dip2c	793	878	781	705
Diras2	168	119	226	84

Diras3	52	63	11	40
Dirc2	1115	1128	1056	1448
Dis3	494	352	373	403
Dis3l	1148	991	1038	736
Dis3l2	382	471	372	281
Disc1	61	94	53	97
Disp1	82	118	129	91
Disp2	129	96	153	260
Dixdc1	147	48	104	252
Dkc1	180	415	120	156
Dkk2	376	125	314	211
Dkk3	4382	3713	5546	5943
Dkk4	359	493	521	591
Dlat	770	793	757	860
Dlc1	978	943	1079	1196
Dld	2612	2044	1632	2369
Dlg1	1378	900	1226	1349
Dlg2	95	72	38	30
Dlg3	362	329	399	500
Dlg4	224	526	414	641
Dlg5	646	538	799	550
Dlgap1	142	100	98	72
Dlgap4	1846	3967	1963	1953
Dlgap5	253	445	307	257
Dlk1	108	28	3	38
Dlk2	137	205	65	59
Dll1	181	259	218	98
Dll4	141	408	325	213
Dlst	4936	5504	5092	6547
Dlx1	26	11	86	0
Dlx3	537	230	877	122
Dlx4	1	5	3	0
Dmap1	260	489	326	362
Dmbt1	1068	1662	2586	2101
Dmd	1129	674	879	741
Dmkn	325	593	146	92
Dmp1	50	56	27	32
Dmpk	4872	6667	3457	2770
Dmrt2	173	340	248	247
Dmrta2	4	7	0	27
Dmtf1	25	60	42	30
Dmtn	14	0	24	35
Dmwd	306	532	376	322
Dmxl1	284	247	302	547
Dmxl2	127	146	102	316
Dnaaf2	319	404	262	410
Dnaaf3	7	35	12	30

Dnaaf5	672	521	639	415
Dnah1	32	51	55	6
Dnah10	99	74	74	145
Dnah11	24	8	36	33
Dnah17	30	23	27	43
Dnah2	9	0	11	0
Dnaja1	4857	4097	5719	5010
Dnaja2	4434	3259	3436	4363
Dnaja3	1376	1677	1283	1398
Dnaja4	5493	2570	2896	3106
Dnajb1	5567	2615	4208	2748
Dnajb12	1033	1151	1362	1315
Dnajb13	107	18	146	7
Dnajb14	156	64	248	264
Dnajb2	2067	1679	1780	1785
Dnajb3	326	149	313	240
Dnajb4	1388	1317	1335	1357
Dnajb5	2132	2007	1339	2069
Dnajb6	2509	2071	2146	2336
Dnajb9	223	397	352	447
Dnajc1	1548	1267	1464	1708
Dnajc10	1860	2198	2034	2260
Dnajc11	930	1114	882	1125
Dnajc12	145	177	361	178
Dnajc13	712	1092	1062	1120
Dnajc14	1150	1094	1297	968
Dnajc15	2383	1700	1763	1831
Dnajc16	15	86	39	34
Dnajc17	257	348	254	250
Dnajc18	331	449	298	377
Dnajc19	3022	2536	2532	2829
Dnajc2	1433	1107	1554	1607
Dnajc21	705	480	611	702
Dnajc22	245	433	352	497
Dnajc24	324	318	448	346
Dnajc25	606	923	579	1100
Dnajc27	246	206	155	283
Dnajc28	127	98	92	52
Dnajc3	1576	1975	1760	2328
Dnajc30	1066	806	847	1056
Dnajc4	388	415	296	333
Dnajc5	3282	2956	3316	4673
Dnajc5b	6	10	3	0
Dnajc7	4478	4415	4708	4594
Dnajc8	2546	3200	2940	3553
Dnajc9	1984	1591	2168	2214
Dnal1	37	59	42	67

Dnal4	271	588	400	430
Dnali1	12	0	14	7
Dnase1	16	20	15	12
Dnase1l1	1827	1201	1222	1732
Dnase1l2	554	508	376	321
Dnase1l3	30	21	21	76
Dnase2	2254	3506	2209	4122
Dnase2b	45	36	17	10
Dner	13	16	9	43
Dnhd1	1017	919	778	683
Dnm1	1650	1621	1762	1250
Dnm1l	1528	1215	1079	1503
Dnm2	2087	2668	2300	2622
Dnm3	753	485	1315	1096
Dnmbp	403	321	358	283
Dnmt1	529	893	591	614
Dnmt3a	818	713	828	623
Dnmt3b	151	67	96	81
Dnpep	2822	2848	2743	2623
Dnph1	330	332	223	199
Dntt	15	7	11	6
Dnttip1	1048	1107	1216	1158
Dnttip2	1575	1369	1784	1463
Doc2a	71	56	5	38
Doc2b	265	402	179	83
Doc2g	134	48	41	44
Dock1	1006	1103	1271	1537
Dock10	379	361	522	814
Dock11	619	629	657	1121
Dock2	469	622	436	1107
Dock3	49	13	26	0
Dock4	25	80	48	16
Dock5	676	537	794	901
Dock6	676	1003	946	621
Dock7	662	829	823	1175
Dock8	413	625	597	634
Dock9	722	842	1058	665
Dohh	375	532	274	370
Dok1	272	387	582	658
Dok2	172	423	397	676
Dok3	206	417	361	396
Dok4	58	203	134	171
Dok5	107	100	17	107
Dok7	148	330	65	63
Dolk	987	1127	966	996
Dolpp1	421	425	341	396
Dopey1	584	865	650	658

Dopey2	708	565	617	827
Dot1l	906	880	767	796
Dpagt1	2199	2663	2610	2774
Dpcd	1634	1604	1587	1552
Dpep1	819	358	632	641
Dpep2	418	604	614	990
Dpf1	57	93	35	78
Dpf2	1527	1841	1557	2029
Dpf3	2	17	0	21
Dph1	1554	1391	1068	939
Dph2	208	165	178	219
Dph3	2655	3063	2114	3052
Dph5	316	383	354	478
Dph6	441	282	423	237
Dph7	430	506	525	328
Dpm1	1692	1214	1595	2009
Dpm2	971	1193	963	985
Dpm3	2	0	3	6
Dpp3	74	254	185	182
Dpp4	627	1400	484	142
Dpp6	7	16	23	40
Dpp7	5772	7592	6024	10037
Dpp8	3723	4066	3606	3656
Dpp9	398	830	537	680
Dppa4	13	0	39	0
Dpt	13429	30396	15440	20746
Dpy19l1	1241	1580	1819	2599
Dpy19l3	296	757	503	460
Dpy19l4	147	139	215	170
Dpy30	2079	1672	1968	2065
Dpyd	16	0	17	24
Dpysl2	2973	3363	3297	5756
Dpysl4	5	55	24	21
Dram1	662	664	879	1293
Dram2	699	330	489	1234
Drap1	3403	5301	4268	3931
Draxin	16	72	24	86
Drc3	69	55	51	78
Drg1	1529	1139	1431	1046
Drg2	1098	1447	1088	1185
Drosha	1725	1916	1890	1655
Drp2	18	35	92	101
Dsc1	13	0	68	3
Dsc2	729	108	319	377
Dsc3	7620	4708	7329	2463
Dscc1	311	243	475	284
Dscr3	1072	1232	1153	931

Dse	601	711	712	1517
Dsel	227	129	217	359
Dsg1	4278	1146	2628	655
Dsg2	1589	833	1643	769
Dsg3	6016	3908	4986	2567
Dsg4	2	14	5	0
Dsn1	257	316	197	289
Dsp	24554	11175	22454	7207
Dst	6863	5020	6372	3320
Dstn	16386	9955	12646	9455
Dstnl1	720	1423	1255	968
Dstyk	594	613	752	611
Dtd1	1213	782	868	810
Dtd2	246	337	248	302
Dthd1	220	157	277	294
Dtl	173	160	248	218
Dtna	663	1049	674	417
Dtnb	289	334	268	254
Dtnbp1	3106	1889	2036	2056
Dtwd1	154	268	259	190
Dtwd2	69	43	74	123
Dtx1	17	44	60	23
Dtx2	239	380	233	173
Dtx3	1571	1351	1559	1276
Dtx3l	7	29	17	35
Dtx4	1220	1871	1742	2949
Dtymk	1330	1584	1208	1097
Duox1	671	391	733	283
Duoxa1	655	540	564	321
Dupd1	201	89	101	86
Dus1l	1489	1602	1342	1128
Dus2	419	404	348	294
Dus3l	458	549	369	452
Dus4l	232	156	153	167
Dusp1	4455	3639	3609	2961
Dusp10	508	249	391	243
Dusp12	718	604	796	695
Dusp13	1	16	26	31
Dusp14	12	21	12	1
Dusp15	17	21	39	82
Dusp16	612	421	676	740
Dusp18	765	631	918	986
Dusp19	75	184	137	146
Dusp2	611	365	564	571
Dusp22	2521	1612	1975	1364
Dusp23	5	24	2	1
Dusp26	1933	521	614	680

Dusp27	973	1764	1455	876
Dusp28	280	194	158	228
Dusp3	2650	2127	3057	2867
Dusp5	770	261	483	225
Dusp6	2535	1697	2802	1790
Dusp7	1584	1333	808	1261
Dusp8	297	221	224	209
Dusp9	12	60	11	15
Dut	857	597	778	450
Duxbl1	137	81	113	92
Dvl1	1446	2006	1401	1140
Dvl2	283	378	272	203
Dvl3	612	837	712	955
Dxo	557	584	465	327
Dym	1211	1027	1055	1547
Dync1h1	203	438	330	85
Dync1i2	3137	3115	3080	3549
Dync1li1	1380	1322	1529	1273
Dync1li2	3078	2503	3442	2550
Dync2h1	161	175	202	237
Dync2li1	590	347	606	441
Dynll1	17917	10969	13722	9367
Dynll2	3694	2517	3033	3063
Dynlrb1	7014	6308	6208	7106
Dynlt1	1570	2058	1724	1399
Dynlt3	2127	1152	1541	1718
Dyrk1a	2154	1983	2049	2064
Dyrk1b	617	576	322	273
Dyrk2	1776	1132	1927	2302
Dyrk3	133	114	38	40
Dysf	1993	3961	2747	1528
Dzf17	393	238	346	437
Dzip1	1042	1259	1395	807
Dzip1l	380	418	745	373
Dzip3	213	111	206	162
E2f1	370	523	421	543
E2f2	474	427	384	298
E2f3	45	23	89	34
E2f4	2658	2130	2267	1779
E2f5	97	79	129	141
E2f6	1677	1670	1268	1732
E2f7	68	91	89	82
E2f8	20	54	15	13
Eaf1	1044	1192	1094	1042
Eapp	1730	1333	1661	1475
Ear11	69	304	256	129
Ears2l1	14	58	18	23

Ebag9	235	256	337	148
Ebf1	734	871	873	1040
Ebf2	25	70	45	47
Ebf3	264	411	337	247
Ebf4	83	77	54	117
Ebi3	159	143	81	182
Ebp	4423	2543	3048	3424
Ebpl	1037	972	1205	1277
Ecd	234	230	274	216
Ece1	1009	3092	1610	1729
Ece2	275	529	388	766
Ecel1	0	8	2	0
Ech1	3773	2210	2880	3078
Echdc1	1767	1060	1076	1789
Echdc2	538	351	287	301
Echdc3	171	105	84	127
Eci1	1514	1404	1122	1187
Eci2	3232	2427	2705	2544
Ecm1	3733	14037	4670	4551
Ecscr	525	1555	1143	939
Ecsit	1223	1267	972	1023
Ect2	184	62	150	38
Eda	43	175	203	298
Eda2r	586	554	748	549
Edar	20	12	20	20
Edaradd	5	0	8	4
Edc3	1027	791	1165	903
Edc4	516	758	530	539
Eddm3b	8	7	51	23
Edem1	2169	2904	3188	4305
Edem2	631	904	667	978
Edem3	388	524	372	598
Edf1	2819	3894	2464	2590
Edn1	290	154	545	220
Edn2	92	46	232	90
Ednra	717	565	847	841
Ednrb	1275	745	880	458
Edrf1	499	329	486	475
Eea1	145	179	202	227
Eed	1001	728	841	744
Eef1a1	4215	7930	9155	8335
Eef1a2	5477	5664	2594	4236
Eef1akmt1	1054	990	809	952
Eef1b2	17870	13992	16972	14317
Eef1d	8030	9549	7983	7520
Eef1e1	498	651	670	819
Eef1g	25657	24747	24291	24646

Eef2	15950	30972	18247	21231
Eef2k	1457	2297	1398	2161
Eef2kmt	133	151	114	171
Eefsec	388	768	448	556
Eepd1	400	296	274	562
Efcab1	0	23	8	0
Efcab10	74	57	38	15
Efcab11	13	3	17	15
Efcab14	1356	1803	1891	2402
Efcab2	15	32	35	0
Efcab6	11	54	45	0
Efcab7	100	147	135	82
Efcab8	0	17	9	0
Efcc1	28	13	47	41
Efemp1	1546	2566	1216	549
Efemp2	1943	3696	2708	4554
Efhc1	131	363	277	351
Efhd1	112	143	98	96
Efhd2	2495	3093	2650	4151
Efl1	720	1078	856	725
Efna1	3578	2684	3366	1757
Efna2	70	95	68	158
Efna3	2399	1040	2045	922
Efna4	1060	378	853	338
Efna5	2053	561	1596	1084
Efnb1	948	1125	1145	710
Efnb2	9	2	15	11
Efnb3	34	17	45	0
Efr3a	1817	1250	1517	1921
Efr3b	107	184	158	168
Efs	1305	778	1300	531
Eftud2	2503	2634	2031	2190
Egfl6	61	49	45	27
Egfl7	822	2015	1432	1454
Egfl8	665	560	559	470
Egflam	514	432	942	535
Egfr	1998	1153	1714	952
Egln1	1296	862	837	1423
Egln2	929	1095	880	792
Egln3	3852	1330	3018	1046
Egr1	3852	2064	3813	1465
Egr2	2512	1106	1822	1663
Egr3	507	127	424	169
Ehbp1	335	215	442	236
Ehbp1l1	1396	3563	1884	1992
Ehd1	1526	2108	1575	2015
Ehd2	5882	10864	8174	7079

Ehd3	928	1793	1196	1222
Ehd4	3052	5712	5128	5776
Ehf	1175	223	432	111
Ehhadh	223	232	281	208
Ehmt1	68	160	125	45
Ehmt2	1166	1411	1189	1011
Ei24	4789	3065	4297	4138
Eid1	5838	3944	4548	5327
Eid2	215	319	206	239
Eid2b	144	190	138	148
Eid3	9	0	6	0
Eif1	17407	13616	15655	15234
Eif1a	1903	1340	1455	2764
Eif1ad	1829	1699	1837	1556
Eif1ax	1441	1758	2010	1962
Eif1b	1804	1646	1691	2251
Eif2a	1464	1334	1518	1890
Eif2ak1	542	644	634	490
Eif2ak2	943	617	718	1066
Eif2ak3	387	420	456	564
Eif2ak4	466	275	578	251
Eif2b1	995	1387	1052	1416
Eif2b2	1130	1418	1202	1206
Eif2b3	693	805	815	850
Eif2b4	1424	1367	1035	1106
Eif2b5	2194	2123	2085	1925
Eif2d	900	866	1097	1013
Eif2s1	3853	3107	3209	3441
Eif2s2	6952	5671	5975	6272
Eif2s3	5851	3325	6865	4126
Eif2s3y	0	1684	0	2275
Eif3a	2882	4541	4429	3554
Eif3b	4866	6182	4521	4406
Eif3c	6890	7773	7481	7161
Eif3d	3279	4566	3199	3423
Eif3e	541	362	1137	1109
Eif3f	20312	11475	15879	13874
Eif3g	4044	4484	3471	3255
Eif3h	77	15	75	106
Eif3i	1586	1780	1982	1950
Eif3j	812	500	385	909
Eif3k	4702	5207	4287	3638
Eif3l	5914	6448	6579	6107
Eif3m	9885	5006	6715	6952
Eif4a1	17682	17018	19487	17893
Eif4a2	3911	3843	4514	4894
Eif4a3	4510	4296	4556	4301

Eif4b	8309	7139	8608	8152
Eif4e	1772	1521	1565	1883
Eif4e2	2604	4079	2928	3966
Eif4e3	1025	713	721	816
Eif4ebp1	3835	4268	3102	3328
Eif4ebp2	302	475	439	414
Eif4enif1	118	200	169	86
Eif4g1	8	39	24	29
Eif4g2	7622	6861	6504	7321
Eif4g3	1124	1669	1357	1902
Eif4h	6562	5953	5671	6497
Eif5	5967	4712	4437	5481
Eif5a	31962	29660	25464	23092
Eif5a2	169	248	241	246
Eif5b	354	313	476	237
Eif6	4484	4253	3907	3076
Elac1	104	155	86	236
Elac2	1118	1549	1034	1250
Elane	1	0	3	0
Elavl1	1270	972	1116	1361
Elf1	858	995	1350	1079
Elf2	843	883	793	1172
Elf3	134	54	47	84
Elf4	736	971	1002	1280
Elf5	41	13	30	18
Elfn2	16	0	8	7
Elk1	2315	1977	2329	2757
Elk3	1809	2457	2106	3221
Elk4	2179	2290	3072	2265
Ell	665	717	668	584
Ell2	1074	724	861	968
Elmo1	305	743	572	1197
Elmo2	914	1279	930	1295
Elmod1	186	98	134	60
Elmod3	436	359	486	429
Elmsan1	0	10	9	0
Eln	3640	4394	4193	2062
Elof1	1774	2169	1712	2208
Elovl1	6861	5062	2957	6594
Elovl3	8045	4270	4769	7185
Elovl4	20657	5236	7744	12783
Elovl5	7526	4583	4929	7264
Elovl7	41	116	23	24
Elp2	440	777	873	769
Elp3	1699	1624	1550	1934
Elp4	92	126	149	142
Elp5	1454	1786	1709	1281

Elp6	301	338	265	265
Emb	1971	1476	2350	3676
Emc1	1215	1210	1097	1300
Emc10	2044	2923	1998	2784
Emc2	1590	1485	1696	1813
Emc3	4345	5167	4887	4459
Emc4	3616	2147	2585	3298
Emc6	2330	1345	1905	1493
Emc7	2617	2942	2215	3234
Emc8	836	1097	998	876
Emc9	147	243	125	191
Emcn	570	1866	1211	1186
Emd	1960	1680	1490	2068
Eme1	393	424	328	946
Eme2	1016	854	679	541
Emg1	1672	1808	1454	1518
Emid1	176	217	178	235
Emilin1	322	1259	558	967
Emilin2	767	3204	1107	2179
Emilin3	72	24	14	0
Eml1	1320	1627	1252	1823
Eml2	525	534	415	395
Eml3	1007	917	1186	1021
Eml4	902	833	943	823
Eml6	23	78	33	97
Emp1	5505	13625	11089	16219
Emp2	7917	3576	6584	3469
Emp3	1797	4726	2600	4474
Emx2	92	100	87	163
En1	243	409	444	1074
Enah	3234	2792	2455	3091
Enc1	1829	1434	2377	1952
Endod1	5032	2916	3698	2699
Endog	132	146	97	93
Endou	1227	381	381	98
Endov	159	113	105	58
Eng	1496	5698	2650	3970
Engase	241	249	223	320
Enho	50	225	117	42
Enkd1	51	79	66	35
Eno1	10268	9737	7136	6924
Eno3	73482	42240	13182	57418
Eno4	20	34	24	0
Enoph1	323	516	539	427
Enox1	14	6	15	24
Enox2	178	177	84	96
Enpep	120	654	290	200

Enpp1	2411	2100	2165	1908
Enpp2	592	429	379	316
Enpp3	2440	4193	3635	6325
Enpp4	389	255	322	463
Enpp5	712	585	602	1221
Ensa	3193	2226	2436	2636
Entpd2	1088	1354	928	676
Entpd3	25	7	39	18
Entpd4	244	550	283	436
Entpd5	555	166	325	140
Entpd6	219	208	244	330
Eny2	3303	3516	4438	3651
Eogt	691	797	865	654
Eomes	41	32	57	63
Ep300	568	396	408	546
Ep400	976	1194	1234	1144
Epas1	2699	3228	2764	3143
Epb4.1l5	325	126	232	40
Epb41	589	995	737	1009
Epb41l1	1440	1620	1339	1351
Epb41l2	1477	1526	2257	2302
Epb41l3	125	403	214	507
Epb41l4a	377	322	272	144
Epb41l4b	1737	590	1243	568
Epc1	900	758	812	882
Epc2	529	383	456	692
Epcam	6021	1499	3310	1428
Epdr1	373	407	334	553
Epg5	577	456	561	561
Epgn	986	333	864	260
Epha1	859	873	1193	419
Epha10	1	0	0	13
Epha2	726	479	794	383
Epha3	14	50	54	266
Epha4	313	110	442	78
Epha7	38	0	11	25
Ephb1	259	142	414	61
Ephb2	346	405	215	220
Ephb3	724	592	951	615
Ephb4	1918	1986	2293	1632
Ephb6	1625	1030	1431	401
Ephx1	834	748	879	992
Ephx2	851	602	760	649
Ephx3	396	259	263	84
Epm2aip1	1601	1001	1267	1172
Epn1	686	963	573	875
Epn2	878	992	867	702

Epn3	36	40	20	2
Epor	29	46	29	32
Eppk1	3145	664	2049	538
Eprs	4715	6386	5955	5923
Eps15	3769	2602	3948	2690
Eps15l1	1205	1356	1071	1217
Eps8	588	919	639	1419
Eps8l1	507	330	285	54
Eps8l2	837	515	697	211
Epsti1	353	230	292	629
Eqtn	66	60	22	0
Eral1	792	737	697	881
Erap1	2003	1538	1793	2952
Erbb2	7	13	14	0
Erbb3	1559	1875	1846	874
Erbin	1055	1155	1452	1123
Erc1	754	983	934	898
Ercc1	897	1696	868	1252
Ercc2	302	565	245	161
Ercc3	1089	1140	1455	1667
Ercc4	5	21	8	52
Ercc4l1	44	51	66	33
Ercc5	318	349	445	220
Ercc6	460	302	447	278
Ercc6l	81	90	120	111
Ercc6l2	356	467	582	615
Ercc8	355	415	298	377
Erf	735	623	808	437
Erfe	244	277	250	93
Erg	293	701	459	354
Ergic1	38	82	20	56
Ergic2	1984	2057	1897	2285
Ergic3	4246	4648	4265	5268
Erh	4677	4293	4172	3988
Eri1	864	754	695	924
Eri2	51	65	57	94
Eri3	851	1284	871	1068
Erich1	242	144	114	121
Erich2	35	9	35	29
Erich6b	3	16	0	21
Erlec1	2847	1611	1977	3378
Erlin1	869	939	677	1011
Erlin2	582	658	805	643
Ermard	731	707	540	463
Ermp1	2204	1533	1527	2486
Ern1	333	384	289	375
Ero1a	1819	1485	1586	2362

Ero1b	42	18	84	146
Erp29	1801	2579	2272	3980
Erp44	2712	2803	2717	3216
Errfi1	2954	3119	3658	1941
Esam	1549	3162	2167	2139
Esco1	746	320	489	437
Esco2	215	138	137	128
Esd	2314	1867	2090	2408
Esf1	524	835	769	839
Esm1	87	340	161	184
Espl1	158	283	152	199
Espn	269	87	77	24
Espnl	4	5	5	1
Esr1	177	115	87	58
Esrp1	4051	1730	2373	1146
Esrp2	3628	1536	2812	1791
Esrra	512	726	343	465
Esrrb	0	36	6	7
Esrrg	5	0	32	20
Esyt1	20	11	26	16
Esyt2	1718	2119	2359	2456
Esyt3	507	266	382	178
Etaa1	2	0	11	16
Etf1	5694	5386	6005	5927
Etfa	3974	3343	3427	3965
Etfb	2908	3762	2647	2947
Etfbkmt	363	600	475	586
Etfdh	1982	1660	1845	1954
Ethe1	1353	1111	1127	1035
Etl4	1072	733	498	399
Etnk1	855	783	1004	1089
Ets1	672	1932	1792	1444
Ets2	4288	2170	3400	2166
Etv1	267	150	256	280
Etv3	464	345	521	703
Etv4	628	585	572	309
Etv5	938	601	1351	470
Etv6	130	192	176	250
Eva1a	508	230	394	210
Eva1b	491	873	439	745
Eva1c	553	250	477	271
Evc	530	850	633	617
Evc2	292	314	314	331
Evi2a	644	460	791	1451
Evi2b	259	290	376	994
Evi5	939	863	885	1097
Evi5l	670	576	575	679

Evl	535	600	745	586
Evpl	360	385	259	145
Ewsr1	41	24	57	31
Exd2	341	451	330	463
Exnef	480	325	351	161
Exo1	107	160	84	85
Exo5	333	309	456	368
Exoc2	1124	918	901	1051
Exoc3	852	1123	969	1050
Exoc3l1	32	119	74	56
Exoc3l2	241	455	205	188
Exoc3l4	123	100	90	75
Exoc4	1264	1391	1363	1568
Exoc5	1419	1350	1250	1300
Exoc6b	1098	1376	1128	1208
Exoc8	933	783	1125	1220
Exog	285	380	194	217
Exosc1	708	663	772	621
Exosc10	1374	1715	1366	1560
Exosc2	708	928	879	736
Exosc3	675	639	704	565
Exosc4	845	711	809	531
Exosc5	256	360	220	254
Exosc7	1163	1190	1065	861
Exosc8	860	482	600	621
Exosc9	861	722	1148	839
Exph5	116	60	72	30
Ext1	1897	3333	2403	2210
Ext2	2207	3056	2191	2997
Extl1	448	245	244	210
Extl2	121	183	159	177
Extl3	99	208	132	267
Eya2	151	127	87	116
Eya3	830	1286	1240	1287
Eya4	81	155	167	85
Ezh1	1226	1189	1211	1061
Ezh2	646	687	642	720
Ezr	6364	4668	5534	3378
F10	83	20	83	45
F11r	2397	1813	3089	1246
F13a1	397	731	743	331
F2	21	54	8	1
F2r	1343	1026	987	1554
F2rl1	636	611	727	304
F2rl2	28	35	24	47
F2rl3	12	37	12	4
F3	945	1433	970	515

F5	273	651	342	639
F7	14	8	5	0
F8	136	225	169	179
F8a1	715	925	542	774
Fa2h	23	4	17	20
Faap100	158	169	126	116
Faap20	545	543	441	429
Faap24	160	205	132	227
Fabp12	151	81	75	117
Fabp3	2192	2204	2130	2007
Fabp4	8791	7501	12176	16785
Fabp5	52905	30866	27482	36449
Fadd	346	396	340	479
Fads1	658	882	1156	1410
Fads2	43	100	87	111
Fads2l1	218	99	23	57
Fads3	234	749	364	390
Fads6	477	228	277	346
Faf1	2142	1609	1705	1703
Faf2	1206	1081	1300	1304
Fah	564	350	495	350
Fahd1	778	467	603	529
Fahd2a	956	687	623	584
Fam101a	17	8	18	47
Fam101b	504	553	740	747
Fam102a	863	589	673	807
Fam102b	429	269	344	294
Fam103a1	719	594	528	614
Fam104a	1258	1541	1031	1559
Fam104b	905	821	796	660
Fam105a	890	848	1001	1885
Fam107a	536	677	581	498
Fam107b	511	374	654	1118
Fam109a	275	302	275	243
Fam109b	152	296	254	439
Fam110a	1014	729	996	790
Fam110b	180	214	248	171
Fam110c	151	49	150	22
Fam110d	41	165	75	73
Fam111a	184	389	348	533
Fam114a1	1194	2656	1870	2674
Fam114a1l	39	168	71	198
Fam114a2	1713	803	1025	1106
Fam115c	2227	404	1512	540
Fam117a	390	411	522	436
Fam117b	1053	944	1335	1236
Fam118a	133	270	221	187

Fam118b	933	736	352	930
Fam120a	3459	2703	2018	2089
Fam120b	777	691	885	697
FAM120C	170	136	199	190
Fam122a	267	322	388	404
Fam124a	96	131	152	64
Fam126a	4	73	20	23
Fam126b	99	20	51	31
Fam127b	2985	2522	2358	3173
Fam129a	110	68	69	82
Fam129b	5235	6153	4735	5481
Fam129c	122	16	32	20
Fam131b	57	68	63	134
Fam131c	18	6	2	11
Fam132a	1759	866	1208	488
Fam133b	560	378	570	451
Fam134a	1514	1542	1672	1783
Fam134c	1982	1919	2624	2111
Fam135a	311	287	354	179
Fam136a	1116	938	1022	1127
Fam13a	176	119	250	183
Fam13b	1049	1029	1380	1337
Fam13c	171	116	181	144
Fam149a	45	122	123	136
Fam149b1	787	832	609	638
Fam151a	177	314	92	200
Fam151b	211	116	254	125
Fam159b	4	0	5	14
Fam160a1	237	63	92	32
Fam160a2	355	503	454	427
Fam160b1	664	701	500	885
Fam160b2	1001	902	837	772
Fam161a	12	0	50	4
Fam161b	88	50	93	73
Fam162a	4455	3717	4571	3561
Fam167a	4210	2384	3922	1562
Fam167b	130	529	265	377
Fam168a	836	1064	1124	1147
Fam168b	602	662	970	863
Fam169a	47	113	104	58
Fam169b	227	103	196	106
Fam171a1	293	643	603	869
Fam171a2	45	379	175	165
Fam171b	162	261	126	418
Fam172a	816	659	534	856
Fam173a	261	348	248	238
Fam173b	763	506	542	490

Fam174a	596	309	569	480
Fam174b	61	31	98	184
Fam175a	220	140	205	163
Fam175b	835	863	803	1102
Fam177a1	976	334	576	634
Fam178b	21	55	42	60
Fam179a	41	188	167	26
Fam179b	130	109	208	196
Fam180a	385	283	457	467
Fam184a	77	101	57	24
Fam184b	76	23	9	37
Fam185a	514	509	467	496
Fam188a	1054	940	1351	1066
Fam188b	199	288	253	311
Fam189a2	750	386	417	485
Fam189b	2177	2356	2233	2286
Fam192a	1166	1156	1500	1529
Fam193a	424	500	459	608
Fam193b	394	285	337	303
Fam196a	28	12	20	0
Fam198a	186	140	126	125
Fam198b	1658	2329	2532	4985
Fam19a1	0	5	6	43
Fam19a3	69	109	123	161
Fam19a5	168	78	141	88
Fam204a	471	321	576	449
Fam205a	23	0	8	0
Fam206a	149	305	229	180
Fam207a	551	502	342	311
Fam208a	831	568	847	744
Fam208b	180	213	155	104
Fam20a	172	315	226	182
Fam20b	3228	2674	3859	3085
Fam20c	656	1398	1029	4632
Fam210a	1432	1169	1121	1473
Fam210b	1468	1076	1347	1416
Fam212a	177	200	143	56
Fam212b	465	603	412	251
Fam213a	3386	1630	3005	1959
Fam213b	726	736	463	509
Fam214a	846	584	889	684
Fam214b	1926	2106	1923	1700
Fam216a	638	755	978	777
Fam217a	12	8	9	14
Fam217b	7	0	20	0
Fam219a	481	629	521	485
Fam219b	1225	1535	1796	1502

Fam21c	2565	2203	2671	2810
Fam220a	444	674	466	774
Fam221a	51	34	102	61
Fam222b	507	501	606	635
Fam227a	27	4	29	36
Fam227b	346	196	64	153
Fam228b	70	1	35	0
Fam229b	30	62	69	30
Fam234b	352	255	408	179
Fam25a	2470	2434	818	459
Fam26d	74	0	27	0
Fam26e	173	292	268	182
Fam26f	93	66	89	97
Fam32a	2530	3012	2388	2619
Fam35a	288	209	301	333
Fam3a	1356	727	1074	971
Fam3b	42	11	48	44
Fam3c	1376	1276	1433	2175
Fam3d	26	14	8	127
Fam43a	422	613	676	557
Fam45a	618	555	525	762
Fam46a	1268	1153	1514	2184
Fam46b	693	1006	515	291
Fam46c	64	34	77	193
Fam49a	99	62	129	94
Fam49b	1302	1899	1816	1520
Fam53a	273	379	185	288
Fam53c	683	712	475	646
Fam57a	1786	1282	1408	1174
Fam57b	566	237	375	709
Fam58b	309	379	345	297
Fam60a	24	29	20	14
Fam63a	1569	1070	1499	1204
Fam63b	317	428	484	503
Fam64a	131	242	248	201
Fam65a	473	1296	784	911
Fam65b	82	136	196	264
Fam65c	103	0	123	0
Fam69a	285	635	517	575
Fam69b	92	197	119	117
Fam69c	28	0	2	8
Fam71e1	196	151	86	131
Fam71f1	61	58	65	30
Fam72a	104	70	92	95
Fam76a	788	875	871	1171
Fam76b	520	347	503	455
Fam78a	267	334	170	418

Fam78b	58	100	134	42
Fam81a	93	107	66	25
Fam83a	591	241	405	147
Fam83b	58	33	44	0
Fam83c	369	349	339	109
Fam83d	123	326	196	277
Fam83f	1636	695	1104	393
Fam83g	89	159	133	45
Fam83h	3979	1836	2585	991
Fam84a	3869	749	2138	887
Fam89a	290	171	354	131
Fam89b	2266	3176	1861	2286
Fam8a1	559	647	393	432
Fam91a1	1110	990	1327	1393
Fam92a1	570	690	724	849
Fam92b	266	427	432	553
Fam96a	1345	1504	1538	2032
Fam96b	1035	1554	1112	792
Fam98a	1634	1528	1581	2437
Fam98c	897	921	736	992
Fan1	121	113	143	137
Fanca	137	56	221	72
Fancb	168	121	119	109
Fancc	101	135	128	71
Fancd2	328	422	400	304
Fance	323	213	209	166
Fancf	186	140	143	158
Fancg	91	24	89	94
Fanci	1090	1202	1223	1360
Fancl	417	241	478	226
Fancm	231	227	172	208
Fank1	2322	3988	3010	8383
Fap	930	1588	1065	1830
Far1	3549	1827	1753	2630
Far2	101	128	55	47
Farp1	1077	1394	1455	1622
Farp2	656	947	674	1056
Fars2	674	860	638	702
Farsa	1058	1555	1291	1167
Farsb	1219	1561	1186	1776
Fas	115	90	226	236
Faslg	33	24	21	0
Fasn	10878	4523	4769	6388
Fastk	1161	1658	866	1130
Fastkd1	3116	4389	3327	2747
Fastkd2	632	405	406	525
Fastkd3	404	297	289	408

Fastkd5	413	460	496	361
Fat1	3645	3056	4512	3237
Fat2	1685	927	1384	467
Fat3	0	17	24	42
Fat4	26	87	51	78
Fau	182	444	114	167
Faxc	53	63	45	19
Faxdc2	4206	1374	2284	1681
Fbf1	267	328	214	203
Fbl	3333	3246	3099	2520
Fblim1	1723	1442	2102	1995
Fbln1	635	1007	847	977
Fbln2	4489	8135	5392	7117
Fbln5	1682	1735	2009	3876
Fbln7	432	351	1246	429
Fbn1	5322	17438	5865	5332
Fbn2	390	611	879	2778
Fbp1	42	0	32	91
Fbp2	707	215	60	921
Fbrs	1208	1260	1204	1484
Fbrsl1	565	479	436	395
Fbxl12	482	429	330	462
Fbxl14	711	784	712	876
Fbxl15	112	93	114	58
Fbxl16	13	43	47	16
Fbxl17	59	49	126	104
Fbxl19	744	616	677	518
Fbxl2	104	199	144	82
Fbxl20	750	937	862	753
Fbxl21	56	62	33	49
Fbxl22	12	40	25	11
Fbxl3	1892	2030	2139	2215
Fbxl4	199	225	191	197
Fbxl5	2006	1742	2421	2434
Fbxl6	547	526	450	478
Fbxl7	247	170	406	283
Fbxl8	282	373	298	209
Fbxo10	176	271	215	166
Fbxo11	348	472	357	253
Fbxo15	18	32	44	20
Fbxo17	41	82	18	23
Fbxo18	1464	2123	2003	2385
Fbxo21	471	492	453	399
Fbxo22	1343	1760	1365	1316
Fbxo25	314	374	274	447
Fbxo27	244	170	269	351
Fbxo28	1659	1177	1375	1106

Fbxo3	789	685	790	795
Fbxo30	358	233	298	427
Fbxo31	484	680	448	352
Fbxo32	504	342	542	359
Fbxo33	624	384	399	558
Fbxo34	663	1092	594	688
Fbxo38	672	644	713	540
Fbxo4	489	427	345	349
Fbxo40	1405	553	500	1036
Fbxo42	444	386	530	507
Fbxo44	123	75	95	40
Fbxo45	617	634	743	643
Fbxo46	246	344	223	222
Fbxo48	11	19	33	6
Fbxo5	150	102	190	153
Fbxo6	1177	1071	706	1317
Fbxo7	991	1177	1116	917
Fbxo8	449	350	525	478
Fbxo9	1081	1014	883	836
Fbxw11	1185	1199	1467	1485
Fbxw17	429	525	531	497
Fbxw2	1481	1845	1428	1775
Fbxw4	247	171	208	122
Fbxw5	1042	1373	709	1026
Fbxw7	461	99	393	161
Fbxw8	691	980	742	751
Fbxw9	281	291	336	180
Fcer1a	26	31	26	25
Fcer1g	9860	7535	8821	22279
Fcf1	3711	2455	2618	2240
Fcgbp	11659	1247	3063	2317
Fcgr1a	2057	2332	2857	5989
Fcgr2a	16	111	32	131
Fcgr2b	1612	1575	1771	2936
Fcgr3a	1174	437	361	1048
Fcgrt	3115	6251	4463	5045
Fcho1	24	2	18	4
Fcho2	990	647	579	1385
Fchsd1	497	280	409	187
Fchsd2	223	440	475	579
Fcmr	368	683	606	143
Fcna	539	1122	984	871
Fcnb	1220	929	1255	908
Fcrl2	1122	1081	1768	4093
Fcrla	53	26	41	61
Fcrlb	81	47	17	36
Fdft1	4326	1958	2563	2576

Fdps	12862	9119	7840	9459
Fdx1	957	813	865	835
Fdx1l	1160	1270	1136	1073
Fdxacb1	296	164	158	251
Fdxr	169	219	206	113
Fech	989	785	843	1049
Fem1a	3080	3562	1801	2898
Fem1b	509	391	448	441
Fem1c	521	535	474	612
Fen1	578	935	873	681
Fer	781	934	889	1003
Fer1l5	27	13	23	34
Fer1l6	11	0	3	0
Fermt1	1268	622	975	488
Fermt2	2189	4278	3006	3548
Fermt3	594	1119	937	1965
Fes	382	772	477	617
Fetub	181	156	138	44
Fez1	293	434	181	117
Fez2	1199	1541	1350	1490
Ffar2	37	14	50	4
Ffar4	8	5	17	0
Fgd2	288	366	298	532
Fgd3	377	316	286	474
Fgd4	47	91	41	158
Fgd5	238	525	456	500
Fgd6	68	68	56	48
Fgf1	357	385	310	276
Fgf10	0	9	3	0
Fgf11	181	301	92	113
Fgf12	97	10	71	36
Fgf13	428	220	298	237
Fgf14	15	12	18	41
Fgf16	61	141	117	22
Fgf18	111	352	45	22
Fgf2	0	5	18	0
Fgf22	47	49	69	26
Fgf7	265	407	525	517
Fgfbp1	3578	2745	1848	995
Fgfbp3	56	51	32	34
Fgfr1	3846	5938	4317	5409
Fgfr1op	612	285	487	576
Fgfr1op2	1708	1821	1882	1981
Fgfr2	2228	798	1766	1152
Fgfr3	1767	550	907	483
Fgfr4	107	557	75	61
Fgfrl1	236	317	373	304

Fggy	555	454	472	556
Fgl1	59	20	33	41
Fgl2	3030	2516	2902	3001
Fgr	592	670	746	1691
Fh	4220	3905	3348	4024
Fhad1	46	31	8	0
Fhdc1	1377	469	1137	376
Fhit	199	57	57	58
Fhl1	83271	48701	34601	44767
Fhl2	1143	791	1292	969
Fhl3	2121	2714	1873	2641
Fhl4	45	57	50	73
Fhl5	22	0	5	22
Fhod1	723	1131	555	1000
Fhod3	943	956	852	690
Fibin	462	762	722	1361
Fibp	1429	1638	1815	1863
Ficd	310	442	275	432
Fig4	559	931	552	870
Figf	255	562	636	744
Fignl1	0	54	36	24
Filip1	236	601	363	440
Filip1l	1544	1411	2046	1614
Fip1l1	848	1136	1216	1184
Fis1	2674	2974	2037	2868
Fitm1	775	956	319	380
Fitm2	2517	1636	1437	1815
Fiz1	836	729	987	755
Fjx1	864	448	952	618
Fkbp10	2729	8681	5481	10801
Fkbp11	953	1655	1357	2317
Fkbp14	1020	1676	1455	2459
Fkbp15	1035	1433	970	1911
Fkbp1a	12284	14975	15568	18635
Fkbp1b	39	33	93	206
Fkbp2	2548	2767	2525	2519
Fkbp3	6235	4184	3838	5087
Fkbp4	5363	4211	3924	3866
Fkbp5	1614	4958	1294	4062
Fkbp7	1534	2406	1857	4351
Fkbp8	2371	3067	1936	1953
Fkbp9	2698	4515	5186	8843
Fkbpl	486	441	451	446
Fkrp	560	642	575	688
Fktn	538	404	620	430
Flad1	938	1198	862	1177
Flcn	1336	1312	987	1193

Flg	247	452	41	34
Flg2	0	19	3	0
Fli1	500	821	844	1224
Flii	3071	4182	3090	3141
Flna	13773	21584	17832	22878
Flnb	102	188	236	24
Flnc	9950	14430	7324	6855
Flot1	1390	2120	1148	1150
Flot2	1765	2161	2225	2020
Flrt1	88	150	167	19
Flrt2	5017	2207	4965	4049
Flrt3	273	106	346	144
Flt1	757	2481	1267	1394
Flt3	142	157	87	401
Flt3lg	218	337	256	184
Flt4	72	355	226	172
Flvcr1	14	6	0	53
Flvcr2	370	314	245	426
Flywch1	717	875	578	549
Flywch2	97	41	56	84
Fmc1	644	552	418	402
Fmn1	81	31	62	37
Fmnl1	492	1013	831	1180
Fmnl3	997	1636	1344	1676
Fmo1	238	92	203	118
Fmo2	101	54	72	4
Fmo3	79	170	21	34
Fmo4	83	36	87	71
Fmo5	120	229	65	151
Fmod	632	782	454	464
Fmr1	123	93	110	66
Fn1	31535	92052	80093	107069
Fn3k	114	91	17	118
Fn3krp	273	313	492	534
Fnbp1	257	182	214	216
Fnbp1l	585	351	581	812
Fnbp4	1419	1122	1244	1213
Fndc1	3331	9046	3402	9344
Fndc10	180	185	128	132
Fndc3a	358	330	326	406
Fndc3b	759	1638	1381	2134
Fndc3c1	0	177	42	4
Fndc5	21	113	47	40
Fndc8	4	7	3	0
Fnip1	240	172	217	356
Fnip2	526	507	528	1348
Fnta	2097	2320	2364	2769

Fntb	446	556	462	368
Focad	725	1100	1033	988
Folr1	78	292	101	71
Folr2	2370	3786	3528	6601
Fopnl	1260	1346	1307	1189
Fos	816	1439	757	805
Fosb	979	193	837	85
Fosl1	617	802	546	290
Fosl2	121	100	134	82
Foxc1	504	300	352	250
Foxc2	27	7	57	12
Foxd3	14	0	20	0
Foxe1	165	67	402	76
Foxf1	14	28	12	16
Foxh1	0	39	39	0
Foxj2	920	739	967	846
Foxj3	1336	776	999	1268
Foxk1	21	53	29	26
Foxk2	1205	1413	897	1364
Foxm1	49	127	58	0
Foxn1	148	9	196	27
Foxn2	371	532	418	589
Foxn3	2802	2784	2469	3228
Foxo1	26	31	65	15
Foxo3	42	45	18	7
Foxo4	944	920	710	1032
Foxo6	118	117	86	89
Foxp2	66	4	71	33
Foxp3	17	1	4	15
Foxp4	914	913	797	920
Foxq1	672	107	397	112
Foxr1	29	3	11	0
Foxred2	10	26	11	0
Foxs1	91	337	134	213
Fpgs	115	255	77	115
Fpgt	34	66	45	92
Fra10ac1	869	635	825	542
Fras1	9	39	2	2
Frat1	167	119	179	164
Frat2	305	227	275	339
Frem1	69	41	99	104
Frem2	17	18	9	12
Frg1	181	221	211	262
Frg1l1	1845	1242	1732	1608
Frk	224	168	217	644
Frmd4a	6	54	23	0
Frmd4b	372	561	691	752

Frmd5	2	10	12	1
Frmd6	1952	2246	1957	1617
Frmd7	27	24	63	0
Frmd8	2242	1891	2188	1802
Frmpd1	25	276	96	50
Frrs1	2199	1832	1672	2850
Frs2	562	380	497	424
Frs3	27	45	44	27
Fry	95	175	140	117
Fryl	1107	881	1214	710
Frzb	33	39	65	10
Fscn1	1987	4459	2875	3143
Fscn2	14	19	21	25
Fsd1l	367	243	200	255
Fsd2	519	548	236	490
Fsip1	4	107	132	123
Fst	1636	1515	1703	923
Fstl1	11365	32220	15351	23196
Fstl3	716	442	728	392
Ftcd	37	27	56	15
Fth1	41735	54952	53325	74216
Ftl1	96	376	104	253
Fto	1292	2021	2033	1962
Ftsj1	896	722	743	588
Ftsj3	1198	1751	1387	1574
Ftx	65	62	66	67
Fubp1	1046	1016	1410	1198
Fubp3	117	204	233	266
Fuca1	3934	5071	4971	6568
Fuca2	670	704	800	726
Fuk	96	115	129	78
Fundc1	461	263	397	817
Fundc2	162	366	126	142
Furin	404	1468	752	1582
Fus	5442	3615	4866	4006
Fut1	1080	419	1181	448
Fut10	98	152	116	104
Fut11	808	855	915	1327
Fut2	262	116	147	235
Fut4	161	312	227	462
Fut7	62	97	169	76
Fut8	567	1017	1019	1354
Fuz	381	343	122	196
Fv1	906	952	850	785
Fxn	691	674	444	395
Fxr1	47	39	47	137
Fxr2	1492	1607	1681	1495

Fxyd1	2707	2910	1431	1480
Fxyd2	2479	3983	3352	5792
Fxyd3	19993	8603	13421	5799
Fxyd4	110	13	84	18
Fxyd5	3400	8937	5046	9587
Fxyd6	640	1580	1162	1547
Fxyd7	15	46	26	8
Fyb	260	204	358	458
Fyco1	1321	845	952	1295
Fyn	817	1754	1581	2029
Fyttd1	2017	1582	1902	1827
Fzd1	1395	1279	2173	2063
Fzd10	332	158	232	67
Fzd2	1384	963	1437	1357
Fzd3	101	26	156	97
Fzd4	944	1045	995	931
Fzd6	2176	936	2021	738
Fzd7	83	80	60	75
Fzd8	16	21	6	12
Fzd9	42	114	60	41
Fzr1	70	252	77	197
G0s2	812	1319	427	160
G2e3	441	267	343	239
G3bp1	5963	5417	5119	5528
G3bp2	1835	1723	1578	1854
G4	370	445	534	513
G6pc3	771	1081	972	1416
G6pd	5113	6202	5343	8264
Gaa	946	1617	1070	1937
Gab1	1335	724	1578	1216
Gab2	329	600	651	1240
Gabarap	19786	14896	17641	30675
Gabarapl1	3219	2788	3485	4233
Gabarapl2	1020	1258	883	956
Gabbr1	1108	985	960	764
Gabbr2	0	169	17	17
Gabpa	415	554	570	366
Gabpb1	565	356	599	387
Gabpb1l	36	88	39	40
Gabpb2	1567	1501	1398	1188
Gabra2	12	35	33	22
Gabra4	22	9	77	26
Gabrd	0	58	3	0
Gabrp	41	10	75	16
Gabrr1	1	39	6	77
Gabrr2	44	103	66	64
Gadd45a	1659	2654	3612	1156

Gadd45b	452	642	436	580
Gadd45g	753	772	372	426
Gadd45gip	979	1469	756	755
Gadl1	676	305	92	209
Gak	1179	1290	1234	1194
Gal	0	90	0	33
Gal3st4	135	110	226	378
Galc	337	283	503	599
Gale	659	980	738	837
Galk1	242	727	287	467
Galk2	671	818	1215	1250
Galm	433	473	492	1019
Galns	502	935	821	1410
Galnt1	1640	1823	2079	2276
Galnt10	845	1110	996	1721
Galnt11	421	739	572	700
Galnt12	138	191	117	193
Galnt14	12	12	47	76
Galnt15	137	148	161	124
Galnt16	826	2621	999	1922
Galnt18	278	450	384	301
Galnt2	1878	2217	2222	2130
Galnt3	217	92	152	45
Galnt4	402	288	260	297
Galnt5	15	10	2	15
Galnt6	390	589	409	772
Galnt7	222	286	220	394
Galntl5	7	11	5	33
Galt	518	556	296	313
Gamt	742	1184	466	821
Gan	172	93	175	93
Ganab	5464	5512	6599	6746
Ganc	667	399	421	441
Gap43	195	796	152	84
Gapdh	21168	17115	10228	13093
Gapt	75	24	5	8
Gapvd1	951	1069	996	1306
Gar1	1023	1058	859	596
Garem1	5	4	8	30
Garnl3	27	72	44	38
Gars	4910	5861	5875	5546
Gart	1515	1764	1485	1420
Gas1	4613	3366	5633	3752
Gas2l1	488	455	246	362
Gas5	2085	1503	1878	1375
Gas6	2586	5624	3020	3724
Gas7	4342	8237	5442	5878

Gas8	79	67	72	43
Gata2	92	241	196	51
Gata3	2780	940	1279	594
Gata6	237	59	53	114
Gatad1	1681	1498	1787	2060
Gatad2a	2445	2241	2227	2047
Gatad2b	548	554	602	635
Gatb	422	590	445	595
Gatc	1192	0	5	6
Gatm	147	608	295	304
Gatsl2	488	821	745	560
Gatsl3	1410	824	1238	569
Gba	1460	2181	1530	3000
Gba2	155	263	140	242
Gbas	4208	2519	2504	3032
Gbe1	484	663	567	668
Gbf1	661	1184	648	926
Gbp1	40	79	5	22
Gbp2	3028	1590	2030	2223
Gbp4	183	12	77	155
Gbp5	435	199	296	421
Gca	31	35	48	7
Gcat	226	156	60	43
Gcc1	339	267	406	524
Gcc2	303	278	221	297
Gcdh	1462	1295	1546	1465
Gcfc2	339	282	316	423
Gcgr	15	14	24	58
Gch1	372	229	275	356
Gchfr	57	260	101	135
Gclc	608	680	700	606
Gclm	610	452	651	641
Gcn1l1	1224	1321	1092	1038
Gcnt1	240	280	296	121
Gcnt2	636	563	663	823
Gcnt6	0	66	20	76
Gcsam	358	68	191	437
Gcsh	1459	1660	1637	1845
Gda	5138	6979	5790	10305
Gdap1	7	50	8	3
Gdap1l1	11	25	2	14
Gdap2	443	254	277	530
Gde1	451	554	498	841
Gdf10	241	69	179	56
Gdf11	29	64	30	24
Gdf5	72	88	77	33
Gdi1	3697	2758	4198	3784

Gdi2	11833	7658	9645	13567
Gdnf	52	107	57	79
Gdpd1	248	186	202	179
Gdpd2	455	178	144	84
Gdpd3	95	50	30	38
Gdpd5	358	357	281	646
Gdpgp1	115	80	71	105
Gem	223	283	525	493
Gemin2	377	333	421	496
Gemin4	1000	850	1035	1039
Gemin5	450	573	512	584
Gemin6	297	227	328	338
Gemin7	184	189	96	121
Gemin8	461	413	426	433
Gen1	60	24	78	70
Get4	1199	1397	979	1249
Gfap	14	23	23	0
Gfer	506	564	501	421
Gfm1	2021	2095	2124	2106
Gfm2	189	233	196	355
Gfod1	310	466	339	421
Gfod2	177	193	218	145
Gfpt1	1083	985	1392	1836
Gfpt2	1578	1674	1416	2571
Gfra1	240	388	311	228
Gfra2	137	232	179	82
Gfra3	310	260	176	149
Gfra4	133	82	83	35
Gga1	1390	1731	1145	1368
Gga2	913	1263	1011	1976
Gga3	578	728	474	649
Ggact	522	172	325	276
Ggct	1856	542	1580	625
Ggcx	1571	2255	1849	3015
Ggh	1377	933	1509	1288
Ggn	16	41	12	10
Ggnbp1	18	13	9	23
Ggnbp2	2563	1554	2281	2338
Ggps1	623	331	570	583
Ggt1	47	10	44	27
Ggt5	298	812	448	408
Ggt6	488	286	280	239
Ggt7	70	177	62	137
Ghdc	315	477	403	660
Ghitm	8537	6367	6477	7480
Ghr	1657	850	985	959
Ghrl	72	46	161	85

Gid4	1129	1032	918	879
Gid8	1322	1476	1726	1520
Gigyf1	984	721	975	585
Gigyf2	793	781	709	882
Gimap1	96	126	155	233
Gimap4	395	885	856	580
Gimap5	374	578	490	339
Gimap6	456	490	692	513
Gimap7	205	99	224	317
Gimap8	120	193	137	138
Gimap9	240	371	336	232
Gin1	123	122	125	98
Ginm1	2183	1966	2701	3191
Gins1	238	391	295	326
Gins2	581	579	559	614
Gins3	149	253	229	146
Gins4	520	725	551	726
Giot1	144	104	110	72
Gip	460	177	527	116
Gipc1	2654	2640	2276	2676
Gipc2	83	171	110	89
Gipc3	18	34	24	16
Gipr	31	27	27	29
Git1	1730	2902	1768	1792
Git2	995	1393	940	1125
Gja1	23456	5364	18949	10945
Gja3	56	116	45	19
Gja4	397	1310	712	783
Gja5	425	457	524	422
Gjb1	5	0	24	7
Gjb2	9217	4062	6507	1747
Gjb3	2846	1494	1670	1157
Gjb4	1724	645	988	551
Gjb5	1009	534	466	544
Gjb6	860	182	215	48
Gjc1	425	891	612	755
Gjc2	90	144	187	81
Gjc3	39	11	8	13
Gk	839	373	432	610
Gkap1	697	553	709	679
Gla	1308	1162	1410	2439
Glb1	1782	3189	2746	4027
Glb1l	456	943	693	1077
Glb1l2	137	36	47	68
Glcci1	136	188	227	204
Glce	1404	768	1222	918
Gldc	21	5	15	7

Gldn	67	10	27	39
Gle1	903	740	718	592
Glg1	533	951	856	1075
Gli1	24	5	3	2
Gli2	325	129	379	186
Gli3	15	42	63	8
Gli4	19	27	14	3
Glipr1	1149	842	1293	3018
Glipr2	1167	3185	2097	2473
Glis1	20	18	27	61
Glis2	591	925	820	856
Glmn	495	508	376	507
Glmp	3874	4973	3903	6266
Glo1	6851	4097	5204	6322
Glod4	5159	3234	4660	3752
Glod5	12	86	42	215
Glrx	3550	2387	1972	3121
Glrx2	2108	1569	1736	1614
Glrx3	7869	5208	7569	6377
Glrx5	2723	1820	1772	1684
Gls	1183	1250	1335	1556
Gls2	558	321	556	546
Glt1d1	25	0	12	78
Glt6d1	6	30	5	0
Glt8d1	1492	1459	1805	2311
Glt8d2	247	1020	778	1408
Gltp	3085	2821	2671	1894
Gltscr1	608	710	688	657
Gltscr1l	320	390	439	522
Gltscr2	6122	4341	5231	4639
Glud1	4773	7791	5844	9543
Glul	9914	16668	12347	19095
Glyctk	198	187	172	125
Glyr1	1093	1452	1425	1370
Gm2a	6155	9803	9354	27881
Gm5471	8616	6498	3936	3976
Gmcl1	638	280	456	386
Gmds	464	518	504	757
Gmeb1	187	166	156	127
Gmeb2	6	13	0	5
Gmfb	3044	1970	2093	3436
Gmfg	2143	3018	3066	6191
Gmip	643	464	575	709
Gmnn	628	682	751	605
Gmppa	783	871	715	724
Gmpr	1363	1031	670	1078
Gmpr2	847	388	714	863

Gmps	2392	2197	2464	2362
Gna11	569	794	536	710
Gna12	136	1565	1224	1369
Gna13	2038	1938	2176	3643
Gna14	83	33	80	121
Gna15	1063	933	925	799
Gnai1	1685	652	978	1243
Gnai2	5705	12158	9405	14278
Gnai3	2568	2827	3179	3901
Gnal	10	19	17	41
Gnao1	38	106	45	27
Gnaq	44	58	104	26
Gnas	31185	34741	23929	36268
Gnat1	15	37	21	61
Gnaz	42	21	8	0
Gnb1	5500	4186	4065	5109
Gnb2	2094	3777	2188	2655
Gnb3	59	51	71	101
Gnb4	937	1120	1353	1761
Gnb5	207	224	293	278
Gne	322	693	373	760
Gng10	1950	2742	2353	3657
Gng12	3578	3150	3710	3233
Gng2	1433	1455	1672	3277
Gng3	76	57	44	39
Gng4	10	0	6	15
Gng5	75	86	32	21
Gng7	14	8	8	36
Gng8	223	159	156	146
Gngt2	1384	1194	1569	3812
Gnl1	1052	1423	721	1037
Gnl2	1671	2101	1663	1752
Gnl3	1425	1254	1758	1390
Gnl3l	1796	1152	1408	1474
Gnmt	53	21	29	0
Gnpat	2383	1922	1801	2150
Gnpda1	1678	1778	2021	2605
Gnpda2	350	227	308	394
Gnpnat1	631	506	497	687
Gnptab	448	636	569	1209
Gnptg	653	635	639	621
Gnrh1	38	15	68	23
Golga1	410	365	340	406
Golga2	754	2152	1377	1771
Golga3	531	432	602	961
Golga4	1930	1699	2179	2179
Golga5	1232	1277	1852	1590

Golga7	1490	1288	1330	1484
Golga7b	235	74	263	205
Golgb1	984	1455	1181	1463
Golim4	584	903	1034	1114
Golm1	314	242	257	325
Golph3	841	971	1206	1596
Golph3l	421	382	277	700
Golt1b	1753	2204	2090	3496
Gon4l	80	223	180	107
Gopc	1032	681	850	1039
Gorab	189	143	357	193
Gorasp1	562	732	560	539
Gorasp2	747	598	432	749
Gosr1	856	901	892	932
Gosr2	2817	2861	3059	3872
Got1	3568	2820	2132	4010
Got2	10062	11403	9009	10133
Gp1bb	1030	962	949	1106
Gp6	10	17	26	11
Gpaa1	1164	1274	1277	1019
Gpalpp1	1024	907	1253	1212
Gpam	2041	896	1326	986
Gpank1	429	574	552	473
Gpat3	206	172	178	259
Gpat4	1874	2139	2221	2084
Gpatch1	189	278	224	251
Gpatch11	522	728	664	524
Gpatch2	560	408	487	502
Gpatch2l	819	586	624	527
Gpatch3	598	674	515	446
Gpatch4	431	458	510	439
Gpatch8	524	590	812	584
Gpbp1	1804	1220	1523	2063
Gpbp1l1	7	15	12	27
Gpc1	4009	7558	4174	5551
Gpc2	45	13	30	0
Gpc3	217	276	497	174
Gpc4	2379	1570	2325	1494
Gpc6	601	661	936	2211
Gpcpd1	1360	618	805	1009
Gpd1	3219	1489	707	2441
Gpd1l	1924	1658	2150	2016
Gpd2	591	559	397	797
Gper1	112	158	185	187
Gphn	425	444	323	555
Gpi	10015	11397	7220	10684
Gpihbp1	349	628	548	732

Gpkow	615	708	826	650
Gpld1	550	113	357	88
Gpm6b	222	135	173	212
Gpn1	954	886	814	1041
Gpn3	357	479	394	295
Gpnmb	19743	21916	32630	142762
Gpr1	2	6	15	0
Gpr107	857	1306	1143	1513
Gpr108	983	1194	808	925
Gpr132	86	80	69	150
Gpr137	223	255	360	285
Gpr137b	831	932	1247	3175
Gpr143	5	0	5	26
Gpr146	938	1263	1220	1275
Gpr15	19	13	20	0
Gpr150	26	23	15	7
Gpr153	302	791	562	1280
Gpr155	589	424	521	467
Gpr157	51	71	32	64
Gpr160	179	115	203	187
Gpr161	379	262	463	269
Gpr162	1728	3429	2433	4193
Gpr171	338	127	265	304
Gpr176	472	470	657	1403
Gpr18	60	7	35	62
Gpr180	564	934	743	890
Gpr182	118	140	144	101
Gpr183	313	208	367	875
Gpr19	109	175	87	153
Gpr27	202	183	295	203
Gpr3	21	15	41	26
Gpr34	85	83	116	284
Gpr35	72	16	74	67
Gpr37	45	20	15	40
Gpr37l1	22	0	23	42
Gpr4	71	417	187	152
Gpr52	17	7	29	54
Gpr63	7	0	15	0
Gpr65	51	95	69	193
Gpr68	490	603	688	856
Gpr84	12	13	11	14
Gpr85	17	0	38	32
Gpr87	1551	637	1133	332
Gpr88	156	154	269	306
Gpr89b	855	620	704	648
Gprasp1	323	313	483	247
Gprc5a	165	400	238	267

Gprc5b	52	462	98	0
Gprc5c	799	1427	1125	338
Gprc5d	234	30	129	0
Gprin1	18	17	19	41
Gprin3	21	0	11	0
Gps1	1246	1811	892	1367
Gps2	2744	2783	2104	2554
Gpsm1	247	412	305	633
Gpsm2	1084	798	1348	690
Gpsm3	390	323	465	795
Gpt	1606	958	925	1125
Gpt2	938	1290	471	727
Gpx1	10837	15125	10899	20131
Gpx2	4848	2403	4261	1388
Gpx3	22831	37358	20396	27655
Gpx4	4013	4990	3339	4681
Gpx7	1733	4071	2189	4975
Gpx8	2210	3579	3492	5342
Gramd1a	509	807	703	598
Gramd1b	454	440	471	393
Gramd3	2047	1521	1793	2925
Gramd4	343	305	432	280
Grap	7	127	8	44
Grasp	454	545	656	504
Grb10	1349	3856	1822	3133
Grb14	506	398	492	353
Grb2	466	733	534	867
Grb7	204	129	129	97
Grcc10	14	10	3	3
Greb1	13	31	23	20
Grem1	252	225	345	971
Grem2	431	225	528	723
Grhl1	941	381	639	381
Grhl2	833	459	614	262
Grhl3	313	139	199	108
Grhpr	996	598	483	524
Gria2	30	17	3	0
Gria3	11	10	33	156
Grifin	20	34	8	22
Grik4	17	13	14	5
Grik5	63	34	21	26
Grin1	7	0	5	0
Grin2d	77	180	155	290
Grin3b	228	144	115	161
Grina	5427	7570	5277	6366
Grip1	113	51	113	18
Grip2	55	13	9	20

Gripap1	878	945	790	1094
Grk2	944	1377	898	1235
Grk3	212	157	299	813
Grk4	120	113	87	200
Grk5	293	676	754	448
Grk6	1260	1467	1470	1697
Grm6	26	9	39	16
Grm8	62	0	63	23
Grn	11427	15865	16064	30960
Grp	9	0	2	0
Grpel1	2499	2322	1902	2104
Grpel2	574	504	570	432
Grsf1	2434	2340	2540	3502
Grtp1	5027	3034	3928	3606
Grwd1	585	694	575	512
Gsap	433	416	308	912
Gsc	10	3	11	0
Gsdma	573	283	339	124
Gsdmc	122	109	96	0
Gsdmd	745	1530	955	1220
Gse1	65	106	42	53
Gsg1l	20	26	17	29
Gsg2	235	285	305	266
Gsk3a	2396	2952	2094	2221
Gsk3b	2352	2026	2487	2575
Gskip	659	771	802	966
Gsn	20304	20508	12786	8416
Gspt2	51	35	77	0
Gsr	806	1123	1035	1105
Gss	1584	1366	2132	1657
Gsta4	1010	442	586	803
Gstcd	301	252	330	235
Gstk1	477	473	576	515
Gstm1	9981	6787	5958	6673
Gstm6	25	20	14	31
Gstm7	530	271	215	349
Gsto1	4639	4025	5248	5364
Gstp1	10942	8757	11313	7436
Gstt1	82	141	81	78
Gstt2	2	23	6	0
Gstt3	687	342	414	206
Gstt4	11	3	0	6
Gstz1	896	562	853	743
Gtdc1	203	331	260	215
Gtf2a1	8	78	21	31
Gtf2b	941	1179	1345	1504
Gtf2e1	443	570	474	404

Gtf2e2	1225	1305	1669	1206
Gtf2f1	1079	1971	1130	1403
Gtf2f2	1123	936	865	1028
Gtf2h1	829	1330	1093	1476
Gtf2h2	702	338	489	514
Gtf2h3	773	853	846	629
Gtf2h4	221	264	244	118
Gtf2h5	3010	2517	3059	2343
Gtf2i	1865	1821	1819	1295
Gtf2ird1	503	312	636	371
Gtf2ird2	179	144	188	180
Gtf3a	854	966	657	693
Gtf3c1	1210	1106	952	769
Gtf3c2	1736	1408	1544	1474
Gtf3c3	533	416	516	343
Gtf3c4	679	833	728	628
Gtf3c5	365	638	423	454
Gtf3c6	969	1400	1199	1308
Gtpbp1	573	850	862	886
Gtpbp10	419	342	330	451
Gtpbp2	687	781	715	786
Gtpbp3	160	100	62	134
Gtpbp4	229	225	182	80
Gtpbp8	253	136	99	285
Gtse1	132	304	223	176
Guca1b	562	408	445	409
Guca2a	21	28	8	32
Guca2b	20	15	38	29
Gucd1	169	294	214	173
Gucy1a3	301	310	427	320
Gucy1b3	75	158	289	81
Gucy2g	31	0	6	0
Guf1	1692	754	825	932
Guk1	2787	2990	3009	2796
Gulp1	161	203	120	425
Gusb	2182	3057	3974	10238
Gxylt1	615	795	633	669
Gxylt2	336	274	515	598
Gyg1	5884	3523	3548	4929
Gypa	0	6	23	0
Gypc	785	945	1080	947
Gys1	3680	2681	2352	2839
Gys2	14	22	29	0
Gzf1	632	429	557	719
Gzmb	181	8	11	45
Gzmk	38	12	77	191
Gzmm	114	41	38	94

H19	173812	170094	89036	71035
H1f0	10443	7832	11284	9514
H1fx	112	150	126	78
H2afv	1027	914	794	981
H2afx	3397	2711	3447	2584
H2afy	2943	3178	2856	3195
H2afz	3963	1896	2294	3059
H3f3b	20631	10275	14417	15590
H3f3c	102	139	38	6
H6pd	884	1497	1028	1479
Haao	3	11	21	13
Habp4	2589	2743	2502	2069
Hacd1	1103	1478	954	982
Hacd2	4456	2283	2466	3009
Hacd3	1540	1072	1172	1290
Hacd4	351	185	312	869
Hace1	143	274	227	219
Hacl1	7754	2843	3590	6847
Hadh	354	413	497	550
Hadha	4309	4831	4434	4967
Hadhb	7817	4416	4693	6436
Hagh	657	819	555	549
Haghl	282	194	310	210
Hal	264	135	12	26
Hap1	133	53	119	38
Hapln3	75	37	33	90
Hapln4	364	202	169	472
Harbi1	390	412	423	435
Hars	2522	2429	2765	2456
Hars2	631	496	549	590
Has1	221	86	172	144
Has2	333	359	652	313
Has3	309	582	271	122
Hat1	1399	1621	1432	1271
Haus3	157	211	257	276
Haus4	446	864	716	913
Haus5	423	569	441	334
Haus7	1143	1800	1304	1393
Haus8	177	272	105	151
Havcr1	15	12	27	92
Havcr2	162	131	167	287
Hax1	868	1039	715	882
Hba1	1346	3361	4458	1219
Hbb	1396	3662	3579	1785
Hbe1	99	1	38	38
Hbegf	440	453	536	156
Hbp1	2062	2047	1738	1994

Hbs1l	2675	2443	2743	2436
Hccs	359	363	349	456
Hcfc1	2	48	59	6
Hcfc1r1	3736	3748	3091	2881
Hcfc2	294	518	433	462
Hck	512	563	569	1983
Hcls1	788	1221	993	2187
Hcn2	25	22	6	20
Hcst	71	61	107	211
Hdac1	2471	2447	2647	2213
Hdac10	298	363	224	185
Hdac11	197	162	170	121
Hdac1l	288	574	757	652
Hdac2	3129	2757	4463	3289
Hdac3	2947	2732	3149	2581
Hdac4	908	876	828	846
Hdac5	664	720	661	891
Hdac7	39	418	164	173
Hdac8	115	85	104	132
Hdac9	57	79	62	146
Hdc	172	1089	363	82
Hddc2	864	723	1067	723
Hddc3	1356	1054	1244	1053
Hdgf	6561	6809	7275	5937
Hdgfrp2	1623	1504	867	1237
Hdgfrp3	53	129	202	138
Hdhd2	2423	1598	1595	2456
Hdhd3	158	199	155	85
Hdlbp	7831	12311	9930	14190
Heatr3	877	719	739	671
Heatr4	267	163	171	177
Heatr5a	726	1072	871	1276
Heatr5b	308	428	448	275
Heatr6	112	124	87	72
Hebp1	828	949	883	1281
Hebp2	481	255	484	279
Hectd1	2952	2371	2099	2990
Hectd2	102	36	117	226
Hectd3	1009	1066	895	1038
Hectd4	622	579	543	395
Hecw2	39	119	137	176
Heg1	1886	3903	3318	2003
Helb	370	298	305	475
Helq	237	130	98	164
Helz	206	302	230	202
Helz2	378	409	331	317
Hemk1	171	255	221	187

Henmt1	26	0	14	0
Hepacam	8	38	33	0
Heph	909	555	992	710
Hephl1	7	0	14	0
Herc1	780	826	661	808
Herc2	996	1005	1324	1111
Herc3	296	154	184	308
Herc4	1160	849	722	911
Herc6	64	75	83	30
Herpud1	2028	2171	1592	2112
Herpud2	701	760	703	1112
Hes1	398	434	697	425
Hes3	415	434	231	499
Hes6	741	1312	1038	882
Hes7	16	37	14	17
Hexa	3111	4441	4214	7044
Hexb	2550	2645	3055	5396
Hexdc	2361	939	1478	1230
Hexim1	778	620	638	594
Hexim2	197	200	146	100
Hey1	0	12	12	0
Hey2	12	85	53	31
Heyl	330	1809	504	620
Hfe	144	139	98	197
Hfe2	4603	5268	2773	5605
Hgf	49	25	42	31
Hgfac	32	0	3	0
Hgh1	942	748	856	997
Hgs	1217	1782	1256	1201
Hgsnat	1990	1980	1953	2218
Hhat	56	33	147	27
Hhatl	1917	1973	901	1036
Hhex	216	417	430	588
Hhip	4	0	12	0
Hhipl2	0	45	8	12
Hibadh	1836	1057	1446	2370
Hibch	477	493	430	673
Hic1	81	163	149	245
Hid1	106	186	169	128
Hif1a	438	1180	1086	1208
Hif1an	1304	1353	1380	1551
Higd1a	5774	5239	3519	3450
Higd1b	185	301	140	281
Higd1c	1318	1290	1411	1532
Higd2a	1905	1545	1748	977
Hikeshi	1260	806	909	1157
Hilpda	1761	1994	1407	2130

Hinfp	419	403	426	372
Hint1	243	327	302	271
Hint2	1173	1076	892	884
Hint3	454	530	330	520
Hip1	627	1352	845	1110
Hip1r	396	529	481	242
Hipk1	1071	1434	1717	1476
Hipk2	322	317	348	284
Hipk4	35	102	32	47
Hira	53	9	27	39
Hirip3	698	910	731	689
Hist1h1a	1	10	14	4
Hist1h1b	4	20	14	6
Hist1h1c	3965	3513	3474	4229
Hist1h1d	7	48	24	29
Hist1h2ah	0	17	2	4
Hist1h2ao	18	49	23	30
Hist1h2bc	13	0	5	7
Hist1h2bd	15	0	11	0
Hist1h2bh	3123	1800	2039	1878
Hist1h3a	15	19	15	10
Hist1h4b	507	398	527	248
Hist2h2be	290	94	155	173
Hist2h3c2	0	1	2	0
Hist3h2a	510	243	302	250
Hist3h2ba	32	32	9	42
Hivep1	127	184	191	206
Hivep2	675	843	924	1180
Hivep3	162	140	131	161
Hjurp	319	507	394	265
Hk1	1093	1261	1348	1449
Hk2	2672	1992	2344	2499
Hk3	353	487	313	1456
Hlcs	832	802	635	616
Hlf	151	79	83	96
Hltf	484	509	648	890
Hlx	309	633	339	614
Hm13	3032	4065	3233	4414
Hmbox1	497	676	656	492
Hmbs	1301	1377	1017	1130
Hmces	313	368	453	358
Hmcn1	195	391	296	230
Hmcn2	294	267	132	37
Hmg1l1	8	2	0	35
Hmg20a	1280	1096	1193	1246
Hmg20b	772	991	817	686
Hmga1	4185	3147	3257	1968

Hmgb2	4	19	15	18
Hmgcl	721	854	736	718
Hmgcll1	33	14	20	14
Hmgcr	6296	3752	3748	4644
Hmgcs1	5357	2702	2958	3483
Hmgcs2	498	289	632	218
Hmgn1	3	4	5	0
Hmgn2	4279	3568	2859	2774
Hmgn3	618	365	469	559
Hmgn5b	8	51	51	59
Hmgxb3	74	186	136	115
Hmgxb4	378	469	555	459
Hmmr	276	376	278	258
Hmox1	1881	12482	3882	9352
Hmox2	2827	2626	2747	2521
Hn1	3278	4418	3527	3583
Hn1l	907	1143	928	1330
Hnf4a	0	18	9	0
Hnrnpa1	6004	3014	2666	3582
Hnrnpa2b1	6315	6453	9557	9214
Hnrnpa3	9053	5458	7031	7920
Hnrnpc	6	11	6	0
Hnrnpd	4581	4710	5398	5207
Hnrnpdl	5802	4221	5108	4873
Hnrnpf	3548	4539	3128	3102
Hnrnph1	2043	2143	2862	2319
Hnrnph2	982	1132	1438	1189
Hnrnph3	14302	10233	9546	8822
Hnrnpk	4459	3596	5341	5381
Hnrnpl	11463	9846	13326	10176
Hnrnpll	1123	806	1091	1245
Hnrnpm	1876	2132	2510	2064
Hnrnpr	5	17	15	23
Hnrnpu	9793	10632	13021	11332
Hnrnpul1	4519	3456	4733	4853
Hnrnpul2	875	1740	1732	1660
Hoga1	583	413	367	672
Homer1	242	309	298	428
Homer2	802	807	596	245
Homer3	688	816	603	802
Homez	151	120	143	115
Hook1	219	112	220	130
Hook2	562	274	334	287
Hook3	256	217	296	400
Hopx	7109	4466	5332	6056
Hormad2	17	10	20	48
Hoxb2	107	119	63	31

Hoxb3	5	14	3	0
Hoxb4	45	67	30	41
Hoxb5	3	0	29	0
Hoxb5os	90	30	71	85
Hoxb7	208	126	486	260
Hoxb8	17	35	56	53
Hoxb9	16	42	26	38
Hoxc10	61	10	218	182
Hoxc13	1284	326	1739	429
Hoxc4	170	253	143	35
Hoxc5	314	139	138	72
Hoxc6	245	217	322	158
Hoxc8	684	494	642	593
Hoxc9	154	149	147	242
Hoxd10	124	97	71	85
Hoxd3	47	89	75	70
Hoxd8	1008	862	787	1059
Hoxd9	617	555	462	590
Hp	73	71	145	143
Hp1bp3	3164	2772	3102	3376
Hpcal1	877	1371	1070	1873
Hpd	73	6	84	8
Hpdl	25	37	44	50
Hpf1	1085	787	873	1147
Hpgd	470	183	202	241
Hpgds	655	341	467	268
Hpn	40	25	11	5
Hps1	543	748	515	632
Hps3	318	218	395	537
Hps4	420	284	382	341
Hps5	153	359	254	442
Hps6	183	227	196	287
Hpse	423	1117	919	1124
Hpse2	47	18	21	20
Hpx	30	58	50	53
Hr	9893	4922	10511	3474
Hras	2116	1781	1628	1045
Hrasls	500	529	215	210
Hrasls5	30	72	50	128
Hrc	8525	7297	4423	7600
Hrct1	310	431	481	259
Hrh1	38	69	122	112
Hrh3	5	2	3	2
Hrnr	56	84	5	4
Hs1bp3	435	532	450	670
Hs2st1	1253	1579	1533	2291
Hs3st1	82	74	86	110

Hs3st5	68	48	11	33
Hs3st6	153	80	60	38
Hs6st1	1510	956	1979	1373
Hs6st2	60	46	47	47
Hsbp1	8460	6925	7873	8541
Hsbp1l1	140	56	93	59
Hscb	781	734	776	925
Hsd11b1	2287	1382	2647	3099
Hsd11b2	38	168	122	20
Hsd17b1	71	57	65	86
Hsd17b10	3596	2667	2817	3164
Hsd17b11	1546	1760	2225	3391
Hsd17b12	5453	3665	3626	4137
Hsd17b13	37	41	111	101
Hsd17b14	809	131	412	299
Hsd17b2	33	41	48	7
Hsd17b4	2125	2067	2051	2547
Hsd17b7	574	360	372	569
Hsd17b8	857	815	793	740
Hsd3b3	57	18	24	81
Hsd3b7	1613	1633	1602	1546
Hsdl1	946	955	1201	1459
Hsdl2	1517	980	1070	993
Hsf1	960	1053	734	691
Hsf2	273	226	250	430
Hsf2bp	33	190	42	20
Hsf5	22	6	11	10
Hsh2d	49	60	26	25
Hsp90ab1	27603	38437	31160	25836
Hsp90b1	12799	17508	18589	19862
Hspa12a	56	134	98	142
Hspa12b	87	517	149	185
Hspa13	34	14	56	44
Hspa14	663	930	988	777
Hspa1l	262	216	158	254
Hspa2	1017	215	855	437
Hspa4	5547	5505	6221	6178
Hspa4l	285	114	201	84
Hspa5	14137	18209	18779	24142
Hspa9	338	1010	846	646
Hspb1	9472	11115	6883	3707
Hspb11	324	132	199	278
Hspb2	1097	1860	967	562
Hspb3	1025	700	834	509
Hspb6	42875	26919	16441	36559
Hspb7	8072	10178	5680	5753
Hspb8	10298	20712	11381	9379

Hspbap1	283	221	274	225
Hspbp1	569	684	518	494
Hspd1	6187	4536	4935	4845
Hspe1	2739	3627	3120	2544
Hspg2	3666	11386	5579	6771
Hsph1	1597	1854	1407	1517
Htatip2	1378	1373	1028	1122
Htatsf1	1255	1732	2167	1555
Htr2b	180	346	259	601
Htr6	24	29	17	5
Htra1	12078	21955	14684	25364
Htra2	1325	1633	1316	1962
Htra3	2560	7154	2618	1475
Htra4	1096	968	1001	1423
Htt	1073	1035	862	947
Hus1	478	259	349	244
Huwe1	4211	3967	4136	3865
Hvcn1	234	452	395	1846
Hyal1	1543	1734	1851	1575
Hyal2	1224	1588	1422	1045
Hydin	33	10	65	7
Hyi	953	669	841	520
Hykk	39	27	18	43
Hyls1	213	283	126	161
Hyou1	1887	2764	1914	2424
Hypk	3724	4414	3913	3890
Iah1	1611	1530	1501	1976
Iars	878	1200	873	996
Iars2	1537	2003	1615	1809
Iba57	506	384	481	349
Ibsp	31	65	75	42
Ibtk	1081	1066	964	1347
Ica1	320	309	459	241
Icam1	2603	1928	2349	2384
Icam2	506	1106	624	1077
Icam5	4	1	8	39
Ice1	405	514	692	641
Ice2	375	154	238	233
Ick	459	739	543	664
Icmt	277	335	337	458
Icos	326	202	438	365
Icoslg	2907	1344	2693	1199
Id1	1257	2197	1348	1374
Id2	189	390	408	660
Id3	612	1785	800	1203
Id4	1265	316	582	441
Ide	3223	2688	2451	2648

Idh1	4836	3086	5322	3955
Idh2	3501	6377	3557	5583
Idh3a	1842	2419	1603	2248
Idh3B	5519	3934	3615	4411
Idh3g	4556	3852	3211	3848
Idi1	2898	1448	2254	3314
Idnk	268	164	265	267
Ido2	3	0	3	3
Ids	998	1258	1295	1575
Idua	1626	1254	1675	1492
Ier2	1585	1017	847	759
Ier3	2418	2462	2149	986
Ier5	1897	1729	1936	1934
Ier5l	292	582	337	567
Iffo1	1867	3365	1905	1785
Iffo2	13232	3167	11691	3096
Ifi27	10630	13941	11095	13182
Ifi27l2b	1278	1861	1320	1599
Ifi30	7975	8133	8060	15007
Ifi35	8943	5574	7136	9005
Ifi44	802	810	794	1175
Ifi44l	295	175	247	405
Ifi47	1279	1006	1041	1345
Ifih1	247	269	293	307
Ifit1bl	1539	2277	2120	6170
Ifit2	311	470	444	534
Ifit3	964	1222	501	346
Ifitm1	7420	6907	7761	22661
Ifitm10	1644	807	883	504
Ifitm2	7419	7991	6054	9681
Ifitm3	14897	13222	11605	11969
Ifitm5	75	156	138	104
Ifnar1	2892	3216	4413	6157
Ifnar2	820	1106	731	1085
Ifngr1	2227	3556	2905	4996
Ifngr2	1232	1394	1563	2168
Ifnlr1	292	313	400	509
Ifrd1	1379	1509	1685	1846
Ifrd2	744	828	368	479
Ift122	209	309	351	255
Ift140	157	279	178	222
Ift172	389	342	319	453
Ift20	2078	2305	2317	2138
Ift22	248	333	178	317
Ift27	598	634	644	712
Ift43	557	585	474	497
Ift46	860	998	826	909

Ift52	1392	1314	1443	1148
Ift57	734	688	1035	744
Ift74	794	617	600	572
Ift80	83	192	156	154
Ift81	337	397	412	293
Ift88	333	188	256	250
Igbp1	1953	1546	1613	1773
Igdcc3	10	0	8	7
Igdcc4	731	852	752	1271
Igf1	1158	1376	1327	2224
Igf1r	694	1097	988	664
Igf2	1840	14816	3206	1650
Igf2bp2	249	233	426	107
Igf2bp3	41	10	11	39
Igf2r	2318	4405	2510	3738
Igfals	0	9	5	0
Igfbp2	1296	1073	826	474
Igfbp3	3177	5704	4452	2479
Igfbp4	14483	12018	23832	14015
Igfbp5	5889	19314	7258	4877
Igfbp6	2757	10869	3295	1787
Igfbp7	21117	25657	25308	21395
Igflr1	21	18	3	11
Igfn1	734	1318	2874	242
Ighmbp2	64	79	38	47
Igip	363	400	323	261
Iglon5	37	136	21	100
Igsf1	63	51	32	89
Igsf10	726	838	1131	3089
Igsf11	20	5	12	29
Igsf23	124	85	54	182
Igsf3	2921	1651	3307	3763
Igsf6	779	465	739	1168
Igsf7	1168	2873	2009	7466
Igsf8	1281	1067	1329	1068
Igsf9	30883	39496	29675	36800
Igsf9b	4	12	6	10
Igtp	1256	925	1029	1143
Ik	2215	2586	3400	3585
Ikbip	1293	2539	1966	2979
Ikbkap	970	799	1049	819
Ikbkb	1266	1064	1107	1286
Ikbke	964	473	672	777
Ikbkg	573	476	582	557
Ikzf1	35	15	84	131
Ikzf2	42	26	32	45
Ikzf5	462	331	304	470

Il10	144	169	72	63
Il10ra	520	576	682	1113
Il10rb	949	1530	1493	2030
Il11	62	15	57	21
Il11ra1	3803	2123	2333	2451
Il12b	172	39	65	106
Il12rb1	58	25	35	37
Il12rb2	204	168	149	145
Il13ra1	128	116	48	158
Il13ra2	105	101	29	1
Il15	394	342	409	292
Il15ra	112	169	182	82
Il16	37	242	92	116
Il17b	30	55	71	4
Il17d	86	106	101	126
Il17ra	1352	2159	1769	2670
Il17rb	95	32	101	107
Il17rc	277	393	187	169
Il17rd	472	208	269	377
Il17re	1003	495	670	370
Il18	870	578	560	1475
Il18bp	1448	1729	1561	4559
Il18r1	661	2173	1787	1696
Il1a	556	789	272	134
Il1b	4381	3880	2474	854
Il1f10	23	0	12	0
Il1r1	1025	1295	1729	2363
Il1r2	1686	1330	1019	897
Il1rap	692	608	468	629
Il1rl2	44	57	56	75
Il1rn	6251	3947	6257	11957
Il20	108	106	39	47
Il20ra	37	39	12	0
Il20rb	2273	2041	1447	1181
Il21r	257	158	259	400
Il22ra1	4	17	9	0
Il22ra2	363	61	113	192
Il23a	128	466	214	84
Il24	365	355	596	235
Il27ra	22	0	0	16
Il2rb	340	72	478	405
Il31ra	28	81	45	50
Il33	1531	1490	2856	5022
Il34	1372	1513	1725	1441
Il36b	207	58	65	260
Il36g	707	147	99	70
Il36rn	722	145	275	62

Il3ra	242	153	237	230
Il4i1	1352	1663	1420	1753
Il4r	1909	4168	2264	2823
Il6	91	73	116	50
Il6r	920	1069	963	1292
Il6st	6093	5613	7539	6845
Il7	3	84	26	50
Il7r	469	342	325	846
Il9r	26	11	18	24
Ildr1	21	24	18	1
Ilf2	2152	2117	2561	1932
Ilf3	964	1023	1088	1041
Ilk	5647	6878	6828	8718
Ilkap	1092	1257	808	1067
Ilvbl	1117	1263	1501	1901
Immp1l	847	613	621	850
Immp2l	179	149	159	128
Immt	5090	4478	4291	4466
Imp3	1451	1054	1228	989
Imp4	1478	1577	1335	1306
Impa1	828	625	998	1244
Impa2	1028	654	808	746
Impact	814	633	1044	817
Impdh1	701	2028	1211	1532
Impdh2	9709	7860	8131	7070
Ina	8	0	5	0
Inafm2	583	858	880	1048
Inca1	25	19	17	0
Incenp	851	1141	979	832
Inf2	2942	2541	2782	2286
Ing1	596	629	785	715
Ing2	952	935	1410	1315
Ing3	168	207	326	311
Ing4	2089	1700	1938	2062
Ing5	493	458	503	400
Inha	50	106	86	55
Inhba	683	474	608	556
Inhbb	1353	702	1529	689
Inip	1165	757	1016	1003
Inmt	40	22	65	21
Ino80	641	534	542	539
Ino80c	1445	1384	1175	1324
Ino80d	367	348	575	627
Ino80e	1053	1093	949	906
Inpp1	376	430	330	349
Inpp4a	368	508	381	293
Inpp4b	94	220	96	118

Inpp5a	774	852	779	815
Inpp5d	638	1156	773	1452
Inpp5e	158	144	25	145
Inpp5f	1298	916	1216	603
Inpp5k	1383	1399	1616	1190
Inppl1	956	1976	1345	1231
Insc	10	14	44	23
Insig1	1865	1390	1768	1749
Insig2	1263	724	1035	830
Insm1	18	37	0	21
Insr	640	762	767	649
Insrr	17	3	12	26
Ints1	685	886	567	654
Ints10	814	842	823	725
Ints12	350	231	364	375
Ints2	85	96	129	125
Ints3	867	1088	838	826
Ints4	1031	1244	967	998
Ints5	666	703	682	651
Ints6	397	360	366	605
Ints6l	320	332	223	223
Ints7	84	192	187	205
Ints8	876	623	762	954
Intu	0	10	6	0
Invs	153	121	146	153
Ip6k1	1698	1674	2096	1870
Ip6k2	1010	992	1041	745
Ip6k3	364	634	339	281
Ipcef1	173	78	93	181
Ipmk	835	701	995	1347
Ipo11	389	393	277	293
Ipo13	1515	1347	1303	1077
Ipo4	1062	2012	1112	1245
Ipo5	4857	5434	4884	4809
Ipo7	2388	1702	1891	2040
Ipo9	1500	2072	1651	1705
Ipp	486	322	463	418
Ippk	477	304	420	259
Iqcb1	240	142	284	204
Iqcc	221	217	184	141
Iqce	255	334	281	275
Iqcg	8	12	6	5
Iqck	297	438	665	721
Iqgap1	10251	9009	10272	11650
Iqgap2	135	215	260	200
Iqgap3	392	623	465	652
Iqsec1	1369	1688	1661	2200

Iqsec2	419	322	185	226
Iqub	29	32	18	0
Irak1	2886	3348	3107	3571
Irak1bp1	146	125	90	87
Irak2	273	287	254	317
Irak3	762	1285	667	1103
Irak4	531	456	674	798
Ireb2	1205	799	1190	1291
Irf1	1364	955	1198	1086
Irf2	464	607	635	757
Irf2bp1	912	819	692	429
Irf2bp2	590	318	404	469
Irf2bpl	1157	781	952	1123
Irf3	3093	2589	2290	1848
Irf4	235	157	265	261
Irf5	1432	1479	1658	2161
Irf6	6805	2368	5960	1807
Irf7	1050	1159	764	1587
Irf8	1453	1321	1480	2247
Irf9	1820	1519	1324	2141
Irg1	159	40	164	210
Irgm	597	443	620	764
Irgm2	1521	1792	1548	1539
Irgq	1144	966	1068	1130
Irs1	27	80	66	93
Irs2	683	575	719	400
Irs3	48	128	170	95
Irx1	796	381	501	291
Irx2	1668	517	1562	593
Irx3	1284	507	1064	458
Irx4	1514	365	1143	282
Irx5	241	208	292	108
Isca1	3818	3615	2946	3844
Isca2	1240	740	922	1225
Iscu	3633	4030	3295	3407
Isg15	838	937	387	670
Isg20	236	111	107	141
Isg20l2	97	115	134	155
Isl1	32	56	21	103
Islr	1105	1971	865	629
Islr2	25	100	15	24
Ism1	27	39	51	0
Isoc2b	780	683	629	907
Ispd	159	56	156	130
Ist1	3123	2450	3285	2826
Isy1	1213	1654	1235	1438
Isyna1	1017	1062	764	450

Itch	819	715	751	905
Itfg1	1906	1763	1550	1754
Itfg2	342	266	254	261
Itga1	50	55	74	37
Itga10	54	18	39	62
Itga11	309	432	387	886
Itga2	474	370	486	304
Itga3	1629	2488	1887	871
Itga4	118	96	128	613
Itga5	1537	4798	2225	3897
Itga6	4236	5411	4801	3210
Itga7	1379	4181	1356	936
Itga8	203	419	245	506
Itga9	431	1268	626	765
Itgad	779	953	1028	1909
Itgae	750	530	569	685
Itgal	119	182	166	358
Itgam	618	1926	1353	4704
Itgav	2803	2157	2138	3345
Itgb1	9418	14358	12477	17742
Itgb1bp1	1573	2164	1496	1689
Itgb1bp2	1300	4674	900	1165
Itgb2	1115	2702	2892	9314
Itgb3	3	56	18	32
Itgb3bp	563	369	444	449
Itgb4	5983	6279	4854	2083
Itgb5	3759	3724	4297	3287
Itgb6	1244	988	1217	603
Itgb7	563	1568	703	1878
Itgb8	370	135	226	121
Itgbl1	1104	1119	1485	1601
Itih3	54	74	53	56
Itk	120	40	47	56
Itm2a	2694	3753	1780	4126
Itm2b	26808	21037	21950	27701
Itm2c	3522	6781	5252	7410
Itpa	3194	2856	2723	3026
Itpk1	794	599	686	652
Itpka	9	23	39	19
Itpkb	1675	1240	1920	1731
Itpkc	1342	890	1229	609
Itpr1	251	164	132	221
Itpr2	1040	808	1064	1052
Itpr3	1425	1472	1091	838
Itpripl1	8	62	48	22
Itpripl2	101	50	90	82
Itsn1	76	308	193	197

Itsn2	1361	1179	2021	1944
Ivd	11744	3027	4900	10812
Ivl	215	62	120	68
Ivns1abp	3171	2294	2316	2283
Iws1	577	629	704	760
Izumo1	7	0	0	19
Izumo1r	1	0	0	4
Izumo4	1823	731	1204	1174
Jade1	341	380	420	229
Jade2	14	43	42	98
Jag1	4058	1436	3426	1379
Jag2	820	1019	650	344
Jagn1	2132	2141	1840	2315
Jak1	4304	4861	5242	6175
Jak2	2098	2189	2294	3950
Jak3	419	588	513	554
Jam2	783	785	891	681
Jam3	259	688	403	389
Jaml	11	51	11	56
Jarid2	114	172	199	148
Jazf1	285	190	284	278
Jdp2	337	569	433	872
Jkamp	138	213	178	276
Jmjd1c	924	803	1124	725
Jmjd6	572	882	487	356
Jmjd7	44	51	65	19
Jmjd8	1080	1734	885	1155
Jmy	780	589	697	400
Josd1	971	902	948	1346
Josd2	352	441	266	280
Jph1	680	650	391	628
Jph2	2289	2739	1092	2403
Jpx	151	45	84	16
Jrk	201	270	239	289
Jrkl	35	69	60	56
Jsrp1	1286	1954	1089	755
Jtb	5186	4713	4855	5376
Jun	3157	2312	2460	2339
Junb	821	1227	674	515
Jund	6093	2406	3578	3204
Jup	16438	10320	14838	5141
Kalrn	113	232	207	234
Kank1	1621	1504	1333	639
Kank2	1397	3269	2445	2154
Kank3	274	467	369	382
Kank4	142	55	247	188
Kansl1	307	545	558	592

Kansl1l	293	231	278	328
Kansl3	1026	1393	1477	1251
Kap	498	15	501	81
Kars	4479	5572	4839	5239
Kat14	396	617	582	695
Kat2b	90	112	119	130
Kat5	1099	1135	1078	1390
Kat6a	1302	1121	1119	1114
Kat6b	370	220	405	220
Kat7	1956	1863	2156	1887
Kat8	1062	995	879	868
Katna1	690	417	642	491
Katnb1	220	366	326	331
Kazald1	399	522	308	472
Kazn	473	464	480	259
Kb15	53	39	87	11
Kb23	1215	127	1074	18
Kbtbd11	15	63	30	44
Kbtbd12	539	194	218	335
Kbtbd2	849	791	1047	689
Kbtbd3	233	129	128	119
Kbtbd4	379	376	330	442
Kbtbd7	41	44	24	15
Kbtbd8	81	63	66	79
Kcmf1	3264	2826	2866	3291
Kcna2	11	10	2	16
Kcna5	0	5	2	0
Kcna7	480	436	123	436
Kcnab1	138	253	180	186
Kcnab2	187	464	239	457
Kcnb1	5	37	98	20
Kcnc1	15	5	0	14
Kcnc4	939	587	376	407
Kcnd1	140	221	361	228
Kcnd3	33	18	11	19
Kcne1	11	0	29	0
Kcne2	21	23	11	0
Kcne3	178	900	328	419
Kcne4	228	255	387	276
Kcne5	69	50	29	7
Kcng1	0	13	3	0
Kcng2	36	79	56	68
Kcng4	0	11	15	0
Kcnh1	101	108	137	179
Kcnh2	3	37	15	10
Kcnh4	4242	5003	3292	5223
Kcnip1	15	10	56	0

Kcnip3	1333	638	730	544
Kcnip4	6	0	0	16
Kcnj11	1097	516	469	538
Kcnj12	540	469	232	192
Kcnj14	71	74	63	73
Kcnj15	242	279	218	193
Kcnj2	408	250	278	288
Kcnj4	3	0	5	0
Kcnj6	7	33	14	0
Kcnj8	264	461	554	706
Kcnj9	3	3	5	0
Kcnk1	496	201	278	287
Kcnk13	90	265	251	177
Kcnk2	301	104	468	237
Kcnk4	109	20	18	22
Kcnk5	32	38	72	10
Kcnk7	2033	1448	1474	1028
Kcnma1	45	93	36	34
Kcnmb1	53	248	54	100
Kcnmb4	122	137	93	68
Kcnn1	106	101	60	68
Kcnn3	324	421	399	203
Kcnn4	408	622	527	1945
Kcnq1	63	49	57	122
Kcnq4	122	411	173	133
Kcnq5	140	246	230	43
Kcnrg	184	70	175	115
Kcns3	77	216	172	290
Kcnt1	52	37	24	1
Kcnt2	12	9	0	12
Kcp	9	72	24	16
Kctd1	1138	719	1131	629
Kctd10	1423	1994	1968	2204
Kctd11	872	725	922	925
Kctd12	4057	2633	4076	7098
Kctd13	185	153	155	336
Kctd14	221	525	345	350
Kctd15	2091	1213	1933	1156
Kctd16	19	7	3	21
Kctd17	1023	998	897	1082
Kctd18	605	981	833	716
Kctd2	496	484	459	365
Kctd20	1226	1145	1338	1196
Kctd21	117	145	164	179
Kctd3	572	597	794	724
Kctd5	1526	1527	1619	1400
Kctd6	242	223	220	284

Kctd7	3	22	0	10
Kctd9	1034	1119	1219	1040
Kdelc1	408	655	549	498
Kdelc2	725	1469	1487	2063
Kdelr1	6395	8631	9003	8596
Kdelr2	1887	3887	2362	3948
Kdelr3	1529	3849	2896	5214
Kdm1a	1701	2005	1941	1783
Kdm1b	62	89	33	25
Kdm2a	1529	1588	2057	1694
Kdm2b	1399	715	840	882
Kdm3a	1001	836	1140	1237
Kdm3b	729	691	909	772
Kdm4a	1210	1147	1577	1118
Kdm4b	521	584	381	584
Kdm4c	69	77	113	30
Kdm5a	1171	671	1219	1017
Kdm5b	2202	1274	2001	1824
Kdm5c	1163	934	1103	1043
Kdm5d	0	158	0	228
Kdm6b	1140	886	1095	681
Kdm8	675	537	594	613
Kdr	359	1473	895	961
Kdsr	1410	1186	1493	1867
Keap1	1191	1234	996	975
Kera	6	15	3	63
Khdrbs1	3938	2660	2898	3893
Khdrbs3	303	384	268	265
Khk	114	145	96	101
Khnyn	346	412	229	224
Khsrp	1277	1555	1202	997
Kiaa0895l	8	30	15	122
Kidins220	1985	1776	2060	2847
Kif11	322	514	554	357
Kif13a	1504	1296	1428	1138
Kif13b	821	839	629	516
Kif14	88	101	98	67
Kif15	266	293	486	326
Kif16b	316	273	354	262
Kif18a	35	107	93	79
Kif18b	227	355	193	301
Kif19	0	8	8	0
Kif1b	4531	3781	3092	3823
Kif1bp	496	624	647	595
Kif1c	8065	6791	6400	6265
Kif20a	524	682	653	572
Kif20b	286	177	220	210

Kif21a	614	156	603	218
Kif21b	187	245	290	253
Kif22	823	1624	1316	1054
Kif23	293	628	606	358
Kif26a	73	137	74	44
Kif26b	338	88	381	761
Kif27	2	33	26	0
Kif2a	435	536	522	874
Kif2c	214	522	320	256
Kif3a	547	444	748	652
Kif3b	576	824	825	775
Kif3c	413	725	665	350
Kif4a	336	489	418	460
Kif5a	128	198	56	183
Kif5b	2051	2360	2158	1781
Kif5c	15	14	22	28
Kif7	358	329	445	295
Kif9	12	31	21	39
Kifap3	1860	1398	1577	1707
Kifc1	360	802	471	552
Kifc2	159	97	102	63
Kifc3	362	458	299	273
Kin	351	360	442	427
Kirrel	1652	1886	2284	2261
Kirrel3	40	31	23	11
Kit	379	292	543	417
Kitlg	513	556	533	461
Kiz	222	259	275	271
Klb	13	12	8	10
Klc1	2476	6139	2964	2924
Klc2	821	846	898	851
Klc3	486	273	353	68
Klc4	327	460	317	229
Klf1	29	10	27	0
Klf10	2496	1169	1854	1322
Klf11	224	275	436	278
Klf12	219	152	208	118
Klf13	44	35	36	52
Klf14	5	0	11	1
Klf15	158	104	60	101
Klf16	1275	1042	1202	838
Klf2	1104	1119	1944	1079
Klf3	877	673	799	985
Klf4	4189	3312	5168	2723
Klf5	1418	557	748	337
Klf6	4295	2013	3948	4229
Klf7	138	256	150	315

Klf9	2204	1947	1592	1940
Klhdc1	81	90	132	70
Klhdc10	1698	1663	2079	1863
Klhdc2	1228	728	909	856
Klhdc3	1553	1579	1259	1817
Klhdc4	556	890	612	710
Klhdc7a	0	7	8	0
Klhdc8a	63	0	38	0
Klhdc8b	172	200	135	207
Klhdc9	163	185	155	101
Klhl11	26	38	29	37
Klhl12	414	444	587	350
Klhl13	289	847	328	565
Klhl15	14	27	21	48
Klhl17	198	161	98	114
Klhl18	726	909	876	856
Klhl2	648	871	1022	639
Klhl20	457	499	483	431
Klhl21	1437	1480	1213	1178
Klhl22	949	1400	1004	1372
Klhl23	178	264	194	297
Klhl24	509	668	698	721
Klhl25	236	168	122	237
Klhl26	235	377	293	190
Klhl28	233	230	343	278
Klhl29	318	265	196	111
Klhl3	9	7	15	15
Klhl30	967	1813	450	786
Klhl31	1935	2553	1217	2185
Klhl33	451	384	334	261
Klhl34	78	83	37	0
Klhl35	79	33	51	44
Klhl36	44	121	59	18
Klhl38	297	232	172	277
Klhl4	0	8	24	0
Klhl40	622	1395	590	380
Klhl41	11353	11942	13932	9673
Klhl42	33	31	45	12
Klhl5	61	133	150	202
Klhl6	239	233	298	544
Klhl7	533	469	683	518
Klhl8	1139	1028	958	547
Klhl9	728	789	790	1097
Klk1	340	2891	1032	1125
Klk10	1352	263	752	178
Klk11	267	111	159	30
Klk13	599	153	51	17

Klk1b3	4	3	2	29
Klk5	82	59	39	29
Klk6	5011	1240	1342	377
Klk7	2871	1072	862	288
Klk8	512	221	209	103
Klk9	156	59	45	38
Klkb1	175	227	244	423
Klra1	45	34	35	21
Klra5	58	25	42	270
Klrb1a	211	44	116	112
Klrb1b	267	102	182	153
Klrb1c	7	29	15	26
Klrc3	150	61	111	116
Klrd1	179	0	54	110
Klre1	43	12	3	3
Klrg1	14	1	21	12
Klrg2	807	372	423	272
Klri1	77	9	50	89
Klrk1	468	194	262	405
Kmo	63	92	69	100
Kmt2a	856	680	883	456
Kmt2b	567	629	626	430
Kmt2d	1471	1176	1711	1026
Kmt2e	349	380	337	205
Kmt5a	1950	2424	1459	1608
Kmt5b	525	551	537	633
Kmt5c	551	381	576	426
Kng1	266	114	287	41
Kng1l1	299	217	436	99
Knl1	39	30	53	112
Knop1	9	58	20	13
Knstrn	490	685	555	497
Kntc1	233	235	236	238
Kpna1	1885	1207	1593	1636
Kpna2	39	200	190	142
Kpna3	1488	1124	1506	1784
Kpna4	942	871	1001	1295
Kpna5	367	180	224	161
Kpna6	353	386	277	364
Kpnb1	4833	5753	5266	5430
Kprp	254	47	32	1
Kptn	567	550	396	393
Kras	1348	1030	1399	1253
Krba1	120	137	111	111
Krcc1	1285	1577	1846	2048
Kremen1	878	2590	1452	1823
Kremen2	41	17	29	0

Kri1	259	599	348	463
Krit1	371	374	439	479
Krr1	340	516	469	487
Krt1	3699	2909	587	569
Krt10	4124	2954	617	895
Krt14	150862	100948	121254	34009
Krt15	47388	32995	25851	16499
Krt18	11	7	14	0
Krt19	42	34	51	41
Krt2	33	7	26	0
Krt23	301	59	239	55
Krt24	302	520	78	50
Krt25	4621	818	10475	45
Krt31	1121	64	713	16
Krt32	151	57	107	8
Krt34	1	5	5	0
Krt5	309070	147005	285840	66700
Krt7	71	38	30	18
Krt71	11952	2530	15742	1293
Krt72	20	10	137	0
Krt73	672	212	1277	0
Krt75	1800	1300	2874	643
Krt78	178	42	18	11
Krt79	15265	4300	7559	4823
Krt8	878	384	554	390
Krt80	1395	671	695	279
Krt84	12	0	143	1
Krt86	118	1	248	23
Krtap11-1	182	0	250	15
Krtap1-3	41	0	131	0
Krtap13-1	283	7	597	0
Krtap14	55	0	128	0
Krtap15-1	129	13	105	0
Krtap22-2	85	9	86	0
Krtap2-4	47	36	131	0
Krtap3-1	104	3	272	11
Krtap3-2	44	4	184	0
Krtap3-3	164	0	333	0
Krtap7-1	161	70	441	0
Krtap8-1	37	0	86	0
Krtcap2	3860	3766	3272	5002
Krtcap3	589	402	482	270
Krtdap	11830	1988	6423	1198
Ksr2	54	3	8	0
Kti12	15	56	44	14
Ktn1	1813	1808	2522	1640
Kxd1	1951	1867	1681	1817

Ky	2063	474	463	1662
Kyat1	1113	1418	539	544
Kyat3	672	204	357	322
Kynu	38	0	20	10
L1cam	185	106	143	85
L2hgdh	171	160	107	134
L3hypdh	83	84	66	89
L3mbtl2	627	766	591	434
L3mbtl3	22	35	33	29
Lacc1	380	290	379	518
Lace1	164	142	78	253
Lactb	860	853	1157	1041
Lactb2	1552	1032	906	1312
Lad1	3113	2218	2141	1049
Lag3	29	17	26	25
Lage3	2379	2614	2073	2079
Lair1	15	0	15	0
Lama1	56	0	71	0
Lama2	1619	2499	1520	1736
Lama3	3424	4584	3333	2026
Lama4	2005	6653	3116	3313
Lama5	1541	1675	2041	1159
Lamb1	5023	9664	8093	7857
Lamb2	1949	3844	1879	1590
Lamb3	6078	6449	4982	2311
Lamc1	4411	8786	5773	5128
Lamc2	6489	10828	7093	4182
Lamc3	90	79	134	107
Lamp1	8016	11359	8489	13240
Lamp2	5584	4160	5537	8590
Lamtor1	243	259	132	209
Lamtor2	1385	1882	1550	1477
Lamtor3	1480	1399	1392	1643
Lamtor4	2367	2794	2410	2749
Lamtor5	3470	2627	2749	3657
Lancl1	1551	1301	1076	922
Lancl2	799	474	651	769
Lap3	3719	3488	3746	3798
Laptm4a	16451	12386	17012	20027
Laptm4b	2761	2815	2600	3024
Laptm5	4763	5542	5824	12613
Large1	1124	997	1097	1166
Large2	371	214	439	150
Larp1	2271	5574	3948	6014
Larp1b	1295	1129	1159	1312
Larp4	13	47	44	0
Larp4b	1973	2464	1918	1689

Larp6	94	113	110	54
Larp7	840	991	1231	697
Lars	2320	2451	2928	2354
Lars2	562	467	484	374
Las1l	1823	1482	1875	1658
Lasp1	6240	7743	8077	10080
Lat	288	113	238	151
Lat2	720	724	862	2825
Lats1	368	404	554	322
Lats2	468	416	475	419
Lax1	5	13	21	8
Layn	317	262	388	665
Lbh	5357	2493	5722	3938
Lbhd1	1159	987	924	1028
Lbp	1636	5491	3504	7559
Lbr	68	114	113	80
Lbx1	308	248	132	234
Lca5	25	34	23	0
Lcat	227	187	200	198
Lce1f	123	10	9	0
Lce1l	71	27	11	6
Lce1m	22	19	0	7
Lce6a	84	7	8	3
Lck	6	18	29	21
Lclat1	103	124	116	189
Lcmt1	557	667	706	572
Lcmt2	237	194	278	251
Lcn2	29	71	18	0
Lcn3	31	5	12	36
Lcor	196	279	328	279
Lcorl	10	46	87	78
Lcp1	8564	6674	9510	18224
Lcp2	768	621	793	1633
Ldah	1341	813	512	986
Ldb1	3414	2893	3500	2192
Ldb2	269	234	334	330
Ldb3	13485	9225	3405	11178
Ldha	28512	19785	11149	17699
Ldhb	2325	1820	1583	1830
Ldhc	14	40	5	24
Ldhd	1467	950	1049	1061
Ldlr	4412	4767	4521	3532
Ldlrad1	126	64	128	156
Ldlrad4	422	717	726	558
Ldlrap1	819	1347	1038	1019
Ldoc1l	282	540	355	472
Lect2	31	74	23	57

Lef1	168	99	159	122
Lekr1	22	38	53	17
Lemd3	336	329	364	304
Leng1	327	207	341	230
Leng8	2293	1809	2315	1469
Leng9	551	426	515	758
Leo1	684	975	775	794
Lep	26	16	39	0
Lepr	1480	2396	2249	2586
Leprotl1	1820	1667	1807	1923
Letm1	344	582	509	293
Letm2	193	184	159	125
Letmd1	707	685	739	794
Lexm	18	5	15	13
Lfng	1130	983	1288	907
Lgals1	14128	41775	18303	36852
Lgals3	29348	35035	41663	94909
Lgals3bp	5924	6415	6346	14152
Lgals4	26	9	21	0
Lgals7	60865	25255	41355	13034
Lgals8	220	338	384	252
Lgals9	623	984	584	717
Lgalsl	1972	1059	1091	1345
Lgi2	31	73	6	60
Lgi3	176	201	147	107
Lgi4	743	618	543	248
Lgmn	8959	16097	17452	32959
Lgr4	918	532	1156	350
Lgr5	671	86	709	318
Lgr6	354	244	242	274
Lhb	9	16	6	30
Lhfp	904	1084	797	977
Lhfpl1	61	29	26	3
Lhfpl2	529	759	907	2168
Lhpp	493	399	503	470
Lhx2	480	374	399	290
Lhx3	411	685	450	642
Lhx6	64	121	101	105
Lias	707	580	515	488
Lif	47	13	24	4
Lifr	49	59	89	96
Lig1	9	20	8	19
Lig3	635	298	215	242
Lig4	323	199	203	252
Lilra5	89	98	302	575
Lilrb1	91	544	167	238
Lilrb3	1346	759	1900	6658

Lilrb4	3466	2958	4293	9158
Lilrc2	32	10	54	147
Lima1	3649	4935	3683	3448
Limch1	1065	1297	818	941
Limd1	1122	2328	1745	1763
Limd2	2018	2138	2221	2915
Lime1	812	770	715	727
Limk1	178	459	224	490
Limk2	1503	1248	1581	1075
Lims1	1036	1533	1450	2627
Lims2	204	531	202	244
Lin37	917	728	462	726
Lin52	334	294	357	365
Lin54	366	146	346	315
Lin7b	3	0	15	0
Lin7c	14	71	56	49
Lingo1	33	38	39	29
Lingo4	313	119	120	118
Lins1	159	221	209	210
Lipc	19	0	18	1
Liph	346	94	117	142
Lipm	367	75	143	69
Lipn	45	0	11	0
Lipt1	55	33	33	81
Lipt2	346	357	278	401
Litaf	5349	5854	5225	9148
Lix1	23	21	3	11
Lix1l	1523	2494	2761	4021
Llgl1	69	268	160	274
Llgl2	121	152	101	91
Lman1	1490	2725	2111	3011
Lman2	3042	3846	2895	3489
Lman2l	810	640	772	667
Lmbr1	331	215	287	236
Lmbr1l	318	292	286	349
Lmbrd1	868	453	582	978
Lmbrd2	22	17	18	0
Lmcd1	5667	3193	2290	2385
Lmf1	285	431	381	358
Lmf2	1109	2002	1256	1621
Lmln	94	234	122	96
Lmna	20273	24077	20197	17152
Lmnb1	688	1058	1225	743
Lmnb2	4	101	20	0
Lmntd2	23	0	3	0
Lmo1	754	799	497	401
Lmo2	919	1715	1912	2252

Lmo4	2084	1389	1860	1997
Lmo7	807	824	912	705
Lmod1	318	255	242	241
Lmod3	2358	3709	2109	1890
Lmtk2	690	598	659	773
Lmtk3	2	1	3	0
Lmx1a	31	103	74	117
Lnc001	29	18	11	12
Lnc134	235	255	194	172
Lnc215	215	149	125	86
Lnp	327	338	314	429
Lnp1	134	0	56	19
Lnx1	454	403	478	417
Lnx2	222	259	103	353
LOC100125	871	647	874	1272
LOC100125	298	309	240	114
LOC100134	14	107	20	29
LOC100151	469	441	677	826
LOC100158	239	273	167	103
LOC100174	466	273	250	314
LOC100233	1624	1353	1387	1560
LOC100233	14	50	26	32
LOC100294	1028	1014	1050	1175
LOC100302	988	1357	1580	1434
LOC100302	14	58	24	12
LOC100359	607	1141	609	698
LOC100359	3	31	4	0
LOC100359	12490	7572	12971	12863
LOC100359	229	634	333	180
LOC100359	225	330	116	161
LOC100359	6193	9106	9465	7066
LOC100359	1072	1852	1119	1260
LOC100359	9	11	3	13
LOC100359	364	282	141	187
LOC100359	73	187	93	156
LOC100359	605	431	480	561
LOC100359	790	1519	1214	1038
LOC100359	841	1668	1490	1316
LOC100359	666	559	283	237
LOC100359	459	719	382	452
LOC100359	463	717	280	301
LOC100359	74	35	51	21
LOC100360	9212	8445	5848	5634
LOC100360	75	20	6	0
LOC100360	8418	8616	3328	2820
LOC100360	75	179	134	111
LOC100360	2352	2510	2832	2581

LOC10036(	37	78	47	50
LOC10036(	0	7	0	14
LOC10036(	34	103	18	64
LOC10036(	707	1782	1408	1305
LOC10036(	1136	1157	450	522
LOC10036(	327	176	206	265
LOC10036(	98	200	96	106
LOC10036(	154	192	247	201
LOC10036(	0	8	6	13
LOC10036(	179	40	26	119
LOC10036(	3859	4204	2812	2155
LOC10036(	0	8	3	0
LOC10036(	3984	3442	3590	3247
LOC10036(	1056	676	904	739
LOC10036(	21	24	8	0
LOC10036(	91	158	41	37
LOC10036(	14068	15823	9879	9598
LOC10036(	396	649	387	506
LOC10036(	154	14	18	30
LOC10036(	25935	17837	11487	10060
LOC10036(	55899	26546	36084	47701
LOC10036(	3232	2131	2216	1611
LOC10036(	9	5	44	0
LOC10036(	51	4	24	92
LOC10036(	3835	4170	2484	3420
LOC10036(	156	447	287	252
LOC10036(	14513	16845	10633	10843
LOC100361	16063	9194	15073	11612
LOC100361	10	14	8	0
LOC100361	3112	4043	2881	2388
LOC100361	6198	5338	5341	5626
LOC100361	14	45	60	38
LOC100361	4	1	2	5
LOC100361	1887	1140	12	12
LOC100361	1038	1453	69	31
LOC100361	46	4	17	6
LOC100361	309	573	263	502
LOC100361	2536	2468	1261	1621
LOC100361	28	24	17	24
LOC100361	1	0	9	0
LOC100361	37	73	57	24
LOC100361	78	148	211	19
LOC100361	1996	2493	624	865
LOC100361	4674	3525	3170	2883
LOC100361	416	431	812	948
LOC10036Z	15	145	65	59
LOC10036Z	34329	20305	22946	19950

LOC100362	24	38	140	73
LOC100362	133	93	108	114
LOC100362	27	7	24	36
LOC100362	109	133	162	151
LOC100362	12119	9883	5785	5900
LOC100362	327	291	293	372
LOC100362	7	23	3	0
LOC100362	3989	1302	11	11
LOC100362	151	403	84	122
LOC100362	4	4	12	3
LOC100362	720	913	623	725
LOC100362	844	1173	1095	1155
LOC100362	631	1124	841	639
LOC100362	0	22	2	20
LOC100362	7397	5451	5302	6522
LOC100362	16	85	41	0
LOC100362	1109	1531	555	1414
LOC100362	88	59	80	52
LOC100362	31	20	5	0
LOC100362	260	70	45	50
LOC100362	325	179	265	261
LOC100362	37012	29368	26190	25275
LOC100362	696	1295	630	588
LOC100362	3	1	2	0
LOC100362	3627	646	468	186
LOC100362	613	675	576	680
LOC100362	5579	3013	5660	3633
LOC100362	470	1167	661	595
LOC100363	30	50	51	51
LOC100363	57	0	212	0
LOC100363	46	24	21	23
LOC100363	16	42	0	3
LOC100363	2103	1861	1485	1852
LOC100363	52	29	17	13
LOC100363	5	0	14	0
LOC100363	11	7	9	19
LOC100363	34	92	59	14
LOC100363	155	535	363	371
LOC100363	5	43	28	60
LOC100363	20	17	29	24
LOC100363	27	0	32	37
LOC100363	11701	9364	10088	10079
LOC100363	61506	40785	51864	45348
LOC100363	0	4	3	8
LOC100363	15	95	39	29
LOC100363	15	43	15	5
LOC100363	2727	2792	1402	1742

LOC10036З	1946	1453	2291	2613
LOC10036З	4539	4033	1474	1691
LOC10036З	2235	1942	2344	1805
LOC10036З	340	522	264	186
LOC100364	471	709	460	358
LOC100364	1298	1943	629	1733
LOC100364	41	13	2	1
LOC100364	3949	4511	3671	3945
LOC100364	4030	9708	2710	5612
LOC100364	224	53	190	51
LOC100364	35	2	27	18
LOC100364	19	20	24	5
LOC100364	26	27	26	8
LOC100364	1452	2496	1742	1381
LOC100365	1	11	17	23
LOC100365	1436	900	987	1932
LOC100365	41	2	99	0
LOC100365	1853	1517	6	15
LOC100365	13882	12706	7004	5640
LOC100365	562	510	615	739
LOC100366	13	0	14	0
LOC100366	961	974	1056	845
LOC100909	8	15	6	10
LOC100909	215	299	212	252
LOC100909	38	50	56	6
LOC100909	94	9	20	93
LOC100909	891	1062	843	1239
LOC100909	20	56	50	90
LOC100909	184	28	93	8
LOC100909	97	8	71	85
LOC100909	103	118	29	113
LOC100909	1581	24	326	56
LOC100909	834	424	510	441
LOC100909	0	16	0	15
LOC100909	96	14	87	73
LOC100909	68	41	77	66
LOC100909	37	0	12	0
LOC100909	93	42	62	92
LOC100909	68	80	113	48
LOC100909	112	175	158	200
LOC100909	75	13	18	56
LOC100909	112	83	104	119
LOC100909	73	72	56	43
LOC100909	277	1271	169	69
LOC100909	1	17	33	39
LOC100909	329	432	349	503
LOC100909	540	229	469	448

LOC10090(	126	90	42	38
LOC10090(	54	168	78	167
LOC10090(	508	407	223	446
LOC10090(	34	14	26	19
LOC10091(	3172	2315	2556	3142
LOC10091(	185	136	185	177
LOC10091(	209	103	169	142
LOC10091(	200	99	96	138
LOC10091(	1675	3794	1205	427
LOC10091(	0	9	6	0
LOC10091(	33	47	3	16
LOC10091(	0	15	15	0
LOC10091(	6	19	5	2
LOC10091(	953	536	649	855
LOC10091(	4	10	2	0
LOC10091(	1252	981	781	2059
LOC10091(	54	0	54	34
LOC10091(	0	19	11	0
LOC10091(	103	96	72	70
LOC10091(	435	432	417	536
LOC10091(	17	11	8	0
LOC10091(	0	19	2	0
LOC10091(	4	0	0	6
LOC10091(	390	296	193	242
LOC10091(	14	1	23	0
LOC10091(	217	152	659	713
LOC10091(	123	105	117	127
LOC10091(	0	9	8	0
LOC10091(	41	35	59	35
LOC10091(	24	71	24	34
LOC10091(	1045	925	1125	987
LOC10091(	3	24	50	1
LOC10091(	2298	1873	1199	790
LOC10091(	1	1	9	3
LOC10091(	533	1192	629	1148
LOC10091(	2449	947	1575	1829
LOC10091(	69	40	83	47
LOC10091(	49	0	21	0
LOC10091(	8	36	24	7
LOC10091(	346	772	638	1366
LOC10091(	3742	2376	3033	3769
LOC10091(	31	0	33	73
LOC10091(	5769	5315	5110	6756
LOC10091(	47	80	107	106
LOC10091(	977	940	737	901
LOC10091(	53	16	47	1
LOC10091(	40	48	38	11

LOC10091(	164	62	11	74
LOC10091(	17	16	32	16
LOC10091(	10390	3544	7251	6524
LOC10091(	59	78	9	17
LOC100911	3072	2821	2737	2905
LOC100911	185	108	90	195
LOC100911	421	924	653	590
LOC100911	53	36	56	80
LOC100911	2578	3766	2594	2523
LOC100911	13	0	12	30
LOC100911	33	0	6	0
LOC100911	962	766	925	827
LOC100911	537	375	537	324
LOC100911	165	164	122	131
LOC100911	767	227	430	168
LOC100911	866	577	879	549
LOC100911	90	122	128	73
LOC100911	155	70	131	89
LOC100911	11	17	5	2
LOC100911	315	190	304	341
LOC100911	2795	2585	2528	1840
LOC100911	5	317	123	73
LOC100911	428	1320	913	584
LOC100911	13	56	60	71
LOC100911	1115	1404	579	861
LOC100911	932	680	847	1962
LOC100911	8052	2	6899	5
LOC100911	102	148	80	58
LOC100911	664	707	664	720
LOC100911	460	177	333	204
LOC100911	204	150	206	66
LOC100911	13	1	0	10
LOC100911	32	17	36	113
LOC100911	3318	2559	2424	2594
LOC100911	33	4	9	5
LOC100911	18	12	8	3
LOC100911	1145	901	1121	1354
LOC100911	65	92	75	76
LOC100911	26	117	26	38
LOC100911	49	116	185	222
LOC100911	7	0	11	49
LOC100911	30372	27410	18955	18822
LOC100911	6	21	24	99
LOC100911	2079	1372	1156	1287
LOC100911	135	57	80	2
LOC100911	58	50	48	44
LOC100911	319	434	337	368

LOC100911	241	24	45	0
LOC100911	140	88	150	54
LOC100912	685	749	677	476
LOC100912	195	143	147	106
LOC100912	2887	2325	2121	1823
LOC100912	3	0	0	30
LOC100912	15	6	8	14
LOC100912	1232	1279	1306	1770
LOC100912	7	17	5	0
LOC100912	0	1	3	1
LOC100912	115	39	134	15
LOC100912	505	492	630	350
LOC100912	64	35	33	6
LOC100912	832	1186	609	449
LOC100912	106	98	96	181
LOC100912	94	128	65	49
LOC100912	47	111	107	56
LOC100912	558	327	310	179
LOC100912	44	52	12	0
LOC100912	64	32	63	105
LOC100912	72	28	11	22
LOC100912	171	107	32	205
LOC100912	75	69	113	147
LOC100912	14	0	12	44
LOC100912	11	49	21	14
LOC100912	103	84	95	45
LOC100912	14	9	17	18
LOC100912	19	14	3	44
LOC100912	1042	2434	1559	1749
LOC100912	12	1	75	99
LOC100912	23	30	61	0
LOC100912	15	0	2	0
LOC100912	52	71	50	67
LOC100912	480	296	382	341
LOC100912	1462	1968	2189	4321
LOC100912	13	12	13	8
LOC100912	39	29	92	13
LOC100912	18	0	122	0
LOC100912	14	13	15	12
LOC100912	179	208	207	179
LOC100912	49	62	33	5
LOC100912	128	60	99	246
LOC100912	199	201	184	255
LOC100912	4	26	2	18
LOC100912	101	38	125	64
LOC100912	18	0	6	0
LOC100912	298	140	220	129

LOC10254€	17	0	6	0
LOC10254€	1	0	2	0
LOC10254€	14	17	15	23
LOC10254€	78	71	81	171
LOC10254€	39	5	24	8
LOC10254€	0	16	3	13
LOC10254€	0	7	11	13
LOC10254€	15	26	8	29
LOC10254€	151	246	193	130
LOC10254€	103	116	99	56
LOC10254€	63	132	65	64
LOC10254€	14	24	30	3
LOC10254€	13	0	2	0
LOC10254€	10	1	3	5
LOC10254€	53	39	53	4
LOC10254€	21	49	20	67
LOC10254€	0	11	0	1
LOC10254€	1167	952	1107	1165
LOC10254€	12	22	3	53
LOC10254€	102	38	215	84
LOC10254€	2	49	9	8
LOC10254€	125	140	254	282
LOC10254€	103	148	20	44
LOC10254€	32	24	9	28
LOC10254€	9	9	15	0
LOC10254€	11	37	2	0
LOC10254€	15	0	3	0
LOC10254€	69	186	114	144
LOC10254€	9	5	2	12
LOC10254€	14	76	53	26
LOC10254€	2	0	15	14
LOC10254€	14	7	6	4
LOC10254€	860	782	407	302
LOC10254€	69	45	36	54
LOC10254€	139	258	175	257
LOC10254€	8	31	23	0
LOC10254€	31	0	5	17
LOC10254€	123	147	62	237
LOC10254€	10	31	24	4
LOC10254€	299	0	0	128
LOC10254€	143	8	78	38
LOC102547	56	92	55	97
LOC102547	0	14	26	60
LOC102547	34	101	35	34
LOC102547	0	20	2	0
LOC102547	14	26	26	24
LOC102547	14	28	12	0

LOC102547	6	17	21	35
LOC102547	18	36	51	73
LOC102547	19	10	2	10
LOC102547	9	15	3	0
LOC102547	295	163	202	118
LOC102547	87	115	35	72
LOC102547	19	0	5	3
LOC102547	46	4	9	60
LOC102547	32	0	21	19
LOC102547	174	85	75	40
LOC102547	6	11	14	10
LOC102547	10	10	14	29
LOC102547	148	77	173	170
LOC102547	3	22	23	40
LOC102547	1178	708	973	1611
LOC102547	18	6	2	5
LOC102547	326	124	150	220
LOC102547	40	41	50	61
LOC102547	23	38	3	18
LOC102547	18	28	12	30
LOC102547	21	45	42	56
LOC102547	0	7	0	19
LOC102547	9	0	2	7
LOC102547	32	39	59	96
LOC102547	0	2	0	15
LOC102547	33	33	42	210
LOC102547	34	17	8	12
LOC102547	264	64	0	92
LOC102547	94	82	99	114
LOC102547	28	100	39	66
LOC102547	408	734	621	775
LOC102547	41	23	8	0
LOC102547	22	5	15	31
LOC102547	2	23	17	5
LOC102547	19	8	6	20
LOC102547	50	58	87	29
LOC102547	9	0	9	0
LOC102547	17	34	11	0
LOC102547	0	16	11	22
LOC102547	1206	716	1001	432
LOC102547	11	0	5	4
LOC102547	5	30	33	72
LOC102547	8	10	8	0
LOC102547	32	93	50	94
LOC102547	20	85	99	45
LOC102547	512	412	382	528
LOC102547	149	70	56	165

LOC102547	14	22	3	12
LOC102547	1	25	12	13
LOC102547	3	69	24	34
LOC102547	63	0	6	15
LOC102547	23	25	6	38
LOC102547	22	8	2	12
LOC102547	14	8	6	0
LOC102547	43	54	5	0
LOC102547	882	786	877	1113
LOC102547	0	10	0	17
LOC102547	59	2	36	21
LOC102547	86	75	205	98
LOC102547	21	0	14	49
LOC102547	284	395	477	736
LOC102547	36	8	41	22
LOC102547	18	14	9	6
LOC102547	84	98	161	94
LOC102547	24	0	5	6
LOC102548	15	17	11	12
LOC102548	151	136	77	64
LOC102548	215	100	92	63
LOC102548	24	30	14	0
LOC102548	18	56	42	15
LOC102548	12	0	9	0
LOC102548	187	253	290	430
LOC102548	47	21	23	0
LOC102548	28	6	33	0
LOC102548	8	0	26	15
LOC102548	6	6	0	8
LOC102548	101	122	169	193
LOC102548	25	32	29	15
LOC102548	8	21	8	0
LOC102548	8	21	9	0
LOC102548	131	124	184	514
LOC102548	75	307	205	271
LOC102548	0	15	6	0
LOC102548	44	147	74	146
LOC102548	222	515	402	86
LOC102548	41	19	20	27
LOC102548	62	23	33	0
LOC102548	34	12	9	0
LOC102548	0	67	45	31
LOC102548	278	90	21	191
LOC102548	1	16	0	29
LOC102548	567	256	448	606
LOC102548	5	0	18	0
LOC102548	42	44	30	4

LOC102548	15	0	21	0
LOC102548	61	57	176	64
LOC102548	39	56	27	82
LOC102548	136	31	36	113
LOC102548	30	27	2	0
LOC102548	12	8	14	8
LOC102548	205	112	123	57
LOC102548	60	87	125	14
LOC102548	175	105	103	144
LOC102548	69	51	80	70
LOC102548	45	691	232	43
LOC102548	17	0	14	0
LOC102548	13	31	11	0
LOC102548	382	1058	668	161
LOC102548	13	0	5	12
LOC102548	123	117	119	136
LOC102548	1	8	9	0
LOC102548	1	5	0	41
LOC102548	8	0	24	15
LOC102548	24	85	42	6
LOC102548	60	1	20	17
LOC102548	8	17	6	26
LOC102548	27	208	45	55
LOC102548	0	2	21	5
LOC102548	277	114	202	50
LOC102548	41	9	18	7
LOC102548	64	46	57	34
LOC102548	23	9	5	11
LOC102548	158	112	104	93
LOC102548	8	12	12	17
LOC102548	33	24	44	72
LOC102548	122	60	56	72
LOC102548	22	0	6	2
LOC102548	5	7	2	0
LOC102548	37	0	24	48
LOC102548	6	47	21	0
LOC102548	0	17	15	0
LOC102548	56	40	2	93
LOC102548	112	94	48	35
LOC102548	206	109	153	224
LOC102548	37	18	47	11
LOC102548	1301	1429	1567	1346
LOC102549	10	9	5	0
LOC102549	18	32	18	7
LOC102549	8	0	2	0
LOC102549	445	160	93	332
LOC102549	18	4	12	24

LOC10254‹	18	17	11	3
LOC10254‹	355	57	128	107
LOC10254‹	175	271	132	55
LOC10254‹	236	197	217	193
LOC10254‹	34	8	32	56
LOC10254‹	35	30	74	84
LOC10254‹	34	63	39	6
LOC10254‹	2	0	2	5
LOC10254‹	430	312	408	359
LOC10254‹	21	40	32	23
LOC10254‹	70	54	54	35
LOC10254‹	60	42	102	96
LOC10254‹	14	2	53	0
LOC10254‹	8	0	12	0
LOC10254‹	126	57	101	136
LOC10254‹	63	17	69	77
LOC10254‹	41	12	36	18
LOC10254‹	5	0	8	0
LOC10254‹	48	31	3	7
LOC10254‹	10	40	20	5
LOC10254‹	141	181	196	98
LOC10254‹	11	29	12	0
LOC10254‹	44	77	50	79
LOC10254‹	22	0	21	32
LOC10254‹	25	16	2	13
LOC10254‹	29	57	17	47
LOC10254‹	18	9	12	13
LOC10254‹	103	115	193	25
LOC10254‹	35	14	36	0
LOC10254‹	0	32	9	17
LOC10254‹	8	19	5	0
LOC10254‹	23	0	17	20
LOC10254‹	23	36	59	35
LOC10254‹	35	22	20	24
LOC10254‹	96	74	53	84
LOC10254‹	72	28	44	0
LOC10254‹	29	26	18	70
LOC10254‹	19	31	11	12
LOC10254‹	67	126	18	43
LOC10254‹	2	28	50	75
LOC10254‹	25	0	6	57
LOC10254‹	10	17	0	19
LOC10254‹	166	74	101	42
LOC10254‹	8	38	18	0
LOC10254‹	47	21	18	14
LOC10254‹	28	67	15	42
LOC10254‹	558	515	725	1236

LOC10254(	88	32	47	89
LOC10254(	16	0	44	6
LOC10254(	151	81	63	11
LOC10254(	263	115	206	210
LOC10254(	121	120	131	329
LOC10254(	34	0	3	0
LOC10254(	6	0	11	19
LOC10254(	0	22	14	13
LOC10254(	26	0	39	0
LOC10254(	4	7	9	24
LOC10254(	607	1060	1121	602
LOC10254(	132	149	217	101
LOC10254(	41	49	38	74
LOC10255(	0	50	23	75
LOC10255(	0	12	6	0
LOC10255(	29	68	95	89
LOC10255(	58	43	74	37
LOC10255(	8	18	8	0
LOC10255(	0	9	9	0
LOC10255(	0	11	6	18
LOC10255(	103	69	101	32
LOC10255(	48	17	11	10
LOC10255(	25	148	108	241
LOC10255(	41	10	35	0
LOC10255(	23	26	21	8
LOC10255(	1444	1346	1270	1611
LOC10255(	9	14	15	0
LOC10255(	0	13	2	0
LOC10255(	141	140	169	116
LOC10255(	22	0	26	0
LOC10255(	44	23	119	50
LOC10255(	56	28	77	77
LOC10255(	3361	1506	847	251
LOC10255(	275	165	169	112
LOC10255(	0	36	0	40
LOC10255(	754	259	257	476
LOC10255(	12	0	30	0
LOC10255(	38	17	14	54
LOC10255(	48	51	36	29
LOC10255(	81	22	17	7
LOC10255(	3	15	3	0
LOC10255(	23	18	3	20
LOC10255(	694	642	682	641
LOC10255(	38	13	48	4
LOC10255(	23	0	21	10
LOC10255(	6	0	3	0
LOC10255(	27	28	12	0

LOC10255(	79	77	30	18
LOC10255(	8	19	18	39
LOC10255(	3	78	5	0
LOC10255(	87	1	62	49
LOC10255(	0	12	17	0
LOC10255(	1058	11	3	6
LOC10255(	10	0	6	0
LOC10255(	21	16	9	18
LOC10255(	3955	1425	2152	3335
LOC10255(	98	121	156	59
LOC10255(	461	134	20	14
LOC10255(	38	55	90	23
LOC10255(	111	102	59	105
LOC10255(	2	5	17	22
LOC10255(	100	0	71	0
LOC10255(	36	7	24	13
LOC10255(	55	12	20	0
LOC10255(	57	41	50	39
LOC10255(	0	4	17	0
LOC10255(	9	12	30	35
LOC10255(	51	34	11	37
LOC10255(	44	47	104	82
LOC10255(	97	48	78	71
LOC10255(	12	20	0	4
LOC10255(	1	0	5	0
LOC10255(	313	159	167	319
LOC10255(	100	123	164	122
LOC10255(	2	10	6	0
LOC10255(	10	0	3	14
LOC10255(	16	0	8	0
LOC10255(	185	136	179	153
LOC10255(	0	53	9	0
LOC10255(	115	212	194	186
LOC102551	30	31	51	37
LOC102551	17	31	18	18
LOC102551	18	6	5	0
LOC102551	38	27	21	5
LOC102551	16	0	3	0
LOC102551	7	0	17	0
LOC102551	74	33	39	34
LOC102551	14	36	8	10
LOC102551	12	0	2	20
LOC102551	892	699	963	703
LOC102551	66	70	93	20
LOC102551	464	341	388	426
LOC102551	32	0	5	10
LOC102551	107	59	41	23

LOC102551	201	73	190	220
LOC102551	0	18	6	0
LOC102551	44	81	17	33
LOC102551	89	56	51	108
LOC102551	0	13	6	0
LOC102551	33	61	50	1
LOC102551	22	0	20	12
LOC102551	50	63	86	56
LOC102551	527	143	284	251
LOC102551	37	0	15	25
LOC102551	54	2	0	6
LOC102551	24	12	9	18
LOC102551	58	28	48	59
LOC102551	25	14	24	53
LOC102551	5	0	8	0
LOC102551	2	0	0	25
LOC102551	12920	14808	13571	4245
LOC102551	261	142	155	291
LOC102551	0	16	14	0
LOC102551	196	277	336	210
LOC102551	64	66	87	34
LOC102551	4	0	2	3
LOC102551	15	8	9	22
LOC102551	27	32	21	16
LOC102551	10	0	3	0
LOC102551	3	6	6	0
LOC102551	4	0	8	0
LOC102551	18	36	0	22
LOC102551	212	338	226	208
LOC102551	21	0	8	16
LOC102551	74	158	105	187
LOC102551	634	0	105	129
LOC102551	4	10	42	11
LOC102551	1499	1314	1647	1563
LOC102551	74	29	18	44
LOC102551	39	50	12	19
LOC102551	7	37	36	82
LOC102551	24	0	14	0
LOC102551	0	2	14	0
LOC102551	128	14	15	13
LOC102551	570	897	886	864
LOC102551	87	97	128	42
LOC102551	57	127	96	107
LOC102551	267	304	272	223
LOC102551	124	50	21	0
LOC102551	64	7	8	36
LOC102551	64	103	89	203

LOC102551	371	153	166	87
LOC102551	32	23	2	29
LOC102552	23	3	8	35
LOC102552	1	22	21	0
LOC102552	13	15	0	10
LOC102552	13	0	0	10
LOC102552	1282	805	957	821
LOC102552	12	18	2	0
LOC102552	20	10	18	22
LOC102552	10	10	30	10
LOC102552	0	1	6	0
LOC102552	21	28	5	46
LOC102552	292	382	256	89
LOC102552	300	117	196	97
LOC102552	370	306	313	419
LOC102552	15	0	17	47
LOC102552	22	3	44	0
LOC102552	42	23	44	14
LOC102552	42	54	8	29
LOC102552	44	62	75	72
LOC102552	0	11	11	0
LOC102552	435	298	584	444
LOC102552	8	0	11	19
LOC102552	16	92	33	97
LOC102552	202	27	9	16
LOC102552	35	57	57	138
LOC102552	71	9	8	11
LOC102552	0	7	3	0
LOC102552	1	0	11	0
LOC102552	1163	1196	1035	1192
LOC102552	116	120	203	134
LOC102552	79	98	41	91
LOC102552	9	6	17	24
LOC102552	231	96	63	85
LOC102552	11	6	12	0
LOC102552	86	0	36	12
LOC102552	0	4	6	0
LOC102552	154	67	137	100
LOC102552	34	84	33	169
LOC102552	1	0	39	0
LOC102552	210	41	6	0
LOC102552	7	0	11	8
LOC102552	7	0	30	18
LOC102552	5	1	8	12
LOC102552	216	0	58	63
LOC102552	1026	509	625	585
LOC102552	15	27	44	16

LOC102552	18	8	21	35
LOC102552	31	35	8	47
LOC102552	8	16	8	0
LOC102552	12	44	6	0
LOC102552	69	56	161	51
LOC102552	116	226	125	136
LOC102552	24	51	48	30
LOC102552	7	4	40	59
LOC102552	101	162	110	138
LOC102552	32	55	18	30
LOC102552	427	321	251	390
LOC102552	42	7	5	29
LOC102552	135	248	284	274
LOC102552	3	12	2	0
LOC102552	30	38	15	68
LOC102552	9	23	2	8
LOC102552	917	1439	891	1361
LOC102552	47	0	26	0
LOC102552	128	223	131	80
LOC102552	46	15	3	0
LOC102552	250	103	50	150
LOC102552	1	9	11	0
LOC102552	17	21	17	42
LOC102552	14	19	56	48
LOC102553	19	68	35	73
LOC102553	345	236	197	135
LOC102553	80	64	69	61
LOC102553	25	0	24	3
LOC102553	75	57	78	41
LOC102553	8	0	2	22
LOC102553	2	31	2	13
LOC102553	16	0	5	0
LOC102553	14	24	29	30
LOC102553	20	84	79	125
LOC102553	190	155	117	126
LOC102553	93	26	65	79
LOC102553	50	113	74	48
LOC102553	4	15	68	33
LOC102553	1	12	3	6
LOC102553	29	6	15	11
LOC102553	23	16	24	0
LOC102553	44	61	56	106
LOC102553	600	410	715	645
LOC102553	14	13	35	14
LOC102553	6	100	35	41
LOC102553	101	30	69	29
LOC102553	25	32	83	30

LOC10255З	603	508	311	457
LOC10255З	1338	1824	1547	1527
LOC10255З	1098	316	830	292
LOC10255З	24	25	44	23
LOC10255З	24	70	24	50
LOC10255З	41	37	33	34
LOC10255З	3	30	0	3
LOC10255З	7	13	2	0
LOC10255З	14	17	8	4
LOC10255З	7	14	15	0
LOC10255З	6	0	2	31
LOC10255З	0	10	5	0
LOC10255З	13	12	0	29
LOC10255З	43	150	102	48
LOC10255З	0	8	2	0
LOC10255З	1372	901	1188	1530
LOC10255З	463	304	388	454
LOC10255З	6	19	23	0
LOC10255З	0	8	15	13
LOC10255З	1952	2032	2089	2468
LOC10255З	25	29	2	22
LOC10255З	40	66	96	53
LOC10255З	34	39	35	53
LOC10255З	280	329	242	241
LOC10255З	126	166	60	168
LOC10255З	65	41	58	102
LOC10255З	7	3	14	37
LOC10255З	8	18	5	2
LOC10255З	41	10	33	26
LOC10255З	426	296	197	363
LOC10255З	1	8	14	12
LOC10255Ч	11	23	9	21
LOC10255Ч	390	276	193	66
LOC10255Ч	503	394	147	167
LOC10255Ч	0	8	2	37
LOC10255Ч	25	0	11	3
LOC10255Ч	41	96	74	110
LOC10255Ч	32	17	17	69
LOC10255Ч	18	28	27	43
LOC10255Ч	0	24	14	0
LOC10255Ч	9	0	2	0
LOC10255Ч	1	14	2	0
LOC10255Ч	252	148	178	153
LOC10255Ч	143	84	99	223
LOC10255Ч	41	160	143	27
LOC10255Ч	14	3	15	40
LOC10255Ч	42	25	15	25

LOC10255‹	11	22	12	21
LOC10255‹	934	1100	888	635
LOC10255‹	34	61	14	19
LOC10255‹	216	167	184	209
LOC10255‹	5	27	12	30
LOC10255‹	0	6	2	0
LOC10255‹	42	80	53	35
LOC10255‹	306	236	289	335
LOC10255‹	21	0	26	0
LOC10255‹	26	7	11	41
LOC10255‹	12	14	5	0
LOC10255‹	162	113	248	142
LOC10255‹	209	31	81	0
LOC10255‹	22	1	9	0
LOC10255‹	19	10	3	25
LOC10255‹	722	442	564	658
LOC10255‹	176	80	99	85
LOC10255‹	5	0	5	0
LOC10255‹	73	38	32	11
LOC10255‹	48	40	12	16
LOC10255‹	10	25	38	49
LOC10255‹	4	0	0	5
LOC10255‹	3	21	21	31
LOC10255‹	341	1334	507	538
LOC10255‹	68	11	59	26
LOC10255‹	1069	572	855	388
LOC10255‹	218	94	211	95
LOC10255‹	141	121	188	44
LOC10255‹	14	0	21	13
LOC10255‹	91	71	102	99
LOC10255‹	10	52	92	55
LOC10255‹	1	18	17	2
LOC10255‹	59	39	56	67
LOC10255‹	6	5	18	45
LOC10255‹	14	19	5	21
LOC10255‹	145	122	184	132
LOC10255‹	123	250	101	213
LOC10255‹	14	0	9	13
LOC10255‹	171	61	215	260
LOC10255‹	2	13	6	0
LOC10255‹	5	24	15	27
LOC10255‹	9	0	9	0
LOC10255‹	16	42	6	17
LOC10255‹	27	51	36	104
LOC10255‹	0	7	12	19
LOC10255‹	98	168	153	109
LOC10255‹	441	64	52	235

LOC102554	21	11	27	27
LOC102554	360	206	271	303
LOC102554	33	16	46	21
LOC102554	0	15	8	0
LOC102554	36	23	45	44
LOC102554	5	0	0	13
LOC102554	80	91	78	99
LOC102554	21	0	3	0
LOC102554	7	31	15	0
LOC102554	10	0	17	0
LOC102555	2	0	3	0
LOC102555	52	37	59	30
LOC102555	3	0	0	6
LOC102555	16	9	27	43
LOC102555	9	23	9	0
LOC102555	446	246	396	383
LOC102555	40	13	51	35
LOC102555	140	290	144	38
LOC102555	368	735	471	591
LOC102555	151	403	296	265
LOC102555	36	12	44	75
LOC102555	257	176	259	460
LOC102555	142	152	98	98
LOC102555	9	12	29	22
LOC102555	47	41	66	14
LOC102555	18	51	32	16
LOC102555	295	97	196	164
LOC102555	84	33	74	31
LOC102555	324	137	271	227
LOC102555	1	0	3	0
LOC102555	1	22	45	8
LOC102555	77	130	125	117
LOC102555	10866	10010	6121	7812
LOC102555	3	0	9	0
LOC102555	9	25	6	14
LOC102555	66	47	68	44
LOC102555	25	17	14	0
LOC102555	147	108	120	39
LOC102555	573	412	293	393
LOC102555	4	11	11	0
LOC102555	15	11	6	52
LOC102555	10	96	38	56
LOC102555	107	149	89	71
LOC102555	399	120	250	189
LOC102555	16	41	17	30
LOC102555	157	76	143	163
LOC102555	26	41	33	16

LOC102555	54	96	47	39
LOC102555	95	0	135	11
LOC102555	23	0	29	0
LOC102555	578	386	504	663
LOC102555	45	63	30	20
LOC102555	206	107	135	60
LOC102555	57	28	26	26
LOC102555	1142	746	518	815
LOC102555	5	6	3	0
LOC102555	3	0	5	0
LOC102555	157	176	301	203
LOC102555	77	78	21	87
LOC102555	229	197	178	229
LOC102555	17	31	12	26
LOC102555	7	4	5	0
LOC102555	4	7	5	7
LOC102556	739	550	584	729
LOC102556	39	62	36	73
LOC102556	69	40	89	133
LOC102556	8	23	3	15
LOC102556	0	4	5	4
LOC102556	23	76	63	91
LOC102556	29	34	41	0
LOC102556	44	59	47	97
LOC102556	113	34	14	49
LOC102556	288	228	263	193
LOC102556	29	5	11	26
LOC102556	390	253	349	282
LOC102556	15	26	6	10
LOC102556	11	6	3	20
LOC102556	683	1008	984	602
LOC102556	23	0	11	0
LOC102556	33	30	26	27
LOC102556	16	10	17	33
LOC102556	1	43	15	33
LOC102556	26	0	9	11
LOC102556	15	0	14	11
LOC102556	8	12	12	0
LOC102556	20	0	8	24
LOC102556	3	0	6	0
LOC102556	88	41	54	88
LOC102556	9	56	41	99
LOC102556	51	0	3	48
LOC102556	38	0	24	8
LOC102556	97	36	42	103
LOC102556	48	60	59	33
LOC102556	95	64	77	107

LOC102557	8	44	20	0
LOC102557	3	14	9	1
LOC102557	6	0	6	8
LOC102557	614	304	462	537
LOC102557	42	36	20	0
LOC102557	3	3	5	7
LOC102557	203	157	65	102
LOC102557	116	58	90	165
LOC103689	57	230	184	203
LOC103689	40	40	0	17
LOC103689	37	102	120	48
LOC103689	5	9	15	4
LOC103689	6	17	3	26
LOC103689	1683	142	298	612
LOC103690	16	97	87	33
LOC103690	23	24	18	27
LOC103690	2405	2964	2609	2785
LOC103690	1	1	3	0
LOC103690	198	6	62	322
LOC103690	7	0	3	0
LOC103690	224	714	388	409
LOC103690	10	10	12	0
LOC103690	71	69	93	8
LOC103690	26	11	2	0
LOC103690	579	525	599	459
LOC103690	0	10	2	0
LOC103690	0	11	20	0
LOC103690	22	0	33	0
LOC103690	0	18	17	0
LOC103690	54	25	22	1
LOC103690	6312	3564	3298	4191
LOC103690	49	44	53	30
LOC103690	50	42	59	89
LOC103690	61	0	20	20
LOC103690	1	13	15	0
LOC103690	715	415	751	912
LOC103690	14	24	27	7
LOC103690	12	10	26	25
LOC103690	0	9	6	0
LOC103690	159	74	86	54
LOC103690	91	0	17	0
LOC103690	14	10	6	0
LOC103690	13	10	3	27
LOC103690	55	18	75	0
LOC103690	1229	483	322	865
LOC103690	37	9	5	49
LOC103690	8	0	6	0

LOC10369(	604	394	573	602
LOC10369(	202	84	196	61
LOC10369(	85	117	117	199
LOC10369(	2	0	9	0
LOC103691	250	153	221	158
LOC103691	173	100	102	82
LOC103691	0	33	11	0
LOC103691	29	64	39	42
LOC103691	12	0	8	5
LOC103691	165	82	131	148
LOC103691	18	55	57	77
LOC103691	88	29	39	50
LOC103691	109	128	54	80
LOC103691	23	0	11	25
LOC103691	2	0	21	47
LOC103691	13	11	18	6
LOC103691	20	0	17	0
LOC103691	115	51	78	93
LOC103691	67	50	6	0
LOC103691	56	99	75	85
LOC103691	9	8	15	16
LOC103691	10	0	6	0
LOC103691	6	19	2	0
LOC103691	0	11	15	0
LOC103691	349	262	197	122
LOC103691	69	12	29	34
LOC103691	18	29	21	0
LOC103691	0	11	8	0
LOC103691	9	47	3	11
LOC103691	41	64	68	74
LOC103691	25	0	5	0
LOC103691	65	130	120	324
LOC103691	0	25	39	0
LOC103691	157	114	135	307
LOC103691	19	20	23	0
LOC103691	3	0	5	8
LOC103691	104	214	217	117
LOC103691	69	69	29	56
LOC103691	3	41	41	33
LOC103691	160	14	8	4
LOC103691	54	16	9	14
LOC103691	5	11	17	0
LOC103691	6	0	5	0
LOC103691	325	407	272	257
LOC103691	1742	1847	1581	1874
LOC103691	14	3	3	0
LOC103691	660	328	813	556

LOC103691	94	50	30	33
LOC103691	0	4	0	7
LOC103691	6	11	6	25
LOC103691	9	81	44	24
LOC103691	168	152	131	133
LOC103691	4	0	2	0
LOC103691	133	183	111	221
LOC103691	7	15	26	53
LOC103691	100	124	80	56
LOC103691	149	241	357	401
LOC103691	11492	19516	11403	10686
LOC103691	3	8	15	17
LOC103691	29	27	69	2
LOC103691	20	162	32	3
LOC103691	6	59	30	16
LOC103692	14	0	11	0
LOC103692	3	0	23	13
LOC103692	5	0	32	0
LOC103692	0	22	6	0
LOC103692	91	116	17	19
LOC103692	11	0	21	4
LOC103692	87	38	50	60
LOC103692	19	53	47	37
LOC103692	167	452	352	357
LOC103692	11	12	5	40
LOC103692	0	47	9	0
LOC103692	88	23	3	6
LOC103692	325	350	507	413
LOC103692	35	0	11	15
LOC103692	56	4	5	0
LOC103692	329	132	117	49
LOC103692	54	10	9	0
LOC103692	62	30	39	125
LOC103692	31	39	44	22
LOC103692	1	30	0	1
LOC103692	1	0	8	0
LOC103692	37	62	14	43
LOC103692	37	31	20	41
LOC103692	42	41	54	84
LOC103692	1351	703	758	931
LOC103692	0	24	8	18
LOC103692	33	29	9	34
LOC103692	17	22	14	2
LOC103692	39	0	9	0
LOC103692	3	3	38	20
LOC103692	3	0	3	0
LOC103692	12	0	12	0

LOC103692	613	766	270	299
LOC103692	15	0	32	41
LOC103692	1	9	3	4
LOC103692	0	9	3	0
LOC103692	47	69	0	12
LOC103692	52	45	89	31
LOC103692	7	21	20	22
LOC103692	16	31	39	36
LOC103692	137	99	152	160
LOC103692	15	3	32	20
LOC103692	29	0	3	0
LOC103692	131	274	150	127
LOC103692	20	77	17	31
LOC103692	335	502	638	455
LOC103692	13	1	23	19
LOC103693	178	123	403	461
LOC103693	12	16	24	31
LOC103693	69	109	98	77
LOC103693	16	15	30	0
LOC103693	211	147	167	255
LOC103693	6	10	51	37
LOC103693	61	73	78	0
LOC103693	15	14	3	0
LOC103693	61	12	15	0
LOC103693	5	0	8	0
LOC103693	23	0	2	3
LOC103693	868	666	684	1148
LOC103693	0	30	5	0
LOC103693	77	49	10	118
LOC103693	1	13	12	31
LOC103693	14	7	17	0
LOC103693	51	2	20	29
LOC103693	635	409	499	324
LOC103693	31	51	14	39
LOC103693	29	12	6	0
LOC103693	16	11	9	5
LOC103693	78	45	66	10
LOC103693	10	3	14	0
LOC103693	20	39	12	23
LOC103693	9	4	23	27
LOC103693	1534	1337	615	287
LOC103693	14	5	11	0
LOC103693	83	88	38	27
LOC103693	6	10	2	0
LOC103693	29	6	4	5
LOC103693	1	0	11	0
LOC103693	67	15	41	33

LOC103693	25	14	33	7
LOC103693	3520	4218	3306	2344
LOC103693	28	33	20	17
LOC103693	23	53	12	10
LOC103693	128	69	57	85
LOC103693	9	18	0	8
LOC103693	237	223	71	157
LOC103693	3	16	6	0
LOC103693	4761	2169	2141	3074
LOC103693	66	59	69	19
LOC103693	127	441	214	363
LOC103693	9	0	24	0
LOC103693	26	0	3	1
LOC103693	23	25	27	52
LOC103693	20	7	21	41
LOC103693	6	0	33	6
LOC103693	96	0	35	39
LOC103693	4	40	9	0
LOC103693	359	291	220	411
LOC103693	238	144	282	116
LOC103693	4	6	17	14
LOC103693	33	37	36	62
LOC103693	3	0	34	8
LOC103693	8	47	41	14
LOC103693	9	17	17	37
LOC103693	5147	5199	5379	5555
LOC103693	5	12	9	0
LOC103693	1157	1214	1440	1469
LOC103693	31	0	30	39
LOC103693	70	208	262	232
LOC103693	56	14	45	54
LOC103693	4	6	17	0
LOC103693	10	12	21	0
LOC103693	33	23	71	22
LOC103693	89	84	144	68
LOC103693	68	47	45	19
LOC103693	29	95	14	3
LOC103693	9	24	15	73
LOC103693	4	0	0	13
LOC103694	17	27	15	21
LOC103694	5	27	17	0
LOC103694	193	63	190	81
LOC103694	28	38	9	1
LOC103694	7	9	3	0
LOC103694	22	41	47	17
LOC103694	24	14	8	0
LOC103694	45	0	17	41

LOC103694	822	480	645	831
LOC103694	544	148	123	40
LOC103694	46	0	41	103
LOC103694	71	271	197	116
LOC103694	0	15	0	5
LOC103694	80	71	45	62
LOC103694	519	800	840	779
LOC103694	55	9	32	63
LOC103694	384	199	336	311
LOC103694	13	3	11	27
LOC103694	23	30	24	313
LOC103694	19	12	11	12
LOC103694	3	0	21	2
LOC103694	7	0	9	0
LOC103694	19	6	5	0
LOC103694	123	86	80	38
LOC103694	12	2	9	19
LOC103694	0	172	14	233
LOC103694	0	2	0	16
LOC103694	0	24	0	10
LOC103694	3	0	6	0
LOC103694	27	43	2	19
LOC103694	66	95	86	63
LOC103695	220	28	2	10
LOC103695	13	21	8	5
LOC103695	40	20	9	39
LOC106182	250	22	147	71
LOC106631	453	542	188	314
LOC106736	866	751	749	191
LOC108348	74	144	159	151
LOC108348	11	34	0	1
LOC108348	2234	2108	1810	1987
LOC108348	609	612	665	594
LOC108348	1	0	11	146
LOC108348	68	69	90	40
LOC108348	5	72	30	53
LOC108348	79	84	114	84
LOC108348	3851	2340	2797	3127
LOC108348	49	13	54	26
LOC108348	339	365	331	419
LOC108348	1052	1368	1235	1452
LOC108348	28	10	2	0
LOC108348	9	59	26	61
LOC108348	273	81	238	272
LOC108348	17	11	44	17
LOC108348	7	28	57	44
LOC108348	2	0	35	18

LOC108348	196	207	299	257
LOC108348	107	54	27	38
LOC108348	5	0	5	0
LOC108348	1	7	6	16
LOC108348	0	6	14	13
LOC108348	29	39	36	52
LOC108348	46	67	21	8
LOC108348	0	116	94	185
LOC108348	0	7	5	0
LOC108348	15	0	9	20
LOC108348	13	10	5	0
LOC108348	39	11	20	19
LOC108348	33	0	41	25
LOC108348	23	0	15	35
LOC108348	6136	3693	6107	4747
LOC108348	9	0	3	0
LOC108348	34	11	3	0
LOC108348	189	390	205	304
LOC108348	10	30	41	16
LOC108348	14	37	16	26
LOC108348	48	10	11	13
LOC108348	10	37	27	0
LOC108348	11	0	3	0
LOC108348	4	5	11	13
LOC108348	15	53	11	0
LOC108348	347	444	340	149
LOC108348	42	18	53	19
LOC108348	1	0	11	0
LOC108348	406	332	420	954
LOC108348	17	7	5	0
LOC108348	232	161	101	0
LOC108348	3	0	5	3
LOC108348	16	0	14	0
LOC108348	16	0	2	0
LOC108348	58	31	24	54
LOC108348	30	0	11	5
LOC108348	14	0	15	24
LOC108348	10	1	3	11
LOC108349	24	120	35	32
LOC108349	139	63	117	108
LOC108349	15	4	5	0
LOC108349	96	37	75	79
LOC108349	0	21	5	2
LOC108349	7	10	6	0
LOC108349	20	44	18	0
LOC108349	7	27	20	0
LOC108349	60	17	95	141

LOC10834‹	28	42	20	0
LOC10834‹	27	0	8	0
LOC10834‹	62	30	47	70
LOC10834‹	27	0	2	20
LOC10834‹	11	2	36	0
LOC10834‹	9	17	5	0
LOC10834‹	73	39	18	14
LOC10834‹	0	19	24	0
LOC10834‹	0	43	23	24
LOC10834‹	3	28	9	48
LOC10834‹	3	8	15	27
LOC10834‹	14	0	21	27
LOC10834‹	0	13	2	0
LOC10834‹	29	135	68	167
LOC10834‹	76	16	57	1
LOC10834‹	3	0	12	5
LOC10834‹	16	26	11	0
LOC10834‹	10	24	26	22
LOC10834‹	10	0	6	0
LOC10834‹	6	18	32	23
LOC10834‹	41	107	44	57
LOC10834‹	13	0	17	0
LOC10834‹	2492	1391	821	1184
LOC10834‹	135	184	149	469
LOC10834‹	1291	2034	1295	1728
LOC10834‹	91	148	134	108
LOC10834‹	1136	409	543	468
LOC10834‹	33	0	14	12
LOC10834‹	164	523	166	127
LOC10834‹	59	27	65	19
LOC10834‹	5	3	5	2
LOC10834‹	7	20	30	0
LOC10834‹	10	20	9	0
LOC10834‹	13	52	14	39
LOC10834‹	0	19	10	0
LOC10834‹	30	15	23	17
LOC10834‹	8	0	29	0
LOC10834‹	0	9	8	1
LOC10834‹	43	0	26	28
LOC10834‹	43	56	12	23
LOC10834‹	130	221	135	307
LOC10834‹	54	148	120	199
LOC10834‹	18	11	45	0
LOC10834‹	547	93	150	157
LOC10834‹	20	0	39	35
LOC10834‹	170	74	90	35
LOC10834‹	64	34	45	17

LOC10834(	4	7	15	13
LOC10834(	0	2	0	17
LOC10834(	151	209	205	165
LOC10834(	20	15	2	0
LOC10835(	112	28	80	27
LOC10835(	18	39	45	16
LOC10835(	55	56	116	124
LOC10835(	564	332	507	477
LOC10835(	175	110	92	140
LOC10835(	0	14	23	22
LOC10835(	25	14	11	13
LOC10835(	43	38	48	71
LOC10835(	13	5	8	23
LOC10835(	21	0	8	12
LOC10835(	44	10	51	38
LOC10835(	6056	715	864	218
LOC10835(	298	146	63	238
LOC10835(	29	11	33	33
LOC10835(	596	365	456	332
LOC10835(	35	0	24	0
LOC10835(	17	0	6	3
LOC10835(	18	24	9	47
LOC10835(	1	1	17	24
LOC10835(	23	7	3	20
LOC10835(	615	293	421	564
LOC10835(	31	14	28	81
LOC10835(	31	0	33	124
LOC10835(	4	0	11	0
LOC10835(	6	17	6	15
LOC10835(	15	55	2	0
LOC10835(	7	5	26	18
LOC10835(	53	14	45	126
LOC10835(	9	7	3	0
LOC10835(	74	101	3	5
LOC10835(	127	292	612	473
LOC10835(	23	21	12	1
LOC10835(	123	84	71	9
LOC10835(	0	10	14	0
LOC10835(	39	0	30	27
LOC10835(	16	0	139	0
LOC10835(	299	183	167	240
LOC10835(	19	40	38	33
LOC10835(	6	72	3	16
LOC10835(	10	0	2	0
LOC10835(	17	0	8	0
LOC10835(	31	78	38	2
LOC10835(	3279	1739	4241	3359

LOC10835(	36	44	84	58
LOC10835(	67	74	98	104
LOC10835(	8	0	9	0
LOC10835(	195	207	152	130
LOC10835(	34	29	50	48
LOC10835(	6	9	20	0
LOC10835(	399	507	492	271
LOC10835(	171	15	120	113
LOC10835(	1101	827	1041	1049
LOC10835(	513	296	357	354
LOC10835(	13	11	9	0
LOC10835(	20	36	44	40
LOC10835(	72	73	102	106
LOC10835(	31	2	60	38
LOC10835(	6	0	6	26
LOC10835(	25	28	11	7
LOC10835(	89	66	36	60
LOC10835(	9	10	3	0
LOC10835(	1	0	2	0
LOC10835(	23	44	6	10
LOC10835(	7	0	0	18
LOC10835(	979	390	345	624
LOC10835(	18	0	15	8
LOC10835(	4	12	14	34
LOC10835(	3	0	5	1
LOC108351	13	28	9	16
LOC108351	76	112	100	86
LOC108351	10	12	2	58
LOC108351	15	13	14	18
LOC108351	68	34	116	92
LOC108351	63	25	47	21
LOC108351	2468	2575	0	1
LOC108351	14	8	3	10
LOC108351	109	37	68	18
LOC108351	105	36	9	141
LOC108351	266	316	342	435
LOC108351	26	0	8	0
LOC108351	24	20	39	0
LOC108351	205	150	150	189
LOC108351	37	0	0	42
LOC108351	41	22	26	14
LOC108351	58	63	20	0
LOC108351	3	19	17	20
LOC108351	34	9	10	0
LOC108351	3	14	5	0
LOC108351	2	9	35	27
LOC108351	140	57	38	75

LOC108351	0	17	3	0
LOC108351	3	0	11	0
LOC108351	22	47	48	29
LOC108351	8	27	17	0
LOC108351	29	14	24	0
LOC108351	10	35	0	22
LOC108351	29	10	9	59
LOC108351	294	116	102	90
LOC108351	0	8	6	0
LOC108351	69	29	35	8
LOC108351	78	58	77	58
LOC108351	1	0	6	0
LOC108351	237	62	149	135
LOC108351	0	17	0	2
LOC108351	13	11	17	4
LOC108351	2	12	2	6
LOC108351	20	0	15	36
LOC108351	9	17	17	0
LOC108351	121	26	38	58
LOC108351	8	0	6	67
LOC108351	16	0	21	0
LOC108351	247	500	296	242
LOC108351	81	51	57	69
LOC108351	13	0	23	0
LOC108351	12	1	18	0
LOC108351	7	0	6	34
LOC108351	16	33	29	0
LOC108351	141	25	35	31
LOC108351	22	34	2	0
LOC108351	21	50	21	33
LOC108351	1	2	5	0
LOC108351	23	6	12	4
LOC108351	7	0	8	0
LOC108351	15	0	0	23
LOC108351	14	28	14	3
LOC108351	3	13	20	0
LOC108351	5	0	0	10
LOC108351	19	24	15	0
LOC108351	11	0	8	0
LOC108351	6	10	2	0
LOC108351	0	30	17	18
LOC108351	21	2	8	32
LOC108351	27	83	63	16
LOC108351	198	61	51	146
LOC108351	22	1	3	0
LOC108351	20	54	18	26
LOC108351	18	8	14	0

LOC108351	39	91	42	0
LOC108351	696	602	736	1357
LOC108351	0	1	35	49
LOC108351	54	24	48	38
LOC108351	25	15	15	41
LOC108352	0	3	24	0
LOC108352	408	1458	1759	247
LOC108352	67	50	60	45
LOC108352	15	28	56	19
LOC108352	2397	1458	972	2157
LOC108352	179	50	149	43
LOC108352	31	19	18	13
LOC108352	8	20	23	8
LOC108352	157	38	125	61
LOC108352	13	0	3	0
LOC108352	11	15	3	0
LOC108352	0	6	21	23
LOC108352	636	641	497	539
LOC108352	14	9	17	0
LOC108352	29	13	5	0
LOC108352	11	9	23	0
LOC108352	7	11	9	4
LOC108352	14	0	20	2
LOC108352	45	17	12	14
LOC108352	243	223	275	328
LOC108352	48	0	6	0
LOC108352	15	11	15	0
LOC108352	176	130	132	218
LOC108352	16	0	9	0
LOC108352	6	73	51	36
LOC108352	25	138	5	17
LOC108352	71	33	74	75
LOC108352	3	0	6	0
LOC108352	33	20	33	16
LOC108352	462	859	582	715
LOC108352	0	16	3	0
LOC108352	232	154	169	222
LOC108352	3	9	17	13
LOC108352	29	32	20	29
LOC108352	230	42	250	86
LOC108352	49	17	24	5
LOC108352	56	42	53	47
LOC108352	93	91	122	84
LOC108352	7	0	3	0
LOC108352	15	0	23	4
LOC108352	15	85	60	81
LOC108352	0	8	3	0

LOC108352	6	14	17	0
LOC108352	11	7	11	11
LOC108352	8	15	2	0
LOC108352	14	25	23	14
LOC108352	93	46	105	30
LOC108352	60	9	60	44
LOC108352	738	59	355	377
LOC108352	3	0	0	4
LOC108352	2	0	20	0
LOC108352	5	1	12	4
LOC108352	1	1	3	8
LOC108352	81	24	77	52
LOC108352	0	10	0	8
LOC108352	22	0	24	20
LOC108352	202	225	47	49
LOC108352	21	12	12	0
LOC108352	7	19	6	20
LOC108352	84	41	56	12
LOC108352	59	2	44	49
LOC108352	58	87	25	0
LOC108352	83	55	47	59
LOC108352	48	34	84	328
LOC108352	1	0	5	19
LOC108352	43	34	36	17
LOC108352	0	9	20	12
LOC108352	30	31	27	14
LOC108352	40	60	0	56
LOC108353	8	7	0	7
LOC108353	16	6	14	0
LOC108353	60	73	33	36
LOC108353	548	320	415	296
LOC108353	14	68	2	0
LOC108353	339	306	200	95
LOC108353	16	13	7	27
LOC108353	0	28	6	0
LOC108353	34	0	41	60
LOC108353	31	64	36	49
LOC108353	76	73	65	39
LOC108353	107	93	62	125
LOC108353	91	116	89	130
LOC24906	300	289	370	640
LOC257642	5	18	12	5
LOC259244	65	32	51	36
LOC288913	2802	3462	2660	2520
LOC288978	279	257	214	154
LOC290595	562	2493	741	388
LOC291276	8	2	0	16

LOC294154	6627	5865	4964	6415
LOC297756	6453	8731	4163	5766
LOC298139	1484	807	838	616
LOC298795	2	8	2	0
LOC299282	2535	3762	3740	1197
LOC299312	309	524	400	298
LOC300249	19677	16941	24201	18496
LOC300303	537	1080	1055	615
LOC301124	5087	5525	3703	4684
LOC301444	124	125	229	70
LOC302022	1	1	6	0
LOC302586	11	0	5	6
LOC303140	372	81	71	69
LOC303566	58	79	81	66
LOC304239	0	1	3	0
LOC306079	76	98	170	119
LOC306766	2050	1806	1682	1642
LOC308990	269	532	478	1225
LOC310902	33	2	11	0
LOC313310	2	2	2	3
LOC314140	709	463	706	565
LOC314407	28	63	104	111
LOC316717	20	7	9	4
LOC316820	0	13	3	0
LOC317456	95	159	60	133
LOC361016	14	21	8	7
LOC361346	79	145	120	273
LOC361635	1663	1647	1784	2357
LOC361646	304	244	334	275
LOC361985	3379	2819	2923	3642
LOC361990	4734	4541	4678	4672
LOC362863	59	45	41	87
LOC364556	97	52	105	45
LOC365085	29	13	18	35
LOC365837	30	2	17	45
LOC365839	108	15	51	133
LOC365949	5	0	17	22
LOC365985	12	1	12	20
LOC367191	1208	1771	2010	1984
LOC367858	981	898	1068	856
LOC497848	122	245	139	201
LOC497899	128	100	111	24
LOC497940	332	249	430	345
LOC498122	147	281	299	123
LOC498154	478	749	567	571
LOC498205	13	29	38	24
LOC498222	783	327	336	180

LOC498265	222	160	210	198
LOC498368	28	45	101	132
LOC498426	0	12	5	13
LOC498453	522	444	459	555
LOC498555	10747	9998	8489	6712
LOC498601	60	67	87	115
LOC498675	2517	2193	2133	2394
LOC498750	220	163	247	395
LOC499179	199	223	262	147
LOC499229	5	8	18	20
LOC499235	45	39	68	14
LOC499331	193	196	308	302
LOC499644	15	75	116	155
LOC499770	801	868	706	529
LOC500028	18	12	39	99
LOC500035	5	35	14	8
LOC500077	585	756	582	884
LOC500300	1480	568	1095	319
LOC500475	30	32	36	79
LOC500584	277	113	143	259
LOC500684	65	143	78	132
LOC500877	141	115	117	144
LOC500881	78	365	173	284
LOC500956	331	229	223	301
LOC500959	270	359	132	402
LOC501116	157	620	280	197
LOC501406	35	19	21	42
LOC501934	25	8	6	0
LOC502940	10	11	3	0
LOC56764	11	17	36	74
LOC619574	620	1129	1119	1971
LOC654482	272	134	193	77
LOC678760	706	1235	651	781
LOC678774	699	654	605	469
LOC678796	21	1	30	14
LOC679087	2	24	11	32
LOC679149	25	101	110	69
LOC679342	158	160	242	158
LOC679581	180	422	564	567
LOC679604	36	130	47	22
LOC679811	1313	1139	1199	831
LOC679881	459	870	576	691
LOC679894	345	284	382	253
LOC679924	40	44	59	78
LOC679963	558	252	326	376
LOC680017	22	31	75	69
LOC680039	1089	928	880	1089

LOC680142	1053	842	894	753
LOC680160	32	0	226	0
LOC680191	7	19	44	45
LOC680200	167	69	95	172
LOC680254	202	429	339	562
LOC680329	56	17	11	43
LOC680346	0	14	12	0
LOC680432	198	251	299	252
LOC680491	34	109	82	77
LOC680663	0	17	3	0
LOC680835	4	51	15	7
LOC680875	28	21	15	11
LOC680989	55	72	41	30
LOC681224	14	19	23	6
LOC681282	940	866	883	666
LOC681290	137	40	128	48
LOC681292	199	132	129	111
LOC681325	60	0	27	296
LOC681355	276	480	385	428
LOC681367	836	795	420	569
LOC681383	137	188	162	336
LOC681410	4668	3377	3743	3447
LOC681458	30213	14446	20740	23430
LOC681658	113	332	146	431
LOC682206	202	121	140	169
LOC682225	43	0	11	0
LOC682357	61	83	72	54
LOC682402	771	1552	600	952
LOC682571	111	135	110	99
LOC682812	930	1014	972	1148
LOC683212	0	19	12	0
LOC683420	131	323	284	197
LOC683674	610	626	510	676
LOC683897	1094	1153	796	1119
LOC683961	17	2	9	0
LOC684270	588	512	453	534
LOC684327	469	1504	605	614
LOC684509	2681	2276	2362	2238
LOC684557	142	287	164	307
LOC684828	152	52	77	81
LOC684988	9823	8035	4110	2700
LOC685025	36	114	110	89
LOC685067	561	232	456	421
LOC685152	558	1428	1140	1485
LOC685158	7	0	6	0
LOC685273	670	892	740	765
LOC685279	1	47	0	22

LOC685352	85	88	110	82
LOC685431	1315	1566	880	1310
LOC685574	35	23	57	161
LOC685590	61	149	113	91
LOC685619	418	563	447	450
LOC685655	23	18	24	0
LOC685668	1	40	11	44
LOC685680	589	120	588	109
LOC685700	241	80	263	85
LOC685849	71	45	30	40
LOC685881	1	0	8	0
LOC686013	2	10	27	3
LOC686087	69	14	65	16
LOC686129	1	15	21	36
LOC686143	213	0	1	17
LOC686151	16	31	41	15
LOC686774	69	192	159	148
LOC687399	10	49	9	3
LOC687560	5	0	3	0
LOC687679	4296	3738	2073	2491
LOC688064	415	690	691	618
LOC688286	45	57	48	86
LOC688390	13	15	3	0
LOC688452	302	257	397	237
LOC688459	272	158	188	117
LOC688583	67	56	71	67
LOC688637	901	1073	784	961
LOC688655	197	485	333	474
LOC688672	3472	3925	2869	3369
LOC688754	665	1007	691	841
LOC688765	73	42	45	96
LOC688812	25	98	117	137
LOC688815	966	1379	593	1036
LOC688869	105	143	134	114
LOC688906	2	31	23	0
LOC689130	28309	36132	37580	48792
LOC689230	13152	6850	2451	1731
LOC689271	1812	1426	952	1448
LOC689316	92	54	69	67
LOC689346	10	3	3	5
LOC689439	42	80	89	61
LOC689488	14	23	83	142
LOC689574	7412	5259	5322	5816
LOC689757	70	115	107	447
LOC689800	31	25	15	56
LOC689899	5278	3411	2273	1482
LOC689955	30	26	3	10

LOC68995‹	713	356	629	653
LOC69000(	788	863	957	995
LOC69003‹	299	322	292	247
LOC69012(	54	9	6	15
LOC69012‹	39	19	33	11
LOC690131	65	25	24	12
LOC69015‹	362	259	274	244
LOC690171	1230	1549	1067	1337
LOC69018‹	187	221	138	141
LOC69018‹	179	221	295	312
LOC69027‹	94	18	59	13
LOC69028‹	0	3	6	0
LOC69031‹	69	0	65	0
LOC69032‹	17	44	33	10
LOC69032‹	250	51	278	207
LOC69042‹	310	371	348	254
LOC69061‹	995	1087	1144	2583
LOC69082‹	14	12	26	27
LOC69086‹	251	330	128	175
LOC690871	650	715	394	388
LOC69091‹	0	33	20	1
LOC69108‹	184	28	193	82
LOC69111‹	884	655	972	786
LOC69113‹	16	12	18	0
LOC691141	555	644	636	1150
LOC69114‹	431	581	497	368
LOC69115‹	307	310	336	248
LOC69116‹	19	0	17	25
LOC69117(	756	512	569	653
LOC69128(	651	497	433	515
LOC69129(	53	131	99	196
LOC69130‹	1142	1251	1619	1606
LOC69135‹	92	120	47	181
LOC69139‹	70	81	138	64
LOC69141‹	3	81	18	20
LOC69142‹	178	90	66	106
LOC69148‹	21	0	21	1
LOC69165‹	480	502	445	456
LOC69167(	16	0	12	4
LOC69167‹	117	132	53	101
LOC69171‹	3143	2888	2287	3024
LOC69180‹	2490	1852	1775	2165
LOC691921	524	333	355	364
LOC691931	354	335	339	499
LOC69199‹	279	268	280	332
Lonp1	976	1436	868	1168
Lonp2	1882	1438	1927	1587

Lonrf1	343	78	345	317
Lonrf2	0	18	14	11
Lonrf3	43	44	50	165
Lor	466	323	30	80
Lox	2161	5206	3652	6908
Loxl1	3267	4692	3942	4335
Loxl2	3847	12902	8222	12172
Loxl3	82	237	122	154
Loxl4	1641	1554	1586	1690
Lpar1	781	1023	1014	1566
Lpar2	105	69	62	0
Lpar3	320	178	244	138
Lpar4	22	111	50	0
Lpar6	636	648	624	960
Lpcat1	704	994	794	1480
Lpcat2	1030	344	1013	1037
Lpcat3	1562	2016	1871	2288
Lpcat4	233	225	169	64
Lpgat1	1278	1719	1881	2391
Lpin1	661	747	457	808
Lpin2	696	499	486	647
Lpin3	676	843	611	477
Lpl	4294	2876	6163	9279
Lpo	67	14	42	0
Lpp	1851	2089	2364	2098
Lppos	218	156	169	117
Lpxn	425	367	487	1220
Lrat	397	726	739	275
Lrba	443	317	414	354
Lrch1	424	396	561	396
Lrch3	584	760	834	875
Lrch4	490	799	512	976
Lrcol1	70	16	99	18
Lrfn1	95	89	101	100
Lrfn3	39	79	68	75
Lrfn4	230	405	226	303
Lrg1	951	1696	1724	1457
Lrguk	19	12	18	19
Lrif1	119	124	144	155
Lrig1	191	391	396	181
Lrig2	626	479	594	786
Lrig3	821	362	1149	657
Lrmp	73	72	70	65
Lrp1	6677	13640	8334	11228
Lrp10	4737	5147	4834	5913
Lrp11	433	662	605	562
Lrp12	369	164	311	237

Lrp2	184	134	71	32
Lrp3	39	80	41	11
Lrp4	553	604	636	426
Lrp5	513	755	636	389
Lrp6	68	496	223	350
Lrp8	132	250	202	229
Lrpap1	1603	2464	2153	2635
Lrpprc	2354	2268	2291	1953
Lrr1	22	23	39	25
Lrrc1	1207	526	957	387
Lrrc14	532	375	519	362
Lrrc14b	895	707	673	670
Lrrc15	4845	8107	8824	17005
Lrrc17	831	3392	2638	4493
Lrrc18	13	0	2	4
Lrrc2	936	681	688	709
Lrrc20	1421	807	725	1163
Lrrc24	820	986	867	565
Lrrc25	419	898	785	1276
Lrrc27	26	30	41	75
Lrrc28	521	279	382	348
Lrrc29	12	45	47	25
Lrrc3	77	214	105	105
Lrrc30	65	49	84	108
Lrrc32	655	1522	992	1022
Lrrc36	34	19	8	0
Lrrc39	935	659	376	807
Lrrc3b	113	32	72	94
Lrrc4	132	45	187	33
Lrrc40	460	509	594	813
Lrrc41	799	1062	678	968
Lrrc42	1548	1332	1494	1397
Lrrc46	33	33	17	23
Lrrc47	800	1041	851	606
Lrrc49	354	352	424	488
Lrrc4c	0	8	29	16
Lrrc51	59	106	32	124
Lrrc56	100	38	63	15
Lrrc58	26	54	74	82
Lrrc59	2449	3076	2970	3999
Lrrc61	324	288	275	372
Lrrc66	6	5	14	31
Lrrc71	1283	1289	1503	1166
Lrrc73	1600	2071	1494	1745
Lrrc75a	465	282	459	237
Lrrc8a	2573	2620	2792	3191
Lrrc8b	565	448	396	564

Lrrc8c	793	1201	1231	1510
Lrrc8d	375	643	537	759
Lrrc8e	57	141	47	17
Lrrcc1	470	104	364	461
Lrrfip1	2509	2844	2737	2653
Lrrfip2	1893	2859	2219	2591
Lrrk1	737	680	728	1190
Lrrk2	151	469	269	609
Lrrn1	15	208	42	77
Lrrn2	17	25	26	16
Lrrn3	24	108	63	16
Lrrn4cl	774	1270	716	844
LRRTM1	4	2	3	0
Lrrtm2	2	3	30	0
Lrrtm3	9	18	14	0
Lrsam1	0	231	120	175
Lrtm2	0	5	9	3
Lrtomt	70	104	99	67
Lrwd1	370	551	435	475
Lsamp	16	14	87	40
Lsg1	469	600	534	662
Lsm1	568	860	621	1135
Lsm10	732	366	513	607
Lsm11	330	455	340	209
Lsm12	4218	3072	3184	4026
Lsm14a	2435	1864	1816	1885
Lsm14b	1448	803	700	878
Lsm2	875	1036	790	817
Lsm3	2441	1824	2633	2343
Lsm4	1664	1885	1247	1424
Lsm5	508	482	489	480
Lsm6	123	165	77	42
Lsm7	675	1018	524	608
Lsm8	987	1323	1547	1400
Lsmem1	630	452	241	361
Lsmem2	825	1154	1026	1301
Lsp1	4402	6399	5258	7943
Lsr	1767	942	1655	623
Lss	206	239	102	197
Lst1	370	439	648	875
Lta4h	1768	2389	2258	2235
Ltb4r	290	290	257	112
Ltb4r2	100	19	38	31
Ltbp1	2842	1949	2634	1793
Ltbp2	1377	1106	1560	831
Ltbp3	413	836	894	595
Ltbp4	2583	2596	2176	946

Ltbr	2328	3544	2458	2554
Ltc4s	202	143	136	63
Ltk	29	47	6	36
Ltn1	1192	662	961	1043
Ltv1	1414	1206	1601	1093
Luc7l	1420	862	1464	1206
Luc7l2	2920	1721	2048	2765
Luc7l3	1211	878	936	739
Lum	9433	15981	15693	44995
Lurap1	45	59	38	62
Lurap1l	1059	510	900	255
Luzp1	1230	1508	1414	1279
Lvrn	123	169	140	123
Lxn	1684	1706	1751	2147
Ly49s6	22	8	6	4
Ly6d	1680	1994	537	424
Ly6e	8761	9189	10019	13420
Ly6g6c	2837	669	1432	881
Ly6g6d	67	11	90	18
Ly6g6e	52	18	17	0
Ly6h	5	12	0	2
Ly6l	10	65	59	58
Ly86	712	450	567	973
Ly96	637	911	891	1490
Lyar	890	1121	853	800
Lyc2	17	14	11	53
Lyl1	5	74	18	47
Lyn	1568	1824	2112	3734
Lynx1	743	384	403	447
Lypd1	36	9	41	16
Lypd3	7380	3997	6355	2237
Lypd6	19	14	8	48
Lypd6b	492	119	251	75
Lypla1	1334	1359	1447	1426
Lypla2	639	786	481	461
Lyplal1	594	380	361	222
Lyrm1	426	418	299	319
Lyrm2	1518	1261	1515	1481
Lyrm5	939	528	657	712
Lyrm7	499	419	298	312
Lyrm9	153	230	158	335
Lysmd1	116	266	108	168
Lysmd2	356	475	388	445
Lysmd3	0	13	2	16
Lysmd4	410	320	272	271
Lyst	70	122	146	67
Lyve1	1061	4034	1450	1095

Lyz2	42632	77746	56960	213786
Lzic	855	838	883	897
Lztfl1	505	352	591	389
Lztr1	1323	1476	1256	1962
Lzts1	234	173	229	146
Lzts2	695	818	824	672
Lzts3	79	81	84	63
Mab21l1	6	46	17	41
Mab21l3	217	68	342	169
Macc1	404	136	310	136
Macf1	5451	4732	6089	3960
Macrod1	1325	1040	639	807
Macrod2	12	12	0	21
Mad1l1	201	367	134	324
Mad2l1	309	501	415	240
Mad2l1bp	504	603	480	591
Mad2l2	708	1252	1139	962
Madd	400	510	445	599
Maea	1854	1818	1812	1924
Maf	1113	2023	1654	2075
Maf1	2950	2821	2738	2956
Mafa	527	607	218	232
Mafb	1631	1992	1986	2806
Maff	47	227	100	99
Mafg	2069	2264	1709	2140
Mafk	601	553	530	357
Mag	14	0	2	2
Maged1	2418	4385	3838	4710
Maged2	999	2459	1174	1488
Magee1	38	30	75	4
Magee2	0	8	5	0
Mageh1	207	268	295	404
Magi1	583	637	603	310
Magi3	213	291	250	446
Magix	222	62	78	161
Magoh	3970	2368	3235	3424
Magohb	165	124	199	296
Magt1	160	67	134	178
Mak16	566	682	530	516
Mal	96	68	166	191
Mal2	331	106	355	150
Mall	950	927	978	605
Malsu1	919	736	803	795
Malt1	311	271	302	354
Mamdc2	81	658	227	481
Mamdc4	66	34	89	19
Maml1	41	40	60	33

Maml2	487	429	740	292
Maml3	12	4	9	6
Mamstr	419	1029	251	94
Man1a1	1410	1760	1393	1835
Man1a2	1278	900	1220	1148
Man1b1	233	767	330	512
Man1c1	795	1246	928	1337
Man2a1	2650	2243	3140	3247
Man2a2	1809	2155	1676	1562
Man2b1	2762	3758	3149	5264
Man2b2	955	2118	1315	2496
Man2c1	724	920	826	772
Manba	554	584	671	915
Manbal	2612	1640	1849	1914
Manea	352	270	375	291
Maneal	9	28	15	0
Manf	4535	5772	5167	6328
Mansc1	248	247	208	336
Mansc4	1	0	26	20
Maoa	995	909	964	831
Maob	99	98	123	141
Map10	8	13	20	73
Map1a	1440	2286	1651	1661
Map1b	515	988	657	640
Map1lc3a	4786	4594	3304	3566
Map1lc3b	6072	6054	5708	5845
Map1s	265	473	289	348
Map2	222	237	83	50
Map2k1	1439	1899	1693	2315
Map2k2	1606	2382	1253	1693
Map2k4	2004	1179	1763	1676
Map2k5	818	674	651	697
Map2k6	416	177	179	294
Map2k7	7	38	21	8
Map3k1	0	7	0	10
Map3k10	95	95	44	51
Map3k12	662	474	577	325
Map3k13	44	26	21	32
Map3k14	314	232	223	223
Map3k2	41	27	35	44
Map3k3	31	351	455	687
Map3k4	638	591	772	624
Map3k5	732	381	749	510
Map3k6	491	915	495	396
Map3k7	343	153	215	103
Map3k7cl	1028	756	504	1082
Map3k8	338	151	299	280

Map3k9	107	188	172	208
Map4	8700	11400	10048	11208
Map4k1	311	300	220	317
Map4k2	118	238	237	163
Map4k3	651	696	790	816
Map4k4	2487	3112	3337	3199
Map4k5	932	641	843	997
Map6	1378	1538	1629	1899
Map7	1363	678	1300	550
Map7d1	3492	6550	4086	4802
Map7d2	9	33	38	20
Map7d3	22	40	32	0
Map9	146	122	153	119
Mapk1	2692	2890	2789	3673
Mapk11	103	105	72	36
Mapk12	2213	1624	931	1419
Mapk13	2433	1230	1347	616
Mapk14	2056	2377	1947	2064
Mapk1ip1	742	597	602	530
Mapk1ip1l	2052	2272	2081	2728
Mapk3	2673	3212	3298	2974
Mapk6	2068	2650	2467	2489
Mapk8	840	657	670	713
Mapk8ip1	536	645	456	515
Mapk8ip3	507	747	394	441
Mapk9	924	1134	1250	1241
Mapkap1	830	1068	743	1010
Mapkapk2	3614	4216	2991	4484
Mapkapk3	2165	1898	1655	1474
Mapkapk5	1771	1940	1577	1935
Mapkbp1	48	116	119	88
Mapre1	4876	4549	4667	4981
Mapre2	2123	2527	1839	3763
Mapre3	1142	1562	1023	863
Marcks	6355	2297	3719	5343
Mark1	288	171	337	124
Mark2	948	936	852	510
Mark3	2104	1747	1890	1800
Mark4	1127	991	1283	752
Mars	2128	2748	2574	2192
Marveld1	1909	3000	2162	4216
Marveld2	1	36	12	47
Mas1l	13	0	9	17
Masp1	2335	2515	2329	3507
Masp2	72	28	164	82
MAST1	1	10	3	0
Mast2	1448	1986	1480	1689

Mast3	537	663	537	669
Mastl	206	248	194	176
Mat2a	4301	3206	2642	2590
Mat2b	978	1121	1232	1370
Matk	204	478	387	1330
Matn2	1428	2464	1232	938
Matn4	14	54	12	1
Matr3	566	452	457	345
Matr3-ps2	112	119	42	49
Mau2	1425	1199	1548	1427
Mavs	678	745	639	638
Max	1709	1378	1192	1534
Maz	3318	3711	3715	4542
Mb	95182	40393	49226	63587
Mb21d1	30	60	27	43
Mb21d2	722	574	605	399
Mbd2	3801	4053	4894	4634
Mbd3	782	1437	694	1039
Mbd4	194	188	215	192
Mbd5	212	229	275	276
Mbd6	98	220	114	42
Mbip	324	127	205	351
Mblac1	233	342	342	525
Mblac2	15	37	11	8
Mbnl1	204	145	205	126
Mbnl2	3139	1185	1235	2543
Mbnl3	732	252	448	178
Mboat1	924	897	1080	890
Mboat2	93	113	137	32
Mbp	264	170	512	550
Mbtd1	419	351	471	401
Mbtps1	2756	2887	2993	2858
Mbtps2	179	137	268	134
Mc5r	206	69	75	210
Mcam	2157	6495	4494	4500
Mcat	400	422	322	341
Mcc	243	86	108	99
Mccc1	754	668	778	729
Mccc2	944	675	858	883
Mcee	1449	969	1267	1490
Mcemp1	328	380	239	399
Mcf2l	1133	764	695	505
Mcfd2	1166	1389	1470	1601
Mchr1	44	48	33	2
Mcl1	3285	2219	3053	2529
Mcm10	140	310	224	230
Mcm2	1026	1377	985	1036

Mcm3	949	1480	1210	1028
Mcm3ap	704	803	608	843
Mcm4	589	682	797	722
Mcm5	801	1256	698	715
Mcm6	836	1378	1154	1084
Mcm7	1783	2224	1994	1531
Mcm8	44	70	45	36
Mcm9	344	401	343	449
Mcmbp	992	1364	1216	1343
Mcmdc2	48	31	20	21
Mcoln1	857	782	674	758
Mcoln2	184	128	123	311
Mcoln3	16	29	57	167
Mcph1	12	26	21	0
Mcpt1l1	1143	1005	1023	243
Mcpt9	61	0	8	2
Mcrip1	1728	2070	1520	1573
Mcrip2	190	197	125	150
Mcrs1	672	1165	719	909
Mctp1	41	72	30	15
Mctp2	118	136	135	99
Mcts1	1833	1025	1189	1602
Mcts2	928	675	799	520
Mcu	645	1324	716	819
Mcub	49	106	35	123
Mcur1	52	62	99	50
Mdc1	755	617	859	724
Mdfi	111	202	99	71
Mdfic	891	471	466	1131
Mdga1	66	44	29	30
Mdh1	13170	9300	8445	9912
Mdh2	9910	8952	5438	7929
Mdk	811	367	778	1440
Mdm1	185	121	167	300
Mdm2	1821	1962	1613	1717
Mdm4	1968	1308	2143	1551
Mdn1	439	552	459	475
Mdp1	853	882	590	839
Me1	7020	3626	4103	5450
Me2	689	1123	1300	1322
Me3	199	109	89	155
Mea1	5627	5477	4765	4452
Meaf6	608	626	543	548
Mecp2	705	669	832	872
Mecr	386	607	426	419
Med1	1235	1304	1237	1261
Med10	1232	878	1223	1105

Med11	1019	717	932	637
Med12	404	803	506	571
Med12l	6	2	29	0
Med13	925	662	910	1074
Med13l	799	847	852	666
Med14	429	206	445	373
Med15	1387	2056	1655	1578
Med16	842	710	630	472
Med17	309	287	275	374
Med18	450	354	325	364
Med19	2973	1535	2548	2003
Med20	1012	772	752	838
Med21	886	890	853	864
Med23	493	730	639	739
Med24	816	842	826	698
Med25	3005	3797	2594	2509
Med26	562	258	366	264
Med27	482	642	376	472
Med28	1898	1537	1741	1826
Med29	787	894	548	901
Med30	961	670	882	744
Med31	707	513	486	524
Med4	642	837	677	662
Med6	875	609	820	478
Med7	580	351	644	538
Med8	1789	1447	1614	1717
Med9	337	456	394	370
Medag	2152	3891	2896	3063
Mef2a	1740	2162	2224	2000
Mef2c	6768	6100	4980	5522
Mef2d	1960	3249	2288	2960
Mefv	228	208	275	277
Megf10	104	472	90	76
Megf11	153	102	132	122
Megf8	18	130	45	27
Megf9	173	98	181	143
Meiob	26	13	27	43
Meis1	377	265	381	410
Meis3	252	286	510	1293
Melk	277	442	340	252
Memo1	1553	1596	1089	1546
Men1	859	713	664	770
Meox1	58	306	125	223
Meox2	364	419	381	376
Mertk	35	85	48	160
Mesdc1	1343	996	1232	1400
Mesdc2	1930	2547	2297	3030

Mest	614	188	66	208
Met	913	887	1211	770
Metap1	1676	1775	1607	1576
Metap1d	157	211	159	82
Metap2	1769	769	835	1034
Metrn	23	47	26	22
Metrnl	238	497	266	548
Mettl1	366	384	509	344
Mettl10	388	333	382	493
Mettl11b	19	23	32	48
Mettl13	464	669	555	602
Mettl14	709	767	957	565
Mettl15	70	26	45	15
Mettl16	528	630	533	426
Mettl17	281	207	212	161
Mettl18	316	197	295	170
Mettl21a	398	198	299	258
Mettl21b	0	2	0	7
Mettl21c	182	46	239	128
Mettl22	785	594	397	492
Mettl23	2494	1098	1705	1786
Mettl25	173	166	149	218
Mettl26	527	298	439	392
Mettl2b	736	460	498	492
Mettl3	2219	1689	2276	2195
Mettl4	44	71	44	114
Mettl5	1632	976	1368	1721
Mettl6	1481	1803	2358	1890
Mettl8	257	231	232	179
Mettl9	1815	1311	1249	2476
Mex3a	563	214	737	448
Mex3b	32	31	71	41
Mex3c	858	602	620	530
Mex3d	182	207	311	319
Mfap1a	1447	1674	1682	1823
Mfap2	325	438	474	1476
Mfap3	16	47	12	108
Mfap3l	341	422	167	371
Mfap5	3787	11487	5469	5894
Mfge8	8795	10102	11108	8606
Mfhas1	1475	846	1324	1086
Mfn1	2538	1990	2495	2221
Mfng	481	704	465	599
Mfsd1	3412	3965	3537	5788
Mfsd10	267	372	245	389
Mfsd11	1239	1714	1381	1740
Mfsd12	15	133	29	22

Mfsd13a	318	210	170	257
Mfsd14b	741	609	650	947
Mfsd2a	1451	724	898	720
Mfsd3	301	364	333	204
Mfsd4	0	27	20	14
Mfsd4b	5	57	24	10
Mfsd5	1137	1473	1162	1213
Mfsd6	1383	548	954	663
Mfsd7	224	279	305	184
Mfsd8	388	321	355	418
Mfsd9	311	292	235	414
Mga	104	188	166	163
Mgarp	14	1	9	10
Mgat1	1846	2249	2079	2307
Mgat2	795	928	891	1205
Mgat3	148	182	317	137
Mgat4a	505	475	382	410
Mgat4b	641	1315	1081	1017
Mgat5	46	17	32	1
MGC10556	93	57	29	180
MGC10564	1701	1157	1783	1734
MGC10882	77	31	38	96
MGC10934	42	7	3	0
MGC11442	14	23	45	0
MGC11612	193	108	284	153
MGC93861	7	2	14	61
MGC94335	970	758	829	834
MGC95208	143	197	161	206
MGC95210	129	184	150	185
Mgea5	3869	2018	3151	2834
Mgll	1089	1946	1491	1607
Mgme1	159	272	113	146
Mgmt	603	528	396	388
Mgp	10805	14638	17993	9977
Mgrn1	1258	2133	1316	1790
Mgst1	8895	4308	5376	7568
Mgst2	2787	1304	2422	2265
Mgst3	1002	910	641	689
Mia	8	0	15	8
Mia3	1555	1985	1861	2471
Miat	247	785	536	805
Mib1	988	1071	1083	1125
Mib2	129	158	105	149
Mical1	552	1063	927	1014
Mical2	1283	1673	2069	2035
Mical3	2638	1224	2747	1075
Micalcl	330	166	221	109

Micall1	81	159	139	96
Micall2	233	452	262	392
Micu1	966	2187	1180	1362
Micu2	2502	2116	2389	2053
Micu3	41	142	75	63
Mid1	894	294	566	625
Mid1ip1	1262	850	793	1177
Mid2	496	316	475	398
Midn	3731	4001	3856	3083
Mief1	1044	1265	1026	1296
Mief2	1406	2063	1247	1360
Mien1	16	51	8	14
Mier1	1244	1229	1214	1355
Mier2	153	174	63	75
Mier3	223	97	289	177
Mif4gd	1265	1166	986	864
Miga1	95	133	110	7
Miga2	594	540	399	455
Mill1	726	259	487	172
Milr1	175	196	129	457
Mina	239	240	312	243
Mink1	2344	1878	2221	1581
Minos1	4945	4345	3653	3872
Minpp1	141	262	184	266
Mios	359	403	316	480
Mip	8	1	0	4
Mipep	996	1097	1047	973
Mir101a	75	68	78	29
Mir133a	119	41	20	61
Mir133b	592	799	737	260
Mir142	180	81	87	170
Mir155	93	0	42	27
Mir22	596	793	772	931
Mir221	142	69	96	71
Mir223	104	149	83	211
Mir3561	22	73	12	31
Mir3582	110	28	5	55
Mir568	178	559	471	354
Mir6314	305	50	231	50
Mir6315	125	78	63	130
Mirlet7i	167	119	95	60
Mis12	741	977	1198	1137
Mis18a	24	33	26	40
Mis18bp1	101	97	72	81
Misp	515	942	429	607
Mitd1	644	518	509	496
Mitf	858	508	584	1023

Mk1	448	738	581	903
Mki67	1895	2136	2737	2241
Mkks	1681	1739	1458	2171
Mkl1	295	591	304	307
Mkl2	5	13	30	0
Mkln1	2043	1772	1980	2259
Mknk1	707	817	657	1017
Mknk2	7030	6623	6426	6046
Mkrn1	2588	1510	2474	2124
Mkrn2	972	1460	1221	1452
Mkrn3	29	9	26	25
Mks1	224	186	330	334
Mlec	3096	5613	4199	5946
Mlf1	2641	1084	575	1535
Mlf2	5384	5753	4425	5032
Mlh1	390	466	299	290
Mlh3	363	296	342	283
Mlip	607	605	581	556
Mlkl	946	846	821	727
Mllt1	985	1261	909	810
Mllt10	369	550	530	339
Mllt3	1665	729	841	956
Mllt6	312	377	689	591
Mlph	43	25	9	6
Mlst8	736	455	635	483
Mlx	1559	1271	1372	1446
Mlxip	66	111	72	58
Mlxipl	56	39	35	14
Mlycd	855	647	513	460
Mmaa	313	394	229	458
Mmab	277	257	248	267
Mmachc	478	377	339	273
Mmadhc	2375	1798	1757	1851
Mmd	677	495	599	557
Mme	358	224	388	418
Mmgt1	1598	1111	1526	1359
Mmgt2	292	184	229	270
Mmp10	707	479	119	87
Mmp12	4810	3803	6826	18180
Mmp13	7120	4767	4270	4065
Mmp14	11295	17399	18384	36975
Mmp15	573	1325	563	532
Mmp16	174	61	149	234
Mmp19	1719	2880	2871	3937
Mmp1b	7	0	6	89
Mmp2	21388	34349	25236	56121
Mmp23	539	1388	1109	2382

Mmp24	986	826	581	705
Mmp27	260	145	105	160
Mmp3	2241	2872	1518	1620
Mmp7	92	138	286	902
Mmp8	105	88	77	242
Mmp9	5834	7136	4584	6330
Mmrn1	73	145	116	72
Mmrn2	819	1151	1094	771
Mms19	492	639	532	655
Mms22l	305	362	307	303
Mn1	405	535	199	287
Mnat1	958	1052	933	1009
Mnd1	142	207	217	154
Mnda	1437	1891	1732	3118
Mns1	112	125	89	75
Mnt	582	551	683	759
Mob1a	3468	2904	2889	4173
Mob1b	44	55	63	52
Mob2	894	849	782	764
Mob3a	1669	1697	1587	1828
Mob3b	0	1	3	23
Mob3c	282	367	463	561
Mob4	1508	1129	1140	1383
Mobp	11004	16317	10943	12988
Mocos	735	489	608	576
Mocs1	1073	1858	1104	1244
Mocs2	1631	1856	1029	1946
Mocs3	167	128	185	158
Mog	69	165	175	116
Mogat1	61	9	24	13
Mogat2	34	4	9	35
Mok	48	170	32	14
Mon1a	225	350	206	202
Mon1b	497	605	510	442
Mon2	773	938	1174	1151
Morc2	814	810	867	936
Morc3	510	511	527	615
Morc4	398	570	564	560
Morf4l1	1237	1667	2049	2045
Morf4l2	6883	6026	5967	8371
Morn1	53	46	60	50
Morn2	145	67	150	37
Morn3	0	9	15	16
Morn4	139	163	119	112
Morn5	1	0	3	0
Mospd1	129	137	78	132
Mospd2	261	355	370	333

Mospd3	621	842	603	799
Mov10	1005	701	1011	818
Moxd1	1704	1535	1503	629
Mpa2l	58	51	68	52
Mpc1	2892	2574	2201	3510
Mpc1l	411	173	245	157
Mpc2	5294	2761	2805	4033
Mpdu1	1895	1610	1913	1932
Mpdz	373	423	277	347
Mpeg1	3526	3106	5128	12941
Mpg	379	444	227	448
Mphosph1(	864	902	1008	900
Mphosph6	842	1159	790	1067
Mphosph8	808	797	870	755
Mphosph9	93	83	113	101
Mpi	1544	1866	1693	1433
Mplkip	330	174	245	184
Mpnd	1748	2080	1586	1499
Mpo	17	0	39	0
Mpp1	356	489	355	221
Mpp2	90	85	98	74
Mpp3	185	481	217	239
Mpp4	2	0	11	0
Mpp5	523	317	382	548
Mpp6	828	786	661	832
Mpp7	53	27	86	0
Mppe1	563	307	334	468
Mpped2	81	41	45	31
Mprip	3298	4490	4441	4588
Mpst	1789	1317	1049	999
Mpv17l	1502	936	1082	1520
Mpv17l2	987	910	803	841
Mpz	856	735	2725	2279
Mpzl1	1055	1854	1774	2127
Mpzl2	1678	706	1211	325
Mpzl3	3	6	5	11
Mr1	290	226	254	287
Mrap	16	0	3	0
Mrap2	0	17	11	0
Mras	423	1137	846	2072
Mrc1	1888	4505	4077	6309
Mrc2	1623	2463	1947	3015
Mre11a	85	166	126	116
Mreg	982	757	1104	1126
Mrfap1	15225	11770	15256	14329
Mrgprb3	73	73	84	241
Mrgprf	447	1641	642	1077

Mrgprx3	33	8	8	36
Mri1	339	351	323	295
Mrln	1025	1518	1064	660
Mrm1	554	355	409	269
Mrm2	149	261	187	108
Mrm3	329	279	229	349
Mroh1	14	0	11	0
Mroh2a	35	13	17	0
Mroh6	88	74	179	39
Mroh7	41	19	57	3
Mroh8	0	21	9	25
Mrpl1	10	26	6	0
Mrpl10	1304	1410	1381	1445
mrpl11	1595	1703	1586	1610
Mrpl12	2697	3479	2227	2034
Mrpl13	2952	2619	3036	2600
Mrpl14	1368	1515	1031	892
Mrpl15	3076	2929	2502	3159
Mrpl16	1957	1554	1304	1455
Mrpl17	6398	6329	5785	6215
Mrpl18	2278	1391	1065	1907
Mrpl19	910	787	742	911
Mrpl2	366	533	305	322
Mrpl20	3045	2890	2619	3122
Mrpl21	1297	1313	1002	1101
Mrpl22	724	880	823	707
Mrpl23	2884	3068	2567	2528
mrpl24	2144	2319	2209	2209
Mrpl27	2334	2754	2073	2422
Mrpl28	784	1171	701	698
Mrpl3	1476	1646	1345	1684
Mrpl32	1244	1237	1471	998
Mrpl34	1365	920	1074	779
Mrpl35	2472	1803	2152	2025
Mrpl37	1687	1877	1742	1264
Mrpl38	724	1109	647	728
Mrpl39	660	531	457	490
Mrpl4	891	967	661	666
Mrpl40	496	621	307	331
Mrpl41	2145	1848	1265	1369
Mrpl42	1506	1324	1417	1177
Mrpl43	474	828	554	670
Mrpl44	1101	1279	1166	1176
Mrpl45	1718	2306	1480	1205
Mrpl46	946	868	736	723
Mrpl47	1199	844	748	949
Mrpl48	2557	2633	2310	2471

Mrpl49	97	319	114	243
Mrpl50	1331	1105	835	1068
Mrpl51	2532	2702	2016	2509
Mrpl52	3824	3080	3423	3392
Mrpl54	2620	3056	2123	2567
Mrpl55	992	1113	910	673
Mrpl57	1836	1137	1282	732
Mrpl58	1821	1861	1682	1987
mrpl9	2164	1957	1863	2116
Mrps11	957	1045	627	592
Mrps12	1828	1266	1647	1107
Mrps14	1689	1767	1572	1773
Mrps15	3775	2536	2829	3266
Mrps16	2171	2083	2034	2030
Mrps17	1551	1749	1493	1440
Mrps18a	1561	1629	1291	1203
Mrps18b	2151	2106	2088	1780
Mrps18c	1387	1004	1517	1305
Mrps2	1111	1022	1351	1337
Mrps21	2779	2357	2379	2298
Mrps22	720	792	506	880
Mrps23	2455	2448	2380	2095
Mrps24	1856	1574	1225	1187
Mrps25	1236	1806	1381	1286
Mrps26	2107	1979	2391	1720
Mrps27	725	715	569	817
Mrps28	1318	1275	1049	880
Mrps30	2120	1429	1238	1354
Mrps31	628	640	639	652
Mrps33	2609	1427	2171	1606
Mrps34	1796	1734	1356	1016
Mrps35	1068	1065	1059	747
Mrps36	2005	1117	1583	2082
Mrps5	628	815	682	771
Mrps6	1228	1153	1041	1224
Mrps7	2140	2842	1810	2131
Mrps9	1157	1424	1184	1153
Mrrf	629	624	872	569
Mrs2	404	326	400	539
Mrto4	1234	1728	1256	1383
Mrvi1	209	341	463	315
Ms4a12	17	0	48	35
Ms4a14	55	17	90	23
Ms4a2	106	47	89	10
Ms4a4a	48	191	119	158
Ms4a6a	1084	1062	812	1608
Ms4a6b	64	26	42	236

Ms4a6c	103	134	153	378
Ms4a7	2349	1210	2085	2291
Ms4a8	26	17	12	4
Msantd1	22	23	38	48
Msantd2	257	243	253	262
Msantd3	744	1064	894	1079
Msantd4	711	571	755	922
Msc	103	172	125	330
Msh2	477	605	345	316
Msh3	191	248	159	141
Msh5	40	68	35	18
Msh6	1695	1157	1626	1486
Msi1	0	5	3	0
Msi2	1440	1675	1524	964
Msl1	2926	2919	3123	3202
Msl2	1224	892	970	1053
Msl3l2	184	226	84	214
Msln	370	2844	1052	925
Mslnl	14	29	12	10
Msmo1	3629	1746	2513	2696
Msn	10786	18259	17042	18966
Msr1	564	1692	1008	2264
Msra	53	79	45	40
Msrb1	4057	3026	2322	3031
Msrb2	254	255	236	405
Msrb3	1494	1098	1025	1037
Mss51	1191	162	211	963
Mst1	41	18	9	3
Mst1r	14	48	29	1
Mstn	54	10	8	30
Msto1	490	617	392	486
Msx1	151	187	129	177
Msx2	323	151	439	86
Mt1	2491	3603	2657	2066
Mt1m	1659	2997	1425	1443
Mt2A	2310	3559	1622	1347
Mt3	21	902	102	1014
Mt4	448	33	348	18
Mta1	962	1220	1037	870
Mta2	3401	2774	3919	2790
Mta3	998	967	933	723
Mtap	1409	1112	1485	1363
Mtbp	41	150	60	77
Mtch1	4250	6139	4235	4406
Mtch2	5645	5047	4980	5776
Mtcl1	76	261	187	211
Mtdh	148	178	122	254

Mterf1	165	332	319	369
Mterf2	169	81	96	106
Mterf3	709	368	513	720
Mterf4	871	842	790	814
Mtf1	18	33	45	2
Mtf2	550	351	426	456
Mtfmt	405	463	459	326
Mtfp1	214	286	135	271
Mtfr1	304	290	349	384
Mtfr1l	4086	4127	3269	4366
Mtfr2	233	304	256	259
Mtg1	816	773	674	547
Mtg2	959	986	796	681
Mthfd1	996	791	1079	898
Mthfd1l	244	410	320	200
Mthfd2	344	463	421	568
Mthfd2l	32	63	54	37
Mthfr	304	248	254	542
Mthfs	701	454	665	719
Mthfsd	183	137	104	106
Mtif2	681	379	442	396
Mtif3	503	522	288	371
Mtm1	305	530	351	347
Mtmr1	507	644	501	530
Mtmr10	1051	637	724	1023
Mtmr11	2152	1659	2239	1719
Mtmr12	340	593	463	407
Mtmr2	2329	2044	2132	2871
Mtmr3	1248	1465	1434	1668
Mtmr4	872	730	718	882
Mtmr6	122	315	332	380
Mtmr7	63	150	66	118
Mtmr9	48	106	108	53
Mto1	418	393	445	468
Mtor	1643	1616	1381	1289
Mtpap	638	713	481	330
Mtpn	2478	2395	2389	3207
Mtr	854	1337	1488	1502
Mtrf1	284	210	278	256
Mtrf1l	374	306	424	303
Mtrr	695	502	536	486
Mtss1	1102	801	1023	1158
Mtss1l	13	15	19	4
Mttp	9	48	32	6
Mturn	491	239	445	166
Mtus1	1299	2240	2107	2940
Mtx1	923	900	837	769

Mtx2	1559	1237	1658	1593
Mtx3	379	672	429	361
Muc1	192	70	51	168
Muc13	10	0	5	0
Muc15	59	0	13	81
Muc20	48	29	14	10
Mul1	594	580	579	653
Mum1	334	355	245	103
Mum1l1	10	32	5	0
Mup5	2493	215	1222	279
Murc	3349	5133	3575	2172
Mus81	328	354	354	369
Musk	10	7	12	0
Mustn1	18738	25967	22948	10216
Mut	357	268	319	378
Mutyh	247	196	119	177
Mvb12a	1230	1593	1091	1506
Mvb12b	568	670	597	703
Mvd	271	403	169	201
Mvk	396	437	373	302
Mvp	2704	5004	2177	2907
Mx1	521	241	248	1316
Mx2	525	372	372	756
Mxd1	1287	724	998	854
Mxd3	51	66	36	44
Mxd4	1380	2846	2102	2463
Mxi1	1762	1187	1608	2018
Mxra7	1633	2804	2774	2057
Mxra8	4324	5089	6277	7900
Myadm	2714	6902	5594	9730
Myadml2	1075	1379	1119	1948
Mybbp1a	2441	3751	3091	2336
Mybl1	67	64	56	39
Mybl2	209	568	310	221
Mybpc1	35433	30133	23393	34289
Mybpc2	23463	9675	2142	17464
Mybph	2488	19378	3894	1965
Myc	2124	1589	1808	936
Mycbp	1387	1059	1313	916
Mycbpap	26	41	50	44
Mycl	490	347	317	539
Mycn	45	62	99	51
Myct1	311	549	712	732
Myd88	1857	1734	1923	2173
Mydgf	2748	3939	2613	3262
Myef2	243	410	414	491
Myf5	35	388	105	29

Myf6	725	2280	1401	725
Myg1	814	936	549	710
Myh10	889	971	1185	2266
Myh11	1298	1501	1697	832
Myh13	0	8	8	0
Myh14	780	511	773	388
Myh15	0	133	27	3
Myh3	2683	28106	10744	4468
Myh6	1355	2496	1805	2261
Myh7b	14	57	10	17
Myh9l1	371	345	488	93
Myl12a	30729	28858	28194	23951
Myl12b	5751	3648	5088	5819
Myl2	1580	2171	1542	2048
Myl3	1188	940	909	1901
Myl4	912	18902	3444	1799
Myl6	30339	30452	33289	39180
Myl9	1166	3253	2052	3660
Mylip	873	856	1064	778
Mylk	1045	640	1195	976
Mylk2	4400	2173	730	3677
Mylk3	54	29	30	17
Mylk4	34	9	3	85
Mylpf	174216	173494	61022	125249
Mynn	474	378	402	400
Myo10	1993	1713	2266	1744
Myo15a	41	105	90	211
Myo16	11	63	2	0
Myo18b	2011	2281	1163	1388
Myo19	514	628	504	432
Myo1a	9	1	5	7
Myo1b	0	13	0	2
Myo1c	5451	8374	6930	8381
Myo1d	1005	1112	1160	1405
Myo1e	440	927	925	1097
Myo1f	967	1184	1390	3367
Myo1g	280	447	310	611
Myo5a	511	619	664	1152
Myo5b	459	175	280	128
Myo5c	19	14	23	30
Myo6	883	646	817	561
Myo7a	157	164	259	399
Myo7b	159	42	87	166
Myo9a	73	106	84	67
Myo9b	1749	2251	1672	2307
Myoc	943	356	415	421
Myod1	601	541	605	459

Myof	1172	2438	1799	3979
Myog	760	4857	2343	662
Myom1	6754	4709	3673	5059
Myom2	5933	8055	2672	6818
Myom3	1968	4973	2398	1976
Myoz1	5877	3719	742	4414
Myoz2	4123	5273	3337	3089
Myoz3	741	230	162	743
Mypn	2162	2175	1479	1604
Mypop	103	186	132	81
Myrf	97	100	150	106
Myrip	69	69	24	58
Mysm1	487	339	442	357
Myzap	426	630	555	347
Mzb1	6	9	8	0
Mzf1	83	29	96	35
Mzt1	262	204	196	122
Mzt2b	1270	864	1360	986
N4bp1	1153	1142	1113	1287
N4bp2	648	502	682	774
N4bp2l1	281	433	302	407
N4bp2l2	1777	1281	1405	1340
N6amt1	465	563	378	389
Naa10	1166	1398	1053	839
Naa15	1834	1207	1199	1683
Naa16	206	196	200	272
Naa20	2750	2721	2459	3340
Naa25	970	962	856	715
Naa30	1178	601	680	865
Naa35	2118	1252	1690	1476
Naa38	3653	3006	2860	2838
Naa40	365	216	364	306
Naa50	113	109	120	151
Naa60	1575	1615	1578	1629
Naaa	888	920	1404	2014
Naalad2	9	7	63	25
Naaladl2	27	67	27	62
Nab1	1176	748	936	1172
Nab2	551	893	760	702
Nabp1	617	354	587	547
Nabp2	2472	3038	2653	2650
Naca	27178	25719	27958	24084
Nacad	14	15	6	4
Nacc1	1517	2216	1601	1447
Nacc2	387	230	442	369
Nadk	2574	2542	2325	2963
Nadk2	253	53	229	293

Nadsyn1	240	274	235	181
Nae1	583	444	396	390
Naf1	220	226	317	300
Naga	1831	1821	1556	1894
Nagk	1569	1976	1527	2412
Naglu	1047	1434	1249	2214
Nagpa	64	138	80	128
Nags	21	40	15	6
Naif1	123	145	184	207
Naip5	58	16	116	185
Naip6	162	55	224	382
Nampt	978	944	1121	1657
Nanos1	209	157	235	219
Nanos3	0	15	2	27
Nanp	349	191	292	347
Nans	1978	2784	2186	2934
Nap1l1	4763	4572	4544	4608
Nap1l2	13	17	15	0
Nap1l3	23	11	30	26
Nap1l4	2575	3225	1915	2741
Nap1l5	12	0	14	0
Napa	3022	2988	2931	3007
Napb	14	11	39	11
Napepld	56	112	111	47
Napg	629	586	767	729
Naprt	876	529	453	346
Napsa	480	598	450	1159
Narf	1590	1057	1168	1432
Narfl	463	614	462	540
Nars	2683	3862	3567	3211
Nars2	225	319	272	282
Nasp	2745	3117	2747	2809
Nat1	546	462	447	745
Nat10	365	748	618	291
Nat14	58	100	69	17
Nat8	68	14	78	12
Nat8f3	51	91	95	93
Nat8l	48	144	84	43
Nat9	1384	1310	1107	1087
Nav1	2300	3808	3205	3878
Nav2	874	578	1056	374
Nav3	19	96	51	67
Naxd	687	595	504	356
Naxe	2158	1623	1810	1324
Nbas	370	604	534	561
Nbea	75	206	202	174
Nbeal1	138	115	128	82

Nbeal2	194	265	156	132
Nbl1	4492	7320	5257	4518
Nbn	1047	1143	1016	1769
Nbr1	3252	3387	3673	3767
Ncald	263	583	510	1406
Ncam1	1344	5227	2950	3896
Ncam2	11	9	0	16
Ncapd2	637	1034	719	615
Ncapd3	189	315	203	117
Ncapg	50	65	60	71
Ncapg2	270	393	484	407
Ncaph	537	770	644	439
Ncaph2	1587	2026	1654	1579
Ncbp1	2186	1587	1956	1732
Ncbp3	458	679	677	552
Nccrp1	343	75	38	0
Ncdn	514	535	409	339
Nceh1	857	753	773	902
Ncf1	1321	1859	2397	3798
Ncf2	1401	1379	1549	1941
Ncf4	918	1416	964	1926
Nck1	689	1044	883	1308
Nck2	780	386	797	396
Nckap1	5751	3393	4666	3607
Nckap1l	956	1398	1086	2602
Nckap5	14	31	47	0
Nckap5l	341	374	364	398
Nckipsd	305	401	208	287
Ncl	1892	2210	4154	2606
Ncln	1041	1652	835	1196
Ncoa1	1093	881	898	903
Ncoa2	20	56	113	34
Ncoa3	1013	1022	1199	1147
Ncoa4	2392	2268	2243	4171
Ncoa5	698	688	683	698
Ncoa6	831	796	978	1076
Ncoa7	923	720	709	808
Ncor1	1126	954	1437	769
Ncor2	992	1329	895	681
Ncr1	15	38	24	21
Ncr3	99	30	91	0
Ncs1	931	870	730	521
Ncstn	2709	3769	3304	3491
ND2	295726	172466	132954	204235
ND3	67550	25420	19621	37149
ND5	112903	71301	46318	101410
ND6	129010	68237	69157	89589

Ndc1	698	707	751	820
Ndc80	406	581	621	609
Nde1	786	705	736	824
Ndel1	1650	1745	1959	2389
Ndfip1	5667	5308	5591	8742
Ndfip2	1138	759	958	1656
Ndn	351	454	498	538
Ndnf	76	94	122	337
Ndnl2	626	575	576	568
Ndor1	623	462	430	358
Ndrg1	12271	6952	10248	10932
Ndrg2	12421	8821	10517	11979
Ndrg3	1025	814	1058	833
Ndrg4	298	901	630	691
Ndst1	1106	1334	1357	1318
Ndst3	2	0	8	20
Ndufa10	1875	1368	1295	1393
Ndufa10l1	1161	1135	1458	1663
Ndufa11	2672	3022	1891	2092
Ndufa12	703	695	572	320
Ndufa2	4965	4704	4210	4114
Ndufa3	2192	1474	2604	2246
Ndufa4	18062	11302	11817	11626
Ndufa4l2	1502	1370	1099	1565
Ndufa5	7811	5597	6579	6542
Ndufa6	9076	6570	7234	5834
Ndufa7	2647	2738	1962	1503
Ndufa8	4071	3607	2771	3520
Ndufa9	4253	4258	3597	4433
Ndufab1	6215	4202	3552	4271
Ndufaf1	277	280	319	312
Ndufaf2	137	218	203	142
Ndufaf3	987	1136	684	693
Ndufaf4	764	473	644	610
Ndufaf5	946	948	784	915
Ndufaf6	178	186	126	117
Ndufaf7	562	625	608	834
Ndufb1	952	672	643	579
Ndufb10	6840	5667	5884	5337
Ndufb11	7580	5843	6074	5770
Ndufb2	8404	5909	5859	5707
Ndufb3	4511	2661	3289	2768
Ndufb4	743	599	815	616
Ndufb5	8225	5810	5642	6002
Ndufb6	3041	2122	1992	2520
Ndufb7	1818	2527	1458	1169
Ndufb8	11506	10538	8239	9661

Ndufb9	9718	8731	7425	8999
Ndufc1	2980	1703	1204	1852
Ndufc2	4146	1765	1502	3125
Ndufs1	4235	3895	3337	4051
Ndufs2	6923	7740	5478	6471
Ndufs3	5743	4819	3698	4357
Ndufs4	6196	4199	4866	4454
Ndufs5	1180	1089	979	1371
Ndufs6	1375	1007	1562	1771
Ndufs7	1280	1302	927	700
Ndufs8	1968	2156	1419	1750
Ndufv1	3045	3679	2242	2864
Ndufv2	9959	8003	8042	8586
Ndufv3	1141	1331	1146	1268
Neb	18862	18601	18979	17529
Nebl	37	0	23	12
Necab2	14	0	9	3
Necap1	526	564	632	718
Necap2	1905	2462	2156	2641
Nectin1	3034	1926	2985	789
Nectin2	1063	1266	1383	1330
Nectin3	302	407	603	482
Nectin4	1898	708	2374	796
Nedd1	156	203	238	164
Nedd4	12898	16408	18034	15553
Nedd4l	300	352	302	330
Nedd8	6717	7119	6843	7501
Nedd9	1645	928	1267	855
Nefh	140	725	307	541
Neil1	425	230	437	291
Neil2	12	26	36	23
Nek1	206	102	129	198
Nek10	45	24	9	45
Nek11	77	47	63	41
Nek2	39	96	56	38
Nek2l1	2	60	3	0
Nek3	118	86	101	44
Nek4	315	262	259	132
Nek6	870	1171	1211	2292
Nek7	2284	2673	2285	3376
Nek8	186	130	144	182
Nek9	2142	1840	2305	2548
Nelfa	726	1170	1076	1067
Nelfb	1166	1705	1109	1357
Nelfcd	1373	1497	1056	828
Nelfe	1827	2110	2052	1553
Nell2	0	27	23	12

Nemf	304	283	330	185
Nemp1	46	25	14	53
Nemp2	152	146	191	169
Nenf	2099	2480	2313	2140
Neo1	1371	1167	1225	1086
Nepn	30	9	12	53
Nepro	477	376	400	395
Nes	674	4779	1778	1698
Net1	8076	3783	5335	3874
Neu1	873	1267	909	1923
Neu2	654	98	179	147
Neu3	273	123	193	142
Neurl1	455	929	466	438
Neurl2	56	91	27	43
Neurl3	282	204	214	162
Neurl4	311	410	355	345
NEWGENE_	0	14	2	0
NEWGENE_	198	254	414	144
NEWGENE_	0	27	3	0
NEWGENE_	78	17	23	23
Nexn	4306	4869	4324	2927
Nf1	77	272	114	123
Nf1x	3344	2974	3121	2893
Nf2	2027	2259	2109	1958
Nfam1	937	1426	1297	2406
Nfasc	171	156	253	56
Nfat5	1890	1147	1896	1780
Nfatc1	768	666	418	976
Nfatc2	427	741	313	421
Nfatc2ip	243	214	253	184
Nfatc3	468	740	465	257
Nfatc4	391	949	539	1097
Nfe2	37	68	33	32
Nfe2l1	6455	6372	5814	5294
Nfe2l2	2181	1980	2675	2720
Nfe2l3	177	27	123	44
Nfia	1275	719	912	1079
Nfib	214	183	322	14
Nfic	2634	3945	2350	2963
Nfil3	1583	786	1548	1034
Nfkb2	1269	1362	996	1377
Nfkbia	5037	3289	4867	3016
Nfkbib	645	615	584	504
Nfkbid	200	155	179	140
Nfkbie	1057	980	1229	1545
Nfkbil1	130	249	185	170
Nfkbiz	418	277	484	298

Nfrkb	607	544	674	592
Nfu1	975	662	871	1473
Nfx1	1913	1661	1994	1934
Nfxl1	195	281	209	223
Nfya	753	812	722	707
Nfyb	555	705	659	606
Nfyc	2029	1975	1756	1671
Ngb	19	0	9	18
Ngdn	1054	1084	1029	733
Ngef	116	321	66	67
Ngf	13	196	99	77
Ngfr	116	140	215	69
Ngly1	888	1052	1100	1279
Ngrn	1487	1194	1544	1201
Nhej1	121	53	47	142
Nhlrc1	9	26	6	5
Nhlrc2	708	555	695	707
Nhlrc3	1059	1361	1372	2188
Nhp2l1	4381	3509	3709	3286
Nhs	120	70	98	3
Nhsl1	95	118	173	78
Nhsl2	57	210	144	57
Niacr1	3284	4632	2680	709
Nicn1	595	510	501	659
Nid1	3261	8265	7043	9494
Nid2	1087	2904	2267	3274
Nif3l1	510	557	385	593
Nifk	2859	1891	2103	2758
Nim1k	11	24	59	6
Nin	670	454	546	889
Ninj1	2478	4369	2863	3437
Ninj2	53	96	30	15
Ninl	68	118	95	6
Nip7	791	837	754	1031
Nipa1	32	48	48	152
Nipa2	1665	1417	1556	1929
Nipal1	205	58	72	27
Nipal2	1228	910	990	616
Nipal3	9	6	12	0
Nipal4	659	309	617	79
Nipbl	1220	1017	1524	955
Nipsnap1	681	465	814	719
Nipsnap3a	1546	1197	1441	1658
Nisch	2788	4038	2923	2970
Nit1	3358	2669	2416	3081
Nit2	2940	1306	1771	1866
Nkain1	672	1588	722	1668

Nkain4	7	9	18	10
Nkap	126	114	114	185
Nkd1	207	128	253	279
Nkd2	554	529	671	484
Nkg7	363	86	307	107
Nkiras1	344	321	323	363
Nkiras2	772	1119	889	731
Nkpd1	194	45	140	25
Nkrf	0	6	24	32
Nktr	1102	991	1387	1212
Nle1	290	589	272	277
Nlgn1	6	0	29	0
Nlgn2	68	183	144	137
Nlgn3	46	40	20	69
Nlk	251	138	161	177
Nln	1371	1231	1505	1364
Nlrc4	112	80	113	166
Nlrc5	874	632	638	644
Nlrp10	1815	327	1670	270
Nlrp1a	213	251	339	445
Nlrp3	217	259	173	255
Nlrx1	599	686	521	575
Nmd3	1218	1081	1100	1158
Nme1	3453	3129	4166	3591
Nme2	17904	19860	22833	19450
Nme3	403	307	363	332
Nme4	237	385	260	162
Nme5	27	0	6	0
Nme6	463	469	325	347
Nme7	1517	993	512	1400
Nmi	507	515	465	486
Nmnat1	418	473	319	402
Nmnat3	382	336	236	486
Nmral1	484	510	355	190
Nmrk1	64	84	83	30
Nmrk2	201	287	59	165
Nmt1	3575	4156	3476	4181
Nmt2	127	275	170	495
Nnat	201	427	161	172
Nnmt	312	554	403	680
Nnt	1718	1716	1491	2824
Noa1	448	497	427	419
Nob1	1585	1698	1398	1135
Noc2l	1854	2030	1670	1210
Noc3l	29	90	29	22
Noc4l	645	548	530	436
Noct	1051	340	527	335

Nod1	1093	1257	1013	953
Nod2	242	166	152	110
Nog	45	16	62	17
Nol10	507	826	654	488
Nol11	1590	1287	1411	1198
Nol12	634	865	642	441
Nol3	3857	4366	3502	3409
Nol4l	223	241	305	534
Nol6	1202	1244	1109	1086
Nol7	3325	2427	3025	2669
Nol9	951	1037	870	986
Nolc1	1930	2668	2594	2145
Nom1	432	381	481	586
Nomo1	1634	2142	1154	1361
Nono	6700	5128	6807	7210
Nop10	3974	3100	3238	3003
Nop14	883	1077	1315	891
Nop16	2456	2770	2916	2616
Nop2	746	954	536	352
Nop56	738	1070	866	821
Nop58	2044	2810	2136	2418
Nop9	882	909	791	900
Nos1	7	15	9	61
Nos2	64	160	30	0
Nos3	108	846	363	431
Nosip	418	727	330	393
Nostrin	96	230	224	208
Notch1	1076	891	1461	646
Notch2	1804	1961	2282	2381
Notch3	2375	2817	2952	1388
Notch4	585	796	918	819
Notum	15	4	81	10
Nov	1584	2683	1793	2088
Nova1	35	51	15	72
Nova2	5	36	12	25
Nox4	114	129	125	138
Noxa1	8	14	5	0
Npap60	1703	1726	1780	1858
Npas2	129	291	167	121
Npat	227	168	310	324
Npc1	902	802	1102	1432
Npc2	10853	12790	14704	29846
Npdc1	2803	3261	3000	2302
Npepl1	2013	2013	1587	1829
Npepo	2675	2181	2245	1613
Npepps	1628	2139	2167	1897
Npff	210	170	135	47

Nphp1	424	395	633	375
Nphp3	308	158	158	221
Nphp4	52	129	80	58
Npl	433	436	558	673
Nploc4	982	1304	1154	1156
Npm1	10577	4950	6400	7094
Npm3	1359	1438	1058	643
Npnt	677	908	531	483
Nppa	7	0	18	0
Nppb	915	1065	1059	546
Nppc	41	0	21	2
Npr1	414	938	620	662
Npr2	659	770	724	733
Npr3	269	377	184	523
Nprl2	676	569	531	584
Nprl3	263	390	289	204
Nptn	2382	2809	2306	3672
Nptx1	8	26	26	27
Nptxr	116	363	242	364
Npw	53	67	23	8
Npy1r	84	154	173	166
Nqo1	172	479	264	613
Nqo2	584	498	703	854
Nr1d1	929	1989	1150	1379
Nr1d2	1281	1271	1091	1276
Nr1h2	864	1326	657	826
Nr1h3	555	474	504	632
Nr1i3	16	0	8	0
Nr2c1	792	647	728	736
Nr2c2	39	54	41	40
Nr2c2ap	1516	1281	1258	969
Nr2f1	23	54	24	45
Nr2f2	343	443	654	562
Nr2f6	808	721	706	562
Nr3c1	1165	1529	1446	1424
Nr3c2	114	97	107	59
Nr4a1	1427	1015	958	779
Nr4a2	134	131	164	100
Nr4a3	502	137	287	242
Nr5a1	16	25	8	15
Nr5a2	36	93	80	73
Nr6a1	113	93	105	0
Nradd	900	591	909	480
Nrap	9808	8910	4162	7293
Nrarp	1751	993	1798	703
Nras	3390	2977	4169	3756
Nrbf2	53	100	69	26

Nrbp1	1734	2669	2139	2443
Nrbp2	3349	1187	2663	1019
Nrcam	144	222	214	715
Nrdc	7289	5724	6591	6319
Nrde2	82	187	93	189
Nrep	4077	9651	5097	9503
Nrf1	293	520	394	423
Nrg1	366	874	402	308
Nrg2	24	24	12	0
Nrg4	902	228	252	281
Nrgn	37	45	27	19
Nrip1	861	394	528	553
Nrip2	34	26	71	31
Nrip3	218	253	278	192
Nrm	546	682	549	469
Nrn1	52	144	126	201
Nrp1	533	1139	1097	1611
Nrp2	913	977	1238	2206
Nrros	791	1022	1005	1612
Nrsn1	114	40	27	21
Nrxn2	38	22	45	15
Nrxn3	0	14	11	0
Ns5atp4	1	7	18	3
Ns5atp9	375	522	444	275
Nsa2	3241	2654	3277	3300
Nsd1	1930	1902	2143	1594
Nsdhl	3055	1256	1971	1714
Nsf	944	1069	1136	1251
Nsfl1c	3524	4399	3662	2826
Nsg1	44	176	22	161
Nsg2	217	220	298	205
Nsl1	99	274	143	94
Nsmaf	756	732	927	1218
Nsmce1	1161	1230	1047	1046
Nsmce2	962	1140	999	824
Nsmce4a	1946	2128	2183	1969
Nsmf	580	671	636	591
Nsrp1	679	852	912	834
Nsun2	3260	3365	3212	3106
Nsun5	772	902	978	816
Nsun6	182	229	203	131
Nsun7	56	51	40	64
Nt5c	730	654	712	558
Nt5c1a	69	48	44	41
Nt5c2	1374	1789	1479	1702
Nt5c3a	962	821	958	1074
Nt5c3b	458	662	661	668

Nt5dc1	394	299	314	438
Nt5dc2	425	836	323	826
Nt5dc3	574	677	475	984
Nt5e	1225	1473	1396	1308
Nt5m	95	119	62	127
Ntan1	121	115	221	192
Ntf3	30	64	42	24
Ntf4	264	156	196	131
Nthl1	264	339	230	278
Ntm	91	273	241	214
Ntmt1	978	1213	661	962
Ntn4	47	141	114	32
Ntng1	7	5	30	37
Ntng2	23	134	38	57
Ntpcr	795	590	548	628
Ntrk2	1447	1027	1177	640
Ntrk3	268	102	159	89
Ntsr1	9	70	39	34
Ntsr2	14	12	2	38
Nuak1	239	208	242	169
Nuak2	1306	630	1232	687
Nub1	1285	1177	1517	1553
Nubp1	1284	1385	898	1006
Nubp2	1921	2060	2158	1658
Nubpl	382	180	307	92
Nucb1	4852	11068	6710	8318
Nucb2	3226	4297	4136	6304
Nucks1	4760	3219	4022	3922
Nudc	29	138	77	81
Nudcd1	206	410	295	281
Nudcd2	535	518	400	554
Nudcd3	0	26	5	0
Nudt1	425	296	272	295
Nudt11	44	0	45	4
Nudt12	136	81	98	94
Nudt13	187	219	161	162
Nudt14	849	651	509	351
Nudt15	190	122	181	228
Nudt16	745	811	806	610
Nudt16l1	1733	1434	1536	1047
Nudt17	24	15	14	23
Nudt18	1118	1442	1241	2070
Nudt19	649	662	576	494
Nudt2	1271	1476	1020	1237
Nudt21	2277	1937	2255	2479
Nudt22	392	370	379	364
Nudt3	1702	1783	1229	1650

Nudt4	5898	4979	5692	4967
Nudt5	2743	1710	1687	1268
Nudt6	339	167	211	172
Nudt7	409	415	433	422
Nudt8	567	355	369	265
Nudt9	1311	2077	1580	2011
Nuf2	574	806	698	518
Nufip1	771	825	939	1021
Nufip2	1255	1167	1149	1289
Numa1	1195	1448	1443	1564
Numb	821	678	839	854
Numbl	488	1279	645	964
Nup107	397	708	606	648
Nup133	627	911	706	746
Nup153	482	546	572	461
Nup155	609	557	543	601
Nup160	134	235	257	146
Nup188	249	501	215	241
Nup205	703	817	940	780
Nup210	312	291	251	288
Nup210l	83	40	90	82
Nup214	833	1134	694	930
Nup35	740	740	855	654
Nup37	676	775	688	590
Nup43	669	516	567	564
Nup54	642	836	700	557
Nup58	559	437	500	413
Nup85	549	976	700	843
Nup88	2124	2189	2242	1806
Nup93	1202	1561	1465	1366
Nup98	1301	1847	1548	1467
Nupl2	381	311	349	370
Nupr2	141	42	155	60
Nus1	3171	1955	2478	3477
Nusap1	622	681	656	610
Nutf2	3774	3165	3290	3213
Nvl	262	269	322	322
Nwd1	10	0	12	27
Nxf1	6611	4944	6194	5375
Nxn	3450	3245	3206	3143
Nxnl2	2	256	132	25
Nxpe1	91	172	190	262
Nxpe2	9	0	6	6
Nxpe3	119	176	206	193
Nxpe4	46	37	42	156
Nxph3	124	264	143	70
Nxph4	31	2	18	4

Nxt1	1063	1188	919	912
Nxt2	15	23	20	42
Nyap1	46	22	15	20
Nynrin	408	520	498	473
Oacyl	24	32	18	1
Oaf	1012	1938	1080	1979
Oard1	622	657	696	969
Oas1a	105	127	63	142
Oas1b	285	261	173	297
Oas2	54	65	45	61
Oasl	290	253	161	322
Oasl2	476	558	325	680
Oat	4508	3864	4803	4329
Oaz1	13972	16081	12718	13024
Oaz2	6164	4887	5185	6078
Obfc1	632	678	549	791
Obp2a	183	17	65	165
Obp3	172	0	33	29
Obscn	3379	3822	1831	2130
Oc90	31	18	17	3
Ocel1	781	675	719	675
Ociad1	5109	4652	4542	5158
Ociad2	784	633	916	756
Ocln	661	289	782	348
Ocm2	3694	1773	1872	1248
Ocrl	361	588	477	847
Odc1	2328	4730	2639	3006
Odf2	392	1047	665	635
Odf2l	117	97	122	62
Odf3l1	56	86	23	40
Ofd1	693	326	633	389
Ogdhl	418	154	229	291
Ogfod1	11	26	17	76
Ogfod2	45	47	5	7
Ogfod3	308	381	310	266
Ogfr	1433	1734	1297	1423
Ogfrl1	2122	2085	2139	2700
Ogg1	138	258	159	85
Ogt	2193	1294	1778	1203
Oip5	54	4	68	39
Ola1	2879	1858	1890	2219
Olah	13	41	26	141
Olfm1	495	577	504	718
Olfm2	76	13	56	43
Olfml1	1541	717	1879	1177
Olfml2b	347	3028	1188	2172
Olfml3	2921	4290	3427	7680

Olig1	0	12	17	0
Olr1	109	204	185	357
Olr1584	0	15	6	35
Olr1637	12	0	11	22
Olr63	100	214	298	69
Oma1	630	530	733	740
Opa1	2085	1669	2180	2380
Opa3	884	1159	889	945
Opcml	24	50	9	18
Ophn1	816	323	800	519
Oplah	394	433	386	351
Opn3	490	288	251	341
Optc	46	122	135	21
Optn	2415	2334	2969	3520
Orai1	534	846	376	336
Orai2	525	428	512	601
Orai3	836	660	891	972
Oraov1	321	344	384	367
Orc1	56	177	87	41
Orc2	762	567	826	852
Orc3	516	255	466	380
Orc4	341	274	365	679
Orc5	754	519	593	511
Orc6	328	318	247	252
Orm1	21	8	8	0
Ormdl1	84	51	60	60
Ormdl2	2365	1654	2067	1959
Ormdl3	1753	1712	1915	1527
Os9	4521	6969	6056	7691
Osbp	2586	2261	2693	2873
Osbp2	71	145	45	76
Osbpl10	326	333	213	379
Osbpl11	431	537	460	499
Osbpl1a	1142	1242	1211	1245
Osbpl2	1383	1338	1229	2030
Osbpl3	1436	798	1028	882
Osbpl5	2811	1566	2001	1313
Osbpl6	331	342	289	354
Osbpl7	199	193	182	73
Osbpl8	80	99	119	263
Osbpl9	26	23	45	11
Oscar	168	144	147	417
Oscp1	192	266	220	206
Oser1	1615	1330	1392	2108
Osgep	824	915	1047	786
Osgepl1	226	214	168	244
Osgin1	141	104	77	77

Osgin2	383	339	336	496
Osm	166	296	129	187
Osmr	2273	1716	2165	2234
Osr1	229	372	188	42
Osr2	97	137	153	295
Ost4	7558	6199	6391	8079
Ostc	2609	3977	3643	4873
Ostf1	1808	2184	2198	3319
Ostm1	151	152	89	322
Otop3	12	0	17	4
Otor	15	2	8	0
Otub1	1906	2269	1765	1692
Otub2	219	163	156	118
Otud1	335	340	471	377
Otud3	36	64	63	22
Otud4	633	530	808	792
Otud5	2486	2445	2275	2482
Otud6b	217	533	364	294
Otud7a	78	63	53	23
Otud7b	10	30	5	10
Otulin	2191	2260	2582	2622
Ovol1	1582	473	839	204
Ovol2	164	106	95	44
Oxa1l	1963	2486	1595	1937
Oxct1	3877	2943	3391	4222
Oxld1	226	224	182	195
Oxnad1	617	713	575	513
Oxr1	983	837	937	1429
Oxsm	487	355	390	420
Oxsr1	2134	2405	2558	2330
Oxtr	79	28	71	0
P2rx1	36	60	84	123
P2rx2	14	0	26	0
P2rx3	30	49	41	39
P2rx4	1845	2884	2278	5424
P2rx5	151	180	238	182
P2rx6	148	78	38	152
P2ry1	428	205	236	270
P2ry10	110	91	59	153
P2ry12	80	51	89	131
P2ry13	585	627	513	350
P2ry14	270	124	152	205
P2ry2	50	116	175	240
P2ry4	75	53	111	106
P2ry6	504	769	627	1525
P3h1	1064	2925	1453	2865
P3h4	933	2204	1223	2641

P4ha1	1841	3105	2744	4010
P4ha2	847	2385	1286	2945
P4ha3	556	1425	969	2325
P4hb	14411	24348	19764	28533
P4htm	222	269	147	144
Pa2g4	5728	6155	6197	4734
Pabpc1	14124	19743	19814	22502
Pabpc4	2659	4055	1980	2723
Pabpn1	4367	4382	4455	3484
Pacrg	24	10	15	0
Pacrgl	36	61	104	44
Pacs1	772	734	686	650
Pacs2	1167	1738	1261	1931
Pacsin2	1576	2436	1648	2866
Pacsin3	4781	6684	4338	3582
Padi1	15	0	41	0
Padi2	1607	859	1369	1278
Padi3	247	108	433	49
Padi4	128	40	51	20
Paf1	2584	3208	2962	3369
Pafah1b1	7199	5133	6576	6347
Pafah1b2	2515	2397	2555	2564
Pafah1b3	246	252	272	233
Pafah2	363	392	352	312
Pagr1	565	817	439	784
Pah	11	26	12	33
Paics	8	68	36	67
Paip1	1870	1335	2204	2106
Paip2	4679	3522	4460	4301
Paip2b	1286	1371	1160	979
Pak1	1462	1206	1292	1188
Pak1ip1	798	962	867	894
Pak3	431	26	250	81
Pak4	531	529	533	378
Pak6	94	159	149	67
Palb2	93	95	125	52
Pald1	624	769	635	636
Palld	41	195	9	48
Palm2	0	17	8	10
Palm3	13	2	17	3
Palmd	4373	2403	5529	1433
Pam	3495	4878	5267	6603
Pam16	1444	1431	1145	849
Pamr1	294	365	107	156
Pan2	746	806	700	450
Pan3	1956	1008	1860	1595
Pank1	2679	981	1300	1717

Pank2	921	778	775	771
Pank3	813	750	964	1057
Pank4	712	341	441	287
Panx1	803	911	645	368
Paox	1179	1037	1047	913
Papd4	908	865	715	819
Papd5	612	452	384	534
Papd7	870	817	728	754
Papln	202	194	157	126
Papola	3215	2392	2695	3594
Papolg	194	134	137	156
Pappa	87	42	66	25
Pappa2	105	165	90	54
Papss1	1364	1019	1137	1393
Papss2	761	906	745	1631
Paqr3	751	249	619	524
Paqr4	450	299	327	436
Paqr5	334	68	169	137
Paqr6	196	163	203	164
Paqr7	449	401	456	633
Paqr8	0	271	251	0
Pard3	1036	829	985	761
Pard3b	65	62	44	34
Pard6a	55	38	6	31
Pard6b	49	48	59	40
Pard6g	6	46	14	3
Parg	558	521	728	1053
Park2	19	0	33	1
Park7	4615	5539	3888	4315
Parl	1783	2127	1813	1754
Parm1	273	258	299	164
Parn	787	680	718	734
Parp1	1225	2032	1395	1693
Parp10	6435	11304	6030	6233
Parp11	166	169	221	153
Parp12	47	161	84	151
Parp14	49	186	178	47
Parp16	345	449	326	307
Parp2	880	864	796	871
Parp3	765	1293	570	858
Parp4	918	836	1103	1322
Parp6	1271	1585	1488	1876
Parp8	1184	762	859	793
Parp9	1228	1285	1586	1295
Parpbp	9	21	27	31
Pars2	282	241	190	330
Parva	3461	4016	4279	3631

Parvb	1516	3318	2144	3777
Parvg	168	348	346	926
Pask	231	215	199	158
Patj	751	414	617	353
Patl1	923	828	931	908
Patz1	379	393	548	388
Pawr	1586	595	1375	917
Pax1	51	16	18	16
Pax3	33	52	17	7
Pax7	154	659	114	122
Pax8	187	167	226	441
Paxbp1	731	540	615	590
Paxip1	223	422	433	371
Pbdc1	2009	1595	2541	2307
Pbk	240	317	233	170
Pbld1	64	51	48	60
Pbrm1	1328	1387	1798	1296
Pbx1	328	178	200	236
Pbx2	1047	1196	1324	1246
Pbx3	621	627	548	558
Pbx4	3	3	2	0
Pbxip1	4070	5808	4992	4689
Pc	876	821	585	746
Pcbd1	162	290	117	78
Pcbd2	450	313	304	232
Pcbp1	4366	5828	4706	5509
Pcbp2	6036	5403	6872	7545
Pcbp3	165	364	215	245
Pcbp4	478	731	543	524
Pcca	1016	652	758	620
Pccb	2018	1654	1423	1704
Pcdh1	1588	795	2007	911
Pcdh12	136	580	265	156
Pcdh17	14	12	45	49
Pcdh18	174	211	123	327
Pcdh19	229	446	454	292
Pcdh20	43	29	3	0
Pcdh7	696	650	557	531
Pcdh9	38	21	24	0
Pcdha4	84	111	205	182
Pcdhb10	10	36	6	0
Pcdhb11	44	50	18	33
Pcdhb12	69	31	42	19
Pcdhb14	0	41	39	19
Pcdhb17	87	48	95	130
Pcdhb19	146	103	77	117
Pcdhb20	9	25	26	49

Pcdhb21	0	15	6	0
Pcdhb22	14	46	24	21
Pcdhb3	20	0	24	36
Pcdhb5	8	25	5	39
Pcdhb6	5	4	8	14
Pcdhb7	9	20	17	0
Pcdhb9	5	21	30	0
Pcdhga1	1859	3975	2896	2654
Pcdhgb5	52	44	59	40
Pced1a	362	468	330	412
Pcf11	528	481	506	283
Pcgf2	575	468	691	431
Pcgf3	128	242	172	229
Pcgf5	217	240	346	451
Pcgf6	269	186	170	195
Pcid2	592	485	459	520
Pcif1	455	623	427	649
Pck1	174	4	135	4
Pck2	443	940	737	875
Pcm1	1405	960	1569	1467
Pcmt1	2421	1842	1994	2819
Pcmtd1	1963	1350	1583	1719
Pcmtd2	200	232	247	104
Pcna	3326	3299	3652	3678
Pcnp	4891	3605	4464	4251
Pcnt	654	946	509	627
Pcnx1	1093	1162	1220	1252
Pcnx3	578	781	421	460
Pcnx4	85	80	140	125
Pcolce	7182	18025	11596	21780
PCOLCE2	208	567	221	364
Pcp4	172	37	143	108
Pcp4l1	74	109	117	36
Pcsk4	21	20	23	10
Pcsk5	643	1250	1208	799
Pcsk6	364	629	247	204
Pcsk7	345	425	475	392
Pcsk9	12	42	14	160
Pctp	3355	1581	2109	2260
Pcyox1	3044	2934	3393	4156
Pcyox1l	136	162	120	103
Pcyt1a	2116	2625	2093	3237
Pcyt1b	61	103	199	67
Pcyt2	809	869	621	482
Pdap1	6169	5568	4942	5596
Pdcd10	1404	1017	1356	1393
Pdcd11	932	1182	831	938

Pdcd1lg2	15	15	14	15
Pdcd2	1210	1045	1131	1123
Pdcd2l	485	514	406	317
Pdcd4	5298	2871	4059	4198
Pdcd6	3276	3290	3348	4002
Pdcd6ip	2286	3433	2908	3278
Pdcd7	726	758	913	707
Pdcl3	1637	1967	1702	1829
Pde10a	24	67	53	19
Pde12	92	87	116	106
Pde1a	76	72	71	79
Pde1b	4	29	50	33
Pde1c	51	86	44	24
Pde2a	454	563	799	751
Pde3a	168	262	323	350
Pde3b	14	144	83	77
Pde4a	273	287	208	306
Pde4b	39	104	77	75
Pde4c	24	16	26	0
Pde4d	496	614	689	805
Pde4dip	13220	9948	6519	10461
Pde5a	317	409	402	394
Pde6a	5	0	21	11
Pde6d	726	1051	683	736
Pde6g	3	27	11	6
Pde7a	544	288	627	477
Pde7b	362	404	379	761
Pde8a	320	679	406	631
Pde8b	94	61	51	86
Pde9a	107	247	141	71
Pdgfa	1484	1304	1247	1307
Pdgfb	241	662	248	368
Pdgfc	297	286	438	240
Pdgfd	14	11	6	41
Pdgfra	1851	2080	2355	3099
Pdgfrb	2189	4201	4255	4377
Pdgfrl	2030	1845	1785	2270
Pdha1	3842	3114	3462	2986
Pdhb	4071	3039	3826	4438
Pdhx	898	993	933	993
Pdia3	19282	20831	26444	30762
Pdia4	2178	4718	4365	4072
Pdia5	598	1069	1265	1402
Pdia6	5215	8471	6111	8023
Pdik1l	222	198	116	178
Pdk1	1435	1338	1372	1096
Pdk2	2828	2402	1924	2264

Pdk3	2344	1421	2123	2086
Pdk4	3171	2706	1508	1920
Pdlim1	4483	5367	5483	3875
Pdlim2	1813	1016	1494	1070
Pdlim3	17209	19213	14549	11045
Pdlim4	1320	4042	2233	2996
Pdlim5	5609	3959	4049	5350
Pdlim7	2989	3811	3054	3210
Pdp1	593	432	454	800
Pdp2	68	12	29	106
Pdpn	4268	7839	6804	9145
Pdpr	102	135	98	148
Pdrg1	2060	1939	2034	1778
Pds5a	1033	1327	1827	1518
Pds5b	537	511	685	695
Pdss1	1712	773	916	924
Pdss2	481	602	424	415
Pdxdc1	82	155	170	237
Pdxk	57	55	35	69
Pdxp	160	158	89	131
Pdzd11	1424	1101	1162	1344
Pdzd2	63	41	100	29
Pdzd3	53	35	24	19
Pdzd4	26	73	53	51
Pdzd8	560	589	764	812
Pdzd9	28	43	22	36
Pdzk1	321	174	143	167
Pdzk1ip1	476	233	203	101
Pdzrn3	1534	949	1455	1344
Pdzrn4	113	41	220	216
Pea15	9588	10485	11229	10519
Peak1	932	1204	1389	2076
Pebp1	4501	5647	4268	4612
Pecam1	2517	5799	5156	4053
Pecr	1138	717	674	1269
Pef1	1907	1951	1471	1736
Peg12	47	161	114	234
Peg3	559	904	1339	1688
Peli1	186	205	170	185
Peli2	556	584	686	1047
Peli3	237	206	172	179
Pelo	1779	2073	1718	1657
Pelp1	1173	1037	1279	1093
Pemt	76	89	51	40
Penk	135	232	209	291
Peo1	625	525	521	475
Pepd	2074	2622	2147	2749

Per1	2122	1326	2012	992
Per2	576	416	378	707
Per3	441	616	325	339
Perm1	125	60	65	141
Perp	35852	14070	27299	13052
Pes1	2071	2755	2228	2141
Pet100	2695	2568	2224	2527
Pex1	427	513	525	336
Pex10	200	178	197	143
Pex11a	298	166	178	193
Pex11b	886	736	627	865
Pex11g	132	100	48	93
Pex13	1159	691	865	943
Pex14	249	536	245	262
Pex16	542	410	307	405
Pex19	514	292	274	260
Pex2	880	707	840	1132
Pex26	205	206	334	187
Pex3	908	689	755	1094
Pex6	16	41	33	53
Pex7	164	207	185	132
Pf4	285	1358	818	852
Pfas	545	697	445	439
Pfdn1	5362	5134	4906	5098
Pfdn2	2443	2083	2281	1749
Pfdn4	605	834	763	827
Pfdn5	5110	4752	4878	5077
Pfdn6	1020	1536	1010	1149
Pfkfb1	191	81	48	114
Pfkfb2	649	373	623	546
Pfkfb3	975	977	751	1098
Pfkl	575	1020	685	1129
Pfkm	17457	10592	5311	20421
Pfkp	4480	2933	3719	2136
Pfn1	12872	14660	12452	15304
Pgam1	14832	12325	13358	11893
Pgam2	20861	11616	4684	15300
Pgam5	1583	1322	1699	1200
Pgap1	60	63	98	44
Pgap2	4111	2939	3347	2860
Pgap3	415	306	248	293
Pgbd1	0	27	44	20
Pgbd2	2	0	2	0
Pgbd5	37	31	24	10
Pgd	3889	5222	4822	6537
Pgf	361	465	331	197
Pgghg	827	1243	961	1283

Pggt1b	213	143	265	285
Pgk1	18981	11139	11048	17780
Pgls	487	509	331	335
Pglyrp1	104	37	86	60
Pglyrp2	270	145	50	64
Pglyrp4	1	0	3	0
Pgm1	8521	5780	3102	8661
Pgm2	954	901	773	1455
Pgm2l1	346	460	280	393
Pgm3	362	762	608	908
Pgm5	661	1387	667	718
Pgpep1	69	84	66	165
Pgpep1l	15	68	27	24
Pgrmc1	5042	2427	2562	4423
Pgrmc2	6330	3706	4884	5189
Pgs1	1139	1292	1151	1033
Phactr2	232	285	367	234
Phactr3	2	3	3	23
Phactr4	245	210	265	250
Phax	1014	979	960	1305
Phb	861	927	721	718
Phb2	7269	6730	5719	5490
Phc1	566	639	683	807
Phc2	1459	2366	1827	2041
Phc3	776	795	766	727
Phf1	275	400	284	290
Phf10	2124	2325	2164	2223
Phf11	3	18	15	18
Phf12	416	444	427	538
Phf13	407	358	478	421
Phf14	1334	1155	1401	1559
Phf19	47	155	77	71
Phf2	515	784	567	680
Phf20	425	460	301	260
Phf20l1	715	309	370	451
Phf21a	550	526	925	584
Phf21b	3	19	12	33
Phf23	2199	1739	1915	2322
Phf24	20	43	33	0
Phf3	1087	836	1362	862
Phf5a	1921	2111	1691	2186
Phf7	507	899	588	368
Phf8	376	262	555	466
Phgdh	430	381	295	208
Phip	639	360	528	419
Phka1	2745	1035	1252	2501
Phka2	61	61	15	0

Phkb	1568	1014	877	1552
Phkg1	1851	647	556	1405
Phkg2	1641	1350	1335	1490
Phlda1	1131	777	828	297
Phlda2	62	37	50	5
Phlda3	3585	3945	2645	1775
Phldb1	319	1271	456	687
Phldb2	102	24	32	57
Phldb3	218	228	197	107
Phlpp1	301	328	199	213
Phlpp2	15	12	3	0
Phospho1	3370	2145	1232	2363
Phospho2	650	722	731	893
Phpt1	2739	2672	2603	2710
Phrf1	957	1080	1142	1005
Phtf1	869	611	916	737
Phtf2	1241	790	746	771
Phyh	3113	2330	2225	3663
Phyhipl	32	64	55	65
Pi16	1696	2983	1628	2845
Pi4k2a	850	993	862	1348
Pi4k2b	581	793	692	673
Pi4ka	1046	993	1178	1181
Pi4kb	2118	1983	2243	2174
Pianp	104	81	113	136
Pias1	358	414	441	389
Pias2	951	919	1010	1082
Pias3	547	653	562	749
Pias4	98	279	87	124
Pibf1	244	275	196	209
Picalm	8682	6812	8647	9715
Pick1	244	210	203	108
Pid1	507	1069	921	1798
Piezo1	441	1343	572	960
Piezo2	39	147	92	100
Pif1	73	128	33	27
Piga	85	56	53	21
Pigb	20	34	18	14
Pigbos1	296	161	162	119
Pigc	1026	1109	1301	1330
Pigf	448	253	292	225
Pigh	956	716	885	990
Pigk	1400	1660	1350	1870
Pigl	340	240	316	310
Pign	391	420	414	493
Pigo	378	249	423	294
Pigp	2514	1261	1757	1843

Pigq	1572	1767	1348	1684
Pigs	1653	1588	1781	1448
Pigt	2776	3199	2659	3167
Pigu	1104	1017	749	944
Pigv	402	295	355	299
Pigw	288	142	226	207
Pigx	1035	991	1542	1230
Pigy	1679	798	949	1233
Pih1d1	397	464	363	452
Pih1d2	647	391	548	501
Pik3ap1	332	284	435	1087
Pik3c2a	393	284	342	354
Pik3c2b	83	159	45	53
Pik3c2g	94	24	24	34
Pik3c3	605	650	582	812
Pik3ca	1372	666	946	907
Pik3cb	33	56	48	97
Pik3cd	751	832	880	1123
Pik3cg	309	512	501	816
Pik3ip1	591	767	700	516
Pik3r1	1801	2145	1691	1910
Pik3r2	895	1430	1056	971
Pik3r3	480	453	433	553
Pik3r4	826	1000	1125	883
Pik3r5	1	26	50	239
Pik3r6	141	277	208	185
Pikfyve	378	567	566	718
Pim1	607	624	627	842
Pim2	137	26	92	85
Pim3	1006	717	862	590
Pin1	1058	1361	927	902
Pin4	1302	1196	1518	1691
Pink1	1708	1921	900	1238
Pinlyp	1587	354	1685	278
Pinx1	275	514	342	345
Pip4k2a	821	1197	1220	1935
Pip4k2b	1516	1883	1553	1787
Pip4k2c	1347	1157	1274	747
Pip5k1a	456	642	707	708
Pip5k1b	48	33	36	22
Pip5k1c	782	1201	924	1250
Pir	299	477	361	345
Pirb	204	294	241	610
Pirt	15	55	60	123
Pisd	1038	1181	1323	1282
Pithd1	2698	2075	3211	2108
Pitpna	4922	6239	6121	6402

Pitpnb	4552	4133	4050	4043
Pitpnc1	535	794	719	1587
Pitpnm1	217	377	260	371
Pitpnm2	517	847	487	412
Pitpnm3	390	190	415	52
Pitrm1	1243	1511	1038	1383
Pitx1	14	30	20	22
Pitx2	80	148	72	61
Pitx3	992	349	423	839
Piwil2	15	41	51	70
Pja1	672	605	421	766
Pja2	1257	1348	1458	1751
Pkd1	44	337	165	223
Pkd2	402	847	558	701
Pkd2l1	2	8	3	22
Pkdcc	207	463	247	382
Pkhd1l1	14	38	105	0
Pkia	3261	1776	1240	2098
Pkib	164	196	120	143
Pkig	2044	2300	1777	1442
Pkm	11683	9760	4106	7495
Pkmyt1	1036	895	1163	1095
Pkn1	471	905	594	1099
Pkn2	1088	961	999	1030
Pkn3	202	720	396	324
Pknox1	168	576	222	215
Pknox2	315	506	271	375
Pkp1	16892	8077	13946	4416
Pkp2	127	123	36	56
Pkp3	3284	1955	2314	727
Pkp4	1330	1459	1461	1095
Pla1a	767	1278	928	1876
Pla2g12a	503	598	436	331
Pla2g12b	6	2	8	0
Pla2g15	1236	1386	1380	1612
Pla2g16	1048	1265	861	1231
Pla2g2a	266	1177	334	184
Pla2g2c	24	0	2	5
Pla2g2d	2220	4489	3128	4084
Pla2g2e	22	6	9	13
Pla2g2f	1373	389	421	482
Pla2g3	26	0	2	21
Pla2g4a	56	93	155	72
Pla2g4b	358	283	174	147
Pla2g4e	605	279	293	160
Pla2g4f	1605	585	302	167
Pla2g5	55	40	104	21

Pla2g6	272	447	319	355
Pla2g7	1722	1381	3075	9345
Pla2r1	125	120	63	77
Plaa	1666	1335	1304	1803
Plac8	440	297	363	398
Plac8l1	11	12	8	12
Plac9	586	1153	597	270
Plag1	40	24	60	12
Plagl1	23	27	39	64
Plagl2	390	288	465	281
Plat	3976	5942	6032	5685
Plau	778	1985	1118	1602
Plaur	1170	1828	1434	1432
Plb1	242	113	134	76
Plbd1	3366	2139	2820	2833
Plbd2	2083	2863	1956	2553
Plcb1	27	107	93	70
Plcb3	831	1298	976	1181
Plcb4	353	384	351	513
Plcd1	823	1179	1011	641
Plcd3	233	397	199	181
Plcd4	521	302	277	227
Plce1	147	369	211	262
Plcg1	2145	2331	2389	2380
Plcg2	869	624	892	1130
Plch2	1545	386	1035	255
Plcl1	43	28	62	77
Plcl2	521	465	430	919
Plcxd2	11	30	18	32
Pld1	1275	1078	1083	1870
Pld2	1005	1461	1177	1139
Pld3	2054	3647	3334	7664
Pld4	1651	1888	1804	2680
Plek	2118	1578	1930	2312
Plek2	1458	650	548	376
Plekha1	1176	1504	1583	1632
Plekha3	342	651	519	739
Plekha4	246	215	233	155
Plekha5	697	525	722	399
Plekha6	734	409	661	411
Plekha8	337	384	314	550
Plekhb1	532	205	438	441
Plekhb2	2372	2625	2672	4364
Plekhd1	3	69	15	1
Plekhf1	437	457	272	386
Plekhf2	1853	1052	1491	1628
Plekhg1	373	152	492	180

Plekhg2	312	656	430	607
Plekhg3	39	74	77	84
Plekhg4	153	255	278	657
Plekhg5	305	405	342	214
Plekhg6	651	455	526	483
Plekhh1	45	15	42	86
Plekhh2	150	215	230	201
Plekhh3	431	497	349	241
Plekhj1	839	931	624	681
Plekhm1	679	900	823	1126
Plekhm2	195	424	342	335
Plekhm3	365	373	474	686
Plekhn1	2067	819	1320	749
Plekho1	1375	1790	1644	3424
Plekho2	1475	2429	1503	3355
Plekhs1	47	29	38	62
Plet1	3695	894	3236	1049
Plg	41	8	21	0
Plgrkt	613	629	522	723
Plin1	52	2	36	8
Plin2	9314	8651	6954	7933
Plin3	2994	2337	2726	2267
Plin4	213	224	56	193
Plin5	141	44	47	57
Plk1	273	666	363	281
Plk2	1394	1324	1592	1555
Plk3	570	499	525	467
Plk4	99	228	271	183
Plk5	28	25	9	6
Pllp	33	50	45	72
Pln	110	89	95	95
Plod1	3031	4153	3972	5042
Plod2	234	1309	880	1314
Plod3	1103	2822	1520	2091
Plp1	97	74	156	113
Plp2	6518	6293	5319	7492
Plpp1	2419	3938	2278	2605
Plpp2	973	532	788	389
Plpp3	918	1923	1696	1076
Plpp4	23	39	32	25
Plpp5	2142	1968	1938	2459
Plpp6	961	510	776	707
Plpp7	1066	1044	558	743
Plppr2	414	245	208	427
Plppr3	12	1	26	13
Plppr5	41	21	21	60
Plrg1	1877	1348	1732	1267

Pls1	24	29	23	85
Pls3	7713	4578	5478	4800
Plscr1	338	619	516	794
Plscr3	1477	1811	1806	1875
Plscr4	366	262	242	318
Pltp	7552	10732	9981	12349
Plvap	2824	7224	5651	4140
Plxdc1	190	176	144	299
Plxdc2	3653	3817	4106	6145
Plxna1	1116	1407	1393	1342
Plxna2	486	607	438	253
Plxna3	256	301	159	224
Plxna4	250	96	352	80
Plxnb1	618	577	609	319
Plxnb2	2165	3662	2734	3883
Plxnb3	21	0	8	16
Plxnc1	307	110	263	442
Plxnd1	896	2873	2174	2345
Pm20d1	175	82	143	169
Pm20d2	915	556	936	533
Pmaip1	763	313	621	649
Pmch	76	48	55	81
Pmel	331	348	176	309
Pmepa1	3883	3046	3096	2019
Pmf1	513	746	620	540
Pmfbp1	105	38	193	125
Pml	789	753	626	1159
Pmm1	1622	1746	1535	1581
Pmm2	1101	1950	1174	1614
Pmp2	3	27	9	34
Pmp22	3656	6149	4642	5738
Pmpca	2416	2722	2450	1958
Pmpcb	1809	2027	1662	1868
Pms1	261	260	289	267
Pms2	517	562	584	671
Pmvk	1474	1139	844	919
Pnck	0	3	8	0
Pnisr	904	1092	1301	998
Pnkd	1275	1594	979	1189
Pnkp	472	402	490	327
Pnldc1	66	20	35	14
Pnmal2	1	66	8	8
Pnmt	17	17	15	61
Pnn	653	564	1040	484
Pno1	962	1224	1151	1379
Pnoc	1	0	0	19
Pnp	15031	14347	17704	18250

Pnpla3	243	109	233	209
Pnpla5	1835	847	838	1296
Pnpla6	446	417	418	412
Pnpla7	1207	1031	1025	1601
Pnpla8	1605	1201	1243	1572
Pnpo	452	246	289	419
Pnpt1	890	509	546	828
Pnrc1	1807	795	1315	1502
Pnrc2	2036	2259	2809	2660
Poc1a	50	150	87	85
Poc1b	375	412	366	373
Poc5	280	331	293	299
Podnl1	34	242	29	119
Podxl	829	2234	1616	1208
Podxl2	212	228	146	314
Pof1b	859	29	286	123
Pofut1	105	134	83	108
Pofut2	1162	2684	1640	2583
Pogk	1085	741	833	1044
Poglut1	743	514	863	640
Pogz	589	420	805	687
Pola1	228	248	253	295
Pola2	148	464	196	308
Polb	822	576	300	332
Pold1	105	169	101	123
Pold2	841	1340	1011	1193
Pold3	346	540	638	503
Pold4	1036	845	727	982
Poldip2	3266	3280	2556	3403
Poldip3	2390	2626	2603	2805
Pole	306	338	220	245
Pole2	142	144	119	51
Pole3	1247	1077	992	1048
Pole4	1056	888	1001	1028
Polg	1014	815	843	896
Polg2	219	220	206	114
Polh	385	523	515	444
Poli	0	73	21	53
Polk	16	18	65	52
Poll	427	622	307	439
Polm	240	366	259	420
Polq	70	92	30	13
Polr1a	419	721	712	687
Polr1b	325	667	460	374
Polr1c	1377	1641	1566	1292
Polr1d	4855	4524	4592	4506
Polr1e	451	645	570	486

Polr2a	1920	3372	3149	2939
Polr2b	1832	1628	1484	1647
Polr2c	1543	1756	1661	1703
Polr2d	967	874	818	1089
Polr2e	2014	2305	1702	1496
Polr2f	1276	1528	1095	1170
Polr2g	2823	2276	2923	2801
Polr2j	1396	1580	982	1182
Polr2k	435	439	504	517
Polr2m	1940	1856	1584	2245
Polr3a	432	646	486	632
Polr3b	464	543	369	376
Polr3c	687	732	630	622
Polr3d	1606	1570	1596	1670
Polr3e	672	731	566	543
Polr3f	560	462	685	623
Polr3g	261	144	200	187
Polr3gl	595	605	772	672
Polr3h	1069	1284	1146	805
Polr3k	1215	1113	665	1141
Polrmt	291	320	251	148
Pom121	43	83	47	0
Pomc	5	10	0	1
Pomgnt1	1415	2623	1521	2075
Pomgnt2	190	349	158	113
Pomk	1014	866	889	784
Pomp	14	0	26	0
Pomt1	710	853	358	876
Pomt2	446	515	513	468
Pon2	3608	3087	4515	4463
Pon3	526	641	480	751
Pop1	88	158	114	94
Pop4	1432	1408	1282	1460
Pop5	1416	933	1153	1017
Pop7	1228	899	847	909
Popdc2	441	713	439	415
Popdc3	711	557	325	447
Por	635	1961	493	1246
Porcn	4240	1844	2770	2701
Postn	19934	36306	41067	118823
Pot1	475	244	363	366
Pot1b	187	87	99	229
Potef	27	1	3	17
Pou2af1	9	12	27	0
Pou2f1	48	62	20	20
Pou2f3	428	132	123	49
Pou3f1	649	409	274	192

Pou3f3	2	0	0	39
Pou4f1	9	0	8	0
Pou5f1	3	6	8	1
Pou6f1	475	319	510	312
Pp2d1	370	289	153	189
Ppa1	3503	3516	3372	3501
Ppa2	1372	868	1151	1324
Ppan	658	738	382	321
Ppara	7	69	80	108
Ppard	347	588	445	406
Pparg	425	496	355	412
Ppargc1a	134	140	35	64
Ppargc1b	17	33	26	4
Ppat	0	257	133	77
Ppcs	653	581	621	705
Ppdpf	4642	2491	4071	1780
Ppfia1	10	112	69	11
Ppfia3	117	27	57	1
Ppfia4	130	579	381	109
Ppfibp1	2412	2949	2541	4290
Pphln1	450	452	441	509
Ppia	102	124	110	152
Ppib	11580	16647	12845	20737
Ppic	3150	4325	3656	8640
Ppid	400	296	421	158
Ppidl1	1188	1117	987	652
Ppie	690	919	614	641
Ppif	627	404	462	533
Ppig	1961	1752	2665	1721
Ppih	1451	1470	1348	1517
Ppil1	853	1156	918	949
Ppil2	819	955	628	938
Ppil3	1012	830	799	726
Ppil4	876	895	832	867
Ppil6	23	52	12	40
Ppip5k1	562	453	626	535
Ppip5k2	20	24	20	0
Ppl	2149	1690	1879	965
Ppm1a	3158	2946	2462	3338
Ppm1b	2868	1940	2180	2280
Ppm1d	653	410	597	352
Ppm1e	0	36	0	15
Ppm1f	949	1657	1026	1218
Ppm1g	2914	3752	3072	3566
Ppm1h	188	239	200	636
Ppm1j	219	159	98	115
Ppm1k	315	179	302	283

Ppm1l	929	711	825	901
Ppm1m	452	613	548	643
Ppm1n	243	210	188	320
Ppme1	828	969	1095	797
Ppox	942	914	953	738
Ppp1ca	7851	10726	7145	9122
Ppp1cb	4964	2324	2604	3000
Ppp1cc	6636	3892	3939	6148
Ppp1r10	507	1089	745	698
Ppp1r11	2671	1919	1833	1816
Ppp1r12a	1002	983	934	1242
Ppp1r12b	1168	1766	1231	906
Ppp1r12c	926	1681	1425	1553
Ppp1r13b	346	639	569	493
Ppp1r13l	2960	1641	1959	972
Ppp1r14a	45	159	21	6
Ppp1r14b	2337	3852	2367	2352
Ppp1r14c	1512	672	1592	554
Ppp1r14d	25	6	29	0
Ppp1r15a	1517	2044	2016	1926
Ppp1r15b	1650	1655	1738	1786
Ppp1r16a	201	214	203	170
Ppp1r16b	150	166	230	179
Ppp1r18	1645	2429	2219	3610
Ppp1r1a	1754	791	656	898
Ppp1r1b	80	24	108	24
Ppp1r1c	45	20	6	23
Ppp1r2	1990	1873	1739	2305
Ppp1r21	401	454	427	529
Ppp1r26	150	110	206	166
Ppp1r27	1945	1654	3036	1456
Ppp1r32	6	16	3	1
Ppp1r36	640	465	608	293
Ppp1r37	311	464	314	294
Ppp1r3a	865	254	238	541
Ppp1r3b	637	320	859	424
Ppp1r3c	2621	1034	1032	2879
Ppp1r3d	307	214	251	509
Ppp1r3g	10	20	3	0
Ppp1r42	5	14	3	0
Ppp1r7	1172	1407	1074	1115
Ppp1r8	1072	1145	1130	1123
Ppp1r9a	1	13	15	12
Ppp1r9b	1646	2579	2082	2642
Ppp2cb	3929	3000	3220	3100
Ppp2r1a	7141	6435	7133	6017
Ppp2r1b	2858	1598	2435	2585

Ppp2r2a	3710	2685	3012	3253
Ppp2r2b	89	11	51	34
Ppp2r2d	1195	944	1153	1239
Ppp2r3a	2245	2297	2166	1412
Ppp2r3b	47	113	38	61
Ppp2r3c	762	680	733	666
Ppp2r5a	1695	1559	1098	1233
Ppp2r5b	589	840	486	650
Ppp2r5c	3678	3014	3488	3962
Ppp2r5d	248	777	408	586
Ppp2r5e	1054	1016	1243	1230
Ppp3ca	3452	1215	1694	1397
Ppp3cb	2625	1902	1162	2456
Ppp3cc	1085	943	1064	1257
Ppp3r1	3591	2859	3229	3375
Ppp4c	2020	1971	1783	1415
Ppp4r1	1440	1894	1339	1981
Ppp4r2	1634	1291	1425	1860
Ppp4r3a	1274	1219	1345	1272
Ppp4r3b	1304	1056	1371	1310
Ppp4r4	0	16	42	7
Ppp5c	577	1119	578	982
Ppp6c	2939	1851	2303	2660
Ppp6r1	1769	3170	2108	2646
Ppp6r2	1114	1263	906	930
Ppp6r3	1744	1640	1866	1297
Pprc1	1368	1056	1208	1109
Ppt1	6271	5548	7300	19187
Ppt2	1027	994	800	1001
Pptc7	26	10	24	60
Ppwd1	576	505	522	633
Pqbp1	1354	2054	1896	2005
Pqlc1	1249	1321	1013	1123
Pqlc2	319	505	280	448
Pqlc3	1473	1462	2152	1888
Pradc1	693	631	569	683
Praf2	1774	3225	2406	4533
Pragmin	503	528	426	411
Pram1	248	276	254	509
Prap1	0	23	15	0
Prc1	982	1659	1317	1536
Prcc	1466	1700	1378	1533
Prcd	29	32	30	17
Prcp	911	1211	1462	2043
Prdm1	286	267	178	436
Prdm10	176	176	153	189
Prdm11	22	104	48	45

Prdm16	14	16	30	8
Prdm2	1303	1832	1380	1963
Prdm4	631	739	770	688
Prdm5	27	64	87	73
Prdm6	4	45	56	42
Prdm8	98	160	60	60
Prdm9	17	14	11	17
Prdx1	12315	7379	10955	7191
Prdx1l1	11275	10459	9969	10344
Prdx2	10362	9999	9201	9246
Prdx3	2442	2636	2018	2803
Prdx4	2636	3953	3125	5284
Prdx5	7377	9379	9137	18315
Prdx6	774	1650	1508	1663
Preb	1129	2046	1003	1301
Prelid1	2345	3474	2768	3270
Prelid2	113	29	80	61
Prelid3a	128	210	99	132
Prelid3b	1268	2041	1479	2013
Prelp	302	51	209	225
Prep	2226	2066	2255	1681
Prepl	1535	2452	1437	1290
Prex1	23	59	59	136
Prex2	144	280	339	219
Prf1	276	39	187	112
Prg3	3	0	5	14
Prickle1	1122	684	1344	1277
Prickle2	1129	570	1107	564
Prickle3	810	761	647	593
Prickle4	5213	4908	4670	4160
Prim1	1138	1367	1115	1171
Prim2	215	440	287	420
Prima1	61	72	30	17
Primpol	91	115	109	140
Prkaa1	1022	727	806	652
Prkaa2	1211	708	569	1138
Prkab1	1109	1117	1095	1175
Prkab2	1686	1186	1229	1749
Prkaca	2012	3116	1998	2051
Prkacb	1742	1528	1675	2329
Prkag1	1103	1561	1273	1630
Prkag2	513	572	480	662
Prkag3	425	852	406	332
Prkar1a	5916	6351	7244	5848
Prkar1b	77	49	53	58
Prkar2a	1269	1141	1351	1079
Prkar2b	532	259	402	515

Prkca	668	579	627	565
Prkcb	814	405	668	747
Prkcd	1516	1042	1491	2147
Prkcdbp	4245	6033	5687	4346
Prkce	13	18	11	2
Prkcg	61	15	29	48
Prkch	1064	891	1178	1174
Prkci	1993	1151	1810	972
Prkcq	652	256	245	381
Prkcsh	3703	4441	4494	4237
Prkcz	185	193	181	57
Prkd1	230	154	144	334
Prkd2	802	594	710	544
Prkd3	1241	953	1488	1625
Prkdc	1001	797	1074	919
Prkg1	1	0	3	0
Prkra	1271	976	1332	1604
Prkrip1	390	482	256	237
Prkx	607	686	793	858
Prmt2	511	631	593	555
Prmt3	649	784	522	610
Prmt5	1145	1877	1109	1065
Prmt7	882	907	667	911
Prmt9	559	556	677	986
Prnd	272	574	403	885
Prnp	11296	8217	9760	6853
Prob1	1040	1153	958	891
Proca1	211	174	236	176
Procr	723	1575	1186	1142
Prodh1	195	217	81	112
Prok2	67	60	54	41
Prom1	171	48	116	178
Prorsd1	391	325	346	321
Pros1	1041	1779	1465	2606
Prosc	1094	682	743	811
Proser1	380	357	402	430
Proser2	89	92	98	29
Proser3	326	370	453	431
Prox1	8	81	59	32
Prox2	33	22	29	0
Prp2l1	233	209	221	142
Prpf18	1449	1005	1414	1324
Prpf19	4619	6829	4038	5002
Prpf3	837	988	945	890
Prpf31	783	873	645	826
Prpf38a	1316	1289	1226	1338
Prpf38b	1082	1306	1723	1213

Prpf39	1028	648	714	808
Prpf4	640	839	629	649
Prpf40a	1506	1040	1590	1867
Prpf40b	288	440	217	386
Prpf4b	1436	741	942	915
Prpf6	1878	2011	1768	1818
Prpf8	4149	4426	3862	4367
Prph	25	88	38	19
Prps1	612	481	492	928
Prps2	772	625	814	833
Prpsap1	1235	1197	1013	919
Prpsap2	566	534	782	689
Prr11	34	57	8	12
Prr12	392	526	420	293
Prr13	5481	5649	6622	6623
Prr14	176	364	194	227
Prr14l	767	541	761	580
Prr15	78	54	50	158
Prr15l	79	20	29	27
Prr16	75	64	57	127
Prr18	9	27	26	55
Prr22	23	50	26	21
Prr27	11	11	3	1
Prr29	151	326	282	182
Prr3	170	485	227	120
Prr32	187	185	44	179
Prr33	1273	752	462	1048
Prr36	88	38	95	108
Prr5	91	176	102	156
Prr5l	206	458	361	329
Prr7	20	82	5	30
Prr9	115	41	236	0
Prrc1	995	1740	1140	1891
Prrc2a	1486	2771	1881	1923
Prrc2b	3723	3848	3778	2801
Prrc2c	105	171	184	149
Prrg1	55	42	57	107
Prrg2	378	180	310	213
Prrg4	38	16	8	36
Prrt1	564	373	489	746
Prrt3	16	36	54	12
Prrt4	269	446	226	132
Prrx1	3057	5182	4490	6150
Prrx2	378	739	475	726
Prss12	154	106	132	54
Prss22	1285	445	799	191
Prss23	2032	3546	2249	1590

Prss27	74	19	11	12
Prss35	1408	1172	1601	5977
Prss36	27	51	60	32
Prss39	11	0	17	4
Prss46	99	158	50	34
Prss53	1053	271	637	290
Prss8	332	167	261	108
Prtfdc1	782	729	826	832
Prune	779	902	1017	843
Prune2	276	419	638	435
Prx	79	113	159	163
Psap	32323	39583	43789	71066
Psapl1	5511	1388	2516	1680
Psat1	1049	1054	1028	795
Psca	24	0	51	298
Psd	24	6	8	16
Psd2	14	28	5	16
Psd3	399	632	484	364
Psd4	396	474	510	735
Psen1	1310	1591	1082	1860
Psen2	698	531	618	576
Psenen	5907	3815	3641	4583
Psip1	525	604	790	414
Pskh1	852	861	757	734
Psma1	5355	5378	5147	5505
Psma2	7468	6884	8210	7233
Psma3	1803	1171	1577	1788
Psma4	8705	7871	9349	7789
Psma5	5831	6606	5498	6195
Psma6	7706	5253	6614	6578
Psma7	8680	9639	7551	7627
Psmb1	11621	11214	9205	10598
Psmb10	1350	1371	1393	1481
Psmb2	5399	4642	4267	5012
Psmb3	7092	6547	5565	4965
Psmb4	10265	9211	10215	9036
Psmb5	5542	7279	4615	4398
Psmb6	918	1067	709	614
Psmb7	8859	8930	8623	8190
Psmb8	204	275	116	256
Psmb9	2232	1546	1763	1725
Psmc1	7123	8641	8591	7243
Psmc2	6059	5866	7071	5667
Psmc3	3882	6412	4280	3979
Psmc3ip	247	357	391	338
Psmc4	4533	5427	5564	4851
Psmc5	7966	8209	7709	6426

Psmc6	2998	2374	2555	2537
Psmd1	4159	5427	4184	4998
Psmd10	83	168	93	72
Psmd11	3716	3895	3904	3865
Psmd12	5766	5427	5749	4974
Psmd13	3358	4738	3874	3963
Psmd14	3427	2954	2836	3418
Psmd2	7644	9092	8052	7909
Psmd3	2641	4276	2710	2512
Psmd4	4489	5996	4861	4824
Psmd5	1129	1308	1393	1097
Psmd6	3838	3183	3658	3703
Psmd7	4646	5952	4766	4714
Psmd8	3889	5020	3751	3863
Psmd9	502	721	539	415
Psme1	8607	5666	7642	7882
Psme2	275	200	182	253
Psme3	3129	2764	2689	2796
Psme4	6745	4195	5800	6250
Psmf1	1140	1343	1202	1169
Psmg1	2360	1960	2165	2218
Psmg2	1040	1004	967	906
Psmg3	522	960	579	700
Psmg4	1470	1320	1231	1172
Pspc1	562	623	963	492
Psph	922	575	796	512
Pspn	3	2	8	0
Psrc1	75	108	62	44
Pstk	651	360	539	592
Pstpip1	627	851	594	1798
Pstpip2	44	47	45	48
Ptafr	585	984	1005	2139
Ptar1	29	55	53	48
Ptbp1	4415	5500	3700	4012
Ptbp2	265	167	345	205
Ptbp3	2404	2178	2164	2988
Ptcd1	317	402	429	234
Ptcd2	335	293	257	243
Ptcd3	1674	1496	2097	5475
Ptch1	34	24	45	59
Ptch2	73	107	80	75
Ptcra	65	0	56	12
Ptdss1	1503	1632	1554	1692
Ptdss2	1238	1643	1390	1936
Pten	4509	3106	3808	4909
Pter	475	416	620	625
Ptgds	43	39	27	17

Ptger2	149	152	196	505
Ptger3	343	90	269	228
Ptger4	496	356	361	309
Ptges	500	307	257	225
Ptges2	995	1094	791	909
Ptges3	5111	1877	1562	3564
Ptges3l	1079	1226	739	808
Ptges3l1	87	81	214	237
Ptgfr	54	124	90	119
Ptgfrn	5424	7813	7317	9782
Ptgir	393	581	644	413
Ptgis	100	732	152	20
Ptgr1	2057	1149	1345	466
Ptgr2	3339	2498	3199	2626
Ptgs1	583	988	970	832
Ptgs2	685	540	456	47
Pth1r	328	355	442	820
Pthlh	92	83	138	6
Ptk2	1550	839	1519	1487
Ptk2b	2695	1300	2066	1895
Ptk7	1675	2171	1926	2670
Ptma	10781	5477	6164	7724
Ptms	6112	9407	5778	5029
Ptn	1239	1431	1601	883
Ptp4a1	15	0	18	17
Ptp4a2	7727	7483	8763	10473
Ptp4a3	2416	2848	1256	1907
Ptpa	2675	4462	3027	3183
Ptpdc1	188	220	196	232
Ptpmt1	1540	997	1094	1055
Ptpn1	1470	1607	1172	1563
Ptpn11	3100	3603	2759	3736
Ptpn12	1769	1062	1450	2107
Ptpn13	377	591	500	275
Ptpn14	2248	918	1784	842
Ptpn18	834	860	1022	1605
Ptpn2	1370	1348	1464	1614
Ptpn21	542	556	475	310
Ptpn22	110	56	71	215
Ptpn23	337	496	357	341
Ptpn3	1137	515	849	676
Ptpn4	455	66	173	308
Ptpn6	1339	1810	1446	2685
Ptpn7	57	304	191	194
Ptpn9	1233	1550	1732	1608
Ptpra	1247	1908	1909	2290
Ptprb	661	1761	1524	1680

Ptprc	532	608	900	1683
Ptprcap	16	0	0	4
Ptprd	127	387	292	222
Ptpre	201	289	366	521
Ptprf	5519	3095	5195	2141
Ptprg	1150	1103	966	1088
Ptprj	815	978	1148	1872
Ptprk	2647	1736	2740	1291
Ptprm	388	681	597	910
Ptprn	27	605	169	276
Ptpro	1176	836	1222	2597
Ptprr	142	152	153	123
Ptprs	214	429	280	280
Ptprt	22	5	12	8
Ptpru	164	39	162	70
Ptprv	663	375	566	395
Ptprz1	127	236	68	68
Ptrf	12251	21939	16800	16028
Ptrh1	682	1003	737	848
Ptrh2	1371	1143	1198	897
Ptrhd1	485	452	430	370
Pts	465	383	438	558
Pttg1	601	735	943	735
Pttg1ip	3956	5565	4293	5663
Ptx3	151	607	141	1047
Puf60	4135	4286	3934	3625
Pum1	1137	897	1277	912
Pum2	3629	2109	3292	3373
Pum3	570	624	486	374
Purb	1059	905	769	1177
Purg	64	128	72	29
Pus1	677	820	587	395
Pus10	461	404	528	546
Pus3	363	342	289	450
Pus7	682	601	555	404
Pus7l	72	89	95	41
Pusl1	357	385	259	323
Pvalb	63691	14778	8367	24102
PVR	1386	2034	1602	1226
Pvrig	21	0	38	16
Pvt1	321	401	316	230
Pwp1	970	820	973	831
Pwp2	594	906	620	578
Pwwp2a	363	413	408	494
Pwwp2b	656	674	463	635
Pxdc1	1776	1858	1641	1866
Pxdn	7541	10355	9719	12059

Pxk	682	1156	945	1310
Pxmp2	522	382	391	314
Pxmp4	3217	1306	1590	2008
Pxn	2450	3445	2773	2509
Pxt1	25	34	18	23
Pxylp1	391	789	609	1095
Pycard	1497	1237	1175	1593
Pycr2	950	1216	1043	904
Pycrl	651	678	524	548
Pygb	1545	1029	1812	1439
Pygl	1232	868	1441	824
Pygm	43017	23825	12456	38751
Pygo1	17	19	44	36
Pygo2	1649	1577	1840	1798
Pym1	1342	1114	1070	756
Pyroxd1	1617	548	934	905
Pyroxd2	233	439	226	275
Pyy	175	63	32	19
Qars	7205	5923	5823	4601
Qdpr	1200	988	943	1018
Qpct	187	171	239	457
Qpctl	611	567	841	696
Qprt	141	287	430	558
Qrich1	844	833	1043	1002
Qrich2	5	7	6	4
Qrsl1	97	120	141	69
Qser1	775	641	540	818
Qsox1	2616	1718	1596	2702
Qtrt1	135	207	122	104
Qtrt2	50	134	98	56
R3hcc1	897	1594	1160	1251
R3hcc1l	579	312	454	468
R3hdm1	981	824	945	1291
R3hdm2	2790	2243	2325	2299
R3hdm4	3516	2931	3241	3053
Rab10	6271	4056	3807	6009
Rab11a	6133	4514	6115	6163
Rab11b	2980	2733	2711	2884
Rab11fip1	835	397	737	1429
Rab11fip2	3	14	5	0
Rab11fip3	681	890	910	533
Rab11fip4	215	74	184	240
Rab11fip5	1737	3546	1947	2947
Rab12	28	115	159	20
Rab13	2353	2718	3382	3987
Rab14	4533	3484	4129	4155
Rab15	1701	1314	1753	1161

Rab18	1263	1006	1062	1022
Rab19	23	0	21	0
Rab1a	5888	6114	6049	9548
Rab20	831	576	519	452
Rab21	2415	1593	2109	2458
Rab22a	2775	2649	2467	2021
Rab23	333	550	477	670
Rab24	1571	1293	1368	1229
Rab25	4032	2076	2994	1066
Rab26	66	120	98	85
Rab27a	368	404	489	722
Rab27b	46	51	42	0
Rab28	1856	1433	1765	2261
Rab29	791	715	685	895
Rab2a	5446	4689	4872	6350
Rab2b	1134	820	1159	1164
Rab30	233	435	325	343
Rab31	3695	5707	5439	8400
Rab32	449	602	436	1121
Rab33a	20	10	30	0
Rab33b	570	412	406	442
Rab34	2098	2629	2222	2723
Rab35	2031	2555	2086	2574
Rab36	4	18	9	1
Rab38	2400	851	1759	819
Rab3a	118	111	65	123
Rab3b	21	37	38	14
Rab3d	1411	1192	1323	632
Rab3gap1	1323	1290	1453	1257
Rab3gap2	241	398	292	460
Rab3il1	30	97	38	100
Rab3ip	320	399	331	386
Rab40b	289	167	143	221
Rab40c	590	611	605	592
Rab42	228	342	265	590
Rab43	14	53	26	12
Rab44	34	79	29	85
Rab4a	1017	770	644	968
Rab4b	914	1236	913	976
Rab5al1	81	185	135	183
Rab5b	5021	4797	5144	7360
Rab6a	3832	2837	3584	3848
Rab6b	40	64	15	47
Rab7a	5631	6362	5693	6431
Rab7b	1176	1107	1267	3054
Rab8a	2384	2386	1917	2419
Rab8b	1459	1291	1502	2350

Rab9a	1393	988	1128	1547
Rabac1	2049	2773	1651	1975
Rabep1	1035	1183	1404	1270
Rabep2	385	581	379	329
Rabepk	517	524	552	643
Rabgap1	1423	1064	1637	1013
Rabgap1l	817	460	393	307
Rabgef1	981	643	722	665
Rabggta	1640	1670	1229	567
Rabggtb	2175	2653	2076	1941
Rabif	244	185	236	182
Rabl2a	406	398	447	381
Rabl3	533	566	466	410
Rabl6	1051	1624	927	1562
Rac1	12194	13211	12416	14963
Rac2	1229	895	1154	2654
Rac3	144	39	80	115
Racgap1	6	25	14	22
Rack1	15036	15199	16218	14283
Rad1	351	316	263	280
Rad17	446	292	481	459
Rad18	306	313	277	399
Rad21	1812	2356	2821	1964
Rad23b	5049	3626	3757	5077
Rad50	644	672	739	547
Rad51	0	57	3	30
Rad51ap1	151	227	89	220
Rad51b	41	95	71	57
Rad51c	141	186	149	128
Rad51d	559	412	369	381
Rad52	3909	2321	3322	3549
Rad54b	7	13	54	19
Rad54l	762	475	609	488
Rad54l2	641	655	781	697
Rad9a	464	587	404	459
Rad9b	1369	1116	948	1190
Radil	32	42	42	18
Rae1	823	1030	823	786
Raet1e	21	36	53	41
Raf1	2006	2436	2589	2422
Rai1	861	738	769	756
Rai14	2141	1865	2738	3259
Rai2	288	198	310	248
Rala	3093	2302	3223	2871
Ralb	2768	3370	2267	2736
Ralbp1	1711	1705	1443	1611
Ralgapa1	645	283	554	494

Ralgapa2	32	66	50	17
Ralgapb	1441	1488	1634	1774
Ralgds	959	1360	1207	1110
Ralgps1	16	13	23	34
Ralgps2	4	0	9	0
Raly	5730	7346	6286	6060
Ramp1	2796	1104	1356	1153
Ramp2	1870	3956	3474	2757
Ramp3	330	569	483	491
Ran	8404	8264	6996	7969
Ranbp1	1488	1782	1443	1273
Ranbp10	798	977	730	1009
Ranbp17	16	58	23	64
Ranbp3	379	496	281	401
Ranbp3l	32	0	5	29
Ranbp6	184	116	181	224
Ranbp9	479	844	905	740
Rangap1	2914	3016	2809	2454
Rangrf	1789	1207	1041	1192
Rap1a	1338	2284	2028	2242
Rap1b	5943	4792	4706	9027
Rap1gap	55	78	51	44
Rap1gap2	247	287	311	320
Rap1gds1	2152	1989	3086	2566
Rap2b	123	93	153	215
Rap2c	1321	673	739	1029
Rapgef1	1805	2716	1888	2157
Rapgef2	352	363	317	304
Rapgef3	144	329	197	207
Rapgef4	89	114	71	169
Rapgef5	466	546	549	463
Rapgef6	59	99	89	29
Rapgefl1	625	415	504	201
Raph1	1	16	34	1
Rapsn	95	409	128	126
Rara	645	1159	686	778
Rarb	263	93	169	79
Rarg	2470	2062	2273	1428
Rarres1	1087	1492	1834	2241
Rarres2	7025	8271	8008	14255
Rars	2964	3117	3134	3049
Rars2	485	356	238	287
Rasa1	823	566	951	802
Rasa3	1263	1840	1435	1933
Rasal1	41	86	63	19
Rasal2	75	106	93	61
Rasal3	415	198	328	444

Rasd1	165	176	182	169
Rasd2	38	54	23	26
Rasef	10	42	12	7
Rasgef1a	19	5	20	8
Rasgef1b	1060	657	910	1269
Rasgef1c	12	28	12	0
Rasgrf1	14	32	21	22
Rasgrf2	15	21	50	20
Rasgrp1	28	52	24	29
Rasgrp2	139	177	129	143
Rasgrp3	300	496	582	692
Rasgrp4	94	386	183	163
Rasip1	499	1020	841	646
Rasl10a	2517	1860	2257	1650
Rasl11a	62	80	89	63
Rasl11b	425	268	349	353
Rasl12	261	301	370	666
Rasl2-9	79	204	119	145
Rassf1	1327	1056	1420	1304
Rassf10	653	162	333	190
Rassf2	796	1084	1240	1314
Rassf3	2241	1934	1693	1935
Rassf4	1428	1229	2300	5731
Rassf5	1000	598	892	811
Rassf6	107	135	80	58
Rassf7	506	305	317	191
Rassf8	425	730	411	880
Rassf9	420	111	426	141
Raver1	1631	1663	1485	1425
Raver2	505	240	516	130
Rb1	954	985	1058	880
Rb1cc1	1446	794	1107	1233
Rbak	158	136	187	225
Rbbp4	1935	1832	1381	1373
Rbbp5	604	728	814	679
Rbbp6	1048	1071	1119	905
Rbbp7	5024	4272	4533	4722
Rbbp8	324	591	597	402
Rbbp8nl	113	86	166	87
Rbbp9	185	135	198	247
Rbck1	1369	1296	1228	1448
Rbfa	920	813	713	745
Rbfox1	835	1347	970	642
Rbfox2	29	253	111	69
Rbks	318	273	303	262
Rbl1	24	60	17	14
Rbl2	182	295	288	351

Rbm10	1132	1282	1154	1132
Rbm14	2233	1641	2103	1787
Rbm15	590	252	290	361
Rbm17	1972	2286	2383	2159
Rbm18	861	1219	1047	1343
Rbm19	53	245	75	122
Rbm20	367	600	162	204
Rbm22	1192	1407	1381	1260
Rbm24	884	1158	680	759
Rbm25	919	505	909	787
Rbm25l1	406	451	579	351
Rbm26	698	378	447	591
Rbm27	503	417	542	554
Rbm28	449	547	653	498
Rbm3	6557	8987	10225	9898
Rbm33	967	925	1326	1101
Rbm34	495	398	423	215
Rbm38	486	878	275	443
Rbm39	19	8	5	18
Rbm41	26	45	38	4
Rbm42	1518	2008	1228	1498
Rbm43	254	316	293	218
Rbm44	0	7	0	6
Rbm45	957	702	930	791
Rbm46	8	0	15	4
Rbm47	536	302	450	712
Rbm48	117	195	166	193
Rbm4b	473	441	354	423
Rbm5	2214	1701	2260	2083
Rbm6	1359	1273	1617	1256
Rbm7	881	862	883	1149
Rbm8a	3164	3621	3435	3081
Rbms1	857	817	814	742
Rbms2	3732	4873	4410	5130
Rbms3	224	172	175	201
Rbmx	1358	1138	1526	996
Rbmx2	403	445	402	491
Rbmxl1	36	114	59	74
Rbmxrtl	1127	1129	1055	965
Rbp1	7659	2631	6551	3670
Rbp2	5022	1827	3733	852
Rbp4	468	163	388	40
Rbp7	15488	6125	5636	12059
Rbpj	1565	1810	1444	2774
Rbpjl2	4	3	35	27
Rbpms	549	1146	1032	1274
Rbpms2	167	222	235	107

Rbsn	551	589	614	579
Rbx1	6654	4960	4920	4436
Rc3h1	964	914	1094	957
Rc3h2	1371	955	1774	1217
Rcan1	3432	3341	3318	3049
Rcan2	197	282	184	316
Rcan3	13	11	29	53
Rcbtb1	592	616	624	804
Rcbtb2	446	499	509	759
Rcc1	2092	1501	1332	1134
Rcc1l	670	785	679	652
Rcc2	4134	4088	4727	3231
Rccd1	8	49	11	0
Rce1	950	770	978	815
Rchy1	1191	1237	1106	1145
Rcl1	1040	1131	949	1032
Rcn1	10552	11256	14485	18298
Rcn2	1021	1285	1089	1411
Rcn3	2271	7868	3686	8124
Rcor1	660	651	492	750
Rcor2	4	0	8	8
Rcsd1	1427	2022	1186	1860
Rd3l	14	11	20	30
Rdh10	462	379	451	306
Rdh11	956	1010	934	961
Rdh12	1124	418	530	312
Rdh13	239	256	274	279
Rdh14	553	472	399	461
Rdh16	2997	1227	1651	2352
Rdh5	31	681	144	60
Rdh7	2451	1520	934	1279
Rdm1	328	314	326	187
Rdx	1190	1164	1359	1360
Rec8	37	28	46	99
Reck	319	439	331	333
Recql	1073	1136	1397	922
Recql4	142	238	85	79
Recql5	458	293	397	411
Reep1	360	806	296	314
Reep2	35	117	66	16
Reep3	1694	1754	2103	1920
Reep4	1215	1145	1228	691
Reep6	279	205	150	179
Reg3b	12	13	6	0
Reg3g	49	48	0	18
Rel	112	94	188	104
Rela	1778	1884	1772	2123

Relb	668	512	555	522
Rell1	914	777	903	1185
Rell2	152	178	184	146
Reln	52	125	119	103
Relt	206	389	274	279
Rem1	228	605	323	345
Ren	0	97	54	104
Renbp	835	1026	778	1393
Rep15	7	9	8	11
Repin1	430	340	390	581
Reps1	60	103	114	98
Reps2	61	114	122	375
Rer1	3747	3830	3644	4220
Rere	1880	1784	1541	2081
Rerg	330	353	400	239
Rergl	14	23	3	6
Rest	827	604	730	836
Ret	667	264	623	276
Retn	351	148	435	11
Retnlg	28	10	32	38
Retsat	3498	3368	3739	3742
Rev1	597	742	576	640
Rev3l	349	200	345	259
Rex2	170	101	77	149
Rexo1	288	440	268	277
Rexo2	3092	3581	3603	4594
Rexo4	1481	1494	1502	1429
Rfc1	1318	1095	1279	1221
Rfc2	462	792	518	499
Rfc3	661	792	566	926
Rfc4	3168	1713	1893	1997
Rfc5	749	860	721	699
Rfesd	303	246	299	197
Rffl	1470	1020	1355	1333
Rfk	502	503	414	408
Rfng	579	802	557	809
Rft1	488	582	484	716
Rftn1	876	1201	960	1589
Rftn2	202	391	280	474
Rfwd2	3365	2741	2943	3599
Rfwd3	451	632	490	626
Rfx1	362	303	262	401
Rfx2	244	284	262	294
Rfx3	69	34	45	67
Rfx5	76	119	141	95
Rfx7	846	314	527	554
Rfx8	41	86	57	55

Rfxank	315	420	216	364
Rgcc	203	404	147	279
RGD13029	601	643	486	419
RGD13030	2371	2287	1796	1701
RGD13045	669	585	447	473
RGD13045	394	236	370	337
RGD13046	118	153	140	142
RGD13046	178	122	78	51
RGD13046	2417	3037	3072	4833
RGD13047	9714	9014	9981	10726
RGD13047	271	334	357	222
RGD13047	10	9	11	0
RGD13048	17	0	29	14
RGD13048	142	817	364	541
RGD13049	86	157	128	100
RGD13049	41	52	24	61
RGD13050	104	158	158	108
RGD13050	484	343	524	467
RGD13051	534	425	412	522
RGD13051	1132	1129	1174	1234
RGD13052	17	4	15	7
RGD13053	434	240	211	155
RGD13053	1175	1090	1157	1181
RGD13054	373	218	313	244
RGD13054	773	1022	806	1110
RGD13055	516	344	441	373
RGD13056	56	105	99	43
RGD13057	11	50	44	29
RGD13057	389	271	348	166
RGD13058	82	101	83	485
RGD13059	39	85	14	19
RGD13059	344	149	256	230
RGD13060	2396	1393	1926	1667
RGD13060	242	206	129	189
RGD13060	5	11	3	0
RGD13061	4698	3724	4049	4159
RGD13062	713	687	487	731
RGD13062	103	61	113	97
RGD13062	3	23	11	0
RGD13065	660	609	551	576
RGD13065	1423	1513	1228	1111
RGD13067	377	284	372	211
RGD13067	495	334	519	475
RGD13067	32	0	11	4
RGD13069	140	237	194	261
RGD13069	1071	1214	1171	501
RGD13071	599	739	1043	976

RGD13071	89	103	181	256
RGD13072	698	569	501	658
RGD13073	296	366	254	301
RGD13074	18	11	8	0
RGD13074	163	66	149	186
RGD13075	803	1197	793	667
RGD13076	76	108	83	72
RGD13077	678	735	728	726
RGD13077	5080	3916	3678	5319
RGD13078	841	783	811	646
RGD13079	2546	4183	4031	4651
RGD13079	421	344	325	399
RGD13080	10	36	6	3
RGD13081	649	632	667	507
RGD13081	323	230	262	233
RGD13081	1000	1329	928	847
RGD13081	412	243	345	337
RGD13084	754	1095	936	964
RGD13084	1225	1292	1252	1259
RGD13085	1296	488	391	113
RGD13086	575	538	597	594
RGD13087	358	574	654	652
RGD13087	18	4	3	32
RGD13088	43	30	5	23
RGD13090	12	7	27	39
RGD13090	398	507	546	569
RGD13091	2673	1801	1861	2310
RGD13091	1820	1714	1596	1627
RGD13093	775	545	846	252
RGD13095	168	307	373	420
RGD13095	1723	1226	1049	1485
RGD13095	173	330	236	311
RGD13096	1243	1347	1521	1976
RGD13096	49	38	50	17
RGD13096	1785	1949	1887	919
RGD13097	1089	867	1076	1094
RGD13097	2092	1256	1598	2033
RGD13097	257	114	80	144
RGD13098	1356	749	1149	1200
RGD13099	814	725	800	1249
RGD13100	39	23	18	42
RGD13101	265	318	244	421
RGD13101	530	841	536	710
RGD13101	14	15	29	79
RGD13102	78	24	81	23
RGD13102	170	215	308	199
RGD13102	31	11	8	0

RGD13103	86	183	86	119
RGD13103	2647	3870	3372	4033
RGD13104	268	309	363	250
RGD13104	100	123	164	150
RGD13105	864	432	484	527
RGD13105	387	406	376	318
RGD13105	1136	1656	1696	1137
RGD13107	1431	1024	900	834
RGD13108	286	214	284	189
RGD13108	261	421	357	354
RGD13109	1602	201	492	309
RGD13109	151	85	152	191
RGD13110	25	29	20	33
RGD13111	433	401	340	458
RGD13111	506	328	352	438
RGD13112	14	4	26	0
RGD13113	877	928	978	1017
RGD13115	70	107	125	176
RGD13115	132	161	236	322
RGD13117	2349	2497	2272	2833
RGD13117	1011	802	875	799
RGD13117	333	356	236	203
RGD13117	744	511	572	663
RGD13117	163	269	215	325
RGD13118	1603	1696	1617	2152
RGD13118	852	719	628	638
RGD13118	1842	613	1473	534
RGD13118	1433	1515	1086	1459
RGD13119	652	1193	784	1310
RGD13120	62	21	159	242
RGD13591	459	296	445	511
RGD13591	568	694	390	485
RGD13591	907	420	718	581
RGD13592	5094	3616	3024	2587
RGD13593	46	34	35	10
RGD13595	103	134	135	84
RGD13596	1108	904	1137	958
RGD15595	8	14	32	12
RGD15595	163	354	278	725
RGD15596	3475	1197	1631	2509
RGD15596	221	23	41	283
RGD15597	133	258	289	218
RGD15597	43	34	50	22
RGD15597	1574	1773	1497	1388
RGD15598	26	24	11	11
RGD15598	675	1201	1327	1648
RGD15598	1577	2651	2415	2618

RGD15599	535	788	838	688
RGD15599	103	48	96	92
RGD15599	1165	1680	1241	1523
RGD15599	7	10	6	0
RGD15600	103	215	117	179
RGD15600	4390	4065	3184	3557
RGD15600	67	31	75	0
RGD15600	551	474	331	510
RGD15601	5476	1459	1565	3106
RGD15601	11	4	2	1
RGD15602	4858	4350	4243	3942
RGD15602	605	367	468	402
RGD15602	225	209	247	187
RGD15603	8	0	35	10
RGD15603	654	913	858	787
RGD15603	82	86	99	117
RGD15604	1824	1733	1641	2464
RGD15604	64	147	116	155
RGD15604	39	76	95	285
RGD15604	310	391	268	353
RGD15604	6	24	10	11
RGD15605	39	80	135	105
RGD15605	22	5	42	8
RGD15607	27	45	69	20
RGD15607	91	0	23	13
RGD15607	23	31	8	0
RGD15607	2	32	48	0
RGD15608	1042	1485	1022	1095
RGD15608	78	226	209	87
RGD15608	348	353	308	470
RGD15608	44	37	21	7
RGD15609	75	182	102	64
RGD15610	5	12	11	0
RGD15611	0	14	8	6
RGD15611	732	785	648	743
RGD15611	16	0	6	0
RGD15611	1089	1368	1267	1687
RGD15611	364	397	373	385
RGD15611	492	359	260	447
RGD15612	109	117	143	114
RGD15612	26	13	14	0
RGD15614	37	32	35	0
RGD15615	1684	1544	1211	1145
RGD15616	4943	2841	8126	6458
RGD15616	1091	1517	1262	973
RGD15616	173	206	179	240
RGD15616	49	10	263	0

RGD15617	703	1457	1082	1519
RGD15617	16	95	86	195
RGD15617	23	1	11	26
RGD15617	21	73	45	42
RGD15617	62	33	45	43
RGD15617	50	48	72	48
RGD15618	3	0	9	0
RGD15618	35	34	24	22
RGD15619	36	23	33	31
RGD15619	40	74	51	25
RGD15620	13	4	18	6
RGD15620	375	679	724	913
RGD15621	59	34	116	100
RGD15621	749	857	654	844
RGD15621	591	685	612	842
RGD15621	838	774	871	788
RGD15621	5	0	2	6
RGD15621	113	177	193	1283
RGD15622	13	9	30	8
RGD15622	1026	1022	1131	1020
RGD15622	282	245	239	382
RGD15622	69	139	105	81
RGD15623	54	98	116	66
RGD15623	822	1011	1256	2062
RGD15623	157	73	117	58
RGD15623	0	1	0	1
RGD15624	22124	12672	11737	10817
RGD15624	802	1064	242	401
RGD15624	1256	1835	1204	1422
RGD15624	210	161	137	481
RGD15624	69	17	66	64
RGD15626	139	123	110	192
RGD15626	153	235	170	160
RGD15626	30	36	69	91
RGD15626	376	228	287	238
RGD15626	93	45	81	20
RGD15626	8696	9690	4159	7380
RGD15627	617	650	821	744
RGD15627	8	12	8	10
RGD15628	14	12	11	11
RGD15629	0	10	23	20
RGD15629	2953	2446	2522	2834
RGD15630	43	103	35	88
RGD15630	28	56	53	80
RGD15630	4	0	3	20
RGD15630	4438	315	3042	760
RGD15630	705	609	400	781

RGD15631	36	32	26	12
RGD15632	53	84	50	38
RGD15632	5	9	3	0
RGD15632	828	1649	516	539
RGD15633	2	1	15	4
RGD15633	1	9	20	18
RGD15633	981	1025	1052	728
RGD15633	3	34	18	24
RGD15633	15	12	44	47
RGD15633	871	920	733	893
RGD15634	52	34	65	22
RGD15635	617	896	614	433
RGD15636	2812	4588	1471	2962
RGD15636	21	82	39	37
RGD15636	5	78	18	12
RGD15636	350	152	135	54
RGD15637	69	135	172	196
RGD15637	3	22	5	0
RGD15638	6614	7517	6261	7651
RGD15639	37	33	62	156
RGD15639	204	95	108	87
RGD15639	20	24	21	15
RGD15640	33	103	22	29
RGD15640	2755	1918	1754	1881
RGD15641	71	99	87	300
RGD15641	116	32	69	77
RGD15642	65	14	18	66
RGD15643	5	21	9	0
RGD15643	758	944	993	887
RGD15644	169	132	162	258
RGD15644	15	1	3	0
RGD15644	1332	838	901	1520
RGD15644	237	354	260	319
RGD15644	573	511	651	385
RGD15644	25	15	21	5
RGD15645	366	214	298	499
RGD15645	4	14	33	0
RGD15645	6	2	5	15
RGD15646	2184	1773	814	591
RGD15646	14	81	59	50
RGD15646	194	377	178	192
RGD15647	3	0	0	12
RGD15648	37	122	56	197
RGD15648	543	561	596	634
RGD15648	222	74	122	75
RGD15648	195	319	236	254
RGD15648	2090	1718	849	1567

RGD15649	11	14	2	8
RGD15649	9	4	2	0
RGD15649	17445	12807	10788	10959
RGD15650	2244	1625	1736	1778
RGD15650	441	376	296	230
RGD15650	1	8	3	0
RGD15650	164	148	152	280
RGD15651	14	10	5	5
RGD15651	41	43	69	62
RGD15652	111	247	196	220
RGD15653	178	22	110	92
RGD15653	1181	1211	1110	1102
RGD15653	58	21	12	36
RGD15654	263	190	175	215
RGD15655	537	995	871	535
RGD15655	15	35	6	0
RGD15656	259	231	436	421
RGD15656	36	57	15	26
RGD15656	3336	1831	2371	1859
RGD15656	143	210	45	56
RGD15656	410	285	313	226
RGD15656	9	32	5	10
RGD15657	265	236	110	136
RGD15657	1748	1292	1598	1471
RGD15657	1306	1051	1001	808
RGD15657	151	81	126	306
RGD15658	13	66	47	30
RGD15660	27	90	17	66
RGD15660	31	328	42	80
RGD15660	168	79	172	170
RGD15660	525	309	388	394
RGD15661	0	2	0	1
RGD15661	2	0	2	0
RGD15662	59	38	33	0
RGD15662	601	538	545	387
RGD15662	170	194	241	167
RGD15663	938	905	856	755
RGD15663	215	184	205	185
RGD15663	1292	446	673	1102
RGD15663	2419	1685	620	917
RGD15663	103	60	63	79
RGD15664	485	1067	1029	935
RGD62109	1082	1493	1007	1803
RGD73502	1957	1747	1613	1491
RGD73506	3320	3767	3864	3683
Rgl1	914	1516	1479	1757
Rgl2	5511	4481	5240	5305

Rgma	1453	1845	1130	913
Rgmb	1214	1021	1089	1138
Rgn	30	11	12	12
Rgp1	1249	1346	1485	1294
Rgs1	452	404	712	2294
Rgs10	1976	2285	2237	4334
Rgs12	2126	1378	1777	1033
Rgs14	176	245	257	250
Rgs16	158	251	348	145
Rgs18	376	257	457	604
Rgs19	795	1172	934	1322
Rgs2	1779	1453	1702	2148
Rgs20	173	80	78	52
Rgs22	3	35	8	8
Rgs3	677	1292	1140	1291
Rgs4	287	706	445	460
Rgs5	8947	11628	16633	15326
Rgs6	33	8	6	0
Rgs7	22	11	20	38
Rgs7bp	57	28	45	21
Rhbdd1	737	520	806	724
Rhbdd2	455	567	527	556
Rhbdd3	241	337	304	259
Rhbdf1	405	586	453	293
Rhbdf2	671	472	525	536
Rhbdl1	94	32	42	16
Rhbdl2	355	214	200	75
Rhbdl3	584	307	487	281
Rhbg	639	287	346	217
Rhcg	70	0	35	13
Rheb	1799	1715	1374	1480
Rhebl1	147	76	140	121
Rhno1	372	345	352	275
Rhoa	12833	17831	16595	20074
Rhob	4976	5587	5260	5686
Rhobtb1	136	226	202	234
Rhobtb2	127	145	72	43
Rhobtb3	152	216	241	354
Rhoc	2805	6764	4843	5236
Rhod	314	187	254	141
Rhof	221	367	271	255
Rhog	3107	2890	3795	3964
Rhoj	1462	2476	2018	2752
Rhoq	1838	1908	1577	3092
Rhot1	1183	1323	1353	1126
Rhot2	1037	1004	886	756
Rhou	1713	686	1157	1687

Rhov	1156	642	1193	389
Rhox5	21	22	20	0
Rhpn1	19	26	29	20
Rhpn2	40	42	20	27
Ribc1	121	131	107	96
Ric1	85	185	173	125
Ric3	27	10	12	50
Ric8a	1704	1840	1447	1578
Ric8b	408	545	460	395
Rictor	431	268	552	285
Rida	669	434	665	673
Rif1	389	166	247	192
Rilp	334	353	245	396
Rilpl1	1451	1192	1140	1360
Rilpl2	525	590	716	1280
Rimbp2	38	32	21	0
Rimbp3	0	34	2	20
Rimkla	132	195	238	288
Rimklb	29	17	9	25
Rims1	12	13	6	0
Rin2	4830	4066	4553	6837
Rin3	416	972	615	1338
Ring1	638	586	429	487
Rinl	126	34	119	70
Rint1	407	486	370	595
Riok1	759	926	772	923
Riok2	627	778	837	593
Riok3	2928	2911	2925	3802
Ripk1	1177	1119	1462	2044
Ripk2	724	535	818	1011
Ripk3	537	1672	703	1237
Ripk4	1023	326	543	210
Ripply1	71	186	248	111
Ripply3	52	55	23	4
Rit1	865	899	877	986
Rita1	348	436	212	240
Rlf	763	751	1001	925
Rln1	0	14	12	24
Rmdn1	616	458	617	555
Rmdn2	136	123	117	132
Rmdn3	23	35	6	10
Rmi1	757	662	710	870
Rmi2	5	4	12	31
Rmnd1	984	813	648	671
Rmnd5a	1647	1684	1881	2438
Rmrp	4	53	9	11
Rnase10	26	0	45	20

Rnase12	1610	279	1124	1389
Rnase13	44	19	8	42
Rnase1l1	39	30	23	5
Rnaseh1	347	549	445	472
Rnaseh2a	1439	1301	949	1314
Rnaseh2b	854	890	952	1199
Rnaseh2c	98	139	113	57
Rnasek	4664	4070	3737	5094
Rnasel	438	446	582	835
Rnaset2	919	1230	1660	3059
Rnd1	105	148	108	131
Rnd2	311	450	447	446
Rnd3	1274	962	1327	1043
Rnf10	5509	6477	5011	5401
Rnf103	760	530	662	920
Rnf111	692	348	557	576
Rnf112	12	27	48	18
Rnf113a2	352	317	296	378
Rnf114	1483	1469	1443	1853
Rnf115	1724	1761	1497	1604
Rnf11l1	96	162	105	74
Rnf122	978	739	918	610
Rnf123	1219	1588	1093	1441
Rnf125	13	9	14	11
Rnf126	330	353	235	149
Rnf128	103	79	84	349
Rnf13	22	80	105	132
Rnf130	1619	1444	1866	3377
Rnf135	177	251	316	322
Rnf138	705	298	465	584
Rnf14	2243	1374	1762	1633
rnf141	615	290	808	624
Rnf144a	414	456	590	1030
Rnf144b	1848	988	1369	953
Rnf145	1600	1426	1878	1580
Rnf146	867	568	703	897
Rnf149	1536	1383	1777	2496
Rnf150	193	168	229	390
Rnf151	310	386	302	198
Rnf152	300	151	477	211
Rnf157	151	196	191	165
Rnf166	1232	923	978	1083
Rnf167	2405	2013	2031	2043
Rnf168	407	240	271	456
Rnf169	360	444	428	726
Rnf170	622	514	528	714
Rnf180	42	32	44	8

Rnf181	1787	1205	1702	1212
Rnf183	8	4	21	43
Rnf185	677	813	715	824
Rnf187	3505	3633	3161	3138
Rnf19a	1015	647	945	687
Rnf19b	1865	1499	2487	2079
Rnf2	614	671	725	642
Rnf20	567	595	597	576
Rnf207	110	111	92	114
Rnf208	114	54	117	17
Rnf212	13	10	0	20
Rnf213	1417	1134	1324	1674
Rnf214	124	227	271	204
Rnf215	920	767	730	956
Rnf216	352	588	516	565
Rnf217	1953	1561	1784	1419
Rnf219	203	265	232	179
Rnf220	1219	1578	1121	1119
Rnf222	39	0	27	0
Rnf223	132	19	63	8
Rnf225	361	60	117	91
Rnf25	816	840	835	787
Rnf26	820	743	748	706
Rnf31	472	550	430	493
Rnf32	41	56	86	92
Rnf34	313	334	382	412
Rnf38	508	509	575	389
Rnf39	30	166	104	53
Rnf4	1798	2458	2717	3432
Rnf40	171	416	251	168
Rnf41	992	1050	981	1244
Rnf43	14	36	12	12
Rnf44	513	883	758	884
Rnf5	719	988	901	951
Rnf6	657	868	876	690
Rnf7	584	845	259	443
Rnf8	309	350	412	331
Rnft1	883	750	931	1190
Rnft2	60	117	102	72
Rngtt	423	405	391	375
Rnh1	4675	6618	5043	5796
Rnls	325	380	606	824
Rnmt	1212	1171	1241	1260
Rnpc3	381	173	405	332
Rnpep	3595	3583	3182	3229
Rnpepl1	2175	2580	1822	2230
Rnps1	3380	3711	3786	3219

Robo1	64	56	72	47
Robo2	432	152	197	226
Rock2	1937	2024	2052	2119
Rogdi	959	1218	1089	843
Rom1	56	70	61	61
Ropn1l	7	21	21	12
Ror1	10	0	24	3
Ror2	153	288	314	433
Rora	1012	472	451	558
Rorb	23	8	12	5
Ros1	4	34	20	21
Rp2	123	156	191	319
Rp9	359	438	373	502
Rpa1	1350	1692	1729	1843
Rpa2	696	1042	873	921
Rpa3	405	437	597	395
Rpain	688	409	495	449
Rpap1	396	402	351	173
Rpap2	540	548	630	705
Rpap3	1197	854	951	728
Rpe	606	679	814	789
Rpf1	700	784	759	737
Rpf2	1020	1213	1450	1295
Rpgr	74	102	77	67
Rpgrip1	242	42	139	0
Rpgrip1l	159	124	152	302
Rph3al	266	245	194	127
Rpia	1402	921	1113	1217
Rpl10	11498	9196	12568	12166
Rpl10a	4959	3836	3197	3322
Rpl10l	3	72	15	16
Rpl11	24981	22515	21530	23690
Rpl12	2184	2717	3026	3315
Rpl13	1875	2105	817	969
Rpl13a	8243	6059	7752	7152
Rpl14	7743	9798	8723	9083
Rpl15	18554	12633	13203	13430
Rpl17	4293	4140	782	752
Rpl18	7145	8975	8000	7907
Rpl18a	18941	19411	15756	14400
Rpl19	4753	5522	4014	3684
Rpl21	2082	1477	1744	1522
Rpl22	3464	3684	3023	2747
Rpl22l1	3758	2059	2630	2483
Rpl23	36431	28091	33213	29855
Rpl23a	10017	5326	13102	10623
Rpl24	13028	12259	12653	11657

Rpl26	2726	2204	415	924
Rpl27	2230	2670	1171	1086
Rpl27a	9364	5820	6846	7412
Rpl28	9410	9119	5705	5080
Rpl29	10481	10628	8593	5929
Rpl3	44720	43853	42387	42384
Rpl30	1659	1398	3074	1969
Rpl31	1094	1115	1419	1116
Rpl32	1164	947	1023	761
Rpl34	1076	1137	1353	1344
Rpl35	7587	8615	7977	6399
Rpl36	34	111	55	33
Rpl36a	27	21	21	18
Rpl36al	8250	6466	6424	5618
Rpl37a	16165	13074	8335	7484
Rpl37a-ps1	95	109	128	67
Rpl39l	9	10	11	27
Rpl3l	5474	1983	825	3135
Rpl4	56011	38700	54905	43401
Rpl41	77950	69319	56782	60568
Rpl5	15701	13356	16447	13812
Rpl6	1593	1044	71	99
Rpl7	29371	22283	29371	24809
Rpl7l1	4202	4120	4196	4168
Rplp0	47299	44193	46708	38209
Rplp1	546	776	193	275
Rpn1	4930	7815	6784	8538
Rpn2	8598	10371	10403	13425
Rpp14	1058	1292	897	1401
Rpp21	1186	1464	1007	734
Rpp25	98	63	95	144
Rpp25l	338	380	289	186
Rpp30	493	448	284	357
Rpp38	1050	447	826	725
Rpp40	792	593	648	643
Rprd1a	1657	1205	1404	1327
Rprd1b	562	612	645	622
Rprd2	791	899	865	746
Rprml	185	92	63	97
Rps10	2809	3015	2862	2676
Rps10l1	5375	6660	4095	3169
Rps11	39446	27707	36791	33366
Rps13	37	10	5	0
Rps14	266	100	141	86
Rps15	7591	7906	6426	4863
Rps15a	1154	1462	886	1078
Rps16	15040	15198	10846	12004

Rps17	2208	1639	5210	3029
Rps18	59	68	81	56
Rps19bp1	1292	1806	1229	1075
Rps2	45	36	2	0
Rps21	580	422	923	937
Rps23	4190	4107	4750	3166
Rps24	6098	5842	8254	7144
Rps25	2679	1599	1727	1357
Rps26	1728	1275	1196	1206
Rps27	5898	3299	8826	5594
Rps27a	166	164	89	54
Rps27l	4355	4047	3442	4800
Rps28	18	2	0	19
Rps29	8757	3258	14114	8503
Rps3	19808	19414	21533	20990
Rps3a	1245	506	2054	1077
Rps4x	28	8	20	0
Rps4y2	106	225	137	170
Rps5	22416	18912	17394	14325
Rps6	56	180	129	25
Rps6ka1	1048	914	1159	1127
Rps6ka2	869	872	614	1141
Rps6ka3	993	1130	1235	1608
Rps6ka4	1895	2234	1703	1810
Rps6ka5	262	191	167	87
Rps6kb1	918	908	831	1105
Rps6kb2	1014	936	657	832
Rps6kc1	864	755	742	774
Rps6kl1	3	3	16	14
Rps7	42	52	29	44
Rps8	9741	7252	9327	8181
Rptn	343	131	11	0
Rptor	548	746	364	620
Rpusd1	101	161	108	151
Rpusd3	341	401	313	264
Rpusd4	428	627	569	522
Rrad	76	973	283	143
Rraga	2910	2212	2818	2698
Rragc	2812	3062	2797	5157
Rragd	447	307	266	357
Rras	2240	2711	2361	2483
Rras2	1043	1212	1286	847
Rrbp1	6140	12215	8337	15261
Rreb1	1020	651	612	633
Rrm1	2874	2816	3206	2712
Rrm2	297	348	191	104
Rrnad1	101	142	88	20

Rrp1	1324	1739	1160	1057
Rrp12	741	1179	784	737
Rrp15	714	1069	981	869
Rrp1b	917	976	948	775
Rrp36	1034	1315	936	1066
Rrp7a	122	669	162	187
Rrp8	280	270	236	284
Rrp9	609	840	429	415
Rrs1	129	218	235	130
Rsad1	395	409	345	533
Rsad2	119	58	44	122
Rsbn1	176	77	214	149
Rsbn1l	236	196	347	206
Rsf1	458	441	417	326
Rsl1	250	211	221	259
Rsl24d1	1939	1458	1530	1776
Rslcan18	496	490	393	404
Rsph1	49	7	38	20
Rsph10b	0	6	5	0
Rsph3	75	18	74	22
Rsph9	51	33	104	21
Rspo1	223	118	185	438
Rspo3	9	6	6	27
Rspo4	6	19	21	0
Rspry1	272	497	361	462
Rsrc1	652	646	727	809
Rsrc2	897	955	1159	1032
Rsrp1	5197	2737	4521	3421
Rsu1	949	1450	895	1576
RT1-A1	1223	1279	818	358
RT1-A2	115	247	57	190
RT1-Ba	4608	4046	3537	4883
RT1-Bb	31587	16889	30308	29233
RT1-CE10	588	485	361	435
RT1-CE2	0	1	2	1
RT1-CE3	1064	615	364	718
RT1-CE7	83	149	41	70
RT1-Da	25071	19137	23611	34494
RT1-Db1	4625	3162	2107	2774
RT1-DOa	403	240	298	292
RT1-DOb	89	34	47	99
RT1-M10-1	102	15	93	19
RT1-M2	209	0	26	53
RT1-M3-1	286	235	334	378
RT1-M5	65	0	20	3
RT1-M6-1	7	12	17	7
RT1-M6-2	97	48	47	39

RT1-N2	495	620	515	649
RT1-T24-3	159	196	197	283
RT1-T24-4	379	590	361	422
Rtcb	2129	2374	1733	2306
Rtcd1	1625	1619	1681	1529
Rtel1	896	1163	1217	1069
Rtf1	1321	1328	1559	1907
Rtfdc1	2592	2777	2208	2518
Rtn1	259	252	495	406
Rtn2	3121	2841	1419	2310
Rtn3	8229	6677	8266	8109
Rtn4	9974	9088	8015	10593
Rtn4ip1	432	544	384	455
Rtn4r	3	10	9	5
Rtn4rl1	194	608	342	435
Rtn4rl2	25	31	12	14
Rtp3	3	98	39	20
Rtp4	1083	766	800	888
Rttn	174	207	169	44
Rubcn	1180	1433	1186	1370
Rufy1	1141	1345	1115	1435
Rufy3	1354	1070	1192	1508
Rufy4	8	11	17	31
Rundc1	867	583	856	950
Rundc3a	48	81	113	72
Rundc3b	9	0	6	0
Runx1	2157	2037	2961	2105
Runx1t1	9	7	2	14
Runx2	269	259	429	608
Runx3	891	472	770	1182
Rusc1	754	501	615	665
Rusc2	248	636	290	406
Ruvbl1	852	1411	763	796
Ruvbl2	1082	1197	1043	654
Rwdd1	2297	2663	2350	2977
Rwdd2b	672	475	408	463
Rwdd3	118	124	147	135
Rwdd4	892	824	821	828
Rxra	2054	2302	1744	1710
Rxrb	492	709	275	448
Rybp	943	549	624	941
Ryk	2693	2314	2288	2129
Ryr1	7155	6405	4387	5215
Ryr3	52	143	102	45
S100a1	754	410	501	516
S100a10	27282	32281	29712	32995
S100a11	37141	37417	31482	25311

S100a13	1334	1697	1301	1879
S100a16	13728	8275	10589	6135
S100a3	1357	715	1350	229
S100a4	25121	59157	29766	50296
S100a5	14	62	68	49
S100a6	58712	78864	61839	50703
S100a7a	34	38	26	0
S100a8	12781	3543	2597	3932
S100a9	14895	4510	2161	2584
S100b	511	490	597	410
S100g	14	0	17	0
S100pbp	381	315	278	535
S1pr1	432	840	811	762
S1pr2	1678	1549	1360	2156
S1pr3	860	1429	1273	1297
S1pr4	83	46	102	192
S1pr5	1308	292	1241	291
Saal1	65	156	44	60
Sac3d1	246	464	337	349
Sacm1l	1591	1362	1532	1537
Sacs	169	471	310	514
Sae1	2815	3267	3038	3030
Safb	855	1100	799	743
Safb2	478	789	469	465
Sall2	230	182	302	298
Samd1	1843	1674	1748	1574
Samd10	129	77	105	63
Samd11	50	38	53	27
Samd15	12	0	50	13
Samd3	46	9	32	13
Samd4a	63	117	101	176
Samd4b	13	103	38	57
Samd5	104	26	107	76
Samd8	1176	968	1351	1035
Samhd1	164	77	110	198
Samm50	2236	2448	1866	2097
Samsn1	171	240	256	267
Sap130	1039	849	1031	1019
Sap18	528	427	498	373
Sap25	118	201	90	168
Sap30	338	594	317	364
Sap30bp	1262	1282	1151	1042
Sap30l	1214	1415	1323	1429
Sapcd1	484	385	487	662
Sapcd2	244	347	214	241
Sar1a	7322	8976	8006	9792
Sar1b	2573	1628	1345	3267

Saraf	4069	3022	2668	4044
Sardh	778	821	764	613
Sarm1	19	46	75	57
Sarnp	2410	2528	2380	2950
Sars	3389	3256	3098	2874
Sars2	237	268	181	251
Sart1	1903	1995	1793	1570
Sart3	502	695	451	403
Sash1	80	42	11	65
Sash3	558	455	679	1283
Sat1	5255	4247	6527	9048
Sat2	562	457	647	697
Satb1	254	147	175	151
Satb2	16	50	30	41
Satl1	7	0	3	13
Sav1	1084	1960	1849	2017
Saysd1	137	191	169	254
Sbds	1901	1885	2308	1812
Sbf1	608	1077	676	695
Sbf2	319	508	438	569
Sbk2	17	33	8	0
Sbno1	526	300	456	536
Sbno2	717	1201	752	876
Sbsn	8797	3682	4150	1830
Sbspon	162	17	101	182
Sc5d	1143	760	1026	1003
Scaf1	1134	1550	1016	835
Scaf11	1528	1400	1754	1478
Scaf4	497	503	676	457
Scaf8	630	759	954	749
Scai	31	15	32	117
Scamp1	1383	1721	1778	2379
Scamp2	2342	2925	2570	3381
Scamp5	8	62	42	48
Scand1	55	104	42	34
Scap	2499	2504	2374	1834
Scaper	559	230	319	317
Scara3	1331	1839	1398	1042
Scara5	1012	2376	1124	581
Scarb1	1683	2730	1923	3265
Scarb2	8327	5120	6938	7317
Scarf1	193	364	468	498
Scarf2	497	843	573	1236
Scd	28687	11785	15816	15355
Scd2	10284	6786	8621	11250
Scel	770	305	427	273
Scfd1	1831	1581	1697	2259

Scfd2	290	425	325	403
Scg3	6	34	9	0
Scg5	44	26	68	29
Scgb1a1	47	54	65	25
Schip1	1663	1606	1059	2348
Scimp	1029	590	1396	3320
Scin	923	246	405	255
Sclt1	521	249	439	449
Scly	379	442	331	469
Scmh1	449	506	546	477
Scml2	21	0	14	41
Scn1b	3284	2773	1620	2356
Scn2a	8	0	15	0
Scn2b	24	7	5	15
Scn3a	87	75	57	156
Scn3b	89	130	125	40
Scn4a	934	1083	948	701
Scn4b	1219	641	492	1038
Scn5a	32	197	293	19
Scn7a	103	39	54	75
Scnm1	855	918	904	769
Scnn1a	285	211	232	199
Scnn1b	135	33	45	21
Scnn1g	59	37	71	16
Sco1	457	383	503	541
Scoc	1258	1089	1005	1409
Scp2	9709	6774	7485	6615
Scpep1	7058	11525	9474	19162
Scrib	1060	1761	1118	491
Scrn1	208	110	222	214
Scrn2	102	203	66	50
Scrn3	314	235	194	381
Sct	9	19	39	3
Sctr	48	21	90	533
Scube2	17	31	14	26
Scube3	48	12	120	70
Scx	523	573	474	76
Scyl1	3469	3317	3379	2587
Scyl2	915	632	767	828
Scyl3	485	323	346	345
Sdad1	355	434	349	450
Sdc1	47120	20131	44082	21365
Sdc2	4962	4349	4763	5476
Sdc3	628	957	838	884
Sdc4	7884	5284	6595	5050
Sdcbp	5926	5684	5750	10950
Sdcbp2	245	85	41	188

Sdccag3	275	536	247	391
Sdccag8	322	367	274	273
Sde2	1307	919	1134	1135
Sdf2	3894	2906	3776	3635
Sdf2l1	1065	1171	1159	1614
Sdf4	6306	6675	7408	9753
Sdha	6393	5032	4857	6091
Sdhaf2	2901	2317	2618	2803
Sdhaf3	228	136	156	153
Sdhaf4	557	631	367	617
Sdhb	4594	4694	3713	4809
Sdhc	7265	7027	5383	7265
Sdhd	9792	6141	5113	8115
Sdk1	229	433	340	366
Sdk2	390	421	474	278
Sdpr	284	664	418	252
Sdr16c5	744	184	257	51
Sdr16c6	1490	495	569	1132
Sdr39u1	1207	590	822	814
Sdr42e1	161	81	104	61
Sdr9c7	117	55	35	19
Sds	179	39	142	0
Sdsl	12	12	3	0
Sec1	44	822	191	63
Sec11a	2796	3752	2856	4209
Sec11c	4582	2281	3849	2478
Sec13	1853	3042	2404	2944
Sec14l1	897	1768	1235	1103
Sec14l2	855	863	679	593
Sec14l4	14	12	2	0
Sec14l5	53	51	0	16
Sec16a	1894	2337	2084	2391
Sec16b	119	324	254	343
Sec22a	757	769	651	924
Sec22b	2769	2822	2789	3881
Sec22c	486	536	623	465
Sec23a	710	1321	906	1160
Sec23b	597	943	900	842
Sec23ip	985	1008	1482	1372
Sec24a	39	69	80	147
Sec24b	661	873	877	1024
Sec24c	2819	2765	3018	2658
Sec24d	404	1785	922	1673
Sec31a	1247	2611	1718	2327
Sec31b	157	56	39	122
Sec61a1	3729	6959	4448	6504
Sec61a2	857	784	1212	659

Sec61b	3357	3178	2905	4218
Sec61g	625	732	1007	817
Sec62	5724	3745	4664	5631
Sec63	2240	2382	2618	2962
Secisbp2	971	885	787	558
Secisbp2l	1094	658	828	956
Sectm1a	1399	221	611	193
Sectm1b	1291	485	900	226
Seh1l	239	324	278	227
Sel1l	1519	1911	1768	2793
Sel1l3	491	926	561	401
Sele	248	715	679	343
Seli	2209	932	1232	1708
Selk	2264	1923	2155	1919
Sell	265	254	274	366
Selm	1929	3995	3343	4819
Selo	1086	1067	994	1037
Selp	317	744	528	367
Selplg	20	229	7	46
Selt	3966	3920	4037	3885
Sema3a	131	122	113	240
Sema3c	1321	1316	859	1439
Sema3d	7	28	26	0
Sema3e	1435	442	1056	403
Sema3f	563	1005	531	544
Sema3g	489	816	910	809
Sema4a	2281	1084	1836	1299
Sema4b	1138	769	835	497
Sema4c	273	428	375	318
Sema4d	1036	694	516	806
Sema4f	142	66	77	196
Sema4g	81	75	69	12
Sema5a	1640	915	1446	1182
Sema6b	186	302	226	283
Sema6c	415	545	414	340
Sema6d	143	356	348	373
Sema7a	272	876	316	567
Senp1	11	13	3	0
Senp2	599	561	749	750
Senp3	1825	1687	1672	1855
Senp5	13	26	18	7
Senp6	1737	2087	2293	2070
Senp8	160	135	172	142
Sephs1	993	1337	1243	1125
Sephs2	850	1154	1059	1567
Sepn1	3457	6211	4762	8529
Sepp1	16191	16063	15436	16142

Sepsecs	476	507	641	510
Serac1	313	176	176	236
Serbp1	6182	6762	6948	5418
Serf1	398	758	623	747
Serf2	14706	20947	12544	16304
Sergef	347	443	364	387
Serhl2	1711	1338	1321	1531
Serinc1	1985	2537	2765	3052
Serinc2	2207	1186	1527	849
Serinc3	3681	3835	4351	5268
Serinc4	15	36	22	19
Serinc5	824	1224	1193	1068
Serp1	3015	2943	3262	5124
Serp2	20	44	15	56
Serpina1	1241	117	1262	146
Serpina11	472	147	417	118
Serpina12	67	37	60	8
Serpina9	96	29	102	35
Serpinb10	3352	1234	1494	685
Serpinb11	260	381	57	142
Serpinb12	83	30	9	19
Serpinb2	2009	1080	1685	543
Serpinb3	1169	331	98	59
Serpinb5	16858	7260	11281	4299
Serpinb6	8201	11518	8757	10901
Serpinb6b	147	56	116	110
Serpinb7	13	29	8	29
Serpinb8	1245	911	1145	1348
Serpinb9	842	980	767	1188
Serpine1	4851	9321	5212	4658
Serpine2	2270	3424	4019	7155
Serpinf1	13790	22546	18198	26739
Serpinf2	19	32	20	53
Serping1	6860	7585	8729	8988
Serpinh1	18447	41557	25915	57296
Serpini1	15	17	15	62
Sert1	71	56	48	7
Sertad1	2167	1577	1831	1230
Sertad2	1172	1056	1153	1165
Sertad3	293	312	336	285
Sertad4	934	860	1192	1085
Sesn1	777	683	551	676
Sesn2	673	679	724	735
Sesn3	1281	595	1503	1087
Sestd1	759	1110	993	1283
Set	1606	2149	1966	1645
Setbp1	223	118	128	86

Setd2	981	1252	1345	1537
Setd3	2329	2391	2123	2183
Setd4	545	494	442	533
Setd5	1353	1273	1560	1106
Setd6	448	228	289	208
Setd7	3489	3426	4225	4390
Setdb1	2819	2491	2753	2414
Setdb2	196	197	223	188
Setmar	149	145	188	128
Setx	1075	888	1306	979
Sez6	4	23	57	37
Sez6l	18	0	5	0
Sez6l2	50	86	60	38
Sf1	4126	2982	3399	3343
Sf3a1	1251	1574	1447	1129
Sf3a2	431	784	451	352
Sf3a3	1307	1720	1648	1526
Sf3b1	6123	5346	7109	7189
Sf3b2	4341	5241	5257	5531
Sf3b3	2140	2814	2303	2250
Sf3b4	1887	2166	1581	1363
Sf3b5	1477	2079	1244	1198
Sf3b6	1405	1318	1300	977
Sfi1	382	368	292	325
Sfmbt1	491	410	592	430
Sfmbt2	15	107	69	37
Sfn	15574	12257	11143	3324
Sfpq	5137	4062	5868	4965
Sfr1	3007	2730	3730	4333
Sfrp1	2739	2419	3551	2186
Sfrp2	484	2805	3160	10153
Sfrp4	6895	23939	6759	7336
Sfrp5	99	45	89	80
Sfswap	384	616	427	346
Sft2d1	902	654	713	744
Sft2d2	1436	1076	1608	780
Sftpc	189	95	169	76
Sfxn1	223	505	340	522
Sfxn3	4698	4271	4011	5240
Sfxn4	184	174	113	127
Sgca	901	1381	594	716
Sgcb	2106	1427	1542	2385
Sgcd	379	399	217	259
Sgce	414	655	584	561
Sgcg	2190	2522	2093	4773
Sgf29	690	734	455	626
Sgip1	40	91	69	62

Sgk1	3791	1954	2531	4399
Sgk3	931	841	937	779
Sgms1	834	801	737	977
Sgms2	165	196	190	311
Sgo1	78	80	146	30
Sgo2	116	256	310	159
Sgpl1	2381	2024	2156	4963
Sgpp1	2617	1569	2386	1612
Sgpp2	100	39	35	24
Sgsh	106	147	126	223
Sgsm1	10	8	9	5
Sgsm2	364	360	420	265
Sgsm3	1130	974	915	1078
Sgta	2146	2831	1757	1997
Sgtb	370	193	190	214
Sh2b1	1128	1500	973	885
Sh2b2	283	468	250	345
Sh2b3	1027	1579	933	2169
Sh2d1a	81	35	57	133
Sh2d1b	2	5	0	60
Sh2d2a	96	118	120	89
Sh2d3c	252	686	481	423
Sh2d4a	409	165	277	202
Sh3bgr	11434	4279	1939	6409
Sh3bgrl	827	730	1044	1536
Sh3bgrl3	8334	9816	8383	10768
Sh3bp1	876	578	763	408
Sh3bp2	912	1000	766	922
Sh3bp4	385	598	414	527
Sh3bp5l	924	1057	988	893
Sh3d19	1319	893	1100	985
Sh3d21	787	363	671	512
Sh3gl1	300	837	394	622
Sh3gl2	11	53	30	34
Sh3gl3	45	26	26	25
Sh3glb1	6553	8036	7959	11255
Sh3glb2	200	226	125	200
Sh3kbp1	1255	1137	1217	2169
Sh3pxd2a	2614	4357	3382	3040
Sh3pxd2b	2025	3680	2931	5442
Sh3rf1	851	567	942	659
Sh3rf2	694	403	403	159
Sh3rf3	60	135	63	179
Sh3tc1	154	401	287	548
Sh3tc2	449	69	352	147
Sh3yl1	497	164	308	354
Shank3	279	537	487	222

Sharpin	1412	1546	1577	1638
Shb	917	781	831	480
Shbg	116	74	128	100
Shc1	2568	3952	3521	4503
Shc2	405	709	490	1085
Shc3	0	2	0	1
Shcbp1	224	307	253	382
Shd	26	40	27	18
She	224	553	478	494
Shf	241	313	253	261
Shfm1	7062	8115	6883	6313
Shisa2	3054	691	2391	1278
Shisa4	1588	1173	704	1375
Shisa5	5896	5669	6095	6170
Shisa8	63	13	74	14
Shkbp1	430	405	460	387
Shmt1	1279	1370	1318	1429
Shmt2	933	2083	1049	1007
Shoc2	89	174	191	111
Shox2	114	128	184	348
Shpk	224	106	185	167
Shprh	287	222	420	263
Shq1	87	103	83	155
Shroom1	0	8	5	7
Shroom2	296	51	224	133
Shroom3	178	140	162	71
Shtn1	899	420	1095	609
Siae	154	147	316	383
Siah1	478	539	653	559
Siah2	800	529	599	573
Sidt2	3505	3224	3412	4622
Sigirr	145	258	232	271
Siglec1	444	815	534	1334
Siglec10	377	506	623	779
Siglec15	30	56	122	227
Siglec5	208	283	342	425
Siglec8	396	368	342	727
Siglech	6	31	3	13
Sigmar1	2170	2232	1897	2113
Sik1	22	38	29	23
Sik2	13	119	144	141
Sik3	999	1460	1038	1614
Sike1	1643	1351	1623	1565
Sil1	426	1119	773	758
Sim1	9	19	12	0
Sim2	35	18	57	37
Simc1	31	93	57	67

Sin3a	1104	1180	1464	1175
Sin3b	1834	2343	2291	2721
Sipa1	519	1245	442	942
Sipa1l1	442	375	569	454
Sipa1l2	244	346	414	293
Sipa1l3	511	421	372	253
Sirpa	3659	3767	5171	5781
Sirpd	64	20	27	23
Sirt1	268	263	292	134
Sirt2	2192	3482	2376	2663
Sirt3	409	415	253	430
Sirt4	144	137	56	60
Sirt5	1091	765	1092	715
Sirt6	288	316	262	195
Sit1	58	24	42	49
Siva1	558	749	438	440
Six1	650	513	323	388
Six4	160	292	153	142
Six5	424	533	593	589
Ska2	520	743	528	638
Ska3	447	519	386	301
Skap1	123	43	104	45
Skap2	2326	1919	2279	3484
Ski	3656	3201	3111	3399
Skida1	30	48	26	8
Skil	1403	581	1130	1130
Skint1	1316	104	639	163
Skint10	15	0	8	0
Skint4	722	10	330	69
Skint8	297	49	169	92
Skiv2l	277	623	460	458
Skiv2l2	1009	1267	1149	1681
Skp1	17535	11317	12207	14129
Skp2	96	149	120	153
Sla	79	116	86	257
Slain1	21	17	32	63
Slain2	2869	2483	2767	1673
Slamf1	14	24	15	25
Slamf6	157	54	244	851
Slamf7	7152	6317	7240	8056
Slamf8	661	288	612	1406
Slamf9	1582	1561	2197	6694
Slbp	669	723	791	930
Slc10a3	521	1100	677	792
Slc10a5	31	18	32	36
Slc10a6	581	540	531	572
Slc10a7	120	143	211	215

Slc11a1	444	847	680	1834
Slc11a2	655	787	609	772
Slc12a1	19	0	20	0
Slc12a2	1226	908	736	681
Slc12a3	58	20	14	0
Slc12a4	1093	1631	1151	1187
Slc12a6	111	145	125	95
Slc12a7	704	972	713	1049
Slc12a8	31	56	35	34
Slc12a9	304	305	274	477
Slc13a3	38	131	83	55
Slc13a4	207	71	159	107
Slc13a5	10	0	5	19
Slc14a1	59	15	39	88
Slc14a2	8	7	8	0
Slc15a1	323	60	140	62
Slc15a2	64	8	33	43
Slc15a3	351	270	379	1148
Slc15a4	793	800	912	900
Slc16a1	2742	2013	1541	2734
Slc16a11	118	64	59	12
Slc16a12	89	190	128	211
Slc16a13	452	287	396	309
Slc16a14	137	114	108	5
Slc16a2	579	913	665	1158
Slc16a3	1119	783	718	1535
Slc16a4	5	10	15	15
Slc16a6	630	421	431	432
Slc16a7	130	116	214	244
Slc16a8	14	27	0	17
Slc16a9	99	186	114	75
Slc17a5	333	427	346	510
Slc17a9	110	115	108	408
Slc18b1	145	230	162	153
Slc19a1	148	321	138	147
Slc19a2	367	123	181	148
Slc19a3	76	19	15	98
Slc1a1	24	26	9	5
Slc1a3	684	155	620	420
Slc1a4	632	900	602	1011
Slc1a5	1174	1292	1381	2007
Slc20a1	3989	2617	4393	3712
Slc20a2	713	889	712	605
Slc22a1	33	17	23	27
Slc22a15	696	278	635	426
Slc22a17	365	433	406	371
Slc22a18	657	428	629	306

Slc22a2	6	18	15	0
Slc22a23	156	223	129	91
Slc22a3	62	58	39	0
Slc22a4	17	16	30	56
Slc22a5	446	512	481	274
Slc23a1	12	0	5	5
Slc23a2	391	431	533	633
Slc24a1	44	21	36	31
Slc24a3	143	232	138	203
Slc25a1	1378	1400	1178	1209
Slc25a10	458	451	444	528
Slc25a11	6326	5033	5325	5260
Slc25a12	1452	1167	703	1168
Slc25a14	242	162	239	298
Slc25a15	467	516	454	499
Slc25a16	268	266	331	413
Slc25a17	2153	1454	1890	1539
Slc25a18	33	7	17	0
Slc25a19	293	263	117	263
Slc25a2	23	0	35	18
Slc25a20	3113	2317	2690	3052
Slc25a21	17	43	15	33
Slc25a23	491	429	278	254
Slc25a24	1298	1184	973	1593
Slc25a25	1059	440	403	292
Slc25a26	1409	840	1252	833
Slc25a27	226	116	147	101
Slc25a28	1629	1410	1306	1243
Slc25a29	121	209	120	190
Slc25a3	21767	19363	16988	19610
Slc25a30	434	283	420	465
Slc25a32	294	270	193	370
Slc25a33	145	134	95	114
Slc25a34	145	39	62	29
Slc25a35	317	139	241	333
Slc25a36	1304	693	967	1064
Slc25a37	564	518	682	539
Slc25a38	488	523	420	449
Slc25a39	2271	2706	2329	2713
Slc25a4	40261	30765	21553	35668
Slc25a40	55	37	62	2
Slc25a42	321	374	217	303
Slc25a43	141	105	179	197
Slc25a44	973	638	948	1169
Slc25a45	126	150	149	161
Slc25a46	1582	1432	1303	1417
Slc25a47	0	11	12	11

Slc25a48	792	391	245	80
Slc25a5	5060	5088	3814	3976
Slc25a6	243	333	172	383
Slc26a1	17	44	15	4
Slc26a11	157	197	212	383
Slc26a2	24	24	51	12
Slc26a4	81	0	6	0
Slc26a6	538	544	343	520
Slc26a7	207	156	113	24
Slc26a8	193	110	193	142
Slc26a9	3	24	21	6
Slc27a1	936	874	897	806
Slc27a2	51	35	23	13
Slc27a3	875	599	692	421
Slc27a4	1609	1539	1103	1276
Slc27a6	0	14	2	0
Slc28a1	12	11	9	0
Slc28a2	1306	3232	2162	6814
Slc29a1	1034	1509	1601	501
Slc29a3	2042	1818	2248	3218
Slc29a4	24	64	27	7
Slc2a1	2921	3301	2976	2168
Slc2a10	142	240	217	483
Slc2a12	43	57	2	23
Slc2a13	79	200	78	136
Slc2a4	2325	1683	1263	3440
Slc2a5	136	104	68	159
Slc2a6	693	855	893	1266
Slc2a8	973	560	873	599
Slc2a9	459	606	445	500
Slc30a1	1049	990	1398	973
Slc30a2	124	177	271	54
Slc30a3	70	57	66	30
Slc30a4	1076	1234	1875	1661
Slc30a5	1159	967	1198	1510
Slc30a6	550	638	415	567
Slc30a7	317	386	326	579
Slc30a9	1549	1244	1509	1340
Slc31a1	3395	2535	3649	4723
Slc31a2	1933	1381	1342	2284
Slc33a1	664	616	651	842
Slc34a1	0	15	2	0
Slc34a2	69	13	15	39
Slc35a1	864	721	826	627
Slc35a2	114	187	54	193
Slc35a3	209	81	193	161
Slc35a4	2627	2921	2255	2733

Slc35a5	308	264	402	466
Slc35b1	2629	3059	2284	3594
Slc35b2	3828	4087	3505	3017
Slc35b3	473	344	536	383
Slc35b4	1644	1923	1727	2358
Slc35c1	858	805	942	1405
Slc35c2	437	676	475	743
Slc35d1	250	361	326	265
Slc35d2	264	243	233	334
Slc35e1	1613	1855	1891	2016
Slc35e2b	436	222	342	444
Slc35e3	501	591	566	940
Slc35e4	1239	1316	1059	1366
Slc35f1	8	14	2	4
Slc35f2	63	23	51	26
Slc35f5	399	847	632	924
Slc35f6	874	1383	1098	1649
Slc35g1	5	19	5	0
Slc36a1	40	10	20	51
Slc36a4	618	695	790	1012
Slc37a1	97	84	75	71
Slc37a2	464	1227	802	3192
Slc37a3	1897	1509	1736	1313
Slc37a4	1836	1092	1122	1486
Slc38a10	2496	4214	3023	5777
Slc38a2	9536	5279	8031	6054
Slc38a3	300	139	83	133
Slc38a4	1076	610	933	1219
Slc38a5	268	41	305	59
Slc38a6	404	539	406	757
Slc38a7	942	1360	852	1483
Slc38a8	12	1	3	16
Slc38a9	475	367	382	476
Slc39a1	5747	6291	7574	7898
Slc39a10	763	498	626	550
Slc39a11	927	704	806	1011
Slc39a13	950	2310	1464	1523
Slc39a14	2288	4419	3670	4649
Slc39a3	326	445	271	377
Slc39a6	3164	2114	4211	2458
Slc39a7	3721	5163	4485	5547
Slc39a8	1322	582	906	927
Slc39a9	996	1112	1112	1593
Slc3a2	7799	6125	9422	6181
Slc41a1	1274	1012	1515	1290
Slc41a2	445	771	612	1344
Slc41a3	221	493	181	404

Slc43a1	401	407	367	624
Slc43a2	3138	4513	3582	4579
Slc43a3	228	280	199	186
Slc44a1	5857	4675	6513	4880
Slc44a2	4762	4840	6093	5317
Slc44a3	37	39	35	34
Slc44a4	100	34	12	17
Slc45a3	298	156	247	187
Slc45a4	299	534	349	503
Slc46a1	387	544	495	435
Slc46a2	40	7	51	0
Slc46a3	128	146	193	281
Slc47a1	268	450	256	356
Slc48a1	3640	6451	3502	5313
Slc4a1	9	0	33	0
Slc4a11	97	37	75	21
Slc4a1ap	4514	3471	2853	3310
Slc4a2	1111	1484	1350	1886
Slc4a3	121	595	304	60
Slc4a4	123	155	108	112
Slc4a7	141	391	172	143
Slc4a8	5	10	14	24
Slc4a9	255	92	132	62
Slc50a1	1080	905	964	1364
Slc51a	127	483	232	182
Slc52a2	94	152	87	129
Slc52a3	267	218	135	82
Slc5a1	6	0	12	0
Slc5a10	2665	1215	2390	770
Slc5a11	6	0	18	0
Slc5a2	24	97	11	0
Slc5a3	31	37	20	0
Slc5a5	239	147	92	186
Slc5a6	1652	1170	1017	1175
Slc5a8	103	107	47	38
Slc5a9	465	64	363	148
Slc6a12	35	57	215	751
Slc6a13	10	0	2	0
Slc6a15	13	0	17	21
Slc6a17	45	119	45	54
Slc6a19	15	6	3	8
Slc6a2	104	67	81	33
Slc6a20	2	9	5	0
Slc6a4	1009	427	635	374
Slc6a6	2150	3042	3703	4130
Slc6a8	4193	2584	2931	2759
Slc6a9	93	303	263	307

Slc7a1	1074	1272	1130	1113
Slc7a10	200	127	116	72
Slc7a11	581	304	528	509
Slc7a15	22	0	32	3
Slc7a2	69	74	156	308
Slc7a3	9	0	9	0
Slc7a4	48	23	41	11
Slc7a5	2689	2374	2722	2722
Slc7a6	2003	1401	1660	2287
Slc7a6os	1341	982	1222	974
Slc7a7	408	824	662	926
Slc7a8	4112	2324	5422	2608
Slc8a1	475	816	697	920
Slc8a3	361	353	134	250
Slc8b1	655	951	659	591
Slc9a1	744	831	650	806
Slc9a2	35	15	83	36
Slc9a3	144	157	203	128
Slc9a3r1	1330	1023	1365	1927
Slc9a3r2	753	2088	1008	1041
Slc9a5	36	93	78	66
Slc9a6	649	290	418	629
Slc9a7	65	11	45	172
Slc9a8	428	586	436	578
Slc9a9	348	635	462	414
Slc9b2	90	81	96	159
Slco1a5	27	0	15	0
Slco2a1	885	1341	1109	569
Slco2b1	1172	1336	1566	969
Slco3a1	898	992	898	685
Slco4a1	170	124	248	223
Slco5a1	302	239	349	172
Slf1	90	135	131	104
Slf2	163	104	149	179
Slfn1	289	258	184	196
Slfn13	1530	1556	2004	2095
Slfn2	3226	4269	4076	5402
Slfn3	154	88	179	167
Slfn4	4188	2543	2319	2939
Slfn5	1847	2983	2883	3520
Slfnl1	5	9	30	26
Slirp	3648	2489	2558	3021
Slit2	391	390	518	673
Slit3	485	1141	575	445
Slitrk4	22	28	8	0
Slitrk6	63	69	50	22
Slk	1420	2355	1774	1817

Slmap	411	568	527	619
Sln	3167	9947	7151	1592
Sltm	1154	1466	1270	1066
Slu7	1206	1284	934	1224
Slx1b	768	683	719	554
Slx4	302	360	266	312
Slx4ip	111	107	96	156
Smad1	871	948	1205	987
Smad2	980	919	1190	1451
Smad3	985	1405	1181	977
Smad4	1716	1597	1766	1770
Smad5	1134	1497	1440	1327
Smad6	29	94	26	63
Smad7	238	325	137	174
Smagp	1675	1374	1468	1864
Smap1	1742	1470	1462	2011
Smap2	1968	2547	2195	3325
Smarca1	7	3	30	34
Smarca2	1243	821	1241	1056
Smarca4	1647	2591	2143	1919
Smarca5	704	609	963	897
Smarcad1	259	395	269	663
Smarcal1	740	743	900	670
Smarcc1	895	1283	1205	1258
Smarcc2	2018	1916	2213	1800
Smarcd1	584	805	698	649
Smarcd2	560	1372	854	835
Smarcd3	1447	1798	1161	941
Smarce1	3074	2862	3600	3633
Smc1a	3522	3154	3810	3280
Smc2	210	380	308	277
Smc3	931	1314	1631	1090
Smc4	1302	1249	1544	1223
Smc5	397	381	629	492
Smc6	1281	1257	1326	1281
Smchd1	510	664	528	503
Smco4	824	696	656	514
Smcr8	35	67	39	4
Smdt1	3783	4078	2564	2886
Smg1	1236	805	1309	1152
Smg5	1191	1390	1038	930
Smg7	61	413	113	2
Smg8	232	338	262	186
Smg9	330	654	420	435
Smim1	311	240	351	382
Smim10l1	0	1	5	0
Smim11	1349	1383	1592	1097

Smim12	570	515	396	515
Smim13	51	73	29	52
Smim14	4191	3114	3691	5235
Smim15	1058	969	987	1232
Smim19	2072	1410	1739	2002
Smim20	1546	1572	973	1144
Smim22	136	57	81	20
Smim24	119	126	71	180
Smim3	617	713	892	1142
Smim4	467	579	531	377
Smim5	143	87	159	74
Smim7	2581	3840	2735	3272
Smim8	686	418	254	447
Smn1	1278	1678	1271	1349
Smndc1	201	263	226	193
Smo	34	143	62	69
Smoc1	2015	291	2543	433
Smoc2	2042	1152	2006	1316
Smox	1100	647	1026	676
Smpd1	2788	3352	2564	3306
Smpd2	419	528	311	319
Smpd4	546	519	497	511
Smpd5	182	288	184	279
Smpdl3a	4846	3745	4729	15716
Smpdl3b	1277	715	1204	927
Smpx	5227	3602	3184	2682
Sms	632	390	478	744
Smtn	445	656	841	730
Smtnl1	1643	602	503	663
Smtnl2	894	665	682	779
Smu1	2110	2197	2213	2527
Smug1	1032	562	674	372
Smurf1	694	798	674	878
Smurf2	655	708	831	660
Smyd1	3044	2295	1333	2620
Smyd2	2395	2274	1425	2428
Smyd3	235	121	197	299
Smyd4	65	75	111	119
Smyd5	445	683	447	567
Snai1	288	825	447	946
Snai2	958	630	1026	722
Snai3	88	84	53	149
Snap23	2056	1949	2308	3031
Snap29	1320	1880	1739	2288
Snap47	380	434	248	487
Snap91	23	20	26	45
Snapc1	122	133	212	300

Snapc2	537	428	530	491
Snapc3	1802	1431	1731	1466
Snapc4	129	168	95	73
Snapc5	919	646	900	834
Snapin	2861	2152	2094	2627
Snca	6	29	42	94
Sncaip	125	201	215	546
Sncg	610	631	614	290
Snd1	4143	5316	4994	5257
Sned1	1672	1992	1777	1312
Snf8	3475	3621	3969	3793
Snn	1492	926	1531	1300
Snph	10	27	45	22
Snrk	958	1001	1032	963
Snrnp200	2571	3332	2631	3158
Snrnp25	1348	1168	1252	1125
Snrnp27	1371	1246	1205	1119
Snrnp35	527	824	533	576
Snrnp40	1222	1192	1094	991
Snrnp48	399	574	417	503
Snrnp70	2906	3753	3318	2436
Snrpa	749	1092	659	665
Snrpa1	1152	1345	1219	931
Snrpb	3480	3995	3575	3210
Snrpb2	1968	2117	2561	1970
Snrpc	69	86	146	182
Snrpd1	757	752	961	599
Snrpd2	1652	1692	1672	1956
Snrpd2l	1191	1407	1174	1595
Snrpd3	3242	4042	3850	3498
Snrpe	87	96	77	59
Snrpf	2089	2181	2719	2168
Snrpg	26	88	38	20
Snrpn	203	134	95	271
Snta1	1939	3165	1456	1887
Sntb1	458	233	328	184
Sntn	14	19	0	10
Snupn	498	473	481	384
Snw1	2217	2427	3159	2145
Snx1	152	602	237	300
Snx10	746	968	793	1861
Snx11	690	705	579	685
Snx12	2475	2457	2809	2969
Snx13	601	732	564	660
Snx14	474	482	573	756
Snx15	482	596	403	339
Snx16	82	92	122	105

Snx17	2527	2614	2183	2223
Snx18	1761	1014	1318	1960
Snx19	699	742	834	715
Snx2	2369	2862	2738	3231
Snx20	342	269	286	547
Snx21	883	630	640	617
Snx24	272	309	524	580
Snx25	455	311	519	366
Snx27	1030	1080	1280	1415
Snx29	396	392	536	732
Snx3	9014	9140	9048	10885
Snx30	160	287	323	478
Snx32	2709	3261	3640	3652
Snx33	1127	1457	1316	1103
Snx4	992	947	1183	1259
Snx5	4901	4138	5146	6676
Snx6	1524	1624	1805	2490
Snx7	123	460	384	408
Snx8	591	1035	742	1337
Snx9	1020	2035	1553	1965
Soat1	7607	4271	4709	10183
Sobp	385	349	152	240
Socs1	279	120	169	42
Socs2	665	597	564	473
Socs3	2399	2838	2249	3000
Socs4	69	44	36	74
Socs5	1043	1108	877	997
Socs6	348	374	500	415
Socs7	661	640	921	601
Sod1	11631	8046	9137	8591
Sod2	5851	7032	5928	10184
Sod3	2175	3400	1765	880
Soga1	9	39	11	2
Soga3	350	160	110	116
Sohlh2	0	126	17	20
Son	2571	2509	2991	2659
Sorbs1	710	1259	879	757
Sorbs2	233	296	278	374
Sorbs3	987	1519	1127	1438
Sorcs1	14	9	8	20
Sorcs2	2059	1675	2302	2366
Sord	748	1152	1146	1373
Sort1	1132	1472	1091	1122
Sos1	632	565	703	524
Sos2	1626	1315	1041	1121
Sostdc1	1404	391	734	332
Sowahb	19	47	21	38

Sowahc	410	294	226	377
Sox10	45	79	68	34
Sox12	313	328	325	448
Sox13	644	594	1130	557
Sox15	667	802	484	193
Sox17	102	245	262	146
Sox18	218	994	399	585
Sox21	238	49	119	0
Sox4	1268	591	1292	716
Sox5	10	37	29	19
Sox6	27	26	35	68
Sox7	200	334	283	248
Sox8	5	37	3	3
Sox9	2739	952	1983	717
Sp1	3319	2133	2570	2611
Sp100	1633	1870	2255	3138
Sp110	1192	1378	1455	1836
Sp140	501	872	680	1304
Sp2	574	735	533	667
Sp3	60	164	65	93
Sp5	5	0	2	0
Sp6	417	107	314	119
Sp7	0	21	6	29
Spa17	184	259	178	228
Spag1	105	49	47	86
Spag5	620	868	585	640
Spag8	35	15	28	30
Spag9	2222	2226	2805	3280
Sparc	104807	247883	194850	401064
Sparcl1	2974	5029	5550	4168
Spast	1891	1324	1587	1793
Spata1	9	32	60	14
Spata13	399	366	534	736
Spata17	0	16	17	4
Spata2	565	465	524	677
Spata20	35	3	9	19
Spata22	3	14	0	5
Spata25	0	0	51	67
Spata2L	70	21	61	33
Spata31d1(	0	11	0	11
Spata33	107	103	99	76
Spata5	395	345	266	377
Spata5l1	261	220	247	173
Spata6	169	310	209	236
Spata6l	267	78	140	108
Spata7	408	391	454	329
Spata9	7	42	43	50

Spats2	626	851	814	875
Spats2l	467	713	477	628
Spc24	163	347	242	123
Spc25	322	274	260	258
Spcs1	4854	3952	3557	5037
Spcs2	3384	3153	2890	3787
Spcs3	2412	1933	2064	2748
Spdef	13	24	26	18
Spdl1	197	281	259	143
Spdya	28	0	9	0
Specc1	817	901	1216	1372
Specc1l	944	1092	855	838
Spef1	362	376	330	293
Speg	917	912	679	673
Spen	523	554	465	349
Spg11	2582	1798	1894	2494
Spg20	861	1154	1336	1717
Spg21	2913	4091	3823	3932
Spg7	749	854	731	880
Sphk1	580	711	481	879
Sphk2	172	276	250	341
Sphkap	14	12	14	21
Spi1	702	1002	754	1475
Spic	143	108	111	99
Spidr	77	114	149	143
Spin1	17	29	18	0
Spin4	5	20	2	0
Spink4	26	16	14	0
Spink8	44	120	105	84
Spint1	816	614	742	320
Spint2	4870	3116	4129	2228
Spire1	691	957	849	995
Spire2	29	21	29	0
Spn	507	561	823	2258
Spns1	615	841	656	555
Spns2	89	147	120	188
Spns3	37	29	12	21
Spo11	0	5	5	7
Spock2	50	52	44	7
Spon1	2260	2181	2458	4681
Spon2	1068	928	861	327
Spop	2366	2096	2457	2324
Spopl	436	187	292	455
Spp1	6866	19311	20781	175763
Sppl2a	1205	1060	1001	1374
Sppl2b	463	554	351	316
Sppl3	2208	1520	1742	1603

Spr	568	363	483	461
Spred1	1900	1276	2129	1998
Spred2	11	7	0	6
Spred3	288	438	411	590
Sprn	0	22	24	23
Sprr1b	1278	913	366	158
Sprr4	940	176	820	127
Sprtn	650	625	657	462
Spry1	400	618	540	373
Spry2	497	682	620	369
Spry4	315	389	326	381
Spryd3	719	1070	894	710
Spryd4	868	829	870	846
Spryd7	556	338	581	761
Spsb1	633	1353	566	887
Spsb3	887	1007	668	842
Spsb4	172	116	181	140
Spta1	2	15	8	0
Sptan1	6768	7679	6709	6197
Sptb	2494	1807	2175	1909
Sptbn1	4366	6208	5228	4419
Sptbn2	1146	488	1255	309
Sptbn4	5	24	14	27
Sptlc1	1653	1480	1228	1290
Sptlc2	1696	2037	1977	2351
Sptlc3	104	12	45	44
Sptssa	3753	2061	2558	3631
Sptssb	46	19	23	0
Sqle	4321	1731	2461	3170
Sqrdl	1295	935	1133	880
Sqstm1	8646	8864	8482	9066
Srbd1	466	485	424	557
Src	1948	2092	1691	1938
Srcap	1631	1494	1688	1216
Srd5a1	644	340	430	501
Srd5a3	1085	680	1189	1032
Srebf1	209	528	229	341
Srebf2	3130	2187	2555	1910
Srek1	1285	1047	1056	842
Srek1ip1	11	20	36	76
Srf	2261	2599	2570	1899
Srfbp1	544	901	710	477
Srgap1	375	327	506	237
Srgap2	1144	1553	1649	1820
Srgn	780	912	1529	1116
Sri	2891	2367	2677	4011
Srl	9832	11435	4878	8029

Srp14	4319	2976	2844	3340
Srp54a	1867	1695	1727	1757
Srp9	2038	2004	2495	2156
Srpk1	1789	1845	1840	1810
Srpk2	1948	1347	1327	1577
Srpk3	666	1869	645	390
Srpra	2663	3667	3744	4046
Srprb	2413	2817	2434	4158
Srpx	1278	2461	2051	4875
Srpx2	766	2033	1598	3035
Srr	22	118	43	115
Srrd	385	325	332	264
Srrm1	2057	1649	1565	1543
Srrm2	8373	7011	9198	6463
Srrm3	0	33	0	1
Srrt	1962	2877	2033	1526
Srsf1	5486	4639	5045	4642
Srsf10	1415	831	1240	1428
Srsf11	1578	1214	1411	1103
Srsf2	7351	5192	6922	5116
Srsf3	6780	4082	4866	4704
Srsf4	968	1306	1593	1406
Srsf5	2949	3908	4649	3767
Srsf6	4894	4037	5113	4307
Srsf7	2070	1769	1992	2042
Srsf9	1817	2368	2237	2967
Srxn1	1338	1549	1283	1281
Ss18	3582	3154	4651	4614
Ss18l2	2481	1821	1523	1995
Ssb	1168	1218	1594	1128
Ssbp1	1080	1116	1298	1248
Ssbp2	960	744	876	851
Ssbp3	2449	1900	2249	1619
Ssbp4	352	659	326	269
Ssc4d	197	206	74	321
Ssc5d	493	2551	790	1789
Ssfa2	1395	682	1119	791
Ssh1	35	62	35	78
Ssh2	983	1302	1407	1172
Ssh3	1096	976	890	890
Ssmem1	21	0	29	12
Ssna1	1560	1766	1238	1282
Sspn	1339	1505	979	1022
Ssr1	4044	4600	4491	6944
Ssr2	5196	8035	6251	10319
Ssr3	3358	4167	4308	5373
Ssr4	4373	5581	3483	5752

Ssrp1	4466	5544	5886	5191
Sst	1	10	2	0
Ssu72	2448	2329	2070	2594
Ssx2ip	359	760	418	557
St13	836	1043	734	588
St14	1311	1068	1020	957
St3gal1	1447	1964	1712	2720
St3gal2	1608	1684	1235	1689
St3gal3	481	473	320	495
St3gal4	2481	3212	2999	4513
St3gal5	826	1700	1617	4169
St3gal6	533	287	489	258
St5	1427	1458	1160	1239
St6gal1	1507	1670	2249	1516
St6galnac2	3627	1500	2755	1415
St6galnac3	78	99	87	126
St6galnac4	793	1825	925	1772
St6galnac6	1246	1282	1375	1538
ST7	572	719	513	854
St7l	321	333	391	318
St8sia1	126	51	69	98
St8sia4	137	148	227	378
St8sia5	87	58	26	62
Stab1	1809	2507	2476	1856
Stac	218	154	217	161
Stac2	44	52	74	168
Stac3	1916	2351	1077	1710
Stag1	727	568	846	849
Stag2	1638	1169	1267	943
Stag3	7	6	23	11
Stam	773	700	825	537
Stam2	392	548	542	447
Stambp	755	675	539	576
Stambpl1	654	680	794	636
Stap1	230	149	223	1089
Stap2	1832	636	1056	571
Stard10	1074	451	676	378
Stard13	467	561	641	544
Stard3	1079	1434	955	1240
Stard3nl	550	691	818	1143
Stard4	159	135	149	253
Stard5	1710	818	1230	1141
Stard6	54	69	69	11
Stard7	3845	3324	4220	4450
Stard8	187	528	466	426
Stard9	310	430	328	430
Stat1	4330	2739	3035	3314

Stat2	1510	1541	1395	1666
Stat3	4321	5072	4560	4685
Stat4	220	79	260	105
Stat5a	744	724	642	950
Stat5b	1223	1244	1289	1323
Stat6	3590	3054	3428	4074
Stau1	1115	1650	1335	1495
Stau2	1074	1150	913	989
Stbd1	1238	1874	1259	1312
Stc1	69	229	104	171
Stc2	433	296	454	417
Steap1	325	841	703	995
Steap2	317	590	492	776
Steap3	1178	2170	1249	2118
Steap4	693	960	1134	812
Stfa2	258	551	20	15
Stfa2l2	3931	2095	417	230
Stfa3	31313	11553	4338	2538
Stil	116	258	212	136
Stim1	2844	2320	2179	2404
Stim2	1040	912	975	1161
Stip1	3502	4957	3406	3455
Stk10	589	863	691	1428
Stk11	1077	1282	955	1000
Stk11ip	624	564	629	609
Stk16	774	1014	688	966
Stk17b	1310	1502	1125	1445
Stk19	26	63	26	33
Stk24	2810	2754	2845	3294
Stk25	1668	2111	1811	1906
Stk26	290	20	149	92
Stk3	679	413	450	550
Stk32c	1	20	3	0
Stk35	473	299	424	344
Stk36	42	88	159	127
Stk38	1982	1431	1998	1646
Stk38l	933	1143	1503	1469
Stk39	591	388	554	484
Stk4	1196	941	1198	1465
Stk40	921	970	677	868
Stmn1	4264	3713	3946	3448
Stmn2	903	493	922	819
Stmn4	51	217	75	116
Stom	10040	7570	10552	13880
Stoml1	177	242	226	275
Stoml2	1570	2289	1461	1427
Ston1	465	382	688	771

Ston2	19	15	51	10
Stox2	247	192	296	214
Stpg3	1	6	5	0
Stra13	613	910	624	588
Stra6	668	121	367	143
Strada	890	1080	886	1118
Stradb	908	455	424	614
Strap	3251	3105	2938	3056
Strbp	589	364	602	428
Strip1	1951	1806	1930	1521
Strip2	158	129	110	196
Strn	1262	1026	1350	817
Strn3	665	564	760	520
Strn4	1672	1298	1294	1129
Sts	248	300	221	259
Stt3a	3985	5298	5356	6800
Stt3b	2434	2397	2215	3097
Stub1	3095	3456	3110	2247
Stum	13	5	42	33
Stx11	131	99	99	201
Stx12	3327	2583	3369	3084
Stx16	416	406	438	473
Stx17	567	363	363	571
Stx18	770	1109	941	1016
Stx1a	22	33	35	39
Stx1b	26	20	32	0
Stx2	280	862	549	568
Stx3	9	0	11	0
Stx4	2516	2489	1848	1865
Stx5	2632	2824	1974	2853
Stx6	2340	1585	2430	2269
Stx7	12	55	24	77
Stx8	1266	1134	1241	1234
Stxbp1	451	1228	642	900
Stxbp2	1359	1172	1354	1192
Stxbp3	1452	1015	1673	1536
Stxbp4	273	233	236	176
Stxbp5	1267	1205	1595	1729
Stxbp6	72	134	72	112
Styx	395	364	298	543
Styxl1	69	42	48	77
Styxl2	16	15	41	25
Sub1	3715	2268	3417	2709
Sucla2	3604	2254	2768	3345
Suclg1	7353	6989	5981	7477
Suclg2	3118	2328	3024	2989
Suco	978	677	907	641

Suds3	1506	1612	1405	1367
Sufu	756	626	590	620
Sugct	66	25	29	23
Sugp1	949	888	1017	779
Sugp2	608	665	597	437
Sugt1	4304	2491	3027	2976
Sulf1	764	1239	1080	1544
Sulf2	3160	4028	3259	5335
Sult1a1	761	816	595	487
Sult1d1	280	106	262	59
Sult2b1	361	308	250	129
Sult5a1	1553	334	903	823
Sumf1	1061	1055	1273	1529
Sumf2	143	494	343	291
Sumo1	5647	3570	4061	5011
Sumo2	1825	1504	2415	2080
Sumo3	4064	3431	4977	4335
Sumo4	417	554	754	466
Sun1	1034	1512	1277	1069
Sun2	3769	2845	3489	3193
Suox	446	393	466	306
Supt16h	1870	2416	2665	2102
Supt20h	419	463	550	658
Supt3h	302	329	259	336
Supt4h1	2580	1999	2051	2342
Supt5h	2728	3053	2919	2749
Supt6h	1619	2221	1857	1909
Supt7l	548	519	445	536
Supv3l1	608	722	504	626
Surf6	493	510	507	419
Susd1	63	81	93	90
Susd2	344	301	361	240
Susd3	103	131	257	399
Susd4	28	25	51	45
Susd5	86	126	42	97
Susd6	1574	1823	2129	2348
Suv39h1	792	803	1023	984
Suv39h2	130	104	51	75
Suz12	550	473	727	618
Sv2a	122	289	206	113
Sv2c	162	392	272	514
Svbp	734	1356	1011	1161
Svep1	160	475	330	450
Svil	2553	2131	1622	2317
Svip	316	119	218	219
Swap70	670	1127	969	901
Swi5	5004	5410	5079	5675

Swsap1	552	426	584	490
Swt1	303	300	271	408
Syap1	882	759	1073	1178
Sybu	45	56	14	42
Syce2	172	93	92	62
Sycp3	79	3	87	32
Syde1	20	59	22	43
Syde2	334	17	232	67
Syf2	3093	2908	3426	3082
Syk	756	997	913	1861
Sympk	320	748	370	505
Syn2	32	9	27	0
Sync	167	327	161	217
Syndig1	18	20	14	60
Syne1	415	1127	542	671
Syne2	1501	1091	1493	527
Syne3	43	82	69	64
Syngap1	21	34	24	13
Syngr1	479	336	248	727
Syngr2	3751	3113	2991	4166
Syngr3	32	0	29	19
Syngr4	12	0	3	0
Synj1	513	733	591	941
Synj2	1139	969	1347	1466
Synj2bp	1933	2018	1505	1928
Synm	5501	3189	2359	4773
Synpo	2096	1565	2070	2307
Synpo2	8701	8118	4499	7340
Synpo2l	4932	7285	5871	4265
Synrg	858	939	900	769
Syp	30	46	35	89
Sypl1	2237	1361	1781	2274
Sypl2	6758	6300	3351	4466
Sys1	1325	1048	1031	1076
Syt11	211	272	215	454
Syt14	0	11	6	0
Syt15	46	17	81	43
Syt16	2	23	8	75
Syt17	10	33	54	47
Syt3	55	34	57	23
Syt5	136	113	117	71
Syt7	137	516	292	92
Syt8	8	37	9	25
Sytl1	738	437	492	140
Sytl3	73	44	23	24
Sytl4	152	211	126	159
Sytl5	4	12	11	8

Syvn1	1375	1596	1395	1548
Szrd1	1550	2351	1288	1832
Szt2	73	112	61	119
Taar4	16	29	11	1
Tab1	831	839	654	661
Tab2	1563	1582	1917	1863
Tab3	126	78	149	125
Tac3	26	521	0	27
Tac4	30	79	138	187
Tacc1	934	2145	1574	2123
Tacc2	3780	3207	3494	2245
Tacc3	987	1425	1256	1458
Taco1	110	139	107	101
Tacr1	258	451	414	310
Tacstd2	4378	1495	2544	1421
Tada1	337	323	260	351
Tada2a	612	394	843	534
Tada2b	457	496	605	673
Tada3	436	656	456	497
Taf1	485	682	539	751
Taf10	2921	2416	2483	2060
Taf11	745	764	662	457
Taf12	1334	1378	1450	1281
Taf13	1779	994	1187	1190
Taf15	729	286	825	441
Taf1a	476	323	317	255
Taf1b	197	158	223	145
Taf1c	330	242	290	274
Taf1d	736	446	676	584
Taf2	572	514	608	623
Taf3	145	433	328	240
Taf4	342	334	271	365
Taf4b	203	116	289	218
Taf5	4	15	62	23
Taf5l	477	289	190	218
Taf6	1600	1538	1532	967
Taf6l	1398	1218	1118	1149
Taf7	244	216	266	269
Taf8	270	413	397	365
Taf9b	283	105	281	166
Tagap	299	292	271	333
Tagln	4822	11446	6727	8481
Tal1	103	222	169	179
Taldo1	4359	5566	4249	5777
Tamm41	847	805	976	1028
Tanc1	797	560	564	796
Tanc2	1036	886	1055	1243

Tango2	881	581	627	532
Tango6	269	312	250	410
Tank	426	412	418	557
Taok1	244	257	345	274
Taok2	1707	2337	2092	2071
Taok3	22	136	54	73
Tap1	1429	1315	1187	1140
Tapbpl	398	623	487	502
Tapt1	1164	693	912	1036
Tarbp1	467	508	285	352
Tarbp2	510	455	432	392
Tardbp	5759	4313	4878	5272
Tars	587	1198	871	871
Tars2	602	710	472	455
Tas1r1	30	8	54	14
Tas2r137	18	12	29	18
Tasp1	224	42	129	104
Tatdn1	1118	873	1202	1142
Tatdn2	1245	1759	1301	1699
Tatdn3	366	257	197	208
Tax1bp1	4565	3994	5137	5450
Tax1bp3	3621	4938	4234	3997
Taz	1932	1456	1658	1437
Tbata	4	16	9	17
Tbc1d1	945	1405	1345	1673
Tbc1d10a	429	589	412	301
Tbc1d10c	296	82	131	147
Tbc1d13	377	691	450	470
Tbc1d14	1269	1733	1441	2268
Tbc1d15	300	365	591	612
Tbc1d16	9	44	20	10
Tbc1d17	784	846	885	892
Tbc1d19	215	156	176	235
Tbc1d2	188	370	259	328
Tbc1d20	2929	3835	3912	4317
Tbc1d22a	41	97	27	70
Tbc1d22b	489	525	534	438
Tbc1d23	705	711	973	1306
Tbc1d24	118	140	102	66
Tbc1d25	244	333	202	315
Tbc1d2b	1468	2311	1827	2389
Tbc1d30	74	62	47	51
Tbc1d31	318	444	341	300
Tbc1d32	141	70	107	145
Tbc1d4	869	698	949	964
Tbc1d5	605	478	558	447
Tbc1d7	52	56	20	7

Tbc1d8	1110	473	752	829
Tbc1d8b	123	210	173	136
Tbc1d9	257	211	244	812
Tbc1d9b	1851	2195	2167	2292
Tbca	4120	3813	3907	3616
Tbcc	622	594	591	499
Tbccd1	68	293	138	152
Tbcd	1020	1353	1186	1300
Tbce	2366	2655	2682	2234
Tbcel	382	492	740	393
Tbck	286	294	274	381
Tbk1	777	761	779	937
Tbkbp1	138	200	152	234
Tbl1x	1479	965	1339	1493
Tbl1xr1	1985	1054	1413	1900
Tbl2	651	921	927	965
Tbl3	651	866	534	376
Tbp	216	254	325	333
Tbpl1	1216	945	943	945
Tbrg1	12	89	56	40
Tbrg4	774	1368	837	632
Tbx1	451	199	609	132
Tbx10	20	31	35	11
Tbx15	50	110	39	108
Tbx18	165	180	199	179
Tbx19	125	68	89	45
Tbx2	103	275	149	190
Tbx21	50	0	42	51
Tbx3	214	331	206	184
Tbx6	64	21	35	31
Tbxas1	353	752	441	1697
Tc2n	114	15	129	30
Tcaf1	926	763	867	641
Tcaim	489	557	578	463
Tcap	340	322	137	118
Tcea1	1357	1268	1183	1317
Tcea2	165	236	196	144
Tcea3	606	514	343	405
Tceal1	115	141	122	89
Tceal5	803	542	478	384
Tceal7	5787	2692	1497	3249
Tceal8	1872	2523	2243	2860
Tceal9	1617	2165	2487	2898
Tceanc	9	5	2	30
Tceanc2	309	328	220	290
Tceb1	822	716	642	438
Tceb2	1556	1823	1274	1424

Tceb3	893	1221	1002	1264
Tcerg1	1192	1032	1413	1348
Tcf12	1477	939	1444	1815
Tcf15	55	59	65	64
Tcf19	617	765	725	628
Tcf20	894	748	949	783
Tcf23	20	64	45	81
Tcf25	3778	4195	3820	4386
Tcf3	713	807	632	483
Tcf4	1752	1422	1805	1768
Tcf7	898	802	787	557
Tcf7l1	548	615	671	461
Tcf7l2	506	377	584	470
Tcfl5	91	23	63	31
Tchh	2734	510	5073	47
Tchhl1	19	1	41	0
Tchp	533	558	659	587
Tcirg1	1457	2063	2027	4558
Tcn2	1345	3395	2070	3346
Tcof1	726	1280	713	691
Tcp1	796	1060	1378	1350
Tcp11l1	96	146	77	219
Tcp11l2	1123	867	1159	1170
Tcta	2254	1722	1766	2064
Tctex1d2	477	480	606	916
Tctn1	582	485	481	386
Tctn2	358	423	477	340
Tdg	2707	1810	2612	2347
Tdgf1	2	29	11	0
Tdh	0	23	11	12
Tdo2	205	104	114	114
Tdp1	217	159	114	144
Tdp2	856	442	603	486
Tdrd1	11	0	3	11
Tdrd3	430	317	457	374
Tdrd7	457	806	525	639
Tdrkh	109	9	21	69
Tdrp	29	52	74	59
Tead1	577	1080	942	1103
Tead2	39	94	64	37
Tead3	630	669	736	623
Tead4	667	1051	925	450
Tec	325	547	710	1161
Tecpr1	658	715	648	1201
Tecpr2	685	462	489	480
Tecr	45	24	14	45
Tecta	110	31	95	8

Tectb	81	28	6	17
Tef	1533	1786	1413	1798
Tefm	222	222	193	156
Tek	523	973	1007	699
Tekt1	29	20	69	7
Tekt2	7	24	8	0
Tekt3	3	0	5	0
Tekt5	9	0	5	4
Telo2	765	686	705	962
Ten1	462	593	378	472
Tenm2	34	93	78	20
Tenm3	393	294	304	341
Tenm4	1512	790	1165	420
Tep1	1696	2119	2433	2341
Tepp	659	565	709	571
Tepsin	331	509	328	326
Terb1	4	0	6	0
Terf1	391	451	460	394
Terf2	495	526	609	607
Terf2ip	1011	861	1130	1344
Tert	61	51	59	82
Tes	1973	1903	1944	3095
Tesc	42	10	41	15
Tesk1	632	979	547	730
Tesk2	6	25	40	0
Tet1	8	32	24	8
Tet2	464	580	540	302
Tet3	544	516	785	447
Tex10	639	613	477	478
Tex12	22	23	0	17
Tex2	514	580	633	486
Tex261	3079	4036	3163	4121
Tex264	1670	1882	1401	2238
Tex30	349	305	271	264
Tex40	379	160	254	142
Tex9	377	314	457	214
Tf	1931	4769	3444	3806
Tfam	1697	1189	1038	1251
Tfap2a	564	305	545	196
Tfap2b	941	655	746	544
Tfap2c	2158	1075	1703	540
Tfap2e	427	286	287	106
Tfap4	306	250	209	136
Tfb1m	353	468	261	596
Tfb2m	899	634	648	700
Tfcp2	430	312	506	361
Tfcp2l1	14	36	26	0

Tfdp1	666	1094	826	734
Tfdp2	849	511	722	562
Tfe3	1291	1654	1848	2072
Tfeb	455	776	444	680
Tfec	274	354	299	611
Tff3	0	11	5	0
Tfg	1275	1593	1301	2099
Tfip11	846	937	655	697
Tfpi	421	631	519	830
Tfpi2	275	241	423	743
Tfpt	688	493	799	721
Tfrc	1880	1044	868	1463
Tg	20	40	11	15
Tgds	271	452	334	484
Tgfa	329	386	320	162
Tgfb1	1506	2524	1920	3248
Tgfb1i1	722	1361	964	1261
Tgfb2	663	993	1055	876
Tgfb3	636	1845	1031	967
Tgfbi	24060	26541	28524	40160
Tgfbr1	131	366	497	1030
Tgfbr2	4624	6991	6889	11463
Tgfbr3	2134	2276	2496	2478
Tgfbrap1	610	743	546	643
Tgif1	2887	1383	1918	1638
Tgif2	112	114	126	106
Tgm2	3151	5352	3825	6011
Tgm3	3276	891	694	375
Tgm4	0	0	1	0
Tgm6	49	18	141	0
Tgm7	237	296	205	169
Tgm7l1	1311	1354	484	405
Tgoln2	1305	1192	1545	2019
Tgs1	307	316	287	318
Thada	561	551	448	500
Thap1	68	69	113	20
Thap11	1302	975	1253	1276
Thap12	1577	1688	1757	1965
Thap2	260	210	229	261
Thap3	695	734	427	320
Thap4	918	792	823	805
Thap6	265	274	378	316
Thap7	356	342	205	320
Thbd	784	1085	1059	904
Thbs1	4590	3527	8863	2549
Thbs2	4729	8248	8677	20613
Thbs3	630	2283	662	1067

Thbs4	2399	8501	2009	369
Thegl	39	22	2	32
Them4	249	266	251	277
Them5	1824	1024	1049	500
Them6	101	119	95	214
Themis2	556	451	682	701
Thg1l	544	578	606	561
Thnsl1	135	98	141	143
Thnsl2	295	323	253	466
Thoc1	1957	1184	1869	1724
Thoc2	392	292	290	219
Thoc3	832	1084	877	1150
Thoc5	1114	1282	1043	1227
Thoc6	952	1080	807	694
Thoc7	2289	1898	1687	1985
Thop1	358	568	301	331
Thra	844	817	592	605
Thrap3	2134	3051	2648	2342
Thrb	129	113	144	162
Thrsp	1703	535	1028	727
Thsd1	304	547	442	285
Thsd4	325	225	442	152
Thsd7a	5	27	63	85
Thtpa	90	140	239	232
Thumpd1	1410	946	1242	1064
Thumpd2	95	162	101	96
Thumpd3	1065	566	662	975
Thumpd3-a	420	119	284	219
Thy1	12196	25480	20530	28685
Thyn1	3436	1047	1828	3253
Tia1	1105	842	1316	702
Tial1	1215	1246	1183	1620
Tiam1	1513	977	1365	919
Tiam2	214	241	246	344
Ticam2	66	90	74	219
Ticrr	0	150	45	31
Tie1	1280	2830	2866	3145
Tifa	365	469	495	958
Tifab	151	241	196	432
Tigar	516	475	375	763
Tigd2	237	300	320	281
Tigd3	82	72	65	50
Tigd4	20	0	15	11
Tigd5	40	60	23	44
Timd2	15	8	30	27
Timd4	322	428	302	523
Timeless	359	491	370	444

Timm10	772	1271	722	659
Timm13	566	1016	558	487
Timm17al1	290	310	331	440
Timm21	732	791	626	606
Timm22	2449	3144	2755	3550
Timm23	560	706	460	514
Timm44	1551	1742	1225	1573
Timm50	1457	1358	1207	1320
Timm8a1	1865	782	1098	1138
Timm8b	4037	3349	2905	3410
Timm9	1176	693	743	796
Timmdc1	931	1054	969	890
Timp2	12523	23717	16914	31803
Timp3	15581	5814	21315	5666
Timp4	182	149	196	170
Tinagl1	923	1899	1204	1478
Tincr	625	339	492	381
Tinf2	573	913	685	570
Tiparp	1340	853	725	714
Tipin	28	6	59	19
Tipinl1	302	305	396	241
Tiprl	707	676	928	598
Tirap	118	96	92	216
Tjap1	672	1234	818	713
Tjp1	587	556	852	733
Tjp2	1541	923	1243	913
Tjp3	15	22	16	15
Tk1	741	1538	871	972
Tk2	522	438	487	525
Tkfc	244	345	313	309
Tkt	7071	8999	6789	7888
Tlcd1	1491	674	864	577
Tlcd2	418	189	166	353
Tldc1	21	14	26	0
Tle1	632	251	426	237
Tle2	158	68	81	61
Tle3	868	1303	1132	1598
Tle4	530	434	414	445
Tlk2	1204	838	1515	1555
Tll1	74	60	110	127
Tln1	21	20	5	6
Tln2	1584	1646	1286	1160
Tlr1	274	60	187	59
Tlr10	27	52	66	76
Tlr11	74	36	38	32
Tlr12	39	32	51	16
Tlr13	775	1273	1717	3295

Tlr2	749	1360	1047	1700
Tlr3	340	272	286	588
Tlr4	174	155	229	258
Tlr5	18	19	42	27
Tlr6	146	225	164	265
Tlr7	324	494	486	1420
Tlr8	25	92	65	73
Tm2d1	862	394	679	869
Tm2d2	3387	3332	3351	3632
Tm2d3	456	377	215	482
Tm4sf1	1812	2441	2127	2108
Tm4sf4	1	5	8	12
Tm6sf1	1033	1236	1029	1262
Tm6sf2	12	9	6	38
Tm7sf2	438	335	259	381
Tm7sf3	1226	1764	1631	2148
Tm9sf1	1374	1500	1651	1985
Tm9sf2	3230	3595	3330	4786
Tm9sf3	5805	5078	5931	7617
Tm9sf4	1978	2083	1831	2087
Tma16	109	145	105	104
Tmbim1	1799	2983	2129	2323
Tmbim4	2175	1985	1870	2772
Tmbim6	11493	11694	9066	11224
Tmc3	37	12	60	16
Tmc4	1255	358	806	436
Tmc6	624	681	856	573
Tmc7	100	38	69	27
Tmc8	25	33	24	27
Tmcc1	495	647	773	839
Tmcc2	196	113	66	158
Tmcc3	1845	719	1341	742
Tmco1	1059	727	775	954
Tmco4	301	291	345	264
Tmco6	360	372	288	480
Tmed1	787	966	597	586
Tmed10	8502	9274	8179	13794
Tmed2	9677	10459	10284	15120
Tmed3	2537	4404	3698	6177
Tmed4	827	805	891	893
Tmed5	78	132	75	118
Tmed7	976	854	1052	1296
Tmed8	310	276	381	362
Tmed9	6377	6703	7016	8486
Tmeff1	36	43	47	34
Tmeff2	465	443	326	219
Tmem100	1116	1116	990	1365

Tmem101	493	582	295	531
Tmem102	129	50	74	29
Tmem104	485	742	522	800
Tmem106a	791	662	834	719
Tmem106b	274	310	372	358
Tmem106c	232	300	263	209
Tmem107	336	386	487	242
Tmem108	26	106	59	144
Tmem109	6438	8473	4836	5607
Tmem11	1428	1390	1241	1012
Tmem110	661	540	448	446
Tmem115	866	1413	626	885
Tmem116	30	49	11	1
Tmem117	617	849	552	336
Tmem119	849	2402	1401	3492
Tmem120a	1363	1757	1550	1745
Tmem120b	213	305	203	320
Tmem121	30	94	35	24
Tmem123	2071	1432	2600	3276
Tmem126a	1276	691	701	1084
Tmem126b	784	589	500	673
Tmem127	2349	2578	3664	3531
Tmem128	1713	1384	1407	1851
Tmem129	675	823	787	1016
Tmem130	0	5	14	0
Tmem131	1659	1329	1569	1611
Tmem132b	56	0	17	0
Tmem132c	70	44	35	71
Tmem132e	40	115	48	148
Tmem134	3924	2932	3272	2837
Tmem135	605	319	500	683
Tmem138	217	361	358	277
Tmem139	39	54	102	72
Tmem140	917	1013	895	950
Tmem143	438	477	337	363
Tmem144	206	257	131	300
Tmem147	1850	1697	1705	1564
Tmem14c	3065	2703	2812	4787
Tmem150a	1222	989	1164	1734
Tmem150b	78	86	149	165
Tmem150c	126	91	96	172
Tmem154	2302	889	1777	1230
Tmem156	76	64	65	32
Tmem159	1783	1022	1410	1297
Tmem160	365	405	256	251
Tmem161a	587	798	573	390
Tmem161b	128	132	205	221

Tmem164	1149	852	1186	1378
Tmem165	1799	1249	1348	2368
Tmem167a	2443	2571	2403	3613
Tmem167b	206	231	187	202
Tmem168	959	518	707	1041
Tmem17	564	538	450	671
Tmem170a	4	27	35	12
Tmem170b	6	2	5	2
Tmem171	19	0	5	58
Tmem173	377	569	451	671
Tmem175	1465	1206	1494	1157
Tmem176a	1853	2420	2460	4132
Tmem176b	5457	6738	8162	14525
Tmem177	152	196	101	129
Tmem178a	259	173	290	492
Tmem18	12	38	20	52
Tmem181	1201	1186	1168	1442
Tmem182	1001	1747	1274	1042
Tmem183a	2369	2360	2671	2518
Tmem184a	353	121	214	154
Tmem184b	1287	2135	1692	2102
Tmem184c	453	492	313	557
Tmem185a	1218	1232	934	1115
Tmem185b	1003	862	882	1167
Tmem186	583	473	560	485
Tmem189	1698	2496	2081	2707
Tmem19	879	610	814	1051
Tmem190	48	74	44	39
Tmem191c	3	14	2	15
Tmem192	685	619	897	701
Tmem198	11	38	5	19
Tmem198b	3	49	22	0
Tmem199	1652	1768	1640	1532
Tmem2	996	1214	1666	1201
Tmem200a	104	94	80	99
Tmem200b	342	399	426	249
Tmem200c	26	33	17	0
Tmem201	375	256	215	240
Tmem203	291	551	379	300
Tmem204	574	688	460	491
Tmem205	508	403	322	233
Tmem206	160	370	272	441
Tmem208	1072	1271	835	1193
Tmem209	634	594	709	576
Tmem212	14	0	27	0
Tmem214	1347	2417	1509	2105
Tmem216	622	457	489	384

Tmem218	293	263	453	419
Tmem220	163	113	172	247
Tmem222	532	552	498	501
Tmem229a	471	102	211	294
Tmem229b	671	791	885	1203
Tmem230	1695	1169	1310	1787
Tmem231	108	251	188	147
Tmem233	491	281	90	401
Tmem234	350	386	272	444
Tmem237	280	371	301	156
Tmem238	64	116	45	53
Tmem241	18	55	48	33
Tmem242	1182	1422	973	1005
Tmem243	640	493	523	642
Tmem245	1121	961	1237	1205
Tmem246	442	468	328	352
Tmem248	3398	3317	3239	3730
Tmem249	48	40	47	19
Tmem25	37	42	17	54
Tmem251	540	263	494	492
Tmem252	491	523	457	315
Tmem255b	69	166	110	72
Tmem256	1260	1287	1365	1390
Tmem258	407	695	611	1055
Tmem258b	1892	1822	1583	2301
Tmem259	432	854	416	566
Tmem26	210	131	211	633
Tmem260	768	747	773	715
Tmem261	809	714	715	741
Tmem263	1149	772	1088	2297
Tmem267	180	75	84	153
Tmem268	500	501	548	704
Tmem30a	2072	1961	1756	3485
Tmem30b	685	131	472	321
Tmem30c	18	30	0	10
Tmem33	453	351	373	432
Tmem35	218	110	212	81
Tmem35b	133	157	161	93
Tmem37	278	489	388	627
Tmem38a	12837	11992	5824	9438
Tmem38b	1302	2138	1628	1046
Tmem39a	496	606	782	1043
Tmem39b	298	333	208	263
Tmem40	894	444	406	223
Tmem41a	1021	801	934	606
Tmem41b	1049	826	662	906
Tmem42	785	682	521	479

Tmem43	1606	3302	1813	3107
Tmem44	142	237	187	355
Tmem45al	172	110	36	89
Tmem45b	450	274	670	302
Tmem47	997	956	698	844
Tmem5	641	943	730	947
Tmem50a	3434	4273	3823	5258
Tmem50b	685	859	495	603
Tmem51	422	449	376	477
Tmem52	894	684	214	436
Tmem53	215	125	152	99
Tmem54	459	206	382	126
Tmem55a	1153	764	855	837
Tmem55b	732	1042	777	1211
Tmem56	6038	1294	1542	3547
Tmem57	1008	949	937	1346
Tmem59	6137	5340	6393	7935
Tmem60	614	504	513	688
Tmem62	633	459	447	565
Tmem63a	654	675	656	691
Tmem63b	965	1311	904	731
Tmem63c	33	143	63	62
Tmem64	522	286	393	431
Tmem65	1203	861	837	1311
Tmem67	93	125	93	49
Tmem68	291	305	345	449
Tmem69	1058	901	909	823
Tmem70	1030	700	847	729
Tmem71	101	105	89	69
Tmem79	528	313	555	271
Tmem80	492	488	399	371
Tmem81	15	37	17	41
Tmem82	52	40	63	4
Tmem86a	1135	1026	1064	2398
Tmem87a	1811	1370	1286	1434
Tmem87b	717	506	614	1035
Tmem88	140	298	380	7
Tmem8a	214	550	391	383
Tmem8b	163	174	126	149
Tmem8c	192	3626	1124	402
Tmem9	981	1301	1094	953
Tmem91	65	34	11	21
Tmem97	3490	2730	2107	4232
Tmem98	587	588	549	807
Tmem9b	1633	970	1072	1375
Tmf1	112	128	209	253
Tmlhe	421	362	348	356

Tmod1	3270	5233	2931	2792
Tmod2	113	155	105	166
Tmod3	1551	2333	1994	1998
Tmod4	4574	3085	1729	3527
Tmpo	26	8	0	12
Tmppe	114	221	101	142
Tmprss11e	211	49	51	78
Tmprss11f	42	6	0	30
Tmprss13	833	343	563	156
Tmprss2	57	14	33	42
Tmprss4	569	203	278	71
Tmprss5	41	55	66	27
Tmprss6	22	18	38	63
Tmprss7	87	9	117	27
Tmsb10	153	369	177	225
Tmsb4x	82091	92736	76892	117655
Tmsbl1	152	94	30	87
Tmtc1	170	208	235	232
Tmtc2	101	78	141	145
Tmtc3	432	305	363	309
Tmtc4	515	532	621	542
Tmub1	335	421	346	352
Tmub2	1894	2179	2120	1769
Tmx1	2427	1856	2231	3083
Tmx2	2072	1961	1756	2058
Tmx3	1122	1228	1461	1562
Tmx4	809	550	725	942
Tnc	10554	28205	23386	26258
Tnfaip1	3034	3669	3557	3590
Tnfaip2	545	1451	940	1298
Tnfaip3	585	401	650	515
Tnfaip6	471	639	1100	1501
Tnfaip8	1727	1004	1461	1237
Tnfaip8l1	233	366	319	245
Tnfaip8l2	1706	2208	2215	4744
Tnfaip8l3	334	277	182	261
Tnfrsf10b	303	634	465	503
Tnfrsf11a	139	158	200	220
Tnfrsf11b	48	180	123	72
Tnfrsf12a	3194	4387	3164	2795
Tnfrsf13b	12	0	29	10
Tnfrsf14	324	88	135	153
Tnfrsf18	852	655	487	284
Tnfrsf19	14	20	36	8
Tnfrsf1a	3833	4441	4439	4539
Tnfrsf1b	1430	1723	1769	3532
Tnfrsf21	1125	1447	1214	1876

Tnfrsf22	299	317	146	255
Tnfrsf26	206	412	391	258
Tnfrsf4	68	20	93	64
Tnfrsf8	85	109	99	221
Tnfrsf9	112	12	80	56
Tnfsf10	250	138	370	172
Tnfsf12	1792	2435	2489	5995
Tnfsf13b	57	80	122	209
Tnfsf14	37	16	23	27
Tnfsf18	18	60	71	36
Tnfsf8	6	12	24	134
Tnfsf9	124	119	116	166
Tnik	545	203	513	384
Tnip1	1963	2282	2117	2442
Tnip2	560	614	623	742
Tnip3	14	5	23	8
Tnk1	1137	594	893	634
Tnk2	803	660	596	326
Tnks	129	127	214	144
Tnks1bp1	2398	2887	2544	2127
Tnmd	397	190	147	218
Tnn	4151	10306	9250	29719
Tnnc1	4787	8937	5905	4760
Tnnc2	105464	71085	35475	61180
Tnni1	4348	17788	4529	4564
Tnni2	78736	52858	27830	43543
Tnnt1	4349	10697	6270	6822
Tnnt2	511	11330	2672	1050
Tnnt3	111103	94130	61258	82849
Tnpo1	1639	1305	1620	2033
Tnpo2	2854	2042	2982	1890
Tnpo3	2620	2332	2073	2205
Tnrc18	891	1085	1133	793
Tnrc6a	1496	912	1213	1273
Tnrc6b	706	731	840	536
Tnrc6c	1179	774	799	820
Tns1	2756	6559	3638	4818
Tns2	675	1721	1001	944
Tns3	1633	2130	2881	4706
Tns4	1771	1720	1137	1014
Tnxa-ps1	2455	6672	1553	1098
Tob1	445	430	418	498
Tob2	1363	1065	1091	831
Toe1	512	738	577	836
Tollip	1986	2084	1852	1982
Tom1	1183	1694	1222	1104
Tom1l1	571	349	644	465

Tom1l2	1823	1573	1602	1237
Tomm20	4467	3523	4633	4407
Tomm20l	47	14	17	115
Tomm22	5927	4296	4559	4517
Tomm34	245	517	385	367
Tomm40	1028	1712	679	642
Tomm40l	938	864	946	1086
Tomm5	3018	2716	2054	2287
Tomm7	3842	3067	3652	3754
Tomm70	477	165	330	340
Tonsl	56	64	51	26
Top1	818	534	894	809
Top1mt	298	534	269	193
Top2a	1257	1913	1855	1494
Top2b	1771	1818	2663	1923
Top3a	138	68	111	106
Top3b	504	458	457	351
Topaz1	18	0	6	0
Topbp1	358	453	333	392
Topors	481	579	767	547
Tor1a	761	1214	897	892
Tor1aip1	1382	1288	1250	1900
Tor1aip2	3912	3082	3215	3623
Tor1b	1153	993	952	1055
Tor2a	761	1217	996	1435
Tor4a	256	308	364	484
Tox	36	0	42	29
Tox3	24	12	15	0
Tox4	699	1514	1052	828
Tp53	3717	3300	3751	2583
Tp53bp1	600	837	653	553
Tp53bp2	352	375	364	362
Tp53i11	698	1361	828	505
Tp53i13	133	344	123	301
Tp53i3	627	742	548	583
Tp53inp1	184	248	239	232
Tp53inp2	6037	5459	5060	6873
Tp53rk	150	174	259	94
Tp63	4611	2407	4733	1969
Tp73	539	348	376	140
Tpbg	1074	954	1408	1310
Tpbgl	192	305	346	644
Tpcn1	945	1992	1041	1269
Tpcn2	315	280	387	330
Tpd52	2549	1394	1857	1784
Tpd52l1	714	148	417	191
Tpd52l2	2002	2037	1599	1590

Tpgs1	211	256	166	93
Tpgs2	1253	1664	1470	1925
Tph1	102	67	51	11
Tpk1	167	115	202	177
Tpm1	140627	118692	77973	111011
Tpm2	85508	92275	59808	62348
Tpm3	3553	5897	4637	8153
Tpm4	4789	2435	5324	8128
Tpmt	911	876	919	1295
Tpp1	4775	4418	4383	8471
Tpp2	1326	1079	924	1146
Tppp	622	383	480	291
Tppp2	115	20	21	73
Tppp3	2101	7032	1861	2084
Tpr	1789	3069	3079	2779
Tpra1	345	699	414	568
Tprg1	923	52	692	150
Tprg1l	2731	2773	3045	3300
Tprn	309	390	289	399
Tpsab1	106	160	69	7
Tpsb2	235	233	220	51
Tpst1	430	600	436	632
Tpst2	1390	1897	1607	2427
Tpt1	52584	30689	60810	69493
Tpx2	884	1265	934	947
Tra2a	1049	1046	1326	1027
Tra2b	3062	1426	1938	2148
Trabd	793	1012	551	716
Trabd2b	151	296	369	190
Tradd	791	936	818	492
Traf1	563	300	555	514
Traf2	720	712	497	575
Traf3	964	756	946	828
Traf3ip1	148	204	229	205
Traf3ip2	467	431	355	343
Traf3ip3	121	197	245	348
Traf4	357	413	402	361
Traf5	341	286	330	240
Traf6	128	72	158	127
Traf7	1455	2169	1493	2034
Trafd1	1710	2125	1730	1464
Traip	113	167	150	133
Trak1	3433	3597	2403	3014
Trak2	868	1518	1085	1582
Tram1	5971	5644	6709	7996
Tram1l1	9	30	2	0
Tram2	118	50	39	33

Trank1	0	11	0	11
Trap1	1659	1977	1675	1414
Trappc1	2763	2941	2678	2769
Trappc10	1035	787	1026	816
Trappc11	889	860	889	980
Trappc12	712	621	555	667
Trappc13	1181	1343	1429	1833
Trappc2	344	244	301	443
Trappc2l	684	935	689	645
Trappc3	1899	1934	1601	2025
Trappc4	1675	1254	1551	1227
Trappc5	1644	1334	1474	1134
Trappc6a	916	837	715	535
Trappc6b	703	648	516	1036
Trappc8	545	949	692	1074
Trappc9	666	835	534	657
Trat1	14	0	17	0
Trdmt1	356	275	489	344
Trdn	7333	4709	2314	4730
Treh	725	729	748	747
Trem1	343	670	403	976
Trem2	1219	1699	2010	6411
Trem3	564	352	412	603
Treml1	7	16	32	61
Trerf1	361	572	561	585
Trex2	473	77	68	48
Trh	94	75	75	44
Trhde	7	0	8	29
Trib1	671	671	847	542
Trib2	586	494	656	468
Trib3	21	36	18	5
Tril	1717	707	1876	1378
Trim11	602	473	645	437
Trim14	64	12	42	119
Trim15	76	50	95	184
Trim16	532	274	293	352
Trim2	210	206	226	335
Trim21	334	261	284	223
Trim23	90	152	137	117
Trim24	605	619	694	782
Trim25	2034	2171	2371	2122
Trim26	919	802	890	810
Trim27	2009	2010	2088	2195
Trim28	3432	4045	4132	3562
Trim29	20140	8728	14662	5567
Trim3	778	1088	850	1064
Trim30	130	223	126	223

Trim32	402	392	379	513
Trim33	810	509	861	665
Trim34	157	157	287	292
Trim35	2545	2344	1702	2423
Trim36	66	110	99	112
Trim37	591	761	691	634
Trim39	658	442	727	341
Trim41	112	248	209	266
Trim43a	0	3	9	0
Trim44	59	27	113	59
Trim45	290	193	223	196
Trim46	20	50	38	110
Trim47	707	1193	676	945
Trim5	641	607	706	676
Trim54	1407	1778	662	558
Trim55	974	1348	1092	775
Trim59	938	599	698	653
Trim6	0	56	3	0
Trim62	363	316	402	362
Trim63	1718	3135	1107	1852
Trim65	247	272	292	386
Trim66	14	9	15	0
Trim69	53	62	47	61
Trim7	99	56	71	113
Trim72	5357	7581	3934	3213
Trim8	1136	2180	1514	1892
Trio	385	859	590	616
Triobp	1308	1568	1366	997
Trip10	1456	1660	1323	1411
Trip11	884	867	892	737
Trip12	4619	3731	4586	5265
Trip13	156	215	215	262
Trip4	163	56	105	86
Trip6	1490	1202	1610	1105
Triqk	83	122	140	187
Trit1	677	826	743	773
Trmo	56	109	71	91
Trmt1	908	1165	809	827
Trmt10a	459	350	299	208
Trmt10b	389	370	403	425
Trmt11	210	214	254	236
Trmt112	3774	3198	3147	2936
Trmt12	178	166	248	277
Trmt13	228	198	195	170
Trmt1l	515	511	462	465
Trmt2a	753	633	697	858
Trmt44	251	244	185	259

Trmt5	293	376	405	296
Trmt6	1006	658	1183	863
Trmu	154	263	169	199
Trnp1	282	398	257	461
Trnt1	518	448	208	222
Tro	6	31	5	67
Troap	64	199	113	54
Trove2	35	46	38	40
Trpc1	13	16	20	52
Trpc3	29	0	71	50
Trpc4ap	1915	2883	2085	2588
Trpc6	150	223	259	191
Trpm1	17	56	14	8
Trpm2	128	128	166	313
Trpm3	47	58	33	25
Trpm4	269	450	236	187
Trpm7	697	751	745	886
Trpm8	69	84	89	96
Trps1	405	320	450	475
Trpt1	317	304	140	408
Trpv1	17	189	18	31
Trpv2	962	1689	1091	3903
Trpv3	233	82	135	93
Trpv4	112	217	110	138
Trpv6	1241	325	1123	234
Trrap	419	542	739	501
Trub1	370	309	349	314
Trub2	946	1231	728	996
Tsacc	12	0	12	8
Tsc1	543	691	536	491
Tsc2	1454	2215	1631	2103
Tsc22d1	19043	16938	10637	11992
Tsc22d2	425	246	311	352
Tsc22d3	4754	3612	3190	3486
Tsen15	755	739	670	627
Tsen2	286	314	247	331
Tsen54	310	384	238	264
Tsfm	169	333	137	169
Tsg101	2289	2676	2886	3190
Tsga10	22	34	6	15
Tshb	0	19	15	0
Tshr	100	66	110	85
Tshz1	255	360	342	225
Tshz2	232	561	503	373
Tshz3	176	342	158	296
Tsku	1084	1510	1216	1811
Tslp	82	142	116	116

Tsn	2478	2048	2197	2758
Tsnax	1716	1875	1482	2053
Tspan1	41	56	29	3
Tspan11	553	631	976	1208
Tspan12	1108	1011	957	665
Tspan13	1093	1293	1262	1110
Tspan14	1310	1673	1951	1317
Tspan15	345	391	367	209
Tspan17	479	696	445	352
Tspan18	97	455	221	877
Tspan2	348	920	639	425
Tspan3	2440	2817	2487	3448
Tspan31	1430	1692	1635	2276
Tspan32	29	6	26	27
Tspan33	93	172	120	52
Tspan5	863	1100	951	1675
Tspan6	1786	1010	1535	1650
Tspan7	1266	1225	1461	768
Tspan8	2683	582	1462	1261
Tspan9	2456	2854	2943	1752
Tspo	2198	3575	1581	2028
Tspoap1	63	19	53	6
Tspy26	28	18	17	24
Tspyl1	1007	874	1053	1362
Tspyl2	347	389	519	462
Tspyl5	178	61	178	152
Tsr1	3016	3278	3191	2905
Tsr3	597	777	593	742
Tssc1	645	620	456	547
Tssc4	602	964	597	689
Tssk3	8	0	2	2
Tssk4	75	36	111	23
Tst	609	375	408	195
Tsta3	993	935	948	835
Tstd1	1043	1261	1270	998
Tstd2	142	181	137	265
Tstd3	303	554	463	606
Ttbk2	26	14	12	15
Ttc1	2281	2247	2021	2387
Ttc12	19	31	21	0
Ttc13	1380	1798	1804	1867
Ttc14	369	151	254	348
Ttc16	28	23	18	0
Ttc17	678	771	697	733
Ttc19	1338	803	808	1173
Ttc21a	5	11	57	0
Ttc21b	151	136	214	78

Ttc22	296	205	181	26
Ttc23	386	279	287	245
Ttc25	36	49	41	115
Ttc26	218	178	167	260
Ttc27	1272	1011	894	1164
Ttc28	319	567	516	425
Ttc3	1736	2005	1843	2296
Ttc30a	98	54	32	49
Ttc30a1	27	32	8	0
Ttc30b	232	118	150	118
Ttc32	254	114	191	132
Ttc33	756	373	703	643
Ttc37	921	819	837	1147
Ttc38	422	320	418	367
Ttc39a	6	0	3	0
Ttc39b	344	222	379	275
Ttc39c	1178	1073	1347	1315
Ttc4	904	1039	812	904
Ttc5	422	364	366	456
Ttc7a	2336	2436	2505	2659
Ttc7b	603	917	659	1066
Ttc8	187	108	138	95
Ttc9	475	1033	570	596
Ttc9c	1175	984	1205	1309
Ttf1	232	295	248	193
Ttf2	264	207	238	353
Tti1	543	384	447	326
Tti2	313	294	240	369
Ttk	313	256	340	281
Ttl	975	1245	885	1193
Ttll1	132	117	131	77
Ttll11	50	35	42	5
Ttll12	1336	1135	1192	662
Ttll3	47	23	29	13
Ttll4	615	751	809	522
Ttll5	240	229	227	201
Ttll7	514	268	439	553
Ttn	83655	70549	48116	48860
Ttpal	1068	1249	1411	2363
Ttyh1	60	40	50	63
Ttyh2	75	264	146	265
Ttyh3	1203	1553	2085	3268
Tub	65	42	122	94
Tuba1a	25242	29682	22824	22219
Tuba1c	13	163	47	16
Tuba4a	7519	9428	7139	4723
Tuba8	1028	600	992	618

Tubb2b	352	829	447	248
Tubb3	289	907	307	423
Tubb4a	16	25	53	21
Tubb4b	9622	9743	6276	5905
Tubb5	14439	23641	17370	21091
Tubb6	6289	19778	9264	7375
Tubd1	15	33	32	29
Tube1	194	241	159	81
Tubg1	687	1305	602	919
Tubgcp3	272	404	387	403
Tubgcp4	647	752	632	492
Tubgcp5	418	376	307	389
Tubgcp6	140	190	110	152
Tufm	3197	3763	2773	3388
Tuft1	712	243	456	130
Tug1	176	190	92	109
Tulp3	743	816	850	734
Tulp4	200	228	269	238
Tusc2	933	1058	1016	879
Tusc3	663	873	966	705
Tusc5	303	512	527	304
Tut1	403	607	439	417
Tvp23b	320	472	563	538
Twf1	2248	2291	1204	1831
Twf2	875	1235	633	866
Twist1	826	667	645	2105
Twist2	537	557	688	1166
Twistnb	765	589	671	613
Twsg1	914	952	873	1315
Txk	80	25	29	33
Txlna	1323	1528	1284	1664
Txlnb	757	530	396	475
Txlng	358	197	269	158
Txn1	11886	11081	14325	10692
Txn2	2806	3613	2300	3306
Txndc11	626	1069	887	1033
Txndc12	1476	2195	1854	2711
Txndc15	51	98	175	234
Txndc16	175	217	337	482
Txndc17	7059	6363	7169	5598
Txndc5	4524	8883	5538	9446
Txndc9	2024	1273	1572	2089
Txnip	15454	9688	15578	12058
Txnl1	4362	3279	4446	4599
Txnl4b	484	658	659	554
Txnrd1	1352	1646	1449	1722
Txnrd2	616	475	459	481

Txnrd3	573	369	749	399
Tyk2	1395	1160	1273	1472
Tymp	27	88	85	22
Tyms	100	149	69	210
Tyro3	619	317	409	193
Tyrobp	6004	7300	8191	15691
Tysnd1	659	560	689	633
Tyw1	121	252	98	284
Tyw3	102	193	132	152
Tyw5	244	186	217	222
U2af1	3841	4061	3522	3301
U2af1l4	858	924	754	678
U2af2	2871	3037	2538	2417
U2surp	645	408	703	689
Uaca	1147	1682	1810	1689
Uap1	1073	2068	1071	1639
Uap1l1	679	1040	573	901
Uap1l2	265	899	358	487
Uba1	5969	8118	7106	6202
Uba2	513	630	615	573
Uba3	788	875	889	959
Uba5	2600	2851	3193	4061
Uba52	9228	10647	5936	5604
Uba6	293	267	290	381
Uba7	158	158	126	107
Ubac1	2553	2313	1593	1786
Ubac2	1415	1498	1228	1752
Ubald1	58	58	29	34
Ubald2	4435	2543	2501	2144
Ubap1	1653	1261	1375	1203
Ubap1l	49	68	89	15
Ubap2	2620	2101	1966	1591
Ubash3b	629	810	760	795
Ubb	2780	6006	2780	4051
Ubc	11080	8974	8445	9268
Ubd	824	271	900	210
Ube2a	1741	1461	1551	1845
Ube2c	858	1214	1019	1106
Ube2d1	1901	1746	1560	1640
Ube2d2	4980	3266	4043	5160
Ube2d3	7339	5399	5270	7562
Ube2d4	9	5	11	30
Ube2e1	2611	1414	1389	2108
Ube2e2	833	895	1059	1043
Ube2e3	1777	1428	1813	1899
Ube2f	1946	1689	1986	2149
Ube2g1	1327	982	1043	1255

Ube2g2	1109	1806	898	1229
Ube2h	4301	3400	4347	3541
Ube2i	1960	1765	2045	1465
Ube2j1	2552	2118	2501	2927
Ube2j2	2179	2095	1998	1878
Ube2k	3605	3440	3319	3592
Ube2l3	6067	6310	5982	7192
Ube2l6	537	698	671	817
Ube2n	269	198	248	218
Ube2o	440	580	477	466
Ube2q1	2317	2153	2792	2572
Ube2q2	43	101	24	69
Ube2q2l	82	73	110	135
Ube2ql1	61	14	18	53
Ube2r2	4654	4083	4490	5338
Ube2s	3116	3947	2916	2871
Ube2t	143	253	146	123
Ube2u	0	12	53	40
Ube2v1	296	357	262	406
Ube2v2	1407	1089	1411	1289
Ube2w	275	262	277	252
Ube2z	50	131	72	101
Ube3a	366	112	265	330
Ube3b	1235	1394	1241	1212
Ube3c	2097	2421	2033	2011
Ube3d	148	168	110	125
Ube4a	1936	1358	1613	1891
Ube4b	1461	1138	1205	1271
Ubfd1	1116	1499	1428	1618
Ubiad1	315	552	323	413
Ubl3	1060	932	1049	1408
Ubl4a	2295	3347	2526	2588
Ubl5	7022	6365	6220	5380
Ubl7	2228	2339	2401	1894
Ublcp1	1396	1110	1464	1590
Ubn1	336	510	511	1051
Ubn2	1483	766	1207	1112
Ubp1	1633	1345	1633	1149
Ubqln1	4527	3316	3906	3661
Ubqln2	532	394	420	517
Ubqln4	2191	3123	2824	2659
Ubr1	295	450	376	350
Ubr2	1403	1556	1195	1329
Ubr3	1151	991	1208	1279
Ubr4	2174	2584	2624	2157
Ubr5	2760	2234	3000	2495
Ubr7	772	756	763	720

Ubtd1	535	912	433	560
Ubtd2	1002	975	1037	1229
Ubtf	1872	2641	2120	2150
Ubxn1	3733	4752	3740	3953
Ubxn11	191	226	167	151
Ubxn2a	749	347	372	504
Ubxn2b	200	123	159	93
Ubxn4	2832	2889	3041	3320
Ubxn6	1578	1847	1561	1448
Ubxn8	317	365	582	440
Uchl1	424	297	304	245
Uchl5	1451	591	966	1010
Uck1	411	576	629	210
Uck2	458	618	381	377
Uckl1	1492	1229	1276	1048
Ucn2	134	88	75	38
Ucp2	12795	7876	13335	8650
Ucp3	1424	1282	1204	585
Uevld	82	116	87	59
Ufc1	4302	4422	4631	4846
Ufd1l	2997	2416	3414	3374
Ufl1	1021	1169	967	1031
Ufm1	1579	2328	1866	2223
Ufsp2	1341	1704	1980	2079
Ugcg	1786	1600	2534	2825
Ugdh	2316	2212	1851	3154
Uggt1	3282	3270	3241	3388
Uggt2	166	488	456	708
Ugp2	7897	4050	3419	8913
Ugt1a1	3001	2749	2568	4567
Ugt8	24	8	92	50
Uhmk1	65	9	47	45
Uhrf1	347	362	226	340
Uhrf1bp1	646	781	606	616
Uhrf1bp1l	1001	704	870	1140
Uhrf2	1318	1222	1140	1303
Uimc1	1275	1262	1628	1527
Ulk1	1688	1163	1676	1018
Ulk2	1362	874	1110	1224
Ulk4	60	46	41	56
Umps	960	1386	1137	1221
Unc119	310	386	328	575
Unc119b	937	852	1010	1209
Unc13a	5	28	5	10
Unc13b	12	26	69	21
Unc13d	366	166	211	266
Unc45a	1235	1934	1175	1158

Unc45b	946	1842	916	707
Unc50	1512	1063	1231	1322
Unc5b	732	739	793	568
Unc80	0	11	3	0
Unc93b1	1884	2948	2532	5332
Ung	709	801	591	571
Unk	208	185	208	169
Unkl	340	263	337	291
Uox	1585	654	269	53
Upf1	437	715	416	333
Upf2	563	695	815	753
Upf3a	610	1020	837	692
Upf3b	601	415	493	623
Upk1a	49	60	33	14
Upk1b	703	199	910	339
Upk3bl	16	2	6	21
Upp1	691	1894	767	1275
Uprt	19	53	39	68
Uqcc1	1104	1487	1225	1331
Uqcc2	1212	1083	1020	1474
Uqcc3	1042	1155	1148	1175
Uqcr10	2413	2642	1107	1034
Uqcr11	5983	5557	3421	3210
Uqcrb	5452	3100	5641	4477
Uqcrc1	5956	6299	3919	5108
Uqcrc2	3948	4264	3753	4604
Uqcrfs1	6950	7030	6602	7612
Uqcrh	13880	10641	9965	12234
Uqcrq	5253	5287	4309	3568
Urb1	224	286	242	206
Urb2	198	216	218	230
Urgcp	347	581	256	382
Uri1	1379	936	1010	1045
Urm1	1180	1217	1118	1003
Urod	2152	2238	2058	2150
Uros	314	330	379	341
Usb1	403	313	339	222
Use1	1652	2163	1345	1434
Usf2	1452	1604	1404	1778
Usf3	757	680	942	1005
Ush1c	82	9	2	14
Ush1g	31	10	21	8
Ush2a	6	0	13	33
Ushbp1	59	210	125	96
Usmg5	556	165	521	824
Uso1	2452	4341	3439	4391
Usp1	667	728	742	651

Usp10	1136	1266	906	837
Usp11	337	629	390	377
Usp12	40	19	32	31
Usp13	2274	1651	1226	1910
Usp14	1718	1241	987	1326
Usp15	1514	1097	1595	1592
Usp16	1013	986	1105	1287
Usp18	677	397	394	1244
Usp19	1468	1837	1508	1110
Usp2	1392	2092	793	719
Usp20	461	767	602	338
Usp21	238	91	213	193
Usp22	14	69	44	43
Usp24	1831	1590	1473	1631
Usp25	31	79	80	25
Usp27x	79	77	114	92
Usp28	885	1298	1022	793
Usp29	248	60	156	128
Usp3	1178	796	1004	976
Usp30	349	439	269	341
Usp31	60	52	87	41
Usp32	335	477	597	386
Usp33	570	420	450	605
Usp34	454	768	655	541
Usp35	96	135	77	108
Usp36	891	524	664	466
Usp37	433	488	486	370
Usp38	379	351	670	450
Usp39	1318	1225	1163	1155
Usp40	918	882	764	986
Usp42	446	291	458	472
Usp43	0	20	29	0
Usp45	90	135	102	85
Usp46	875	649	797	746
Usp47	2889	3664	3436	3308
Usp48	44	41	60	0
Usp49	35	58	41	38
Usp5	22	129	27	38
Usp53	302	207	250	273
Usp54	107	130	179	86
Usp6nl	718	751	912	715
Usp7	2918	3315	3536	3073
Usp8	973	896	888	890
Usp9x	2088	2349	2445	2804
Uspl1	738	619	639	556
Ust	124	116	126	125
Utp11	1105	1178	1276	1580

Utp14a	945	1083	1216	1052
Utp15	680	570	516	556
Utp18	622	630	497	801
Utp20	588	721	639	512
Utp23	530	431	447	419
Utp3	2255	1968	2437	1874
Utp4	1262	1218	1249	1274
Utp6	1679	1790	1629	1696
Utrn	1266	873	1080	814
Uvrag	827	698	775	867
Uvssa	75	118	102	67
Uxs1	1160	1465	1301	1662
Vac14	1042	1213	1025	1140
Vamp1	590	470	295	425
Vamp2	3563	2817	3217	2765
Vamp3	4384	4220	4351	4519
Vamp4	867	622	922	1163
Vamp5	631	792	390	429
Vamp7	623	716	615	721
Vamp8	3793	3106	3239	3488
Vangl1	1237	907	1153	787
Vangl2	866	194	1154	310
Vapa	6569	5302	5447	6626
Vapb	2324	2917	1974	2548
Vars	848	1424	966	817
Vars2	350	353	373	307
Vash1	674	1175	990	1474
Vash2	23	1631	468	175
Vasn	354	732	432	765
Vasp	3704	4438	4942	5587
Vat1	3674	9529	9775	14748
Vat1l	19	18	59	27
Vav1	192	286	257	541
Vav2	416	603	366	329
Vav3	1458	871	1106	702
Vax2	2	4	6	8
Vbp1	188	396	107	119
Vcam1	240	557	305	606
Vcan	2449	7717	4414	7326
Vcl	2541	2904	3196	2930
Vcp	12372	16300	14799	14283
Vcpip1	362	276	420	484
Vdac1	8633	6435	5946	5924
Vdac2	11266	10011	11619	13208
Vdac3	11925	10102	9249	11383
Vdr	4583	1274	3334	1373
Vegfa	1059	1177	1107	907

Vegfb	1220	1036	1210	1837
Vegfc	161	216	161	281
Vezf1	1045	1084	1166	1236
Vezt	272	172	294	140
Vgll2	573	390	504	454
Vgll3	431	199	331	705
Vgll4	1446	1260	1904	745
Vhl	874	702	701	1059
Vil1	9	0	14	0
Vill	70	6	59	0
Vim	41562	111619	83998	159002
Vimp	2886	3976	3391	4411
Vipas39	1326	1236	1310	1609
Vipr1	50	78	38	37
Vipr2	146	98	108	130
Vit	21	19	9	0
Vkorc1	2865	3308	3203	5150
Vldlr	1064	1196	1204	1239
Vma21	41	0	15	12
Vmac	531	897	635	1182
Vmo1	19	7	20	26
Vmp1	5566	4638	4774	7681
Vnn1	276	267	260	386
Vof16	21	0	57	40
Vom2r44	10	7	18	6
Vom2r60	159	237	101	110
Vopp1	834	1054	984	955
Vps11	1210	1258	1183	1443
Vps13a	520	347	438	308
Vps13b	743	711	820	869
Vps13c	1056	954	818	1479
Vps13d	1149	1492	1347	1297
Vps16	825	1170	912	1151
Vps18	534	542	319	502
Vps25	2819	2952	3557	3228
Vps26a	2602	2588	2677	2671
Vps26b	1943	1915	2079	2372
Vps28	2989	3057	2573	2461
Vps29	2052	2564	2031	2617
Vps33a	833	872	724	910
Vps33b	585	502	551	470
Vps35	2373	2026	2589	3107
Vps36	2243	2134	2805	2370
Vps37a	621	385	570	495
Vps37b	365	751	429	296
Vps37d	10	37	42	18
Vps39	839	904	1031	1112

Vps41	2101	1521	1559	2098
Vps45	845	1097	940	1107
Vps4a	1988	2330	2351	2787
Vps4b	1509	1152	1169	1569
Vps50	27	47	69	43
Vps52	1117	1426	916	973
Vps53	1330	1036	1044	1076
Vps54	1644	1144	1485	2124
Vps72	1035	1382	1094	895
Vps8	778	841	758	961
Vps9d1	900	1104	859	1153
Vrk1	596	675	656	469
Vrk2	582	804	542	553
Vrk3	1446	1299	1470	1467
Vsig10	627	603	673	671
Vsig10l	754	611	694	796
Vsig2	13	19	5	13
Vsig4	0	19	45	40
Vsig8	162	14	269	30
Vsir	2375	3588	3934	6252
Vsnl1	1324	387	402	170
Vstm4	534	453	602	801
Vstm5	18	0	3	0
Vta1	1398	1372	1316	1375
Vtcn1	98	46	42	18
Vti1a	236	251	122	191
Vti1b	1087	1064	748	1278
Vtn	374	394	615	649
Vwa1	4142	5671	4178	2906
Vwa2	6	11	9	0
Vwa3b	85	7	39	58
Vwa5b2	126	142	117	57
Vwa7	67	45	53	36
Vwa9	699	663	630	716
Vwf	776	2303	1611	1194
Wac	169	278	307	407
Wapl	2259	1379	2033	2203
Wars	1794	2760	1846	2666
Wars2	468	488	519	424
Was	611	489	624	1163
Wasf1	79	73	116	111
Wasf2	2737	2027	2203	2573
Wash1	592	743	644	609
Wasl	973	746	784	833
Wbp1l	2402	3523	2499	2859
Wbp2	1923	2237	2070	2222
Wbp4	883	602	821	755

Wbscr17	467	633	265	384
Wbscr22	828	1159	885	839
Wbscr27	96	84	80	113
Wdcp	169	117	101	118
Wdfy1	1249	1321	1559	1974
Wdfy2	1538	1236	1360	1109
Wdfy4	368	332	391	649
Wdhd1	224	366	289	228
Wdpcp	204	166	167	232
Wdr1	8664	8647	9119	8641
Wdr11	981	642	862	1121
Wdr12	1025	994	1107	820
Wdr13	1245	833	1243	1130
Wdr17	5	0	0	6
Wdr18	432	775	385	365
Wdr19	68	28	26	19
Wdr20	301	428	379	335
Wdr24	271	346	241	207
Wdr25	177	119	200	121
Wdr27	82	63	66	12
Wdr3	992	785	809	981
Wdr31	165	96	135	69
Wdr33	1675	986	1336	1532
Wdr34	392	213	253	212
Wdr35	273	291	439	282
Wdr37	352	266	399	335
Wdr4	191	288	168	146
Wdr41	609	772	581	830
Wdr43	1094	1252	1235	973
Wdr44	66	128	56	108
Wdr45	1624	1063	1589	1274
Wdr45b	1583	1345	1246	1314
Wdr46	701	915	513	591
Wdr47	483	405	435	312
Wdr48	881	795	808	765
Wdr5	1043	1355	1189	950
Wdr53	458	425	337	385
Wdr55	436	286	367	241
Wdr59	48	168	87	129
Wdr5b	209	190	214	297
Wdr6	1157	843	1510	1225
Wdr60	162	102	93	61
Wdr61	1566	1287	1459	1643
Wdr66	102	18	54	17
Wdr7	511	307	445	555
Wdr70	814	652	533	621
Wdr73	425	297	230	345

Wdr74	1215	1231	1112	1212
Wdr75	1468	1354	1405	1198
Wdr76	31	31	35	30
Wdr78	125	97	80	185
Wdr81	830	1033	882	1075
Wdr82	1403	1461	1348	1324
Wdr83	633	480	708	485
Wdr83os	3788	4114	3817	3145
Wdr86	17	6	18	31
Wdr89	77	202	144	97
Wdr92	304	225	161	185
Wdr97	7	8	3	0
Wdsub1	375	312	328	222
Wdtc1	756	823	609	491
Wdyhv1	552	306	493	330
Wee1	567	459	375	262
Wfdc1	433	997	412	243
Wfdc2	79	164	90	130
Wfdc3	24	37	9	15
Wfdc5	318	65	150	115
Wfikkn2	44	175	33	37
Wfs1	632	671	554	687
Whamm	314	337	339	239
Whrn	52	100	39	56
Whsc1	471	996	595	758
Wif1	110	153	104	101
Wipf1	421	733	818	1087
Wipf2	14	23	65	30
Wipf3	307	177	167	158
Wipi1	1255	1382	1271	1937
Wipi2	1553	1380	1493	1227
Wisp1	15	44	35	42
Wisp2	1841	1583	2185	2442
Wisp3	10	0	0	26
Wiz	749	579	530	525
Wls	2471	2730	3330	3255
Wnk1	434	1701	1255	697
Wnk2	315	172	144	260
Wnk3	8	11	32	11
Wnk4	601	190	506	185
Wnt10a	1157	610	599	230
Wnt10b	9	11	21	34
Wnt11	181	140	170	122
Wnt16	179	63	92	392
Wnt2	309	841	268	161
Wnt2b	23	47	42	58
Wnt3	449	290	212	79

Wnt3a	66	100	53	29
Wnt4	1235	997	1029	1110
Wnt5a	754	1231	888	1024
Wnt5b	131	46	126	233
Wnt6	178	56	84	92
Wnt9a	268	303	439	297
Wnt9b	10	23	5	0
Wrap53	317	316	322	259
Wrap73	314	385	149	168
Wrb	438	739	507	612
Wrn	399	357	438	350
Wrnip1	499	596	561	718
Wsb1	1629	1045	2296	1238
Wsb2	4147	4557	3424	4749
Wscd1	93	155	123	70
Wscd2	14	21	12	38
Wt1	0	19	5	32
Wtap	2646	1880	2186	2905
Wtip	278	506	339	446
Wwc1	263	196	230	138
Wwc2	274	284	375	413
Wwc3	771	689	1159	1121
Wwox	230	253	141	127
Wwp1	1121	680	981	1089
Wwp2	616	636	676	514
Wwtr1	1806	1801	1815	2022
Xab2	670	698	578	483
XAF1	195	342	287	271
Xbp1	4984	4977	4756	7033
Xcl1	41	4	18	24
Xcr1	179	39	96	281
Xdh	10399	9960	8743	14110
Xiap	452	569	507	544
Xirp1	4071	6069	3519	2654
Xirp2	21766	14302	10514	12816
Xk	87	47	68	47
Xkr5	28	71	62	48
Xkr6	29	0	0	1
Xkr7	6	19	3	0
Xkrx	159	66	86	35
Xpa	726	401	642	412
Xpc	452	554	537	486
Xpnpep1	2132	3387	2916	3044
Xpnpep2	171	128	348	112
Xpnpep3	21	73	50	38
Xpo1	2303	2249	2424	2124
Xpo4	789	964	1001	740

Xpo5	173	448	267	226
Xpo6	1652	1885	1751	1534
Xpo7	1731	1418	1681	1636
Xpot	1692	1322	1375	1770
Xpr1	47	24	8	44
Xrcc1	1686	1553	1255	1176
Xrcc2	73	119	56	61
Xrcc3	116	207	80	118
Xrcc4	200	118	199	179
Xrcc5	1056	1468	1320	1382
Xrcc6	892	946	844	1186
Xrn1	516	444	540	607
Xrn2	992	1373	1324	1359
Xxylt1	804	1236	835	1347
Xylb	663	477	829	695
Xylt1	23	46	50	40
Xylt2	535	785	513	588
Yae1d1	741	715	691	936
Yaf2	864	589	578	577
Yap1	5266	3197	4020	3140
Yars	2077	2963	1992	1935
Yars2	174	195	155	147
Ybey	128	74	86	47
Ybx1	828	509	96	86
Ybx1-ps3	4653	4668	2210	3182
Ybx2	582	586	456	804
Ybx3	12115	9087	8296	7678
Ydjc	71	65	45	86
Yeats2	721	677	928	607
Yeats4	1336	1205	1166	1643
Yes1	842	972	931	958
Yif1a	963	1054	712	1044
Yipf1	864	943	993	1454
Yipf2	1897	1943	2231	1850
Yipf4	302	214	239	196
Yipf5	2920	2547	2704	3263
Yipf6	726	484	716	850
Yipf7	514	233	230	339
Ykt6	3614	4955	4959	5766
Ylpm1	1146	918	1103	1190
Ypel1	24	69	27	41
Ypel2	411	285	761	821
Ypel3	2028	1786	2415	2042
Ypel4	352	181	354	309
Ypel5	2549	1879	2025	2255
Yrdc	1080	957	1112	1151
Ythdc1	1623	795	975	1013

Ythdc2	89	64	87	39
Ythdf1	1888	1675	1902	1743
Ythdf2	1118	828	906	1085
Ythdf3	879	708	818	882
Ywhab	8482	8987	9313	10506
Ywhae	13690	13183	14113	13410
Ywhag	8377	6934	6069	7868
Ywhah	7298	6158	5880	9188
Ywhaq	11947	4182	6011	8042
Ywhaz	22095	12573	18380	15185
Yy1	1211	904	972	893
Zadh2	996	758	927	812
Zak	9298	5184	6256	5235
Zan	48	14	38	13
Zap70	87	10	63	39
Zbed3	466	675	898	867
Zbed4	148	131	116	108
Zbed5	54	83	66	53
Zbed6	3673	3020	2798	2421
Zbp1	177	35	57	183
Zbtb1	131	217	385	211
Zbtb10	0	9	47	118
Zbtb11	287	174	202	311
Zbtb11os1	238	166	263	221
Zbtb12	50	54	147	96
Zbtb16	1645	2539	803	2037
Zbtb17	432	514	391	356
Zbtb2	300	310	320	465
Zbtb20	52	75	26	45
Zbtb22	749	837	864	852
Zbtb24	273	133	170	233
Zbtb25	245	396	450	214
Zbtb26	253	238	236	222
Zbtb3	111	71	119	87
Zbtb32	28	8	9	0
Zbtb34	0	16	18	0
Zbtb37	28	27	9	0
Zbtb38	270	391	384	367
Zbtb39	378	258	340	310
Zbtb4	1457	1244	1688	1630
Zbtb40	323	345	343	312
Zbtb41	240	129	224	290
Zbtb42	642	647	578	511
Zbtb43	257	246	345	272
Zbtb44	465	312	495	324
Zbtb45	409	198	323	385
Zbtb46	74	226	102	97

Zbtb47	575	769	468	723
Zbtb48	105	170	98	78
Zbtb49	52	86	54	23
Zbtb5	441	438	384	383
Zbtb7a	1518	1335	1231	1153
Zbtb7b	160	298	117	151
Zbtb7c	417	216	375	130
Zbtb8a	159	309	244	173
Zbtb8os	1585	1392	1447	1659
Zbtb9	193	243	188	270
Zc2hc1a	583	167	370	294
Zc2hc1c	21	51	24	0
Zc3h10	420	347	450	482
Zc3h12a	592	467	444	490
Zc3h12c	47	18	77	21
Zc3h12d	260	104	190	366
Zc3h13	394	450	403	366
Zc3h14	908	944	1073	1562
Zc3h15	1039	484	438	551
Zc3h18	377	590	468	386
Zc3h3	256	317	251	170
Zc3h4	1345	1255	1446	1525
Zc3h6	89	120	153	135
Zc3h7a	883	818	1270	723
Zc3h7b	1529	2026	2452	2417
Zc3h8	326	298	360	137
Zc3hav1	2018	2128	1994	2193
Zc3hav1l	63	154	104	199
Zc3hc1	593	771	648	588
Zc4h2	151	184	196	294
Zcchc10	104	200	166	279
Zcchc11	90	168	155	148
Zcchc12	21	31	23	87
Zcchc14	153	213	178	205
Zcchc17	1185	1433	1097	1239
Zcchc2	185	130	159	261
Zcchc24	1229	1556	1625	2301
Zcchc3	187	210	331	426
Zcchc4	260	152	191	138
Zcchc5	34	143	3	48
Zcchc6	1761	1663	1842	1946
Zcchc7	790	1138	1482	800
Zcchc8	538	645	570	614
Zcchc9	589	732	648	420
Zcrb1	1473	1916	1264	1513
Zcwpw2	26	104	48	51
Zdbf2	16	269	107	50

Zdhhc12	494	437	485	375
Zdhhc13	528	456	546	487
Zdhhc14	209	416	295	534
Zdhhc15	84	170	215	209
Zdhhc16	1123	1278	1059	1347
Zdhhc17	280	444	551	350
Zdhhc18	821	1537	1070	1216
Zdhhc19	0	1	3	0
Zdhhc2	278	490	381	317
Zdhhc20	345	215	403	368
Zdhhc21	47	49	51	79
Zdhhc23	92	112	93	98
Zdhhc24	340	332	336	253
Zdhhc3	3840	3363	3425	2793
Zdhhc4	478	675	447	325
Zdhhc5	1054	1599	1453	1511
Zdhhc6	1064	1194	985	1458
Zdhhc7	777	1129	706	1037
Zdhhc8	349	527	308	359
Zdhhc9	976	938	1115	1350
Zeb1	757	1058	615	657
Zeb2	867	859	1213	1784
Zeb2os	24	22	81	140
Zer1	499	602	468	375
Zfand1	279	215	265	265
Zfand2a	1379	1848	1532	2156
Zfand2b	625	650	468	382
Zfand3	1384	2006	1690	2014
Zfand4	112	190	89	92
Zfand5	3713	1837	3145	3457
Zfand6	1598	1036	933	1840
Zfat	264	437	382	578
Zfc3h1	1474	1049	1408	1120
Zfhx2	95	59	81	105
Zfhx3	136	197	206	241
Zfhx4	132	187	161	282
Zfp1	265	332	304	253
Zfp105	585	464	551	491
Zfp106	7078	5149	4915	5822
Zfp11	192	88	104	177
Zfp110	843	872	940	852
Zfp111	199	353	176	295
Zfp112	53	31	69	59
Zfp113	34	30	71	26
Zfp12	201	208	266	126
Zfp128	596	601	728	760
Zfp131	321	366	376	228

Zfp133	498	312	363	452
Zfp136	24	50	26	43
Zfp141	4	12	18	13
Zfp142	490	686	554	584
Zfp143	245	345	304	488
Zfp148	1094	936	1005	909
Zfp157	212	190	260	271
Zfp161	285	294	304	448
Zfp167	200	226	184	149
Zfp174	25	20	9	149
Zfp18	670	690	651	593
Zfp180	949	601	874	879
Zfp182	155	137	241	134
Zfp184	121	78	123	66
Zfp185	814	394	679	597
Zfp189	462	162	378	540
Zfp192	240	120	181	135
Zfp2	7	7	35	0
Zfp202	265	321	238	282
Zfp207	2749	3076	2765	2870
Zfp212	837	629	828	713
Zfp217	922	566	1125	1028
Zfp219	1227	820	1338	886
Zfp236	258	205	259	235
Zfp238	226	267	290	206
Zfp24	807	658	868	765
Zfp251	337	377	388	497
Zfp259	1556	1825	1477	1980
Zfp26	412	257	305	358
Zfp263	498	293	310	477
Zfp266	1412	1075	1997	831
Zfp275	254	293	283	452
Zfp276	255	416	153	340
Zfp277	813	596	582	617
Zfp28	78	113	138	235
Zfp280c	125	70	153	108
Zfp280d	455	288	466	321
Zfp281	290	287	248	330
Zfp282	908	538	676	384
Zfp286a	27	26	21	27
Zfp287	154	74	190	137
Zfp292	407	208	489	293
Zfp295	312	316	295	261
Zfp296	91	79	45	16
Zfp3	128	246	164	262
Zfp316	194	125	159	155
Zfp317	208	117	144	220

Zfp318	456	488	525	282
Zfp319	1	8	6	0
Zfp322a	244	209	329	235
Zfp324	112	203	265	114
Zfp326	95	61	83	79
Zfp329	148	21	137	90
Zfp330	1061	848	900	1100
Zfp334	69	63	156	269
Zfp335	742	844	638	642
Zfp341	398	239	209	191
Zfp346	405	417	503	255
Zfp347	260	157	137	162
Zfp35	136	97	227	200
Zfp354a	107	113	135	115
Zfp354c	16	42	44	93
Zfp358	651	623	537	668
Zfp36	4004	2964	4855	3207
Zfp362	375	374	462	350
Zfp365	37	18	57	32
Zfp366	0	9	12	0
Zfp367	77	170	123	114
Zfp36l1	1481	1377	1694	1885
Zfp382	0	61	20	32
Zfp384	1072	1194	1422	1646
Zfp385a	922	1870	877	655
Zfp385d	41	73	89	22
Zfp386	1017	792	1034	1166
Zfp39	38	44	92	51
Zfp394	617	475	436	638
Zfp395	352	396	495	521
Zfp397	140	96	122	142
Zfp398	53	35	18	62
Zfp40	100	42	32	61
Zfp407	203	273	197	163
Zfp41	288	315	263	263
Zfp414	242	295	245	248
Zfp418	90	7	65	50
Zfp422	781	608	831	501
Zfp423	56	126	51	35
Zfp426	283	279	357	318
Zfp428	80	180	93	72
Zfp438	93	117	95	86
Zfp444	310	421	357	393
Zfp445	1143	672	1071	964
Zfp449	133	202	179	101
Zfp451	614	643	664	607
Zfp458	31	26	17	17

Zfp46	661	956	653	856
Zfp462	194	174	190	378
Zfp467	251	277	304	368
Zfp469	272	431	346	506
Zfp472	134	118	215	176
Zfp496	944	1010	979	945
Zfp498	9	15	21	17
Zfp503	314	258	429	413
Zfp507	6	2	15	17
Zfp51	24	60	39	8
Zfp511	480	563	400	456
Zfp512	765	776	563	905
Zfp513	57	252	71	138
Zfp516	488	315	593	490
Zfp518a	91	137	68	152
Zfp518b	280	156	209	215
Zfp52	45	57	21	109
Zfp521	284	689	462	722
Zfp523	384	424	367	394
Zfp524	382	478	352	374
Zfp526	10	25	0	7
Zfp53	86	46	24	87
Zfp532	841	647	785	631
Zfp536	14	60	9	0
Zfp560	141	129	169	300
Zfp563	115	62	77	196
Zfp566	32	51	41	12
Zfp57	25	20	11	22
Zfp574	294	262	208	184
Zfp579	151	276	262	299
Zfp583	9	47	18	16
Zfp592	654	921	808	621
Zfp593	488	695	381	254
Zfp598	896	1540	802	963
Zfp605	42	30	17	31
Zfp606	131	80	60	213
Zfp608	391	270	427	454
Zfp609	719	542	758	661
Zfp61	574	495	436	517
Zfp612	646	370	509	162
Zfp617	276	246	272	291
Zfp618	236	196	326	158
Zfp62	315	269	346	324
Zfp628	297	163	251	315
Zfp629	1688	1163	1437	1609
Zfp637	1764	1501	1696	1508
Zfp638	984	851	1235	1231

Zfp639	557	400	301	548
Zfp64	187	259	111	122
Zfp641	233	136	68	191
Zfp644	567	494	415	431
Zfp646	307	694	461	503
Zfp652	152	168	98	181
Zfp653	109	141	83	96
Zfp654	283	92	184	146
Zfp655	204	364	236	239
Zfp667	287	133	236	154
Zfp668	186	233	196	148
Zfp672	64	49	94	161
Zfp68	873	497	715	890
Zfp689	125	185	53	267
Zfp69	45	63	50	49
Zfp691	313	423	305	386
Zfp692	1813	1124	1372	1019
Zfp703	1287	1583	1363	959
Zfp704	41	45	68	44
Zfp706	4387	2297	2597	3674
Zfp709	225	139	229	201
Zfp710	436	669	686	918
Zfp717	617	344	408	386
Zfp746	538	688	694	665
Zfp748	346	152	257	363
Zfp758	144	89	71	126
Zfp763	178	124	156	126
Zfp770	126	99	164	53
Zfp771	151	178	101	44
Zfp772	596	515	620	406
Zfp773-ps1	0	20	2	0
Zfp775	338	355	304	301
Zfp777	137	230	152	198
Zfp780b	237	105	199	250
Zfp786	53	30	51	50
Zfp787	356	302	545	317
Zfp799	151	133	129	163
Zfp800	43	58	203	56
Zfp821	570	538	408	380
Zfp827	196	298	211	237
Zfp830	282	307	408	336
Zfp839	629	595	329	442
Zfp84	495	558	516	496
Zfp846	349	325	393	300
Zfp853	53	39	23	59
Zfp862	101	143	181	225
Zfp865	159	211	214	273

Zfp867	137	95	144	62
Zfp868	1819	1085	1496	1691
Zfp870	181	181	178	176
Zfp879	25	34	38	3
Zfp9	55	136	110	109
Zfp90	226	248	280	266
Zfp91	3012	2637	2925	3364
Zfp93	62	55	63	19
Zfp94	55	64	75	97
Zfp945	35	12	33	36
Zfp949	79	103	56	30
Zfp952	94	113	81	199
Zfp955a	262	238	199	424
Zfp958	575	282	265	315
Zfp964	44	34	41	17
Zfp978	133	115	161	136
Zfpl1	611	907	763	790
Zfpm1	196	180	120	182
Zfpm2	13	11	33	50
Zfr	1220	968	1013	954
Zfr2	40	68	62	64
Zfx	501	547	657	759
Zfyve1	573	469	549	710
Zfyve16	215	288	361	367
Zfyve19	289	276	317	317
Zfyve21	1010	749	736	785
Zfyve26	576	533	454	514
Zfyve27	833	807	826	710
Zfyve28	1	35	24	0
Zfyve9	570	437	639	429
Zglp1	9	4	12	62
Zgrf1	99	83	95	82
Zhx1	358	450	633	461
Zhx2	721	719	748	549
Zhx3	526	747	752	712
Zic1	95	112	41	270
Zim1	24	0	26	18
Zkscan1	523	400	471	366
Zkscan3	562	618	709	760
Zkscan4	151	110	102	221
Zkscan5	560	592	615	575
Zmat1	88	135	129	105
Zmat2	3519	3138	3399	3253
Zmat3	531	681	438	676
Zmat5	338	343	258	370
Zmiz1	2626	2438	3056	3606
Zmpste24	1105	1290	889	1341

Zmym1	315	312	227	383
Zmym2	1293	700	1426	1125
Zmym3	609	658	844	648
Zmym4	569	452	450	635
Zmym5	1412	1280	1419	1102
Zmym6	211	246	311	264
Zmynd10	83	131	77	66
Zmynd11	1518	1080	1315	1128
Zmynd12	3	0	8	13
Zmynd15	162	147	171	116
Zmynd19	56	175	110	110
Zmynd8	1187	1338	1318	1137
Znf235	11	72	45	41
Znf354b	9	60	6	10
Znf408	127	202	181	193
Znf48	449	411	564	658
Znf740	532	284	558	38
Znf750	242	211	233	79
Znf768	390	557	454	465
Znfx1	317	594	316	482
Znhit1	678	868	600	612
Znhit2	204	400	182	256
Znhit3	453	372	337	517
Znhit6	934	1000	945	949
Znrd1	1171	1073	1130	1158
Znrd1as1	287	233	290	291
Znrf1	1567	1570	1192	1804
Znrf2	1283	700	1506	1260
Zpbp	5	13	0	20
Zpbp2	63	56	102	55
Zranb1	1058	707	1390	869
Zranb2	1381	1029	1136	1273
Zranb3	173	99	205	135
Zrsr1	83	155	66	67
Zrsr2	225	312	337	200
Zscan12	525	403	372	625
Zscan2	53	50	71	63
Zscan20	204	249	202	155
Zscan21	565	532	552	481
Zscan26	503	392	510	576
Zscan30	310	216	290	177
Zswim1	463	267	426	198
Zswim3	314	332	336	299
Zswim4	425	555	515	472
Zswim5	41	33	45	14
Zswim6	76	142	111	188
Zswim7	136	163	86	99

Zufsp	625	702	754	654
Zw10	338	588	704	475
Zwilch	107	233	205	238
Zwint	1404	1256	1447	1193
Zxdb	269	72	256	136
Zxdc	929	576	736	807
Zyg11b	217	358	280	344
Zyx	2393	4354	2334	3290
Zzef1	457	652	740	701
Zzz3	696	855	805	743
